# Diastereoselective synthesis of atropisomeric pyrazolyl pyrrolo[3,4-*d*]isoxazolidines *via* pyrazolyl nitrone cycloaddition to facially divergent maleimides: intensive NMR and DFT studies†

**DOI:** 10.1039/c9ra10039c

**Published:** 2020-01-06

**Authors:** Awad I. Said, Talaat I. El-Emary

**Affiliations:** Chemistry Department, Faculty of Science, Assiut University Assiut Egypt 71516 awadsaid@aun.edu.eg +20-1012991716

## Abstract

A pyrazolyl nitrone (2) underwent 1,3-dipolar cycloadditions to afford some *N*-substituted maleimides (3a–o). An atropisomeric character was introduced into the formed cycloadducts by using maleimides that have a restricted rotation around the C–N bond. Also, facial selectivity of both *endo* and *exo* cycloaddition was observed where the major atropisomer was one that is formed by attacking the nitrone from the less hindered face of the dipolarophile. On the other hand, maleimides with free rotation around the C–N bond led to *endo* and *exo* cycloadducts without atropisomerism. The presence of atropisomerism in the formed cycloadducts was confirmed by extensive NMR studies and DFT calculations.

## Abstract 2

Diastereoselective pyrazole-based atropisomeric cycloadducts were formed by cycloaddition of a pyrazole-based nitrone and maleimides with restricted rotation around C–N bond caused by bulk *ortho* substitution.

## Graphical abstract

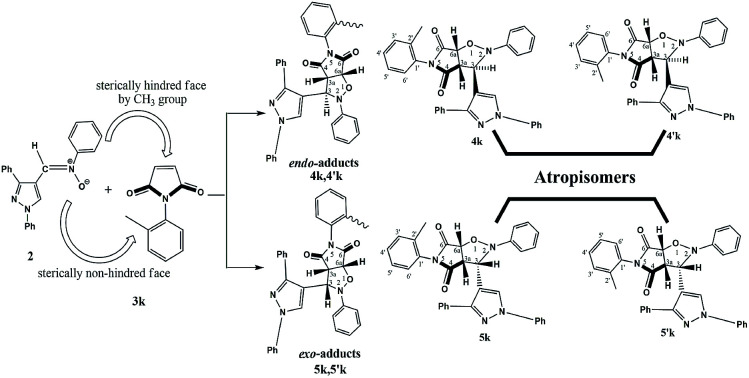

## Introduction

Chemistry of nitrones has received significant attention since its discovery, more than a century ago.^1^ Most of the studies have focused on exploring the 1,3-dipole character of nitrones in [3 + 2] dipolar cycloadditions with dipolarophiles to construct functionalized five-membered heterocycles which are important intermediates in organic synthesis.^2^ The dipolarophiles include alkenes,^3^ alkynes,^4^ allenes^5^ and other cumulated double bonds.^6^ Isoxazolidines, formed from the [3 + 2] dipolar cycloadditions of nitrones and alkenes, exhibit good biological activities^7^ and the possibility of their transformation *via* ring opening into open-chain derivatives^8^ makes them valuable for the synthesis of natural and biologically important compounds such as amino sugars, amino alcohols, alkaloids, β-lactams, and amino acids.^9^ Also, pyrazoles have received significant attention because they are widely used as core motifs for a large number of compounds in various applications such as agrochemicals and medicine, due to their broad range of biological activities.^10^

Several computational studies have been carried out to understand the origins of the regio- and stereoselectivities of cycloaddition reactions.^11^ The stereochemical course of the 1,3-dipolar cycloaddition reaction has been well explained in terms of secondary orbital interactions, steric factors, H-atom bonding and/or dipole–dipole electrostatic interactions.

Atropisomerism^12^ is a well-known phenomenon resulting from hindered rotation about single bonds where the energy barrier to rotation is high enough to allow the isolation of the conformers. Atropisomeric compounds have several applications including in the design of bioactive molecules, and construction of molecular switches and motors.^13^ To the best of our knowledge, little work has been reported about the effect of single bond restricted rotation on the route of cycloaddition reactions and the formation of atropisomeric cycloadducts.^14^ In most of the reported cycloaddition reactions, the used reactants have no restricted rotation near the cycloaddition region of the molecules and the maximum number of cycloadducts was known to be two isomers, namely *exo* and *endo* isomers.

In this work, pyrazole-based nitrone 2 underwent 1,3-dipolar cycloaddition with a series of and *N*-substituted maleimides (3a–o) (as dipolarophiles) with different degrees of rotational restriction around the C–N single bond. The effect of restricted rotation around the C–N single bond on the route of cycloaddition has been studied.

## Results and discussion

**Scheme 1:**
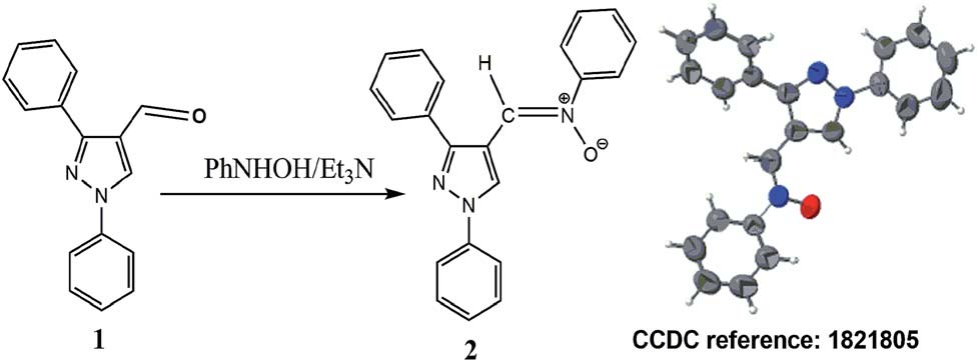
Synthesis of nitrone 2.

Nitrone 2 was prepared by reacting 1,3-diphenyl-1*H*-pyrazole-4-carboxaldehyde (1)^15^ with *N*-phenylhydroxylamine^16^ (Scheme 1). Nitrone 2 was obtained in *Z*-form as was confirmed by spectral analysis and single-crystal crystallography.^17^

**Scheme 2:**
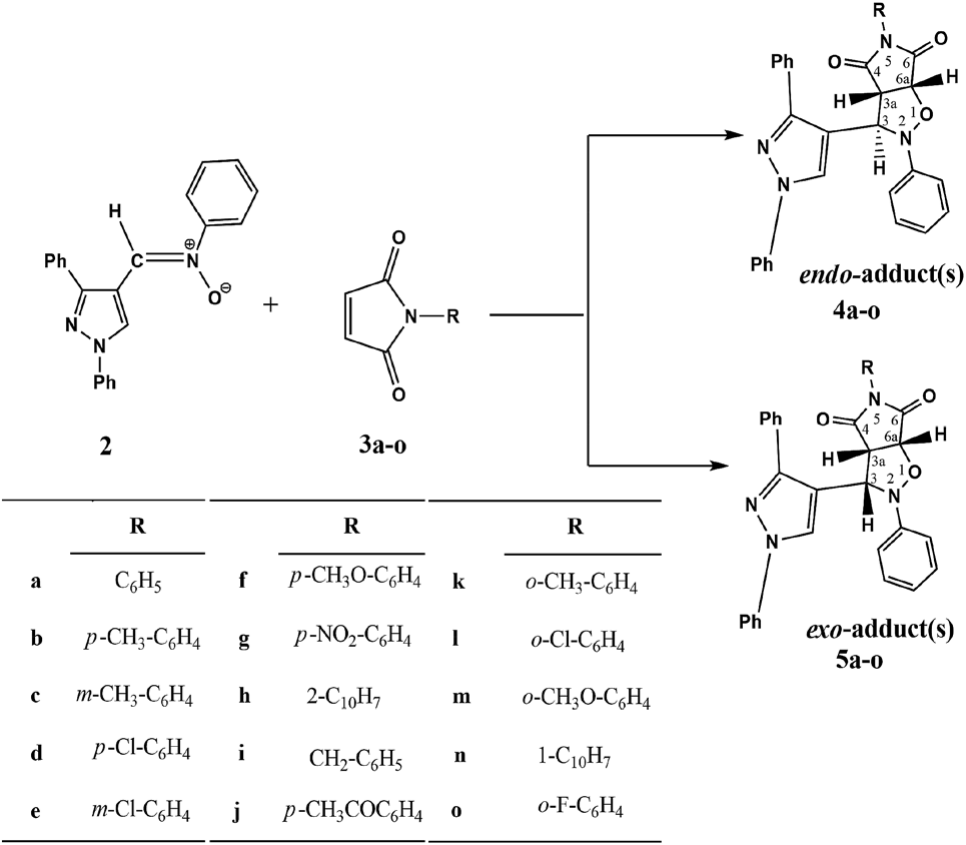
Cycloaddition reaction of nitrone 2 with dipolarophiles 3a–o.

Cycloadditions of 2 to *N*-substituted maleimides 3a–o (Scheme 2) were performed in toluene under reflux and were monitored by TLC. A high-resolution ^1^H-NMR spectrum (400 MHz) (ESI†) of the crude reaction products was measured to investigate the formed cycloadducts and estimate their ratio^18^ (Table 1). *Endo* and *exo* isomers were afforded by maleimides 3a–j at a ratio of 3.5–24 : 1. The majority of *endo* isomers was interpreted by the presence of a stabilizing secondary orbital interaction in the transition state leading to the *endo* product. This interaction is not present in the transition state of the *exo* product.^19^

**Table 1:** Total conversion, yield of *endo* and *exo* cycloadducts and their ratio for the cycloaddition of nitrone 2 and dipolarophiles 3a–o

Entry	Conversion^a^	*Endo* (%)		*Exo* (%)		*Endo* : *exo* ratio^b^
		Conversion^a^	Isolated^c^	Conversion^a^	Isolated^d^	
a	97	87	80	10	—	9 : 1
b	99	92	60	7	0.95	13.3 : 1
c	87	80	65	7	—	11.5 : 1
d	87	83.5	60	3.5	—	24 : 1
e	99	95	55	4	0.37	24 : 1
f	92	88	60	4	0.36	24 : 1
g	—	—	68	—	—	—
h	99	92	68	7	1.8	13.3 : 1
i	90	78	65	12	1.14	6.7 : 1
j	75	69	60	6	—	11.5 : 1
k	88	78	58	10	0.95	8.1 : 1
l	93	72.5	65	20.5	3.1	3.5 : 1
m	99	85	55	14	—	6.1 : 1
n	97	84	55	13	—	6.7 : 1
o	97	79.5	55	17.5	5.2	5.9 : 1

a Conversions were calculated from ^1^H-NMR integrations (400 MHz) as the conversion of the nitrone to the products.

b *Endo* : *exo* ratio was calculated from ^1^H-NMR integrations (400 MHz).

c Isolated *endo* isomer as obtained by filtration (first crop).

d Isolated *exo* isomer as obtained using preparative TLC of filtrate.

**Fig. 1:**
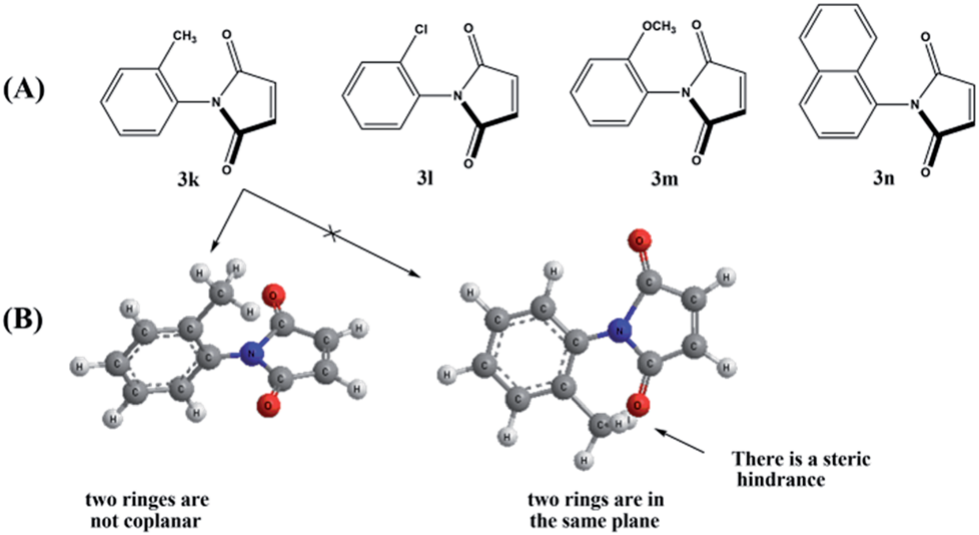
(A) The restricted rotation in dipolarophiles 3k–n. (B) The steric hindrance between the *ortho* substituent and carbonyl group forces the two rings to be not coplanar.

**Fig. 2:**
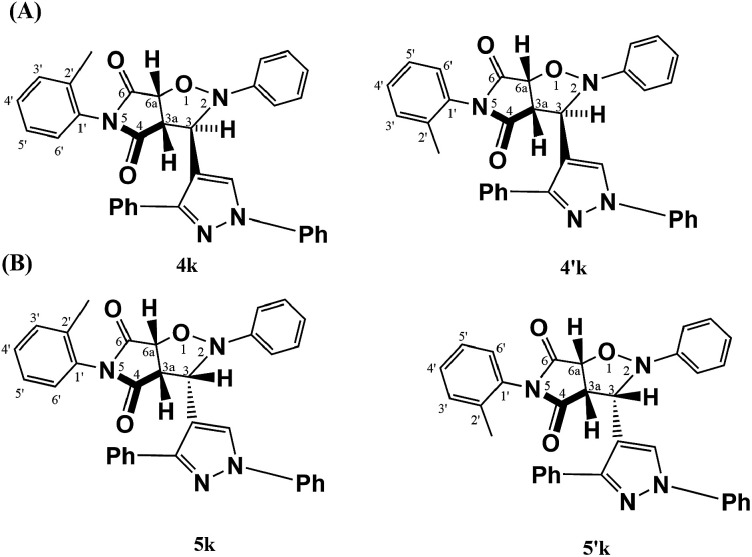
Four cycloadducts from the cycloaddition of nitrone 2 and dipolarophile 3k. (A) Cycloadducts 4k and 4′k were obtained by *endo* cycloaddition. (B) Cycloadducts 5k and 5′k were obtained by *exo* cycloaddition. 4k and 5k were formed by cycloaddition from the face anti to methyl group (lower TS potential energy) but 4′k and 5′k were formed from the face containing methyl group (higher TS potential energy).

On the other hand, maleimides 3k–n gave, strikingly, four cycloadducts as a result of the steric hindrance between the *ortho* substituent of the phenyl group and the carbonyl group of the maleimide moiety (Fig. 1) that forces phenyl and pyrrolidinedione rings to be tilted away (non-coplanar) to alleviate that steric hindrance.^20^ So, when the nitrone attacks the dipolarophile, whether the attack is *endo* or *exo*, the nitrone will find that the two faces are energetically different (divergent) and hence *endo* and *exo* atropisomers can be formed. The major *endo* or *exo* atropisomer is the product formed by attacking the nitrone through the dipolarophile face *anti* to the *ortho* substituent, where the transition state of the attack has lower energy (Fig. 2).

**Fig. 3:**
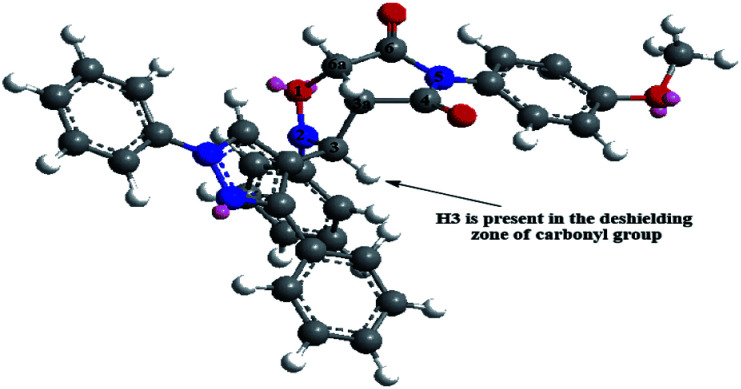
3D modeling of *endo* cycloadduct 4f from the cycloaddition of nitrone 2 and dipolarophile 3f.

The above mentioned effect of single bond restricted rotation on the route of the cycloaddition was confirmed using ^1^H-NMR and COSY spectra (ESI†). ^1^H-NMR spectra of *endo* isomer 4 formed from cycloaddition using any of dipolarophiles 3a–j showed only three signals in the region 3.8–6.2 ppm, a doublet, a doublet and a singlet corresponding to H3a, H6a and H3, respectively. On the other hand, ^1^H-NMR spectra of the crude reaction products showed in that region, in addition to the signals corresponding to *endo* isomer 4, three other signals: a triplet, doublet and doublet corresponding to H3a, H3 and H6a, respectively. The new signals correspond to the *exo* isomer 5 as was confirmed by ^1^H-NMR spectra of the separated *exo* isomers (ESI†). The yield and isomer ratio were determined from ^1^H-NMR integrations; *endo* and *exo* isomers were formed in conversions of 69–95% and 3–20%, respectively (Table 1). The unexpectedly higher *δ* value of H3 than H6a for *endo* isomer was ascribed to the presence of H3 in the deshielding zone of the carbonyl group as is shown in the optimized structures of *endo* and *exo* isomers (Fig. 3). But H3 in *exo* isomer is far from this deshielding zone.

**Fig. 4:**
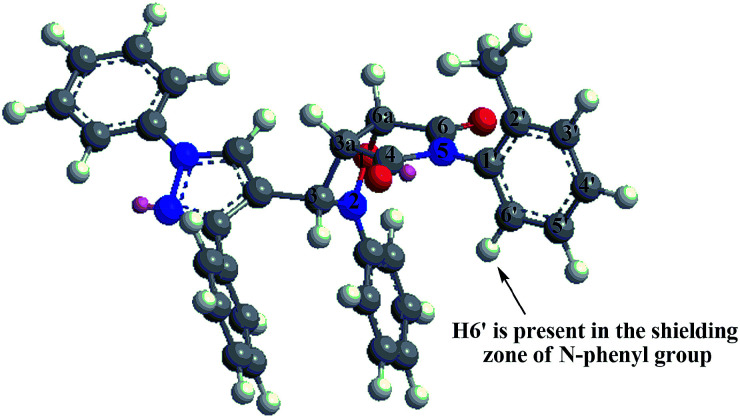
3D modeling of *endo* cycloadduct 4k from the cycloaddition of nitrone 2 and dipolarophile 3k.

On the other hand, ^1^H-NMR spectra of *endo* atropisomers formed from the cycloaddition using any of the dipolarophiles 3k–n showed signals in the region 3.8–6.2 ppm corresponding to seven hydrogens. For example, the ^1^H-NMR spectrum of *endo* atropisomers (4k, 4′k) formed using dipolarophile 3k (ESI†) showed two doublets at 4.06 ppm (*J* 7.32 Hz) and 4.09 ppm (*J* 7.32 Hz) corresponding to H3a of major and minor *endo* atropisomers (4k, 4′k), respectively; two doublets at 5.17 ppm (*J* 7.32 Hz) and 5.22 ppm (*J* 7.32 Hz) corresponding to H6a of major and minor *endo* atropisomers (4k, 4′k), respectively; two singlets at 5.71 ppm and 6.06 ppm corresponding to H3 of minor *endo* and major *endo* atropisomers (4′k, 4k), respectively; and a doublet at 5.68 ppm (*J* 8.08 Hz) with integration similar to that of any hydrogen of major *endo* isomer 4k, the origin of this signal being assigned to H6′. The lower *δ* value of H6′ for 4k was ascribed to shielding effect induced by the pyrazole ring as deduced from the optimized structure (Fig. 4).

Furthermore, ^1^H-NMR spectra of the crude reaction product from the cycloaddition using any of dipolarophiles 3k–n were measured (ESI†) and showed signals in the region 3.8–6.2 ppm, in addition to the signals corresponding to seven hydrogens of two *endo* atropisomers (4, 4′), corresponding to 6 hydrogens of two *exo* atropisomers (5, 5′) as was confirmed from the ^1^H-NMR of the separated *exo* atropisomers. The ratio of *endo* and *exo* isomers was determined from the ^1^H-NMR integrations (Table 2).

**Table 2:** Ratio between atropisomers of both *endo* and *exo* attacks of nitrone 2 to rotation-restricted dipolarophiles 3k–n

Entry	4 : 4′^a^		5 : 5′^a^	
	Total^b^	Isolated	Total^b^	Isolated
K	2.5 : 1	2 : 1	1.4 : 1	—
L	1.4 : 1	—	1.7 : 1	1.4 : 1
M	2.7 : 1	7.3 : 1	1.5 : 1	—
N	1.4 : 1	1.3 : 1	1.4 : 1	—

a 4 and 4′ are major and minor *endo* atropisomers, respectively, and 5 and 5′ are major and minor *exo* atropisomers, respectively.

b The isomer ratio was determined from ^1^H-NMR integrations (400 MHz).

Moreover, the effect of single bond restricted rotation on the route of the cycloaddition reaction was confirmed by using dipolarophile 3o, where the *ortho* substituent (*o*-fluorine atom) does not cause steric hindrance between the *N*-maleimide moiety and the carbonyl groups of the pyrrolidine ring. *Endo* cycloadduct 4o gave only three signals in the region 3.8–6.2 ppm: two doublets and a singlet. At the same time, the ^1^H-NMR spectrum of the crude reaction product showed, in the same region, only six signals corresponding to two cycloadducts.

Also, GC/mass chromatography (ESI†) confirmed the above mentioned results. The chromatogram obtained for the crude reaction products of the cycloaddition using dipolarophile 3f is simple (fewer peaks) than the chromatogram obtained for the crude reaction products of the cycloaddition using dipolarophile 3m (more peaks), and this confirms the formation of more products in the case of dipolarophile 3m than dipolarophile 3f confirming the occurrence of atropisomerism in the formed cycloadducts obtained by cycloaddition using dipolarophiles with *ortho* substituent (such as 3m).

**Fig. 5:**
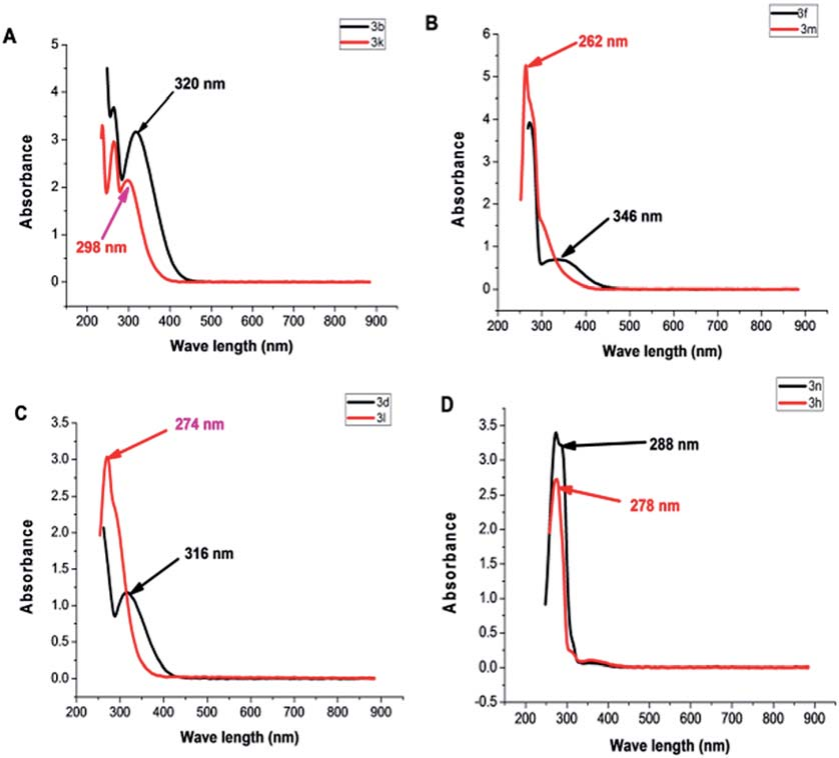
Absorption spectra of dipolarophiles with *para* or *ortho* substituents: (A) 3b and 3k, (B) 3f and 3m, (C) 3d and 3l, (D) 3h and 3n.

Ultimately, non-coplanarity in dipolarophiles 3k–n was confirmed by comparing their UV-visible spectra (Fig. 5). Dipolarophiles with *ortho* substituents have lower *λ*_max_ than the corresponding dipolarophiles having the substituent at *meta* or *para* position (Table 3).

**Table 3:** *λ*_max_ of different dipolarophiles (in CHCl_3_)

Dipolarophile	*λ*_max_ (nm)	Dipolarophile	*λ*_max_ (nm)
a	316	i	298
b	320	k	298
c	285	l	274
d	316	m	262
e	314	n	288
f	346	o	300
h	278		

**Fig. 6:**
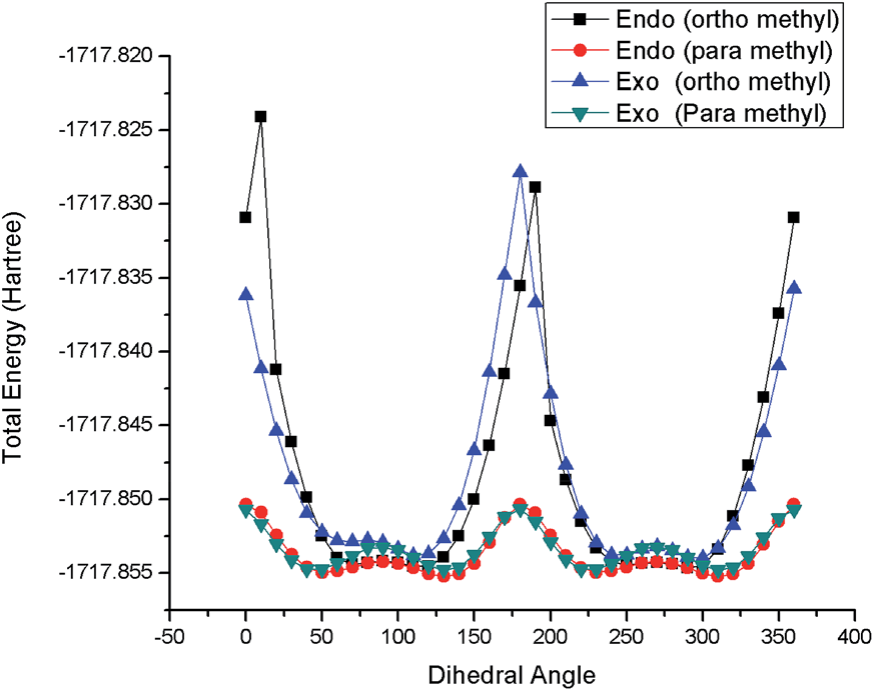
DFT calculation of the energy barrier for the C–N rotation in *endo* and *exo* isomers with either *para* or *ortho* methyl substitution.

The formation of atropisomers, as a result of restricted rotation around single bonds, was confirmed by density functional theory (DFT) calculations, which were performed using the Gaussian 09 package. The geometrical optimization and (C6–N5–C1′–C6′) dihedral angle scans of *endo* and *exo* cycloadducts, with methyl substitution at either *ortho* or *para* position, were performed using DFT at the B3LYP level.^21^ The solvent effect has been considered based on the polarizable continuum model.^22^ The solvent used in this calculation was toluene. The 6-311G(d,p) basis sets were employed for all atoms. Fig. 6 reveals the energy barriers for rotation around C–N bond of cycloadducts 4b, 5b, 4k and 5k. The presence of *ortho* substitution (4k, 5k) increases the energy barrier of rotation regardless of whether the cycloadduct is *endo* or *exo*. This confirms that the formation of separable atropisomeric cycloadducts of *endo* and *exo* cycloaddition duplicated the formed cycloadducts to be four rather two as in the cases of cycloaddition using dipolarophiles with free single bond rotation.

## Conclusions

We have reported the significant influence of restricted rotation in dipolarophiles on the route of cycloaddition leading to the formation of atropisomeric cycloadducts. Furthermore, this restricted rotation could induce facial selectivity of the addition where the major cycloadduct(s) was the one formed by cycloaddition at the face with less steric hindrance.

## Conflicts of interest

There are no conflicts to declare.

## Supplementary Material

RA-010-C9RA10039C-s001

RA-010-C9RA10039C-s002
