# Supplementary material for: Diastereoselective synthesis of atropisomeric pyrazolyl pyrrolo[3,4-d]isoxazolidines via pyrazolyl nitrone cycloaddition to facially divergent maleimides: intensive NMR and DFT studies
Source: RSC Adv. 2020 Jan 6;10(2):845–50. doi: 10.1039/c9ra10039c (PMC9047523; doi:10.1039/c9ra10039c)
Supplement: RA-010-C9RA10039C-s001 [file RA-010-C9RA10039C-s001.pdf]

Electronic Supplementary Material (ESI) for RSC Advances.
This journal is © The Royal Society of Chemistry 2020

1,3-dipolar Cycloaddition Reactions of Pyrazole Based Nitrone with N-substituted Maleimides: Atropisomers Formation and Evidences by NMR Studies and DFT Calculations

Awad Said and Talaat El-Emary

Chemistry Department, Faculty of Science, Assiut University, Assiut, Egypt 71516

This work is dedicated to the soul of prof. Galal M. El-Naggar (1942-2016)

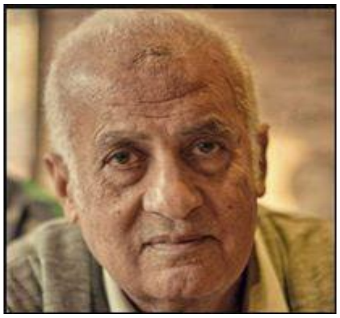

Table of Contents

Materials and reagents 2

Nitrone (2) 2

Cycloaddition reaction with N-phenyl maleimide (3a) 5

Cycloaddition reaction with N-(4-methylphenyl) maleimide (3b) 8

Cycloaddition reaction with N-(3-methylphenyl) maleimide (3c) 13

Cycloaddition reaction with N-(2-methylphenyl) maleimide (3k) 17

Cycloaddition reaction with N-(4-chlorophenyl) maleimide (3d) 22

Cycloaddition reaction with N-(3-chlorophenyl) maleimide (3e) 27

Cycloaddition reaction with N-(2-chlorophenyl) maleimide (3l) 30

Cycloaddition reaction with N-(4-methoxyphenyl) maleimide (3f) 34

Cycloaddition reaction with N-(2-methoxyphenyl) maleimide (3m) 41

Cycloaddition reaction with N-(4-nitrophenyl) maleimide (3g) 48

Cycloaddition reaction with N-(1-naphthyl) maleimide (3n) 50

Cycloaddition reaction with N-(2-naphthyl) maleimide (3h) 53

Cycloaddition reaction with N-(benzyl) maleimide (3i) 59

Cycloaddition reaction with N-(4-acetylphenyl) maleimide (3j) 63

Cycloaddition reaction with N-(2-flourophenyl) maleimide (3o) 66

Computational Calculations 71

References 79

Materials and reagents

^{1}H-NMR spectra (400MHz) were recorded by JOEL-400 spectrometer in deuteriated Chloroform ($CDCl_3$) as a solvent and the chemical shifts are quoted in δ and were related to that of tetramethylsilane (TMS) (δ in ppm). Proton decoupled or DEPET ^{13}C-NMR spectra (100.5MHz) were recorded by JOEL-100 spectrometer in deuteriated Chloroform ($CDCl_3$) as a solvent and the chemical shifts are quoted in δ and were related to that of tetramethylsilane (TMS) (δ in ppm). Chemical shifts (δ) and *J* values (*J*) were reported in ppm and Hz, respectively. Mass spectra and GC/Mass were recorded on a JEOL JMS600instrument. Elemental analyses were recorded on Gmbh VarioEL V2.3. Melting points were determined on a Mel-Temp II melting point apparatus and are uncorrected. All three dimension structures are drawn using Chem 3D ultra 9.0 (chem. office 2008) program. IR spectra were determined with a Shimadzu 470Infrared spectrophotometer using KBr wafer technique (ύ cm^{-1}). Reactions were monitored by thin layer chromatography (TLC) using plastic sheets coated with silica gel, POLYGRAM®SIL G/UV254 (Merck). TLC plates were inspected under UV light.

Nitrone (2)

(*Z*)-N-((1,3-diphenyl-1H-pyrazol-4-yl)methylene)benzenamine oxide $C_{22}H_{17}N_3O$ (2)

A solution of (20 g, 0.08 mol) 1,3-diphenyl 4-formyl pyrazole in ethanol (20 ml) was added to a solution of (9.0 g ,0.08 mol) N-phenylhydroxylamine in ethanol (50ml) and the mixture was heated under reflux for 2 hours. Pale yellow crystals were separated, yield 25 g (92 %,), mp: 157-159°C, from ethanol. FTIR (KBr) (cm^{-1}): 3150(Ar. C-H), 3050(H-C=N+), 1597(C=N), 1579(C=N^+). ^{1}H-NMR spectrum: δ ppm(400 MHz, $CDCl_3$) 7.1-8(m, 16H, Ar H), 9.7(s, 1H, CH=N^+). ^{13}C{H}NMR spectrum: δ(100.5 MHz, $CDCl_3$) 132.32, 129.69, 129.52, 129.19, 128.99, 128.89, 128.79, 127.11, 127.03, 121.32, 119.45, 113.22 Mass Spectrum (electron impact): e/m(%) 339.2(0.5), 286.8(100), 219.1(58). Anal. Calcd for ($C_{22}H_{17}N_3O$) (%): C, 77.86; H, 5.05; N, 12.38. Found; C, 77.78; H, 5.04; N, 12.38.

^{1}H-NMR Spectrum

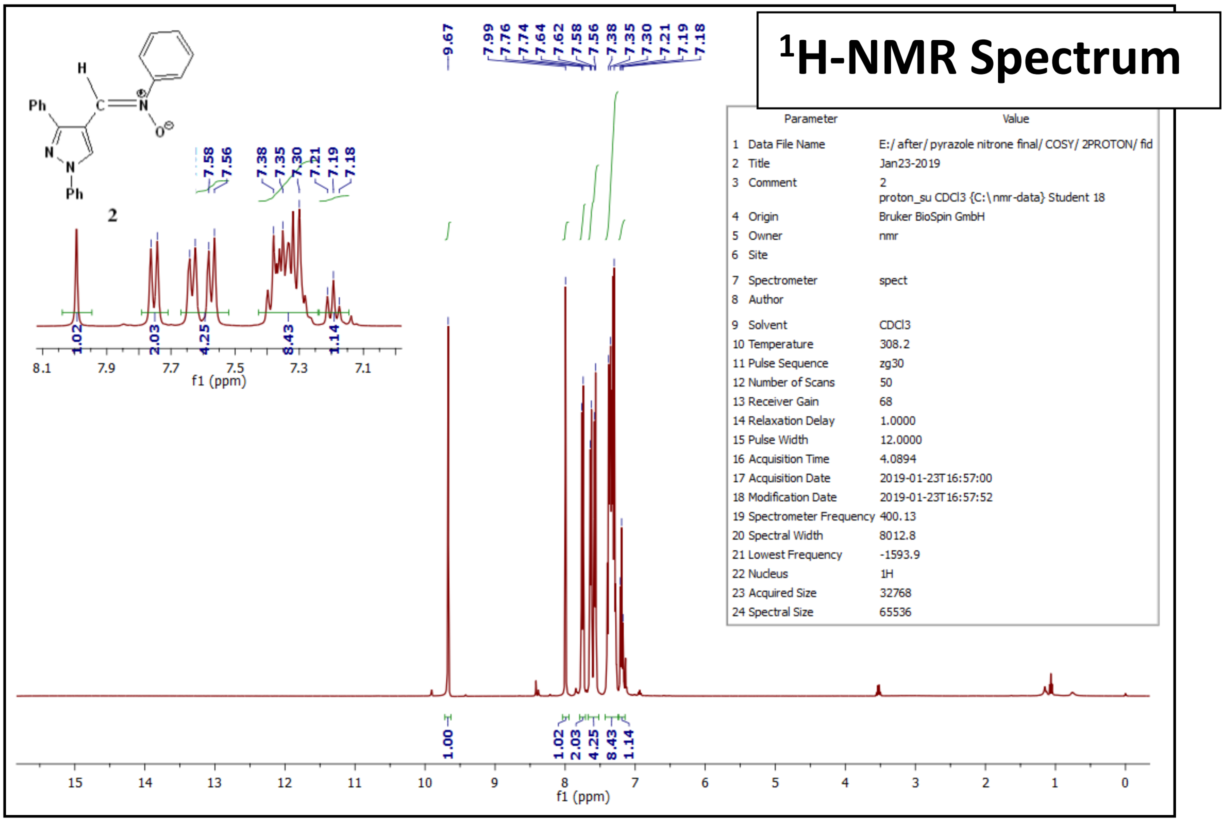

^{13}H-NMR Spectrum

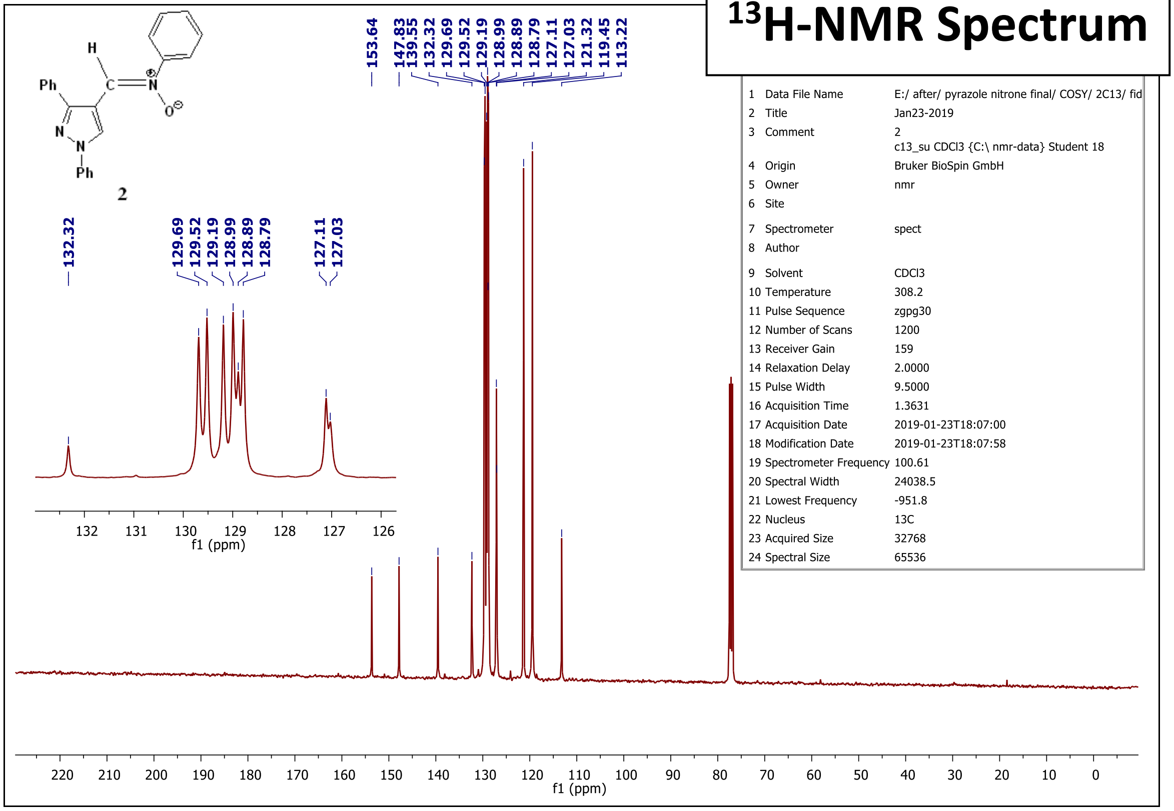

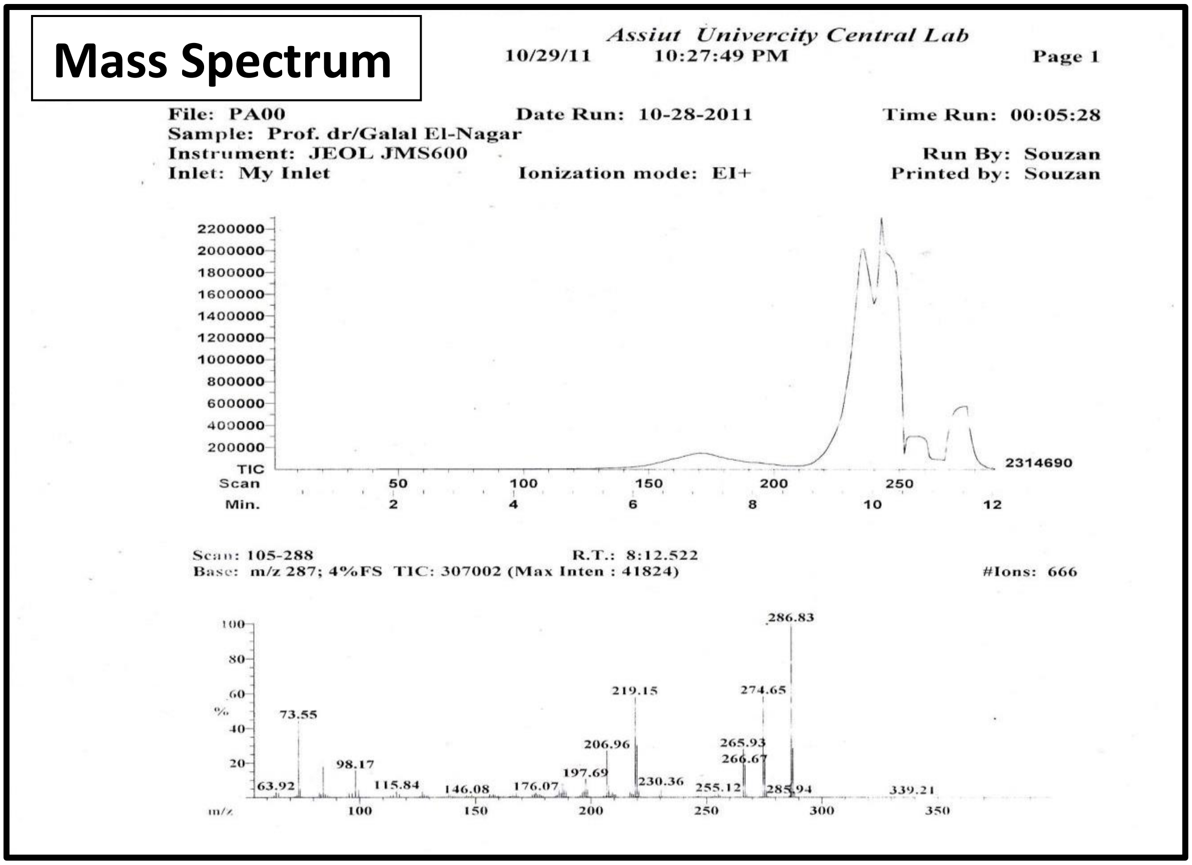

Cycloaddition addition reactions of N-substitued maleimide (3a-3o) with the Nitrone (2).

General procedures:

A mixture of the appropriate N-substituted maleimide (**3a-3o**), was prepared as recorded in literature[1], (3mmol) and (*Z*)-N-((1,3-diphenyl-1H-pyrazol-4-yl) methylene)benzenamine oxide (**2**) (1.1g, 3 mmol) in toluene (15 ml) was heated at 100°C under reflux for 24 hours, the reaction was monitored by TLC (adsorbent: silica gel and eluent: petroleum ether(40:60)& ethyl acetate (2:1)) to determine the number of the formed isomers. By filtration, the *endo* isomer was filtered off as a major product and recrystallized from toluene and petroleum ether 40-60^{0}C. The filtrate was concentrated to minimum volume and the *exo* isomer was separated using preparative TLC (Silica gel F254, eluent pet.ether 40-60/AcOEt(2:1)).

Cycloaddition with N-phenyl maleimide (3a)

Formation of 3-(1,3-diphenyl-1H-pyrazol-4-yl)-2,5-diphenyldihydro-2H-pyrrolo[3,4-d]isoxazole-4,6(5H,6aH)-dione $C_{32}H_{24}N_4O_3$.

Reaction mixture **(4a,5a)**: ^{1}H-NMR spectrum: δ ppm (400 MHz, $CDCl_3$) 4.1(d, *J* 7.2 Hz, 1H, H3a(endo)), 4.4(t, *J* 9.2 Hz, 1H,H3a(exo)), 4.91(d, *J* 8.8 Hz, 1H, H3(exo)), 5.5(m, 2H, H6a(exo)and H6a(endo)), 6.02(s, 1H, H3(endo)), 6.53-9.34(m, 42H, Ar H), 9.98 (s, 1H, Nitrone CH=N).

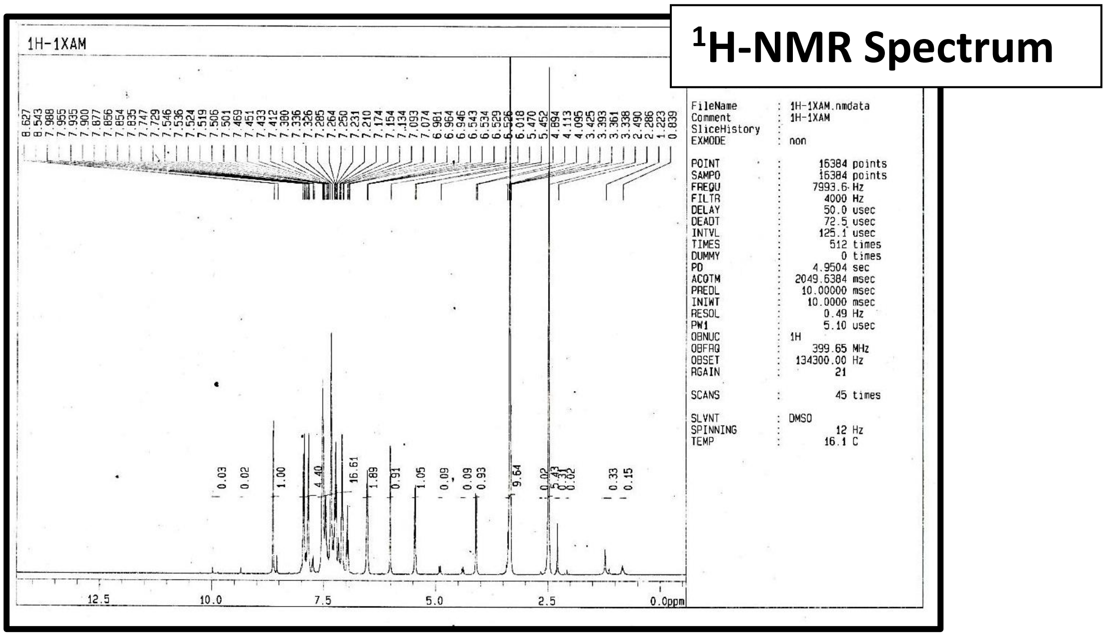

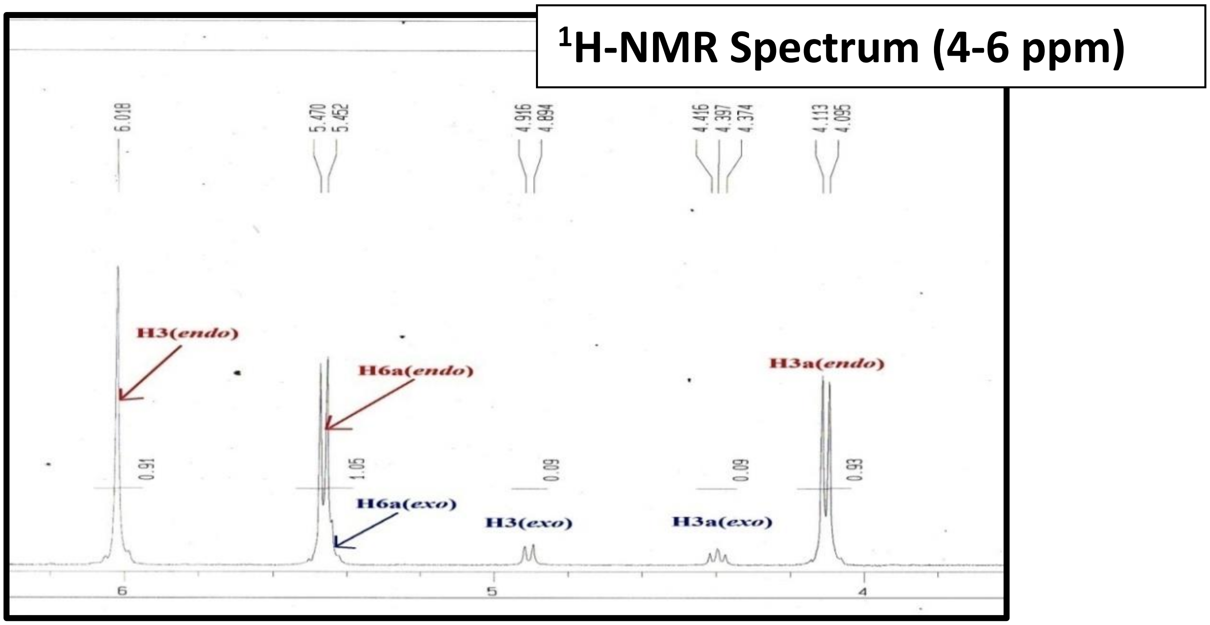

Endo-isomer **(4a):** (1.4 g, 80%); white crystals; mp: 144-146°C; FTIR (KBr) (cm^{-1}):3059(Ar. C-H), 2971(Aliph. C-H), 1717(C=O). ^{1}H-NMR spectrum: δ ppm (400 MHz,$CDCl_3$) 3.99 (d, *J* 7.32 Hz, 1H, H3a), 5.11 (d, *J* 7.32 Hz, 1H, H6a), 6.05 (s,1H, H3), 6.6-8.15 (m, 21H, Ar H). ^{13}C{H}-NMRspectrum: δ ppm (100.5 MHz, CDCl) 24.8, 55.9, 63.3 (3 Aliphatic CH); 114.7(2), 119.1 (2), 119.4, 123.2, 125.5, 126.2(2),126.8, 127.4, 128.3 (2), 128.6, 129.1(4), 129.6(4), 130.9, 132.3,139.8, 148.3, 150.4 (27 Aromatic **C**); 172.7 and 173.8 (2 **C**=O). Anal. Calcd for ($C_{32}H_{24}N_4O_3$) (%): C, 74.99; H, 4.72; N, 10.93. Found; C, 74.96; H, 4.55; N, 10.85.

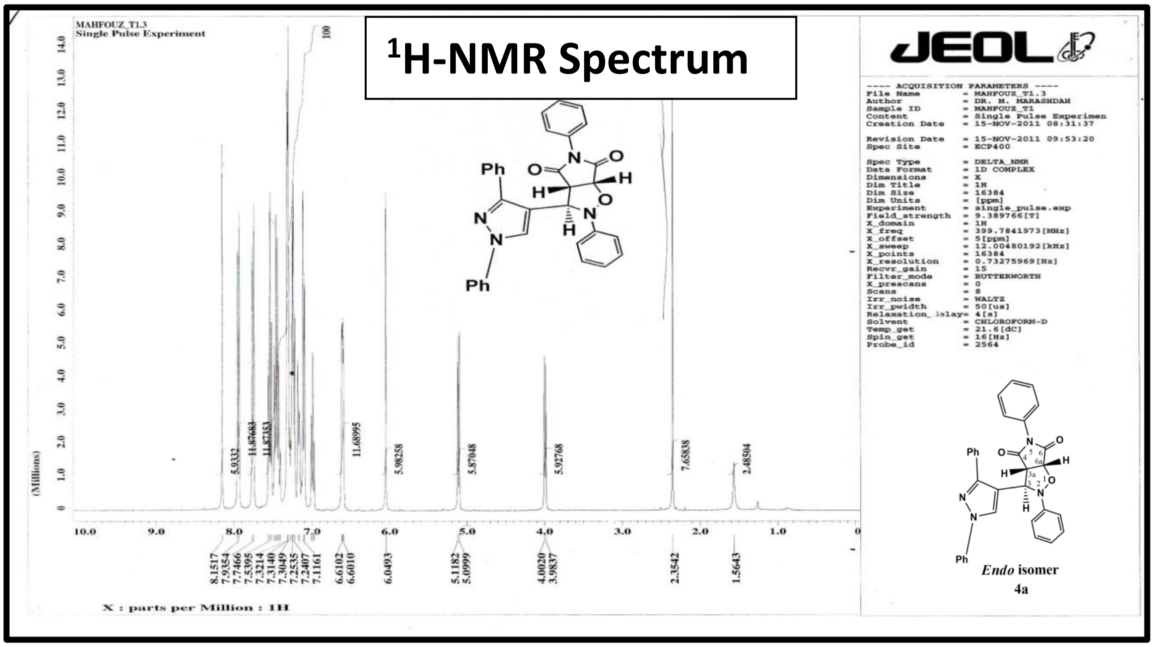

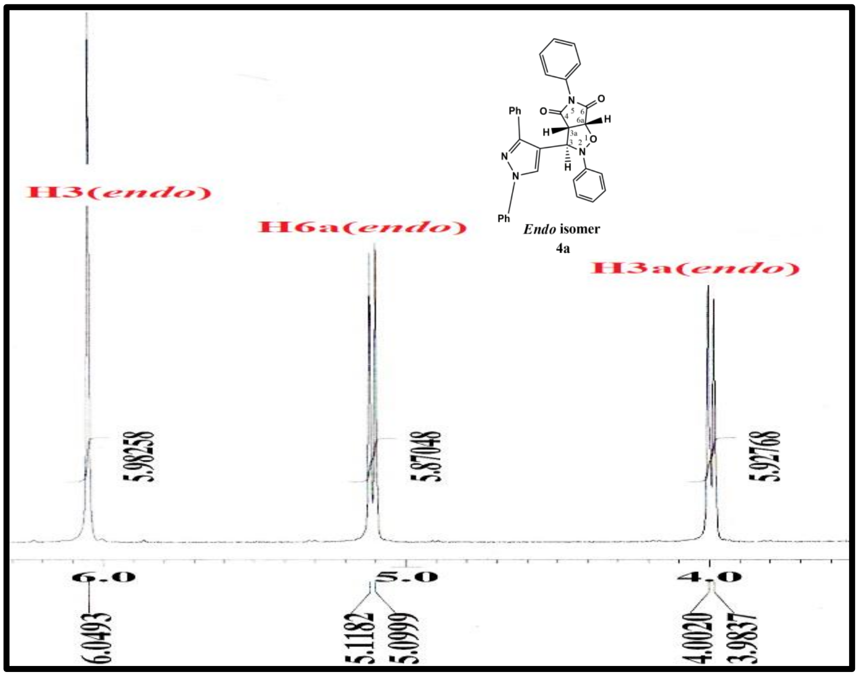

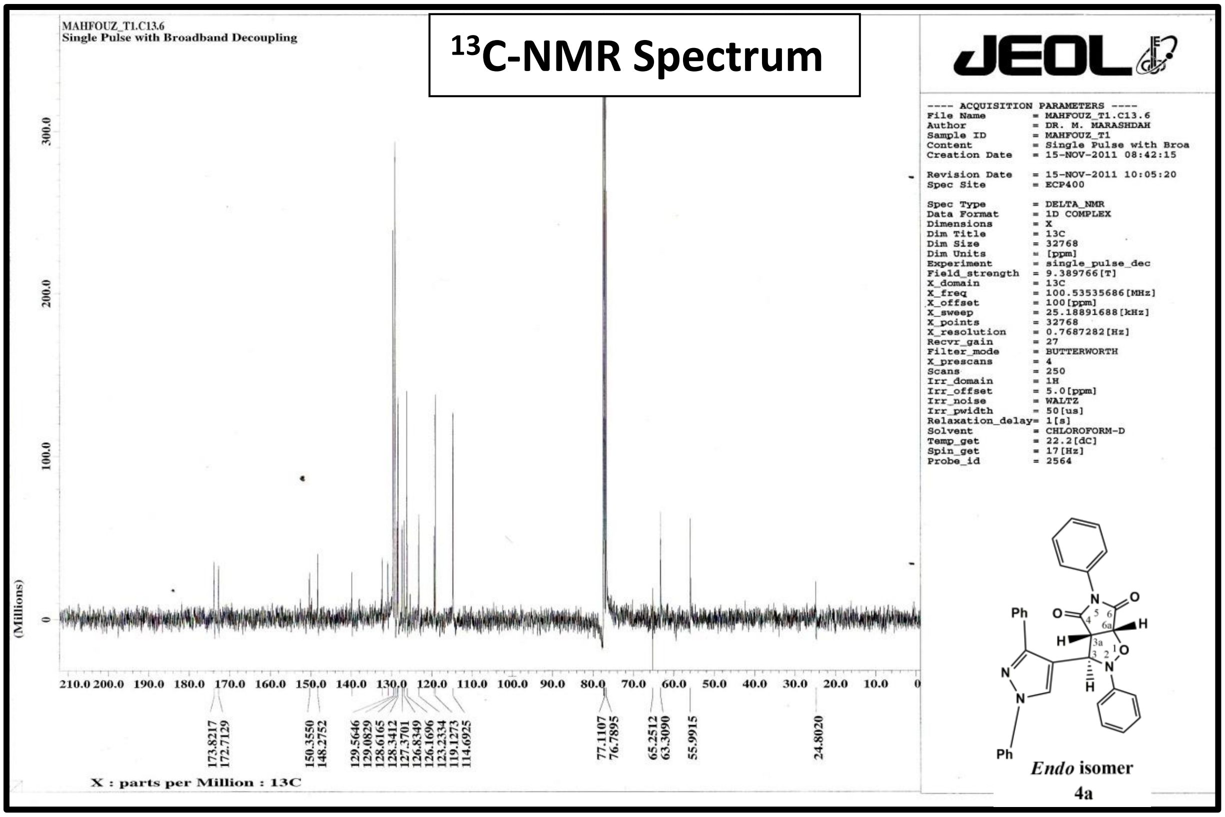

13C-NMR Spectrum
MAHFOUZ_T1.C13.6
Single Pulse with Broadband Decoupling
JEOL
---- ACQUISITION PARAMETERS ----
File Name = MAHFOUZ_T1.C13.6
Author = DR. M. MARASHDAH
Sample ID = MAHFOUZ_T1
Content = Single Pulse with Broa
Creation Date = 15-NOV-2011 08:42:15
Revision Date = 15-NOV-2011 10:05:20
Spec Site = ECP400
Spec Type = DELTA_NMR
Data Format = 1D COMPLEX
Dimensions = X
Dim Title = 13C
Dim Size = 32768
Dim Units = [ppm]
Experiment = single_pulse_dec
Field_strength = 9.389766[T]
X_domain = 13C
X_freq = 100.53535686[MHz]
X_offset = 100[ppm]
X_sweep = 25.18891688[kHz]
X_points = 32768
X_resolution = 0.7687282[Hz]
Recvr_gain = 27
Filter_mode = BUTTERWORTH
X_prescans = 4
Scans = 250
Irr_domain = 1H
Irr_offset = 5.0[ppm]
Irr_noise = WALTZ
Irr_pwidth = 50[us]
Relaxation_delay= 1[s]
Solvent = CHLOROFORM-D
Temp_get = 22.2[dC]
Spin_get = 17[Hz]
Probe_id = 2564
(Millions)
300.0
200.0
100.0
0
210.0 200.0 190.0 180.0 170.0 160.0 150.0 140.0 130.0 120.0 110.0 100.0 90.0 80.0 70.0 60.0 50.0 40.0 30.0 20.0 10.0 0
173.8217
172.7129
150.3550
148.2752
129.5646
129.0829
128.6165
128.3412
127.3701
126.8349
126.1696
123.2334
119.1273
114.6925
77.1107
76.7895
65.2512
63.3090
55.9915
24.8020
X : parts per Million : 13C
Ph
O
N
O
5
4
6
6a
H
H
3a
1
O
3
2
N
N
N
Ph
Ph
H
Endo isomer
4a

Cycloaddition with N-(4-methylphenyl) maleimide (3b)
Formation of 3-(1,3-diphenyl-1H-pyrazol-4-yl)-2-phenyl-5-p-tolyldihydro-2H-pyrrolo[3,4-d]isoxazole-4,6(5H,6aH)-dione $C_{33}H_{26}N_4O_3$.

Reaction mixture **(4b,5b)**: ^{1}H-NMR spectrum: δ ppm (400 MHz, $CDCl_3$) 2.31 (s, 3H, CH_3(*endo*)), 2.3(s, 3H, CH_3(*exo*)), 3.98(d, *J*7.6 Hz, 1H, H3a(*endo*)), 4.1(t, *J* 8.4 Hz, 1H, H3a(*exo*)), 5.1(d, *J*7.2 Hz, 1H, H6a(*endo*)), 5.13(d, *J* 8.8 Hz, 1H, H3(*exo*)), 5.3(d, *J*8 Hz, 1H, H6a(*exo*)), 6(s, 1H, H3(*endo*)), 6.45-8.55(m, 40H, Ar H), 10.05 (s, 1H, Nitrone CH=N).

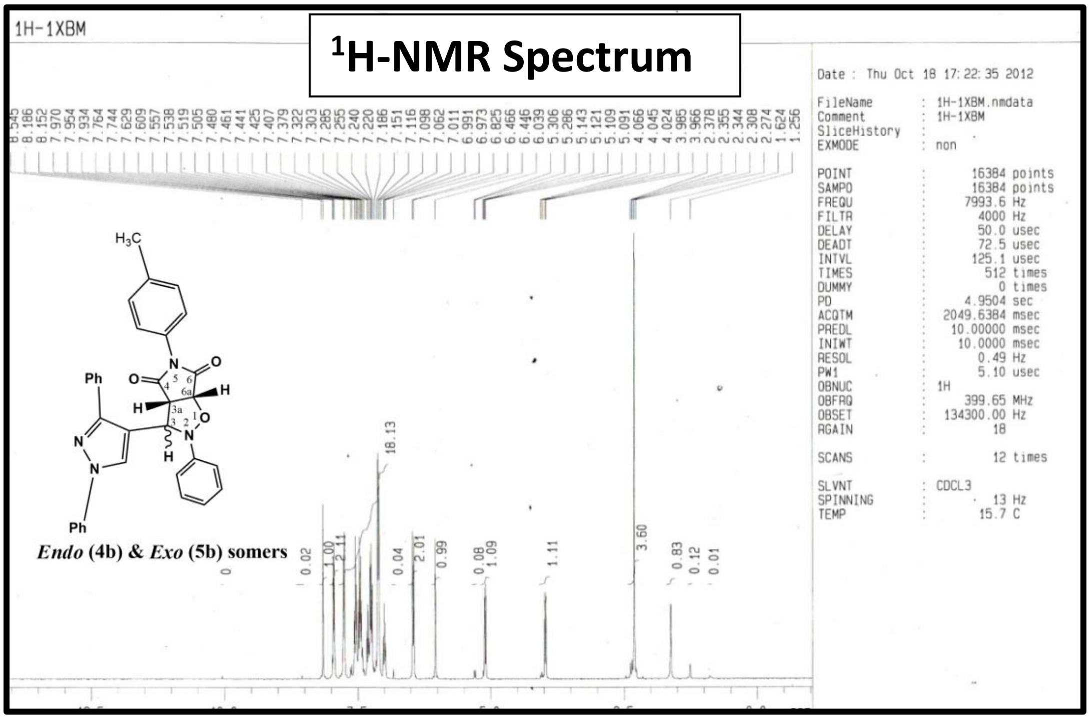

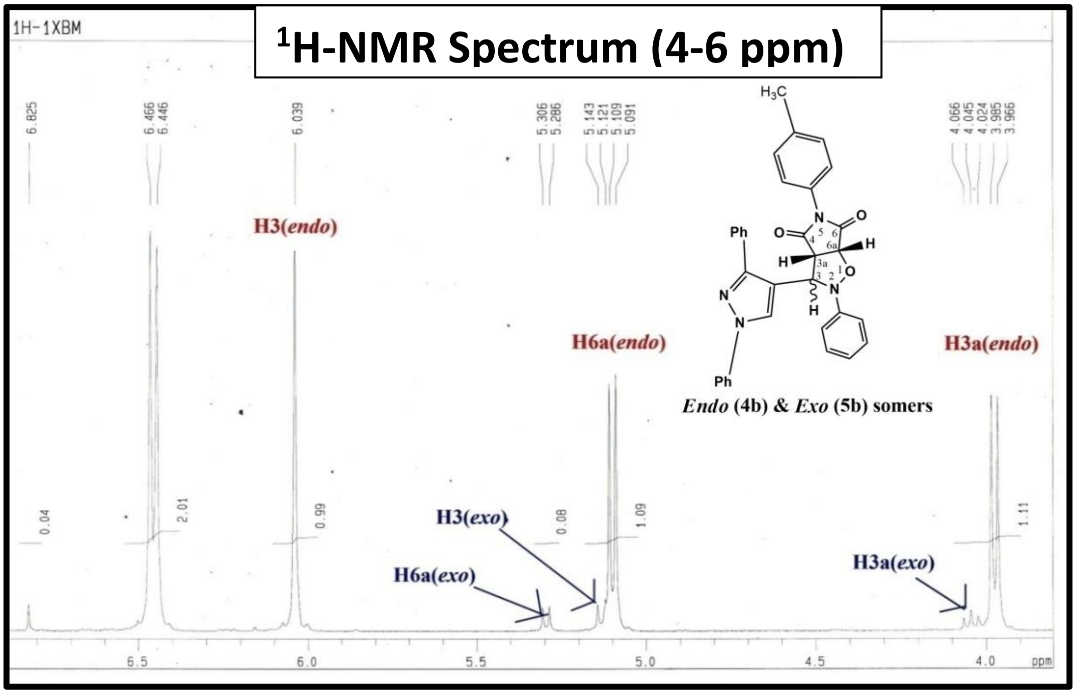

Endo-isomer **(4b)**: (1.1 g, 60%); white crystals; mp: 200-202°C. IR: (KBr) (cm^{-1}): 3020(Ar. C-H), 2910(Aliph. C-H), 1723(C=O).^{1}H-NMR spectrum: δ ppm (400 MHz, $CDCl_3$) 2.31(s, 3H, CH3), 3.98 (d, *J* 7.32 Hz, 1H, H3a), 5.1(d, *J* 7.32 Hz, 1H, H6a), 6.03(s,1H, H3), 6.5-8.2(m, 20H, Ar-H). $^{13}C\{H\}$NMR spectrum: δ ppm (100.5 MHz, $CDCl_3$) 114.7(2), 119.1(2), 119.4,123.2, 125.9(2), 126.8, 127.4, 128.4(3), 128.6, 129.1(2),129.5(5), 129.7, 132.3, 139.2, 139.8, 148.3, 150.1 (27 Aromatic <u>**C**</u>); 172.8, 173.9 (2 **C**=O). Anal. Calcd for ($C_{33}H_{26}N_4O_3$) (%): C, 75.27; H, 4.98; N, 10.64. Found; C, 74.95; H, 4.83; N, 10.52.

^{1}H-NMR Spectrum

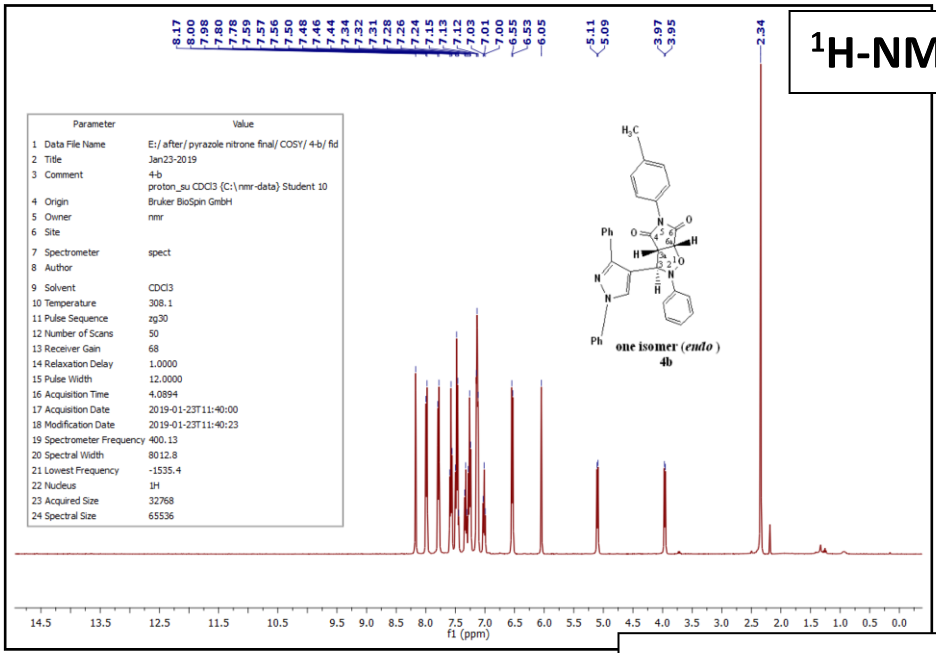

^{1}H-NMR Spectrum (4-6 ppm)

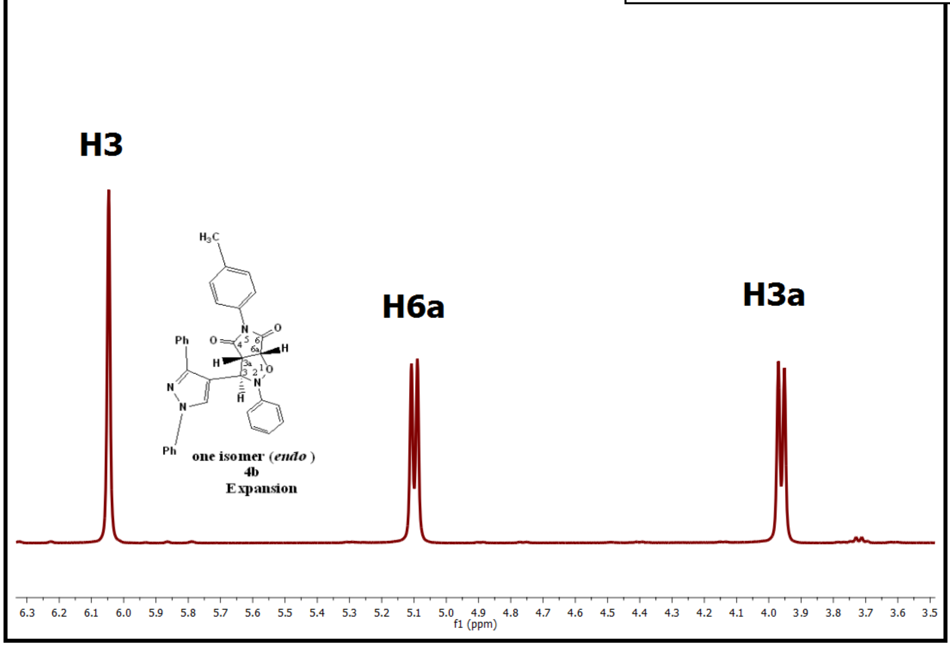

NOE 2D NMR

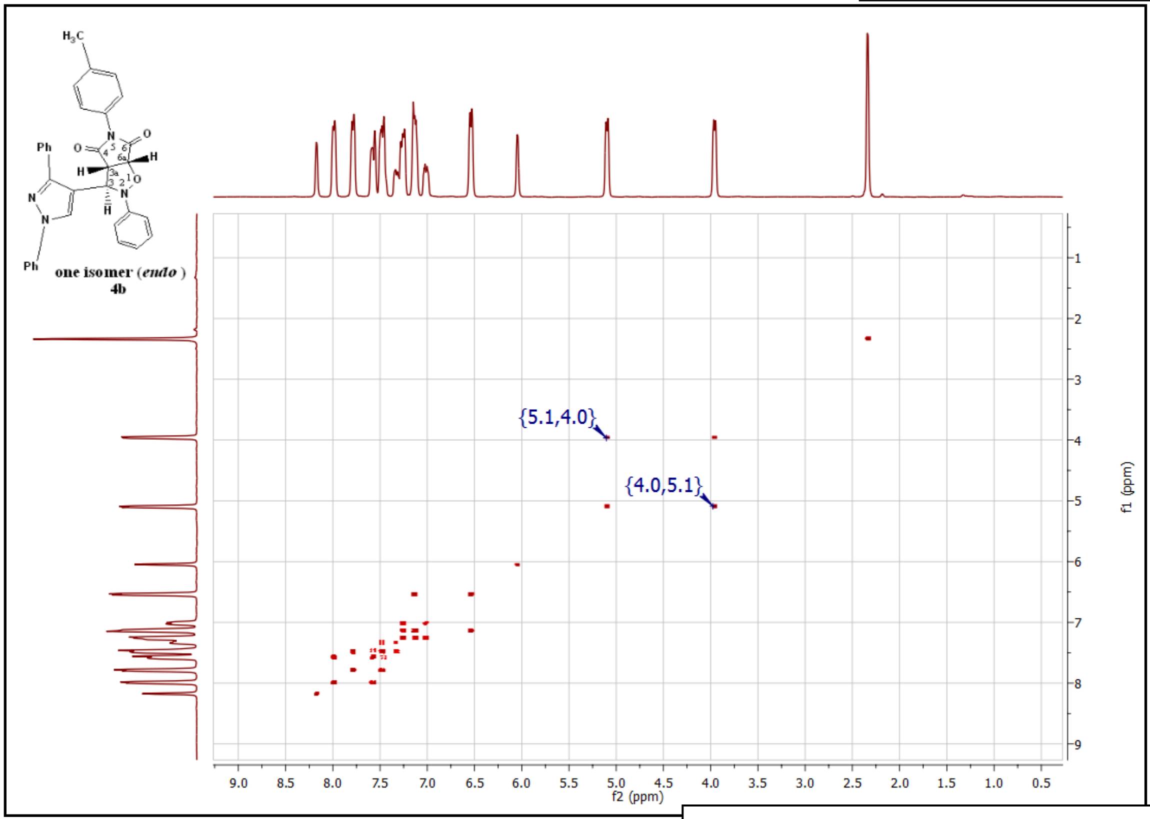

NOE 2D NMR (4-6 ppm)

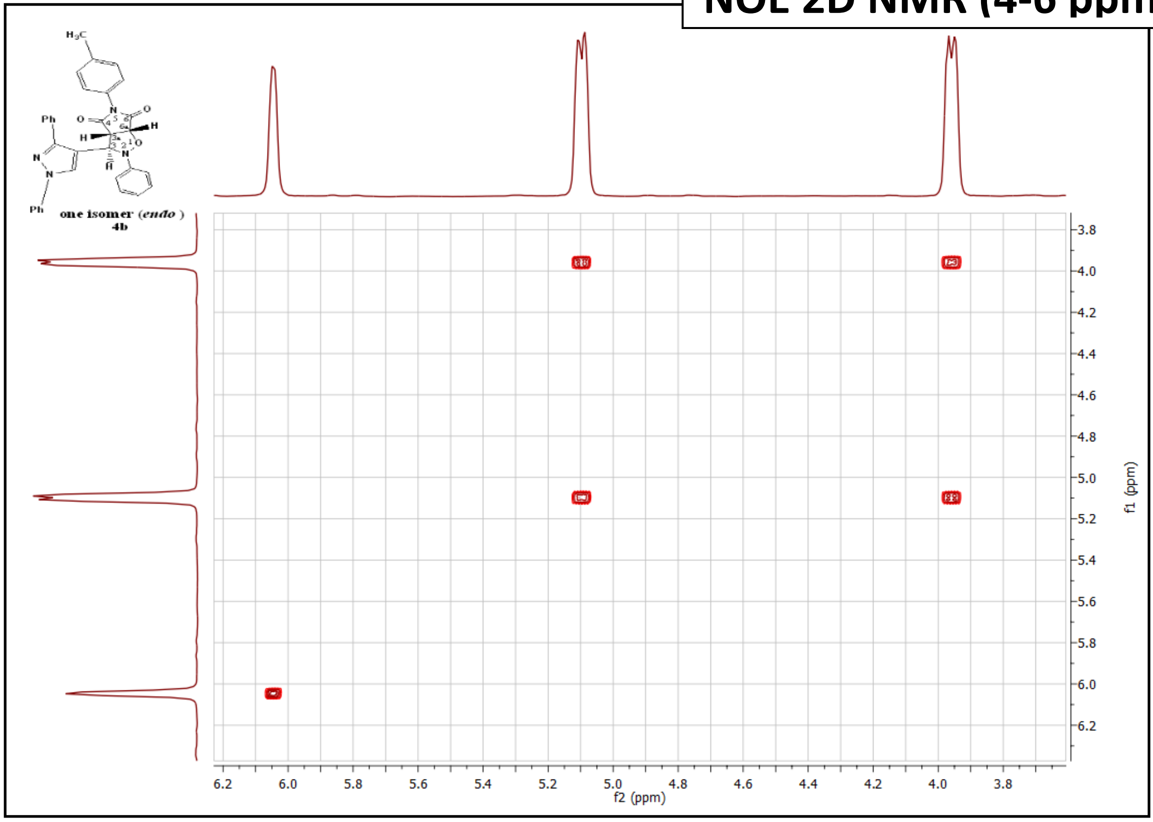

^{13}C-NMR Spectrum

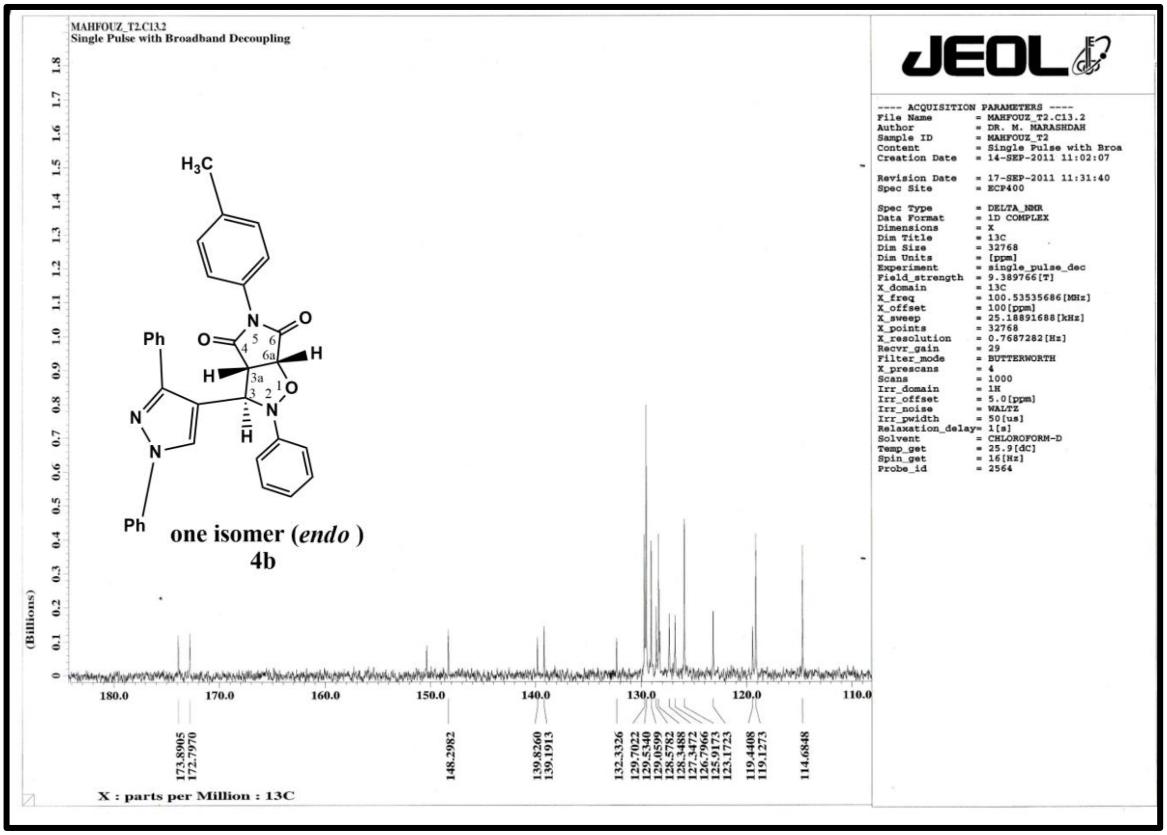

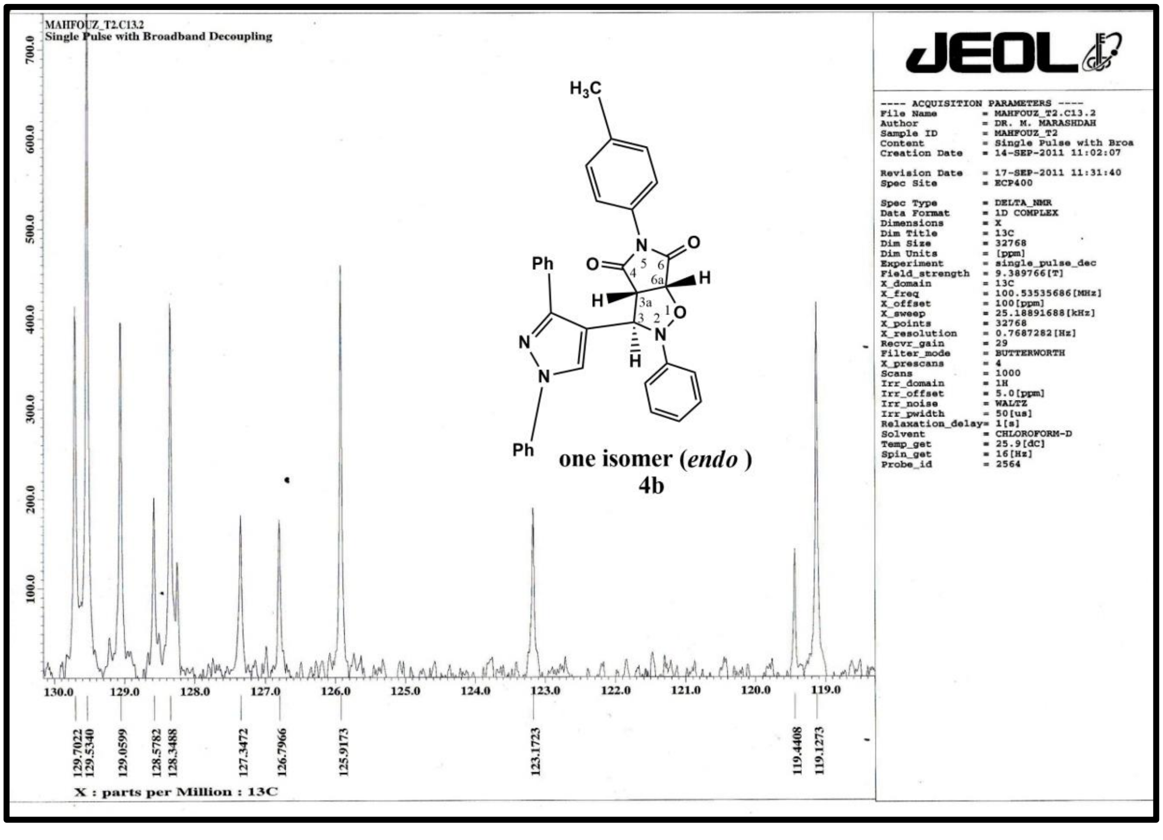

Exo-isomer **(5b)**: (0.02 g, 0.95%); white crystals; m.p.; 160-162°C. Mass spectrum (electron impact): m/e (%) 526.2(13), 339(17), 91.2(12.5), 76.9(100).

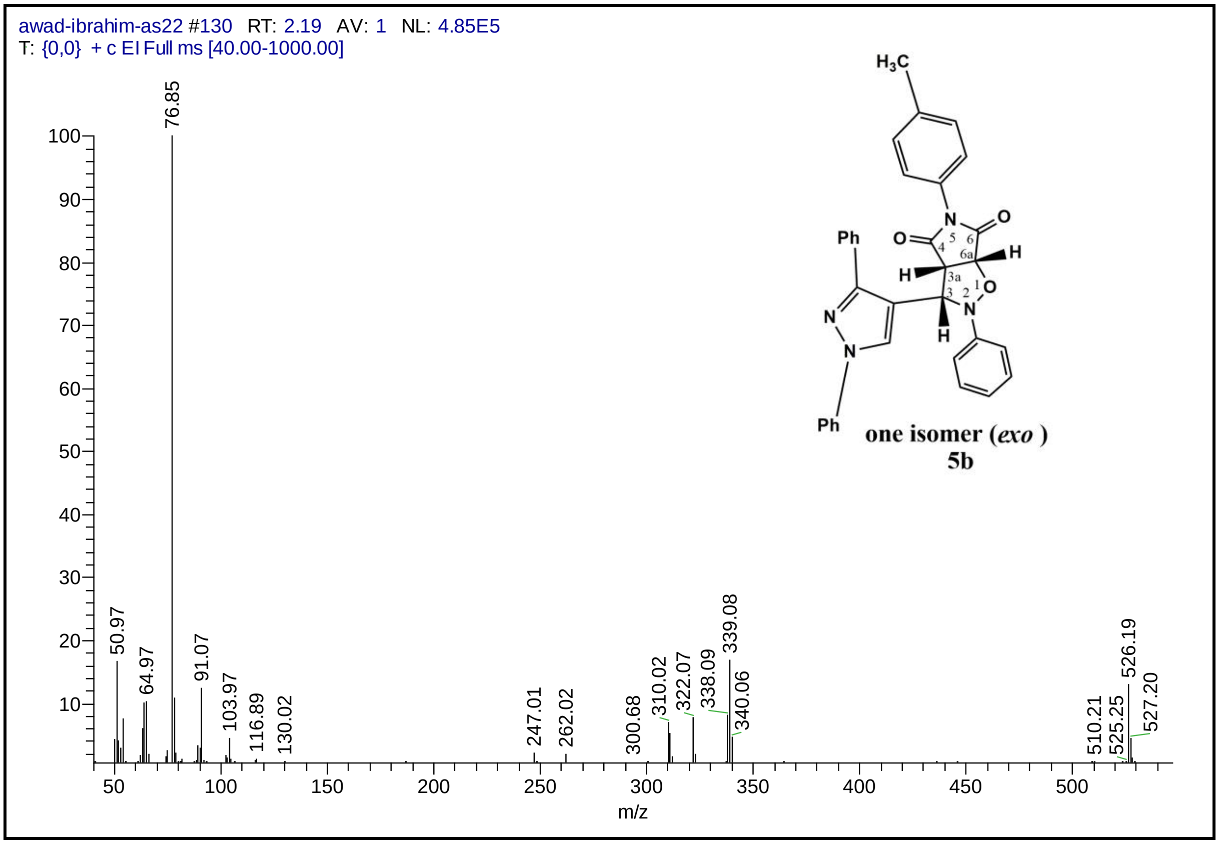

Cycloaddition with N-(3-methylphenyl) maleimide (3c)

Formation of 3-(1,3-diphenyl-1H-pyrazol-4-yl)-2-phenyl-5-m-tolyldihydro-2H-pyrrolo[3,4-d]isoxazole4,6(5H,6aH)-dione $C_{33}H_{26}N_4O_3$.

Reaction mixture**(4c,5c)**:^{1}H-NMR spectrum: δ ppm(400 MHz, $CDCl_3$) 2.08(s, 3H, CH_3(*exo*)), 2.12(s, 3H, CH_3(*exo*)), 3.97(d, *J* 8 Hz, 1H, H3a(*endo*)), 4.05(t, *J* 8.8 Hz, 1H, H3a(exo)), 5.09(d, *J* 7.6 Hz, 1H, H6a(*endo*)), 5.14(d, *J* 8.4 Hz, 1H, H3(*exo*)), 5.3(d, *J* 7.6 Hz, 1H, H6a(*exo*)), 6.04(s, 1H,H3(*endo*)), 6.2-8.5(m, 40H, Ar H), 10.03(s, 1H, Nitrone CH=N).

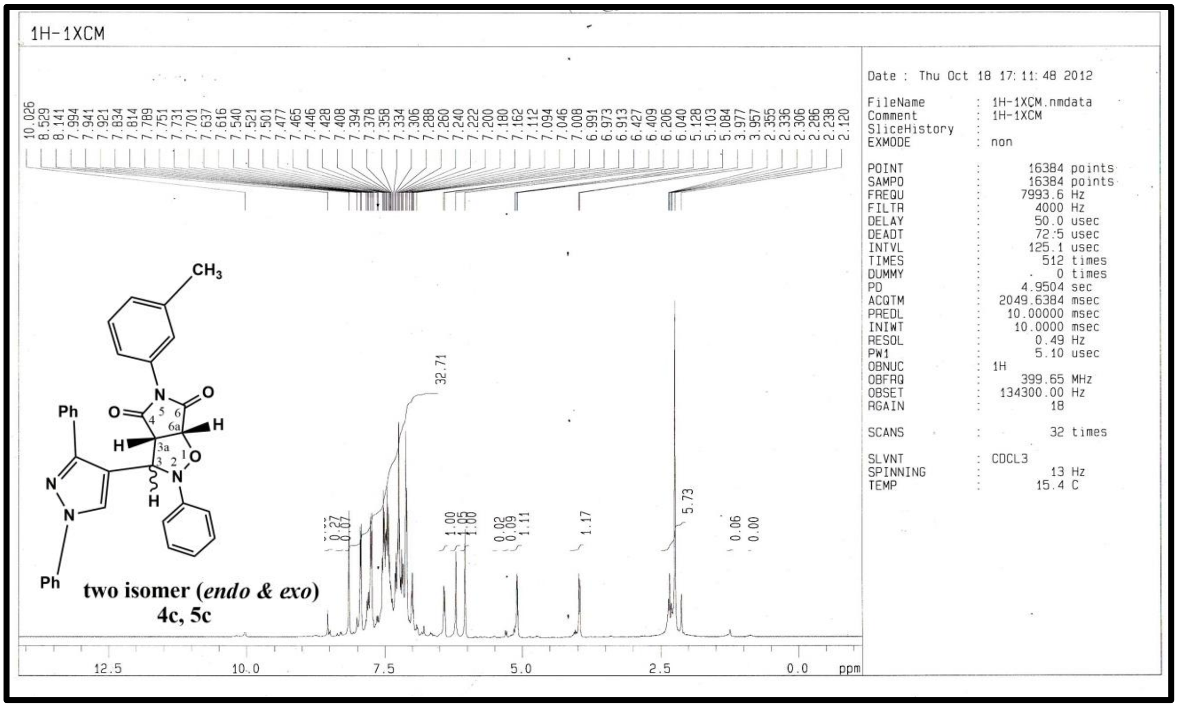

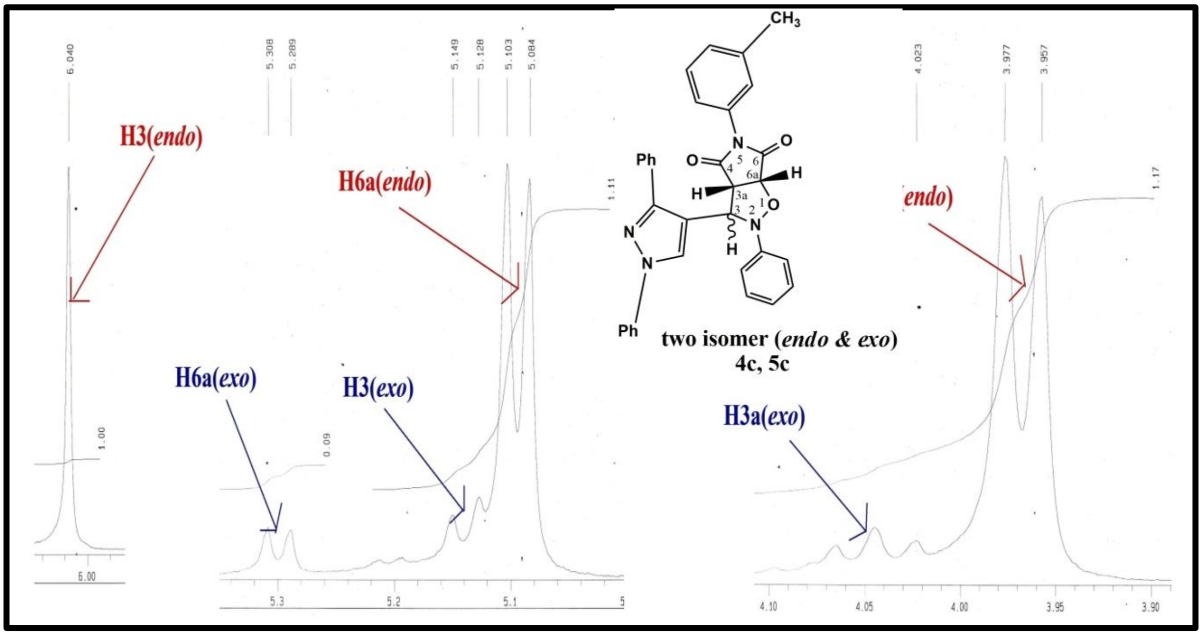

Endo isomer **(4c)**: (0.9 g, 50%); white crystals; mp: 200-202°C. 1H-NMR spectrum: δ ppm (400 MHz, CDCl3) 2.3 (s, 3H, CH_3), 4 (d, *J* 7.2 Hz, 1H, H3a), 5.1 (d, *J* 7.2 Hz, 1H, H6a), 6.1 (s, 1H, H3), 6.5-8.18 (m, 20H, Ar-H). ^{13}C{H}NMR spectrum: δ ppm (100.5 MHz, CDCl3) 21.3, 21.5, 55.9, 63.2(4 Aliphatic **C**); 114.6(2), 119.1(2), 119.4, 123.1, 123.2, 126.7, 126.8, 127.3, 128.3(2), 128.6, 128.8, 129(2), 129.5(2), 129.5 (2),129.9, 130.7, 132.3, 139.2, 139.7, 148.3, 150.3(27 Aromatic **C**); 172.8, 173.9(2 **C**=O) Anal. Calcd for ($C_{33}H_{26}N_4O_3$) (%): C, 75.27; H, 4.98; N, 10.64. Found; C, 74.94; H, 4.87; N, 10.52.

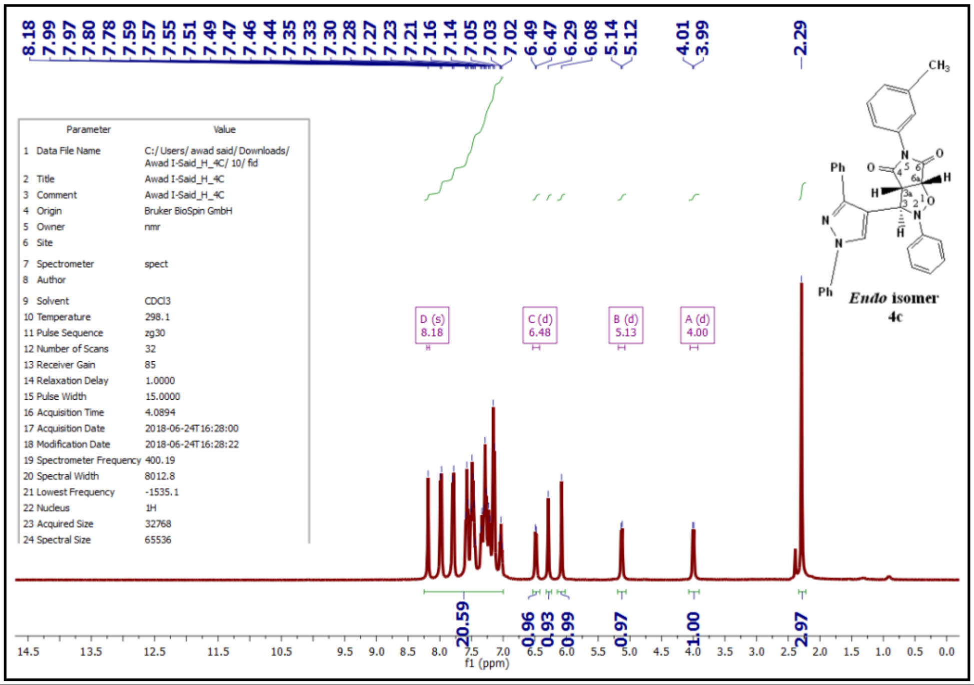

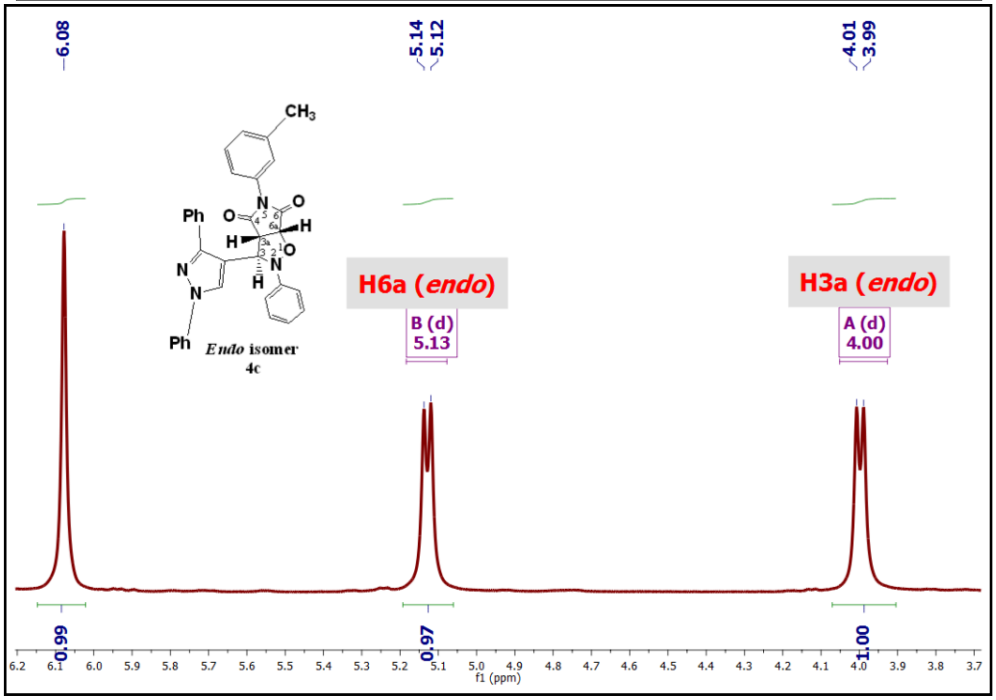

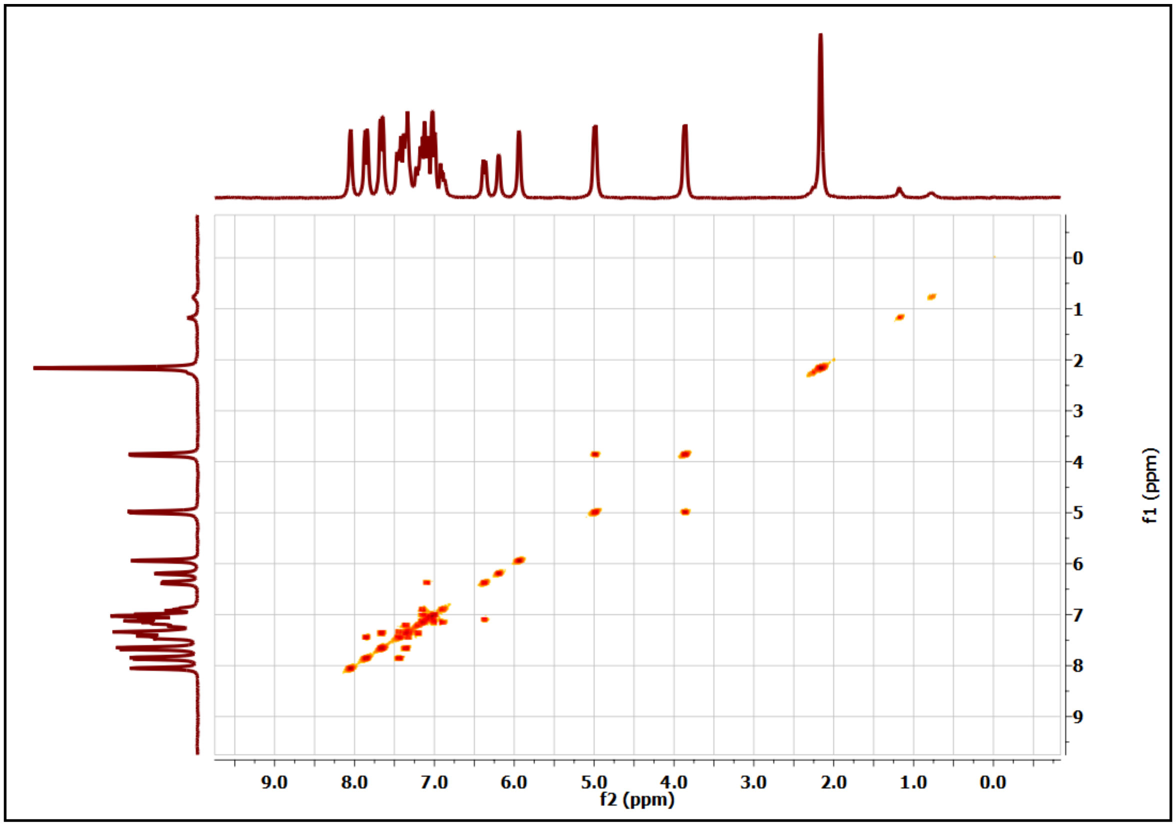
9.0
8.0
7.0
6.0
5.0
4.0
3.0
2.0
1.0
0.0
f2 (ppm)
0
1
2
3
4
5
6
7
8
9
f1 (ppm)

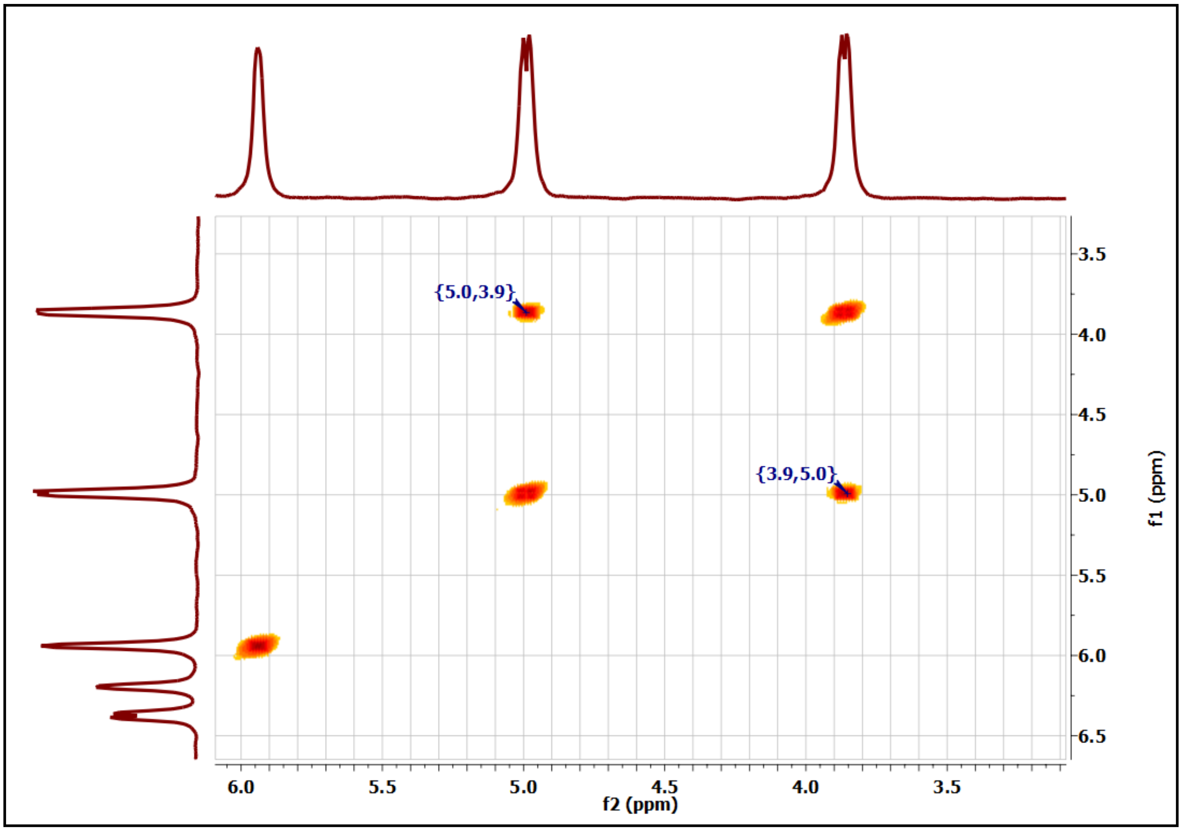
{5.0,3.9}
{3.9,5.0}
6.0
5.5
5.0
4.5
4.0
3.5
f2 (ppm)
3.5
4.0
4.5
5.0
5.5
6.0
6.5
f1 (ppm)

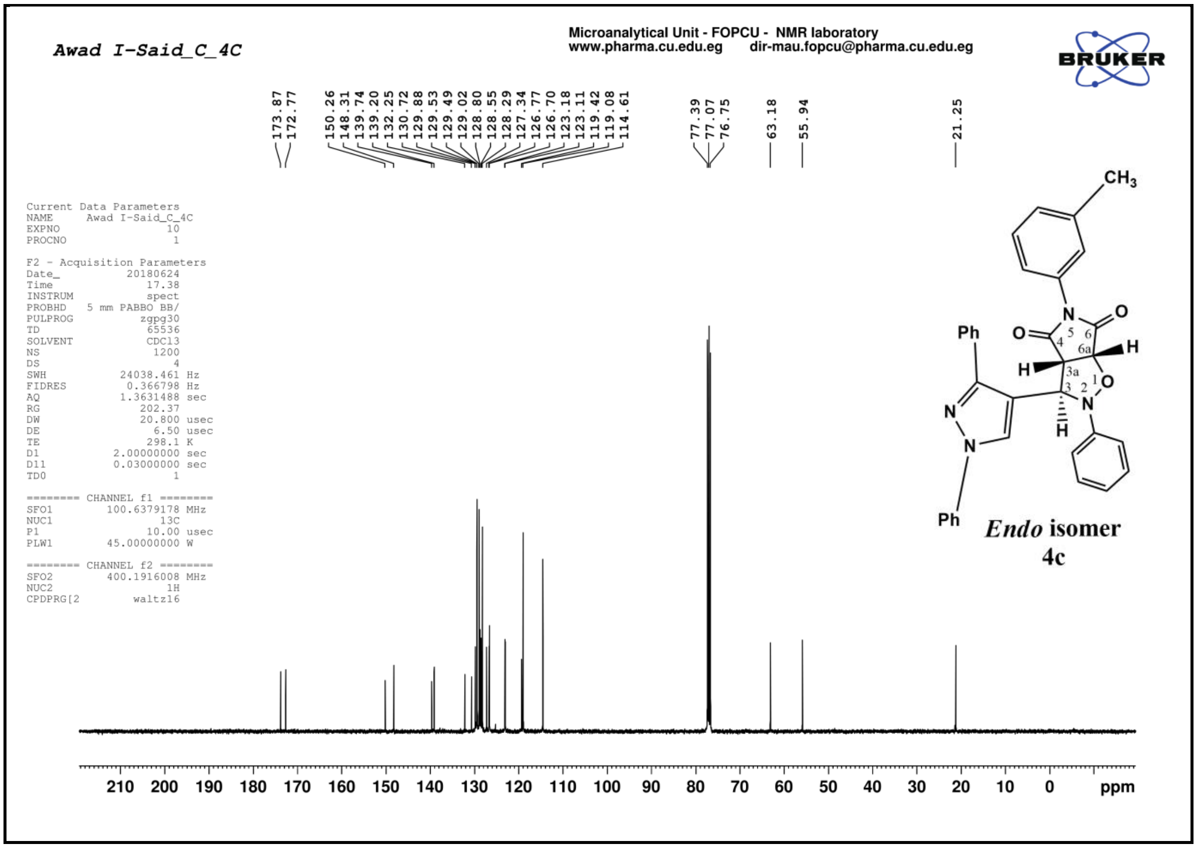
Awad I-Said_C_4C
Microanalytical Unit - FOPCU - NMR laboratory
www.pharma.cu.edu.eg dir-mau.fopcu@pharma.cu.edu.eg
BRUKER
Endo isomer
4c
ppm

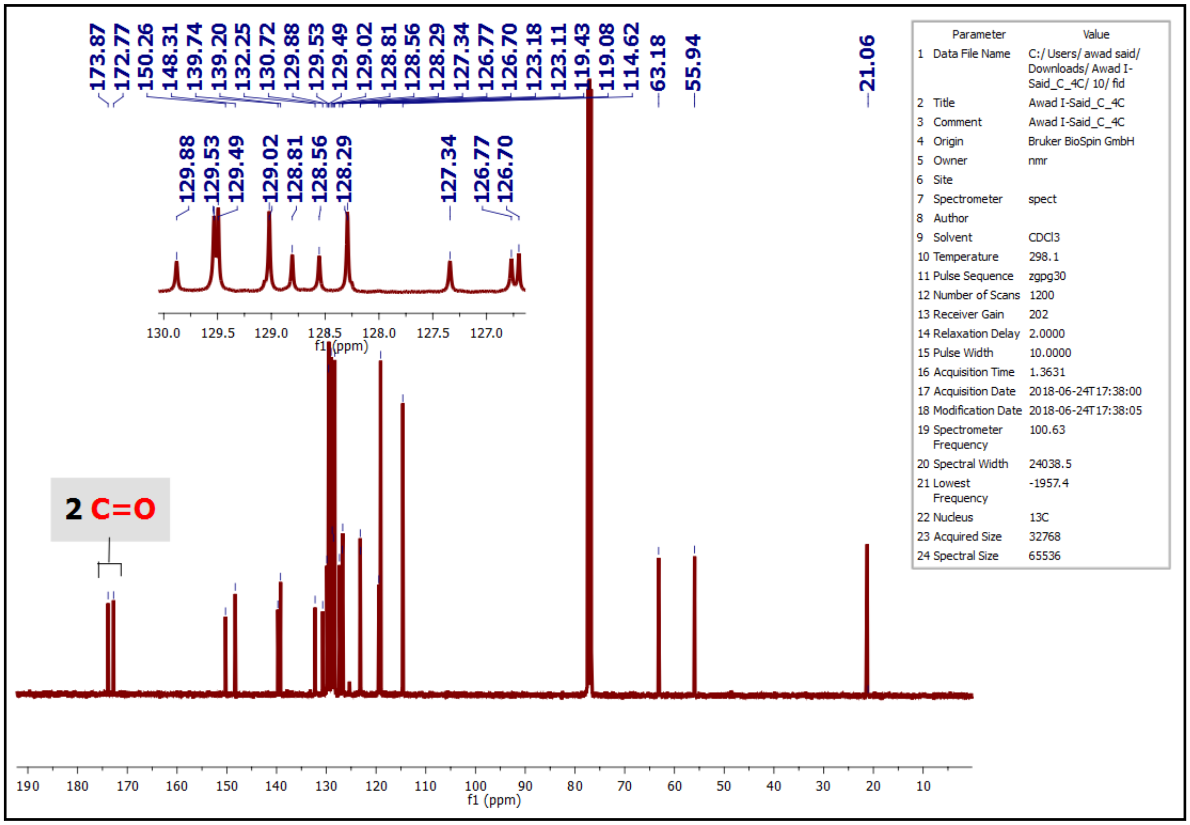
2 C=O
f1 (ppm)

Cycloaddition with N-(2-methylphenyl) maleimide (3k)

Formation of 3-(1,3-diphenyl-1H-pyrazol-4-yl)-2-phenyl-5-o-tolyldihydro-2H-pyrrolo[3,4-d]isoxazole-4,6(5H,6aH)-dione $C_{33}H_{26}N_4O_3$

Reaction mixture **(4k,4'k,5k,5'k)**: ^{1}H-NMR spectrum: δ ppm(400 MHz, $CDCl_3$) 1.8(s, 3H, CH_3(**4'k**)), 2(s, 3H, CH_3(**4k**)), 2.06(s, 3H, CH_3(**5'k**)), 2.09(s,3H,CH_3(**5k**)), 3.94-4.06(m,4H, H3a (**4k**,**4'k**,**5k**,**5'k**)), 4.95-5.35(m,6H, H3(**5k**,**5'k**); H6a (**4k**,**4'k**,**5k**,**5'k**)), 5.62(d, *J* 7.6 Hz, 1H, H6'(**4k**)), 5.67 (s, 1H,H3(**4'k**)), 6.03(s, 1H, H3(**4k**)), 6.6-8.13(m, 80H, Ar H), 9.95(s,1H, Nitrone C<u>H</u>=N).

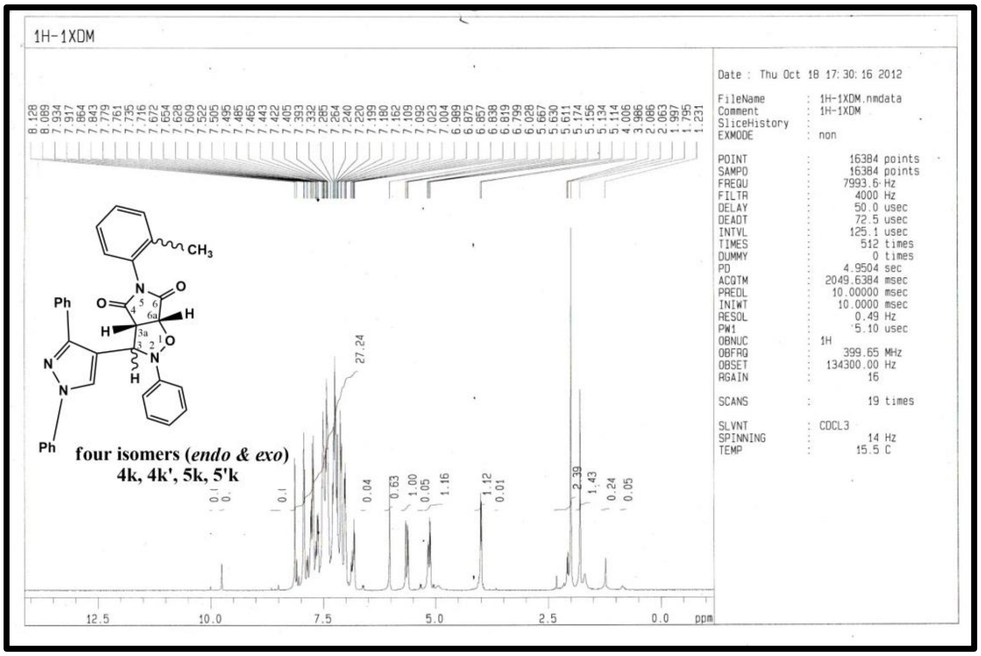

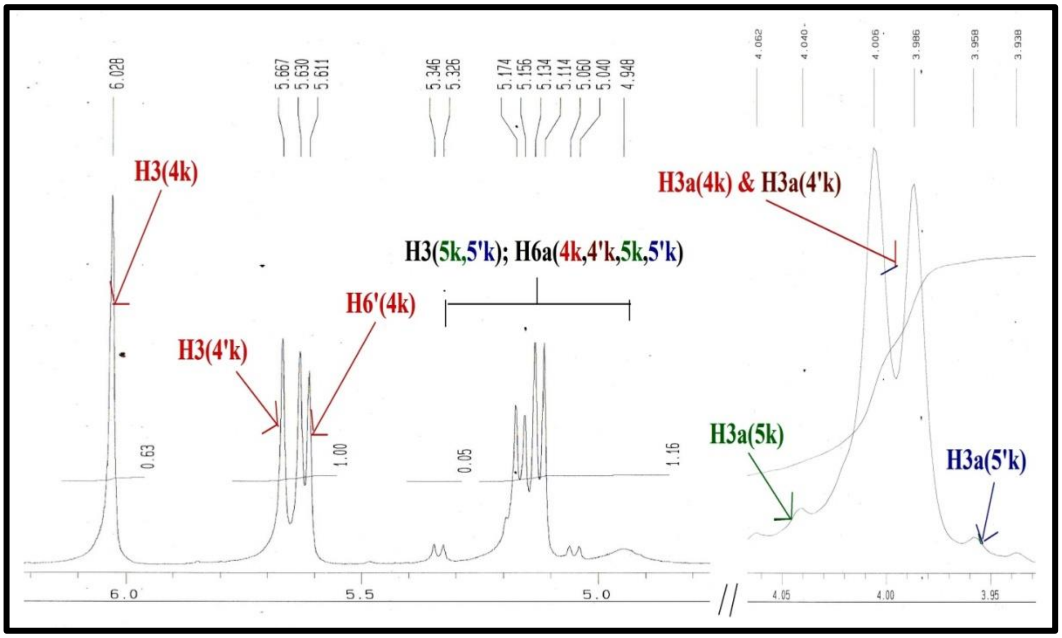

Endo-atropisomers **(4k, 4'k)**: (1 g, 58%); white crystals; mp: 188-190°C. FTIR (KBr) (cm^{-1}): 3003(Ar-H), 2970(Aliph-H), 1713(C=O).^{1}H-NMR spectrum: δ ppm(300 MHz, DMSO-d$_6$) 1.47(s, 3H, CH_3 (**4'k**)), 2.04 (s, 3H, CH_3 (**4k**)), 4.06 (d, *J* 7.32 Hz, 1H, H3a(**4k**), 4.09(d, *J* 7.32 Hz, 1H, H3a(**4'k**), 5.17(d, *J* 7.32 Hz, 1H, H6a(**4k**)), 5.22(d, *J* 7.32 Hz, 1H, H6a(**4'k**)), 5.68(d, *J* 8.08 Hz, 1H, H6'(**4k**)), 5.71(s, 1H, H3(**4'k**)), 6.06(s, 1H, H3(**4k**)), 6.16-8.84(m, 40H, ArH). ^{13}C{H}NMR spectrum:δ ppm (100.5 MHz, $CDCl_3$) 17.7 (**C**H_3); 56.3, 56.5, 63.2(3Aliphatic **C**); 114.7, 116.1, 119.1, 119.2, 119.5, 123.2, 126.7,126.8, 126.9, 127.1, 127.4(2), 127.6, 128.3(2), 128.6, 128.6,129.1(2), 129.5, 129.54(2), 129.7, 130.2, 130.5, 130.9, 148.3(27Aromatic **C**); 172.5, 173.8(2 **C**=O). Anal. Calcd for ($C_{33}H_{26}N_4O_3$)(%): C, 75.27; H, 4.98; N, 10.64. Found; C, 74.96; H, 5.11; N,10.51. The ratio of the isolated *endo*- atropisomers **4k:4'k** is 67:33.

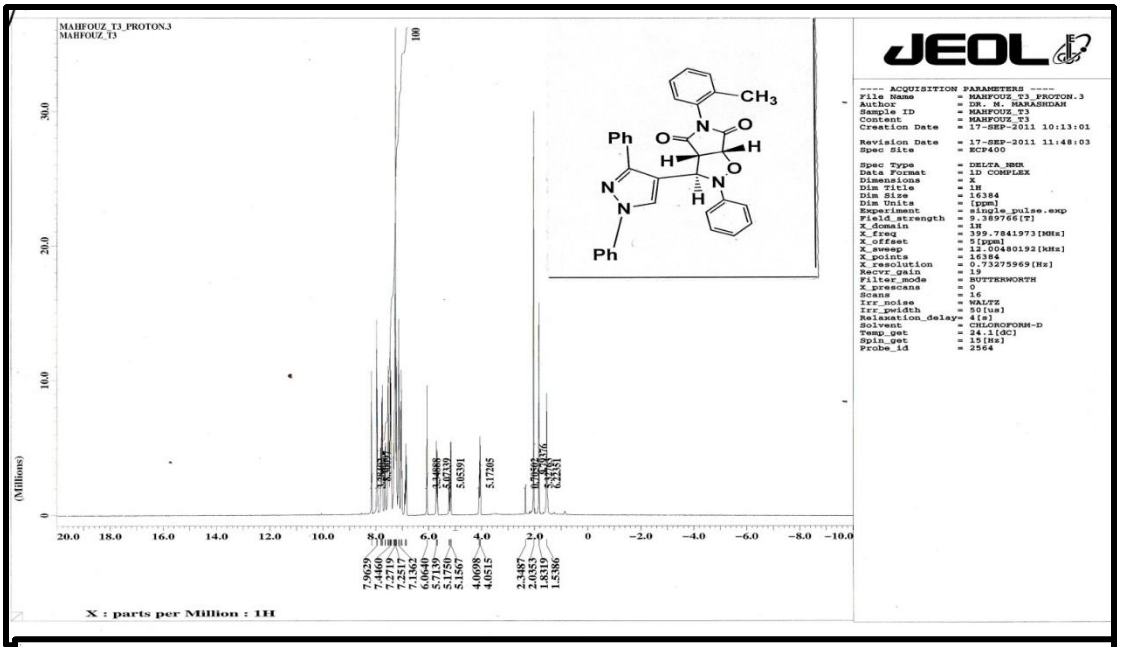

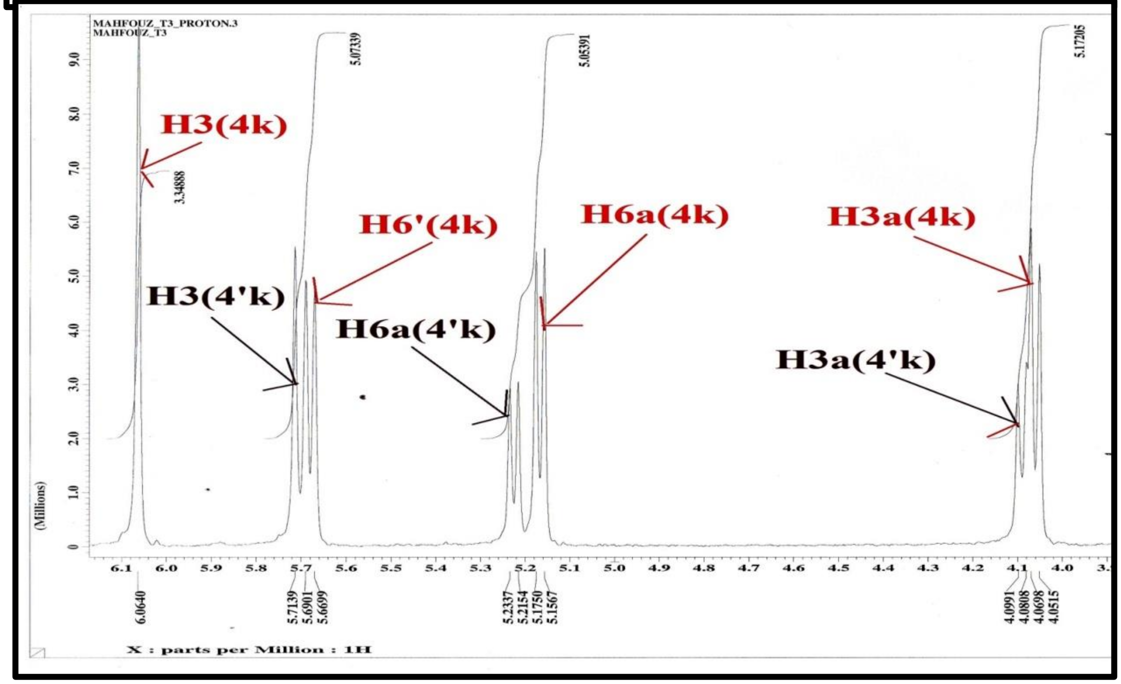

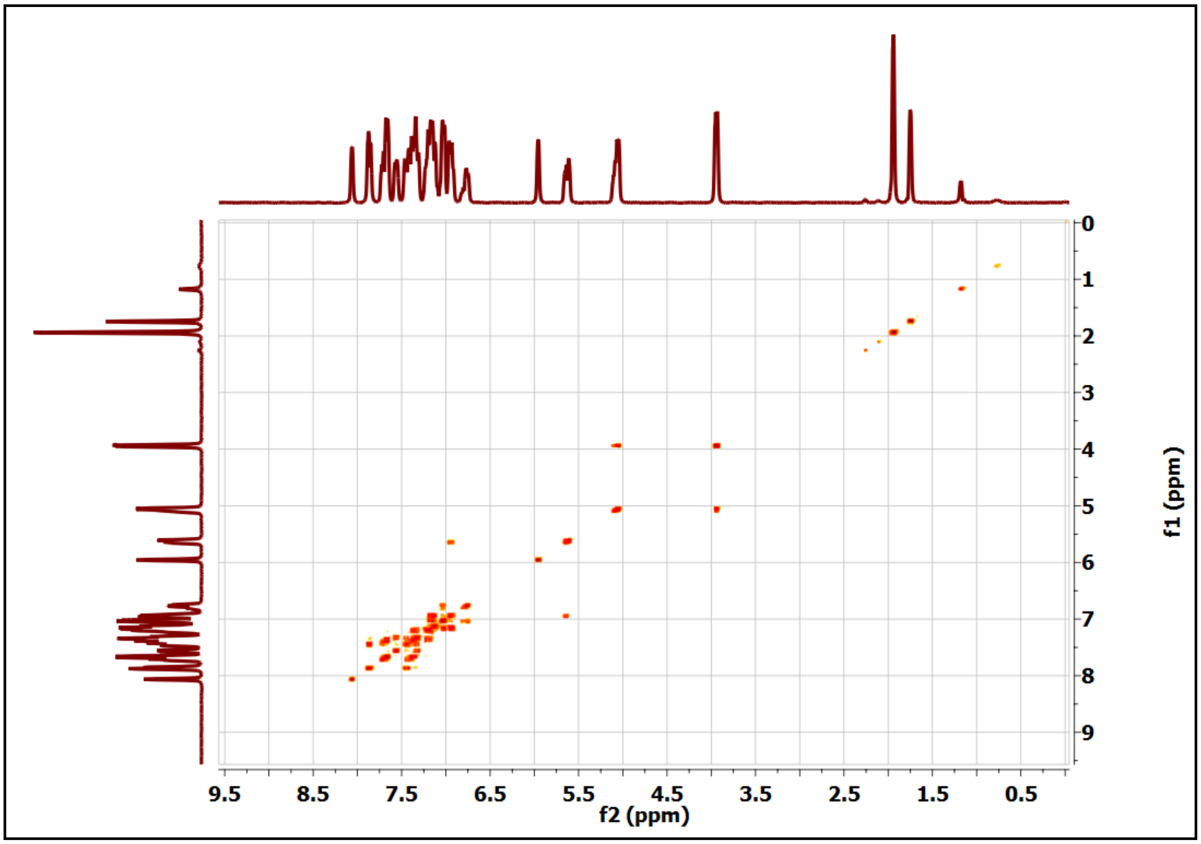
9.5
8.5
7.5
6.5
5.5
4.5
3.5
2.5
1.5
0.5
f2 (ppm)
0
1
2
3
4
5
6
7
8
9
f1 (ppm)

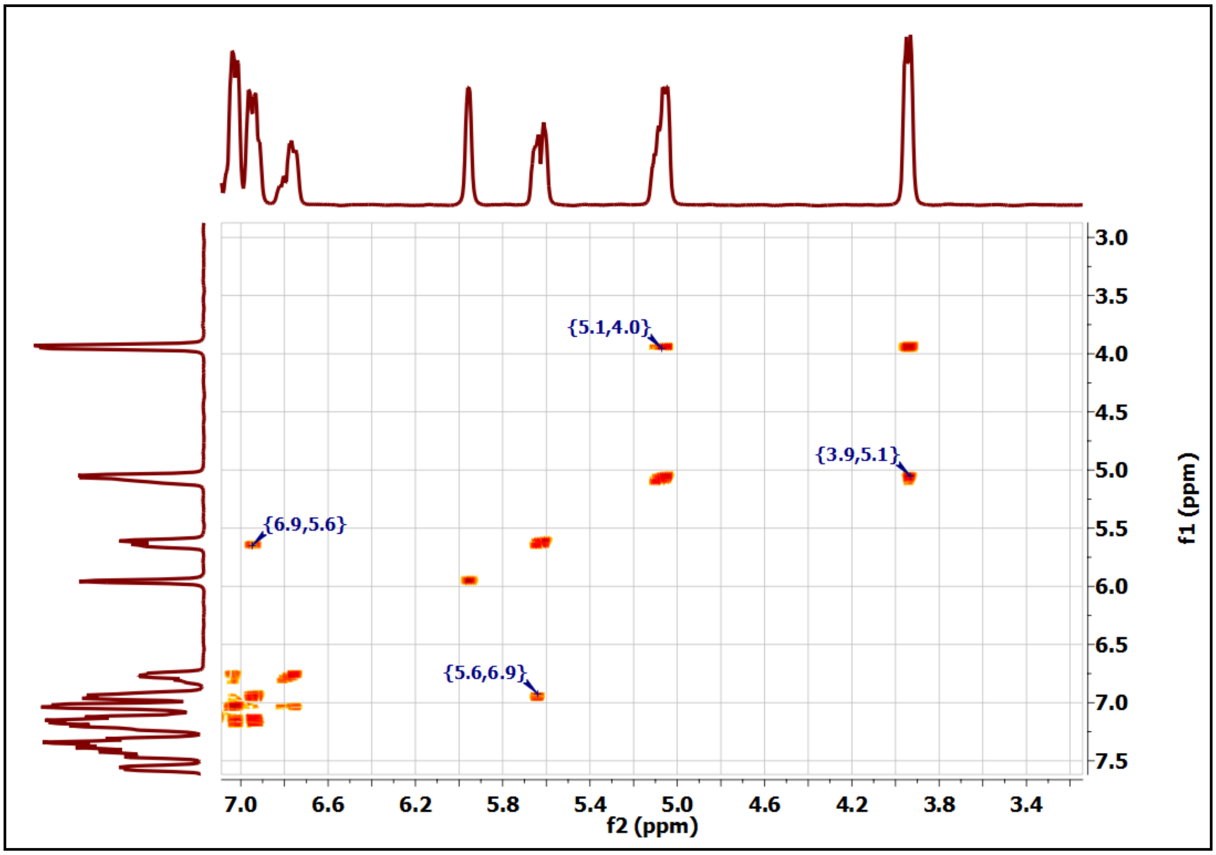
{5.1,4.0}
{3.9,5.1}
{6.9,5.6}
{5.6,6.9}
7.0
6.6
6.2
5.8
5.4
5.0
4.6
4.2
3.8
3.4
f2 (ppm)
3.0
3.5
4.0
4.5
5.0
5.5
6.0
6.5
7.0
7.5
f1 (ppm)

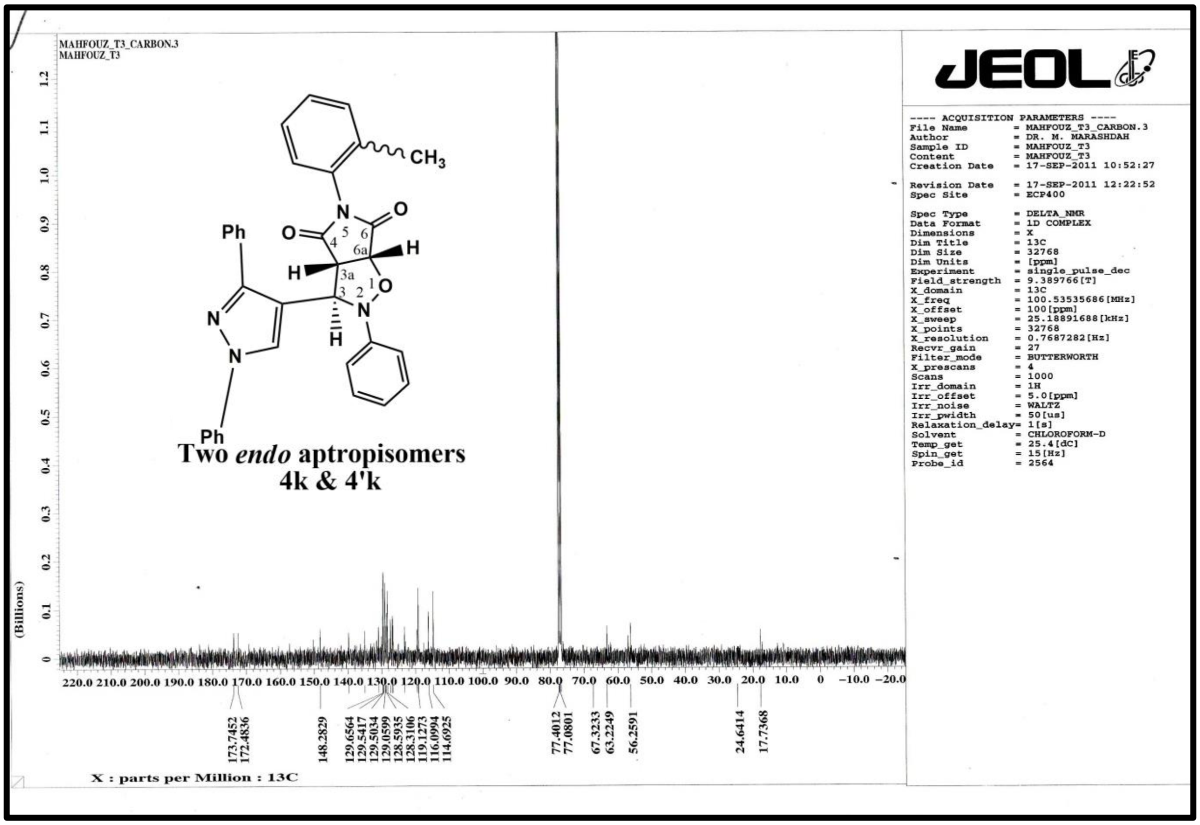
MAHFOUZ_T3_CARBON.3
MAHFOUZ_T3
Two endo aptropisomers
4k & 4'k
(Billions)
X : parts per Million : 13C
173.7452
172.4836
148.2829
129.6564
129.5417
129.5034
129.0599
128.5935
128.3106
119.1273
116.0994
114.6925
77.4012
77.0801
67.3233
63.2249
56.2591
24.6414
17.7368
JEOL
---- ACQUISITION PARAMETERS ----
File Name = MAHFOUZ_T3_CARBON.3
Author = DR. M. MARASHDAH
Sample ID = MAHFOUZ_T3
Content = MAHFOUZ_T3
Creation Date = 17-SEP-2011 10:52:27
Revision Date = 17-SEP-2011 12:22:52
Spec Site = ECP400
Spec Type = DELTA_NMR
Data Format = 1D COMPLEX
Dimensions = X
Dim Title = 13C
Dim Size = 32768
Dim Units = [ppm]
Experiment = single_pulse_dec
Field_strength = 9.389766[T]
X_domain = 13C
X_freq = 100.53535686[MHz]
X_offset = 100[ppm]
X_sweep = 25.18891688[kHz]
X_points = 32768
X_resolution = 0.7687282[Hz]
Recvr_gain = 27
Filter_mode = BUTTERWORTH
X_prescans = 4
Scans = 1000
Irr_domain = 1H
Irr_offset = 5.0[ppm]
Irr_noise = WALTZ
Irr_pwidth = 50[us]
Relaxation_delay= 1[s]
Solvent = CHLOROFORM-D
Temp_get = 25.4[dC]
Spin_get = 15[Hz]
Probe_id = 2564

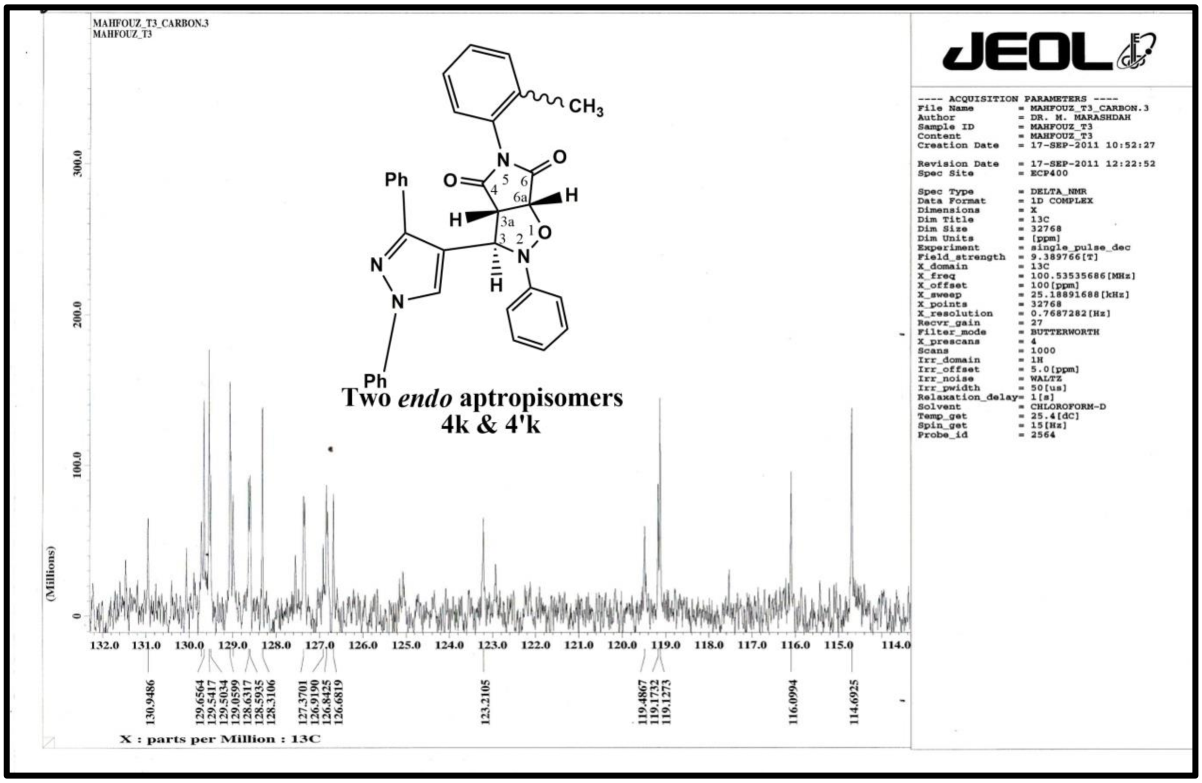
MAHFOUZ_T3_CARBON.3
MAHFOUZ_T3
Two endo aptropisomers
4k & 4'k
(Millions)
X : parts per Million : 13C
130.9486
129.6564
129.5417
129.5034
129.0599
128.6317
128.5935
128.3106
127.3701
126.9190
126.8425
126.6819
123.2105
119.4867
119.1732
119.1273
116.0994
114.6925
JEOL
---- ACQUISITION PARAMETERS ----
File Name = MAHFOUZ_T3_CARBON.3
Author = DR. M. MARASHDAH
Sample ID = MAHFOUZ_T3
Content = MAHFOUZ_T3
Creation Date = 17-SEP-2011 10:52:27
Revision Date = 17-SEP-2011 12:22:52
Spec Site = ECP400
Spec Type = DELTA_NMR
Data Format = 1D COMPLEX
Dimensions = X
Dim Title = 13C
Dim Size = 32768
Dim Units = [ppm]
Experiment = single_pulse_dec
Field_strength = 9.389766[T]
X_domain = 13C
X_freq = 100.53535686[MHz]
X_offset = 100[ppm]
X_sweep = 25.18891688[kHz]
X_points = 32768
X_resolution = 0.7687282[Hz]
Recvr_gain = 27
Filter_mode = BUTTERWORTH
X_prescans = 4
Scans = 1000
Irr_domain = 1H
Irr_offset = 5.0[ppm]
Irr_noise = WALTZ
Irr_pwidth = 50[us]
Relaxation_delay= 1[s]
Solvent = CHLOROFORM-D
Temp_get = 25.4[dC]
Spin_get = 15[Hz]
Probe_id = 2564

Exo-atropisomers **(5k,5'k)**: (0.02 g, 0.95%); white crystals; mp: 142-144°C. Mass spectrum (electron impact): m/e (%) 526.2(98.5),29 339.1(100), 76.9(17).

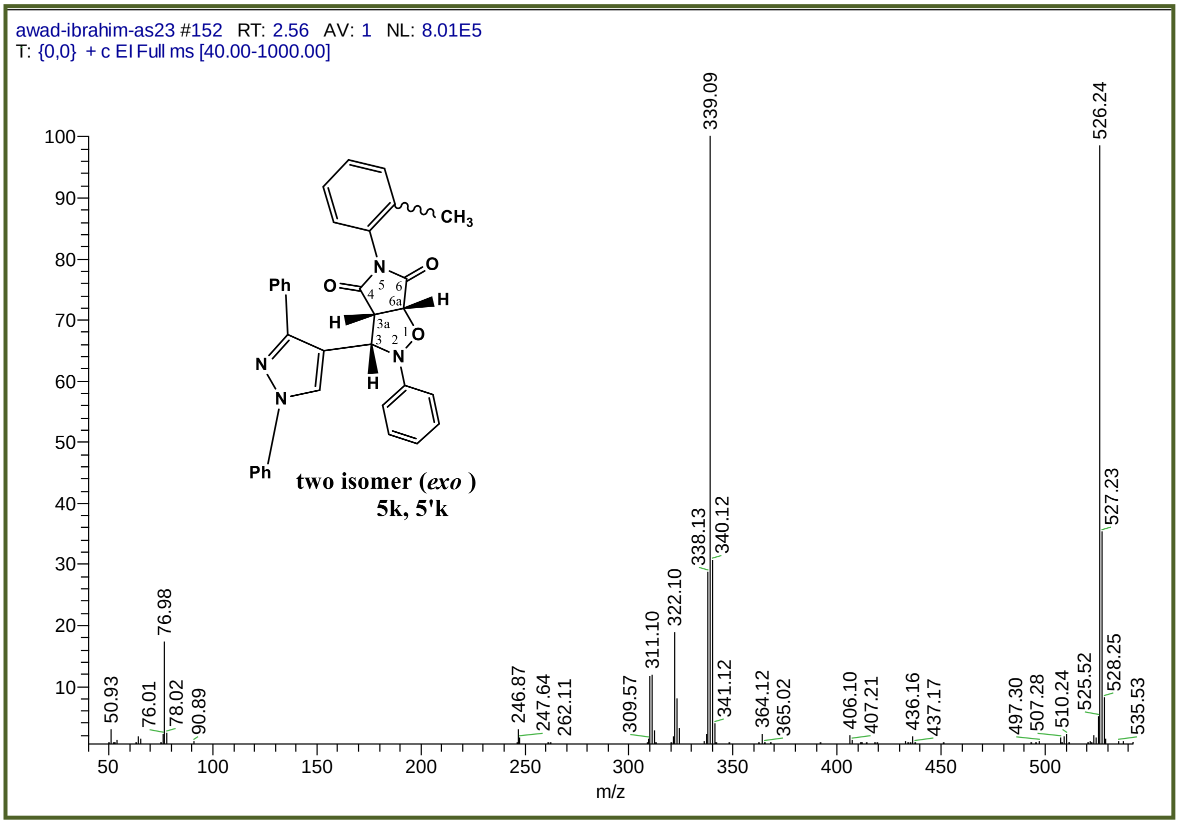

Cycloaddition with N-(4-chlorophenyl) maleimide (3d)

Formation of 5-(4-chlorophenyl)-3-(1,3-diphenyl-1H-pyrazol-4-yl)-2-phenyldihydro-2H-pyrrolo[3,4-d]isoxazole-4,6(5H,6aH)-dione $C_{32}H_{23}ClN_4O_3$.

Reaction mixture**(4d,5d)**: ^{1}H-NMR spectrum: δ ppm(400 MHz,$CDCl_3$) 4.1(d, *J* 7.2 Hz, 1H, H3a(*endo*)), 4.4(t, *J* 8 Hz, 1H,H3a(*exo*)), 4.88(d, *J* 9.2 Hz, 1H, H3(*exo*)), 5.48(d, *J* 7.2 Hz, 1H,H6a(*endo*)), 5.38(dd, *J* 8.2; 4.6Hz, 1H, H6a(*exo*)), 6.02(s, 1H,H3(*endo*)), 6.57-8.63(m, 40H, ArH), 9.86(s, 1H, NitroneCH=N).

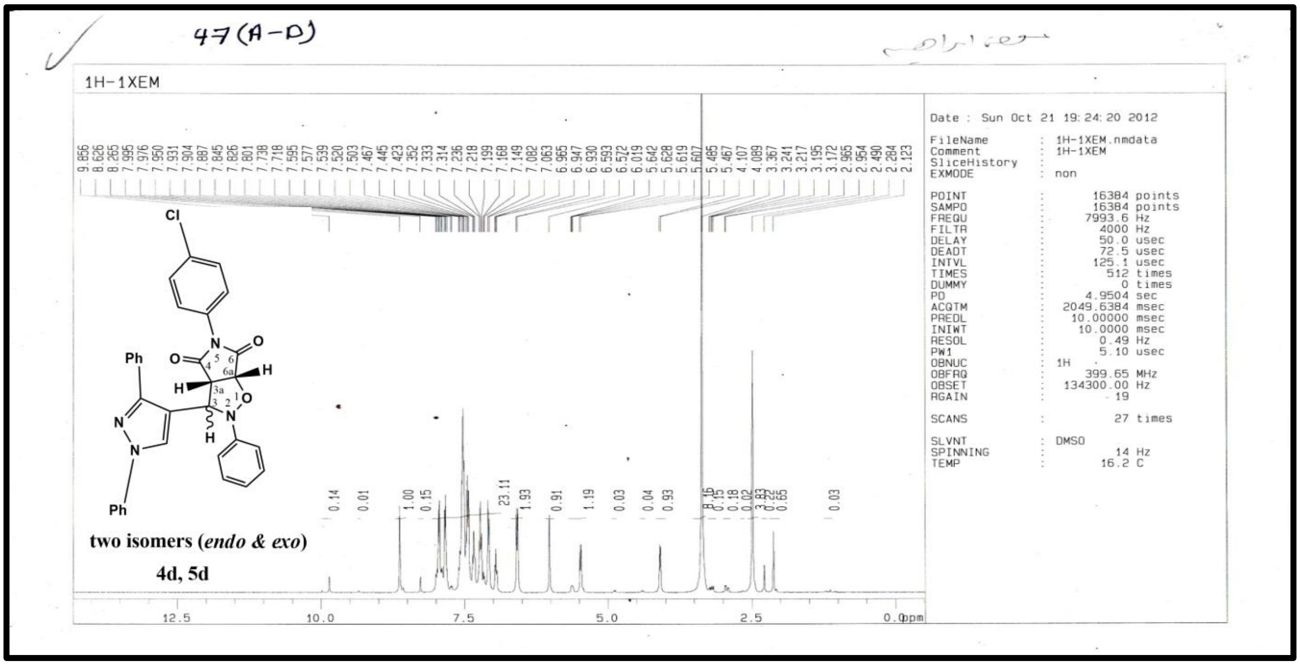

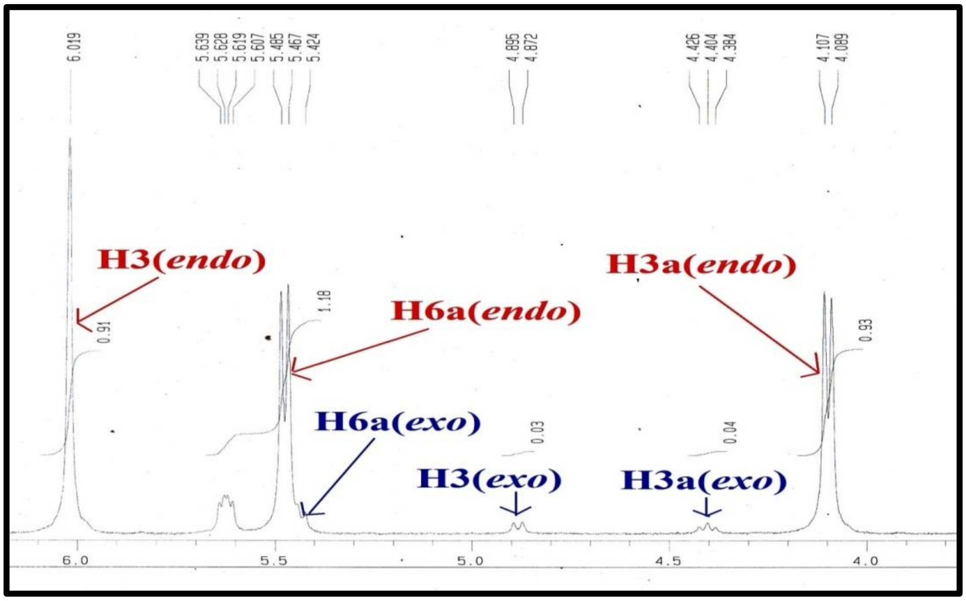

Endo-isomer **(4d)**: (1.1 g, 60%; white crystals; mp: 218-220°C. FTIR (KBr) (cm^{-1}): 3130(Ar. C-H), 2950(Aliph. C-H), 1727(C=O). ^{1}H-NMR spectrum: δ ppm(400 MHz, $CDCl_3$) 4(d,*J*7.2 Hz, 1H, H3a), 5.1(d, *J* 7.2 Hz, 1H, H6a), 6.1(s, 1H, H3), 6.6-8.2(m, 20H, Ar-H). ^{13}C{H}NMR spectrum: δ ppm(100.5 MHz, CDCl3) 55.9, 63.3(3 Aliphatic **C**); 114.6(2), 119.1(2), 119.2, 123.2, 126.8, 127.3, 127.3(2), 128.3(2), 128.6, 129. (2), 129.2(2), 129.2, 129.5(2), 129.5(2), 132.2, 134.9, 139.7, 148.2, 150.2(27 Aromatic **C**); 172.4, 173.5(2**C**=O). Mass spectrum(electron impact): m/e(%) 338.7(26.2), 206.7(34.1), 208.9(14.9), 76.9(100). Anal. Calcd for ($C_{32}H_{23}ClN_4O_3$)(%): C, 70.26; H, 4.24; N, 10.24. Found; C,70.16; H, 3.90; N, 10.11.

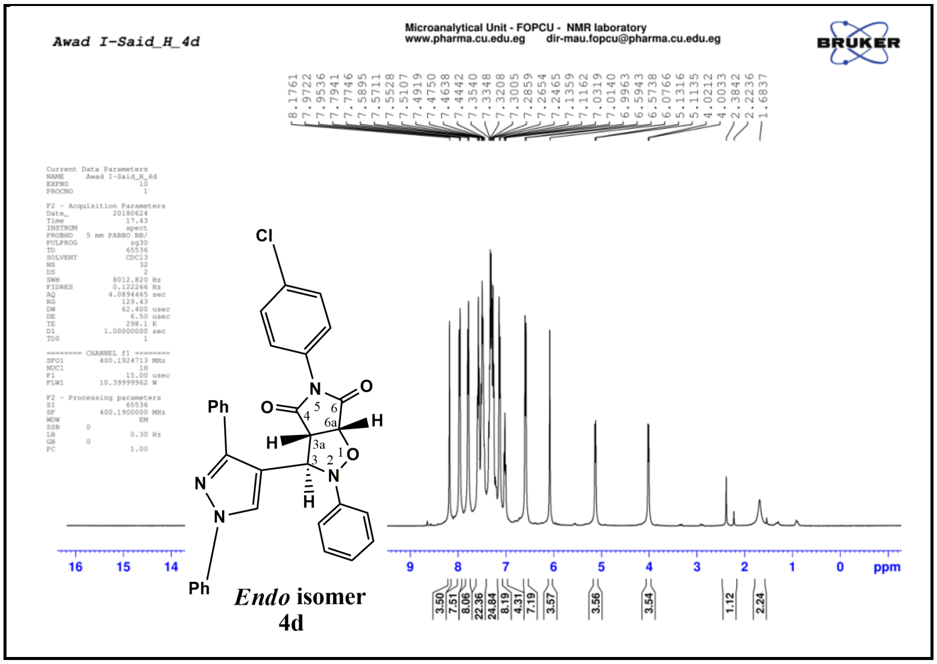

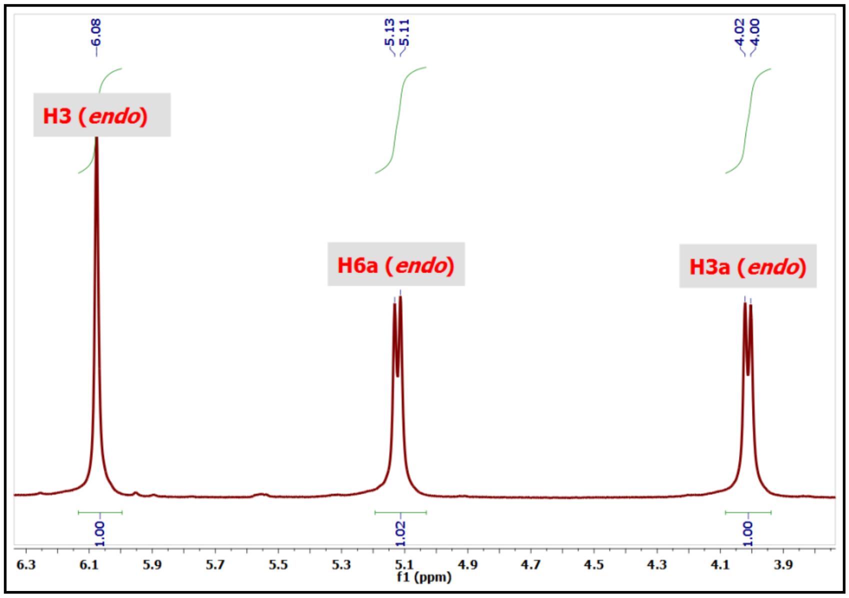

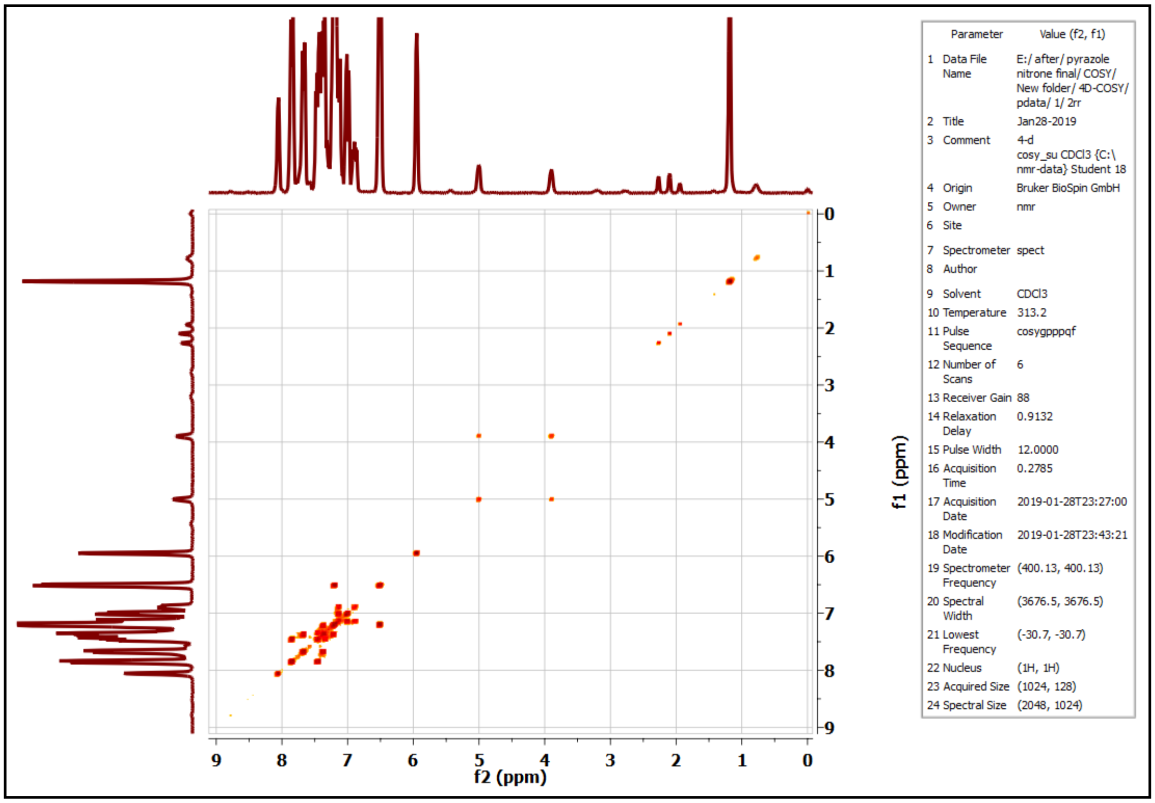
Parameter Value (f2, f1)
1 Data File Name E:/ after/ pyrazole nitrone final/ COSY/ New folder/ 4D-COSY/ pdata/ 1/ 2rr
2 Title Jan28-2019
3 Comment 4-d cosy_su CDCl3 {C:\ nmr-data} Student 18
4 Origin Bruker BioSpin GmbH
5 Owner nmr
6 Site
7 Spectrometer spect
8 Author
9 Solvent CDCl3
10 Temperature 313.2
11 Pulse Sequence cosygpppqf
12 Number of Scans 6
13 Receiver Gain 88
14 Relaxation Delay 0.9132
15 Pulse Width 12.0000
16 Acquisition Time 0.2785
17 Acquisition Date 2019-01-28T23:27:00
18 Modification Date 2019-01-28T23:43:21
19 Spectrometer Frequency (400.13, 400.13)
20 Spectral Width (3676.5, 3676.5)
21 Lowest Frequency (-30.7, -30.7)
22 Nucleus (1H, 1H)
23 Acquired Size (1024, 128)
24 Spectral Size (2048, 1024)
f2 (ppm)
f1 (ppm)

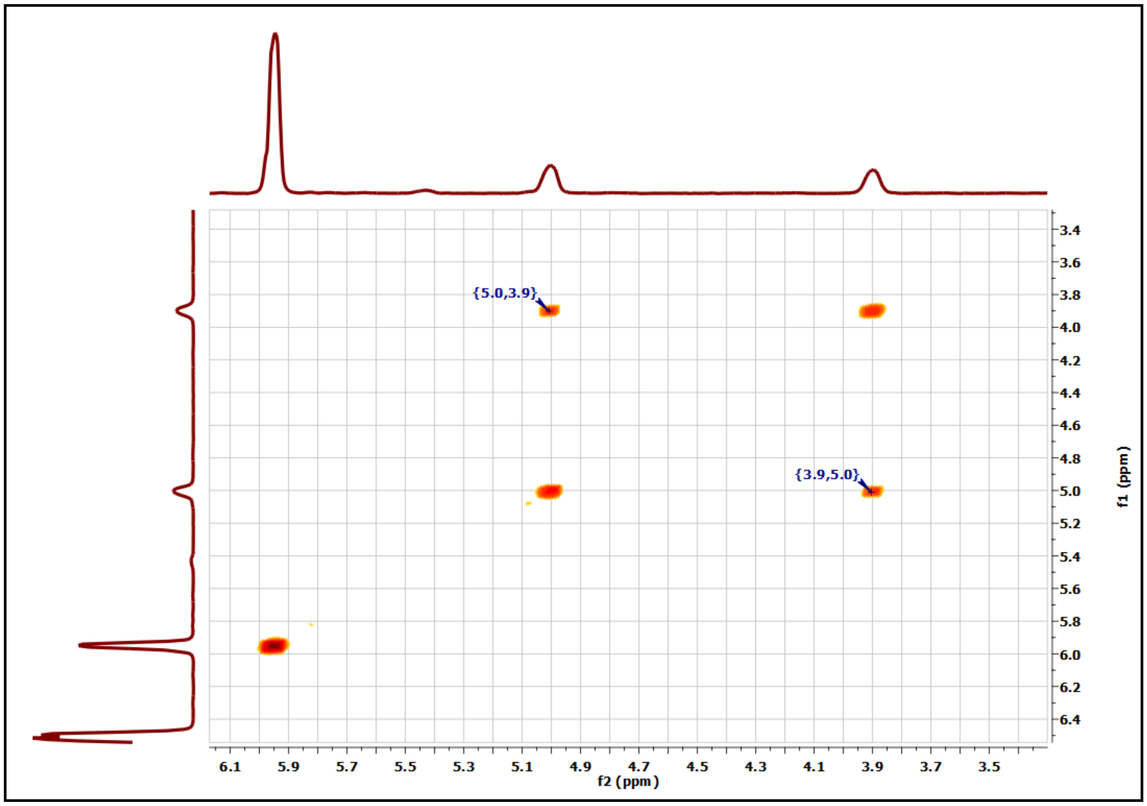
{5.0,3.9}
{3.9,5.0}
f2 (ppm)
f1 (ppm)

Assiut Univercity Central Lab

12/25/11 10:14:29 PM Page 1

File: PAO13 Date Run: 12-17-2011 Time Run: 22:44:22
Sample: prof Dr/Galal Elnagar
Instrument: JEOL JMS600 Run By: Souzan
Inlet: My Inlet Ionization mode: EI+ Printed by: Souzan

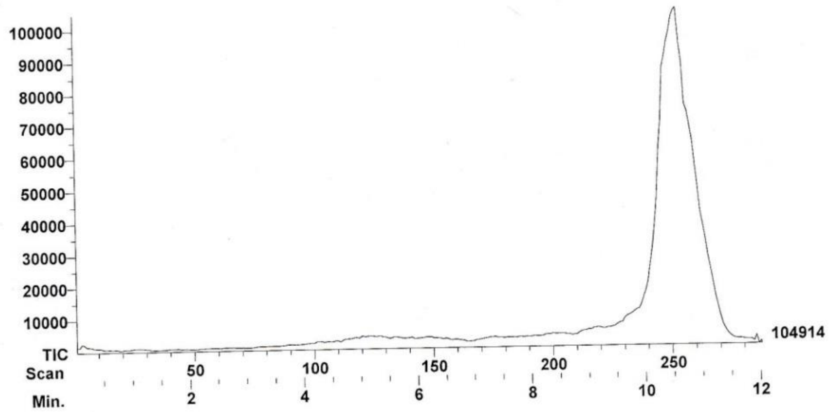

Scan: 69-287 R.T.: 7:27.297
Base: m/z 77; .1%FS TIC: 11040 (Max Inten : 1021) #Ions: 508

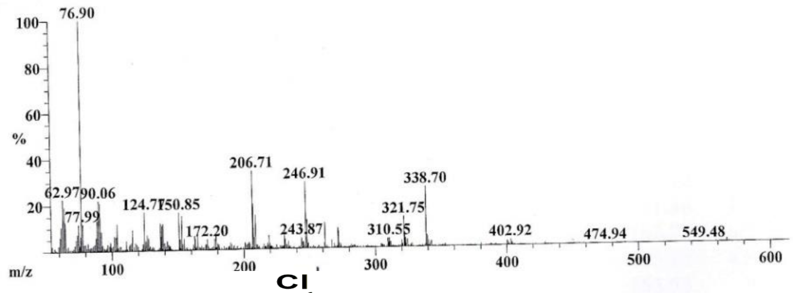

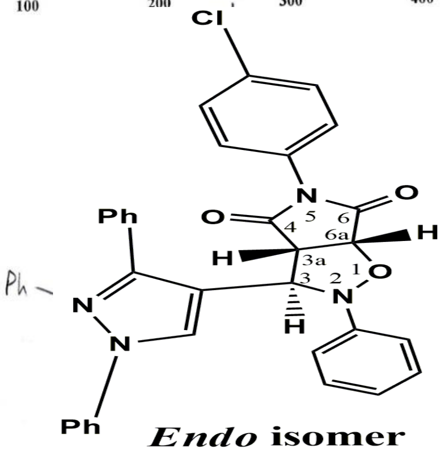

***Endo* isomer**
4d

Exo-isomer**(5d)**: (0.0008 g, 0.18%; white crystals; mp: 122-124°C. Mass spectrum(electron impact): m/e(%) 548.1(86.7), 546(30.1), 339.2(24.5), 193.8(100).

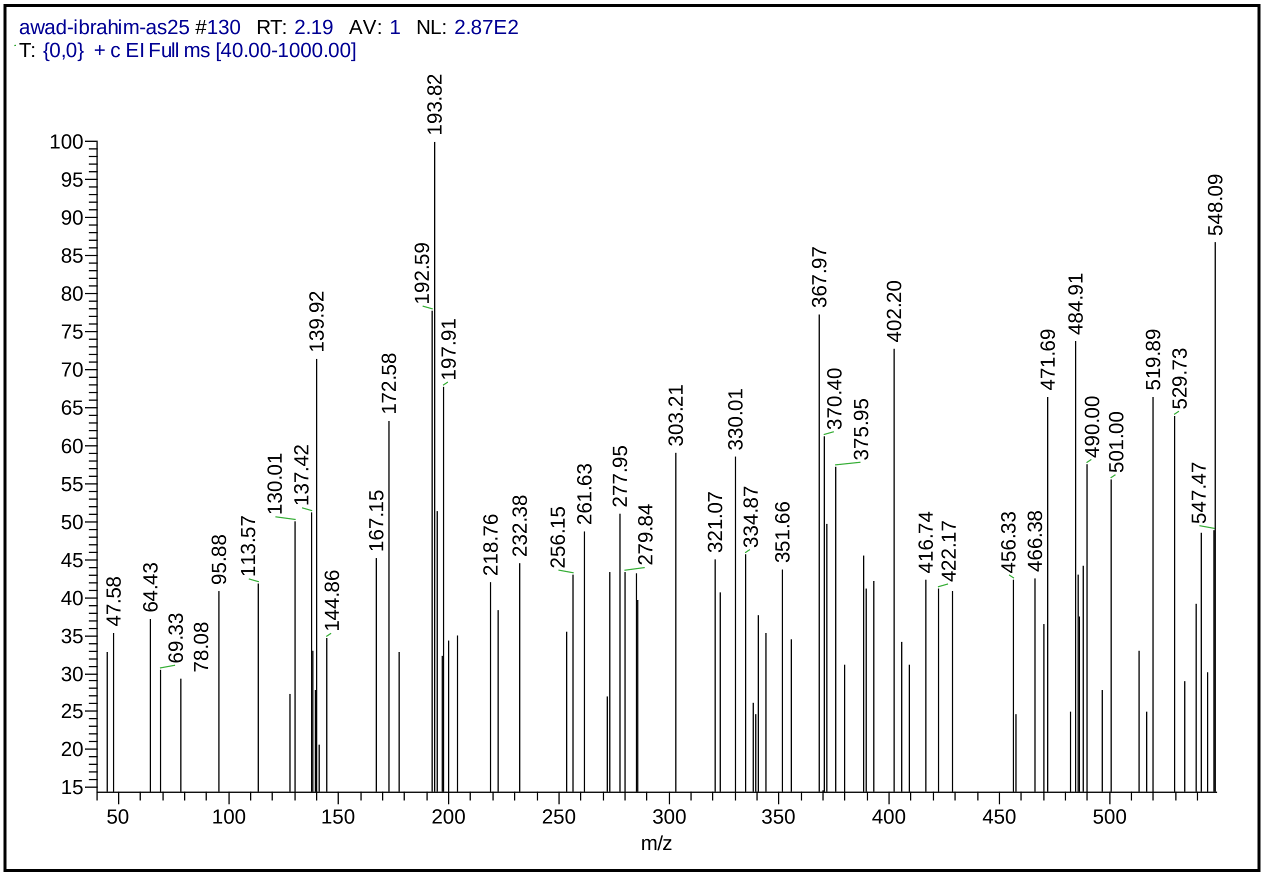

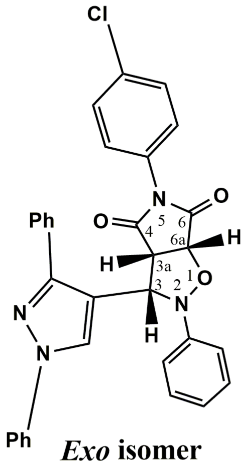

***Exo* isomer**
5d

Cycloaddition with N-(3-chlorophenyl) maleimide (3e)

Formation of 5-(3-chlorophenyl)-3-(1,3-diphenyl-1H-pyrazol-4-yl)-2-phenyldihydro-2H-pyrrolo[3,4-d]isoxazole-4,6(5H,6aH)-dione $C_{32}H_{23}ClN_4O_3$.

Reaction mixture **(4e,5e)**:^{1}H-NMR spectrum: δ ppm(400 MHz, $CDCl_3$) 4.1(d, *J* 7.2 Hz, 1H, H3a(*endo*)), 4.42(t, *J* 9.2 Hz, 1H, H3a(*exo*)), 4.89(d, *J* 9.2 Hz, 1H, H3(*exo*)), 5.44-5.49(m, 2H, H6a(*exo,endo*)), 6.06(s, 1H, H3(*endo*)), 6.4-8.7(m, 40H, Ar H), 9.98(s, 1H, Nitrone C<u>H</u>=N).

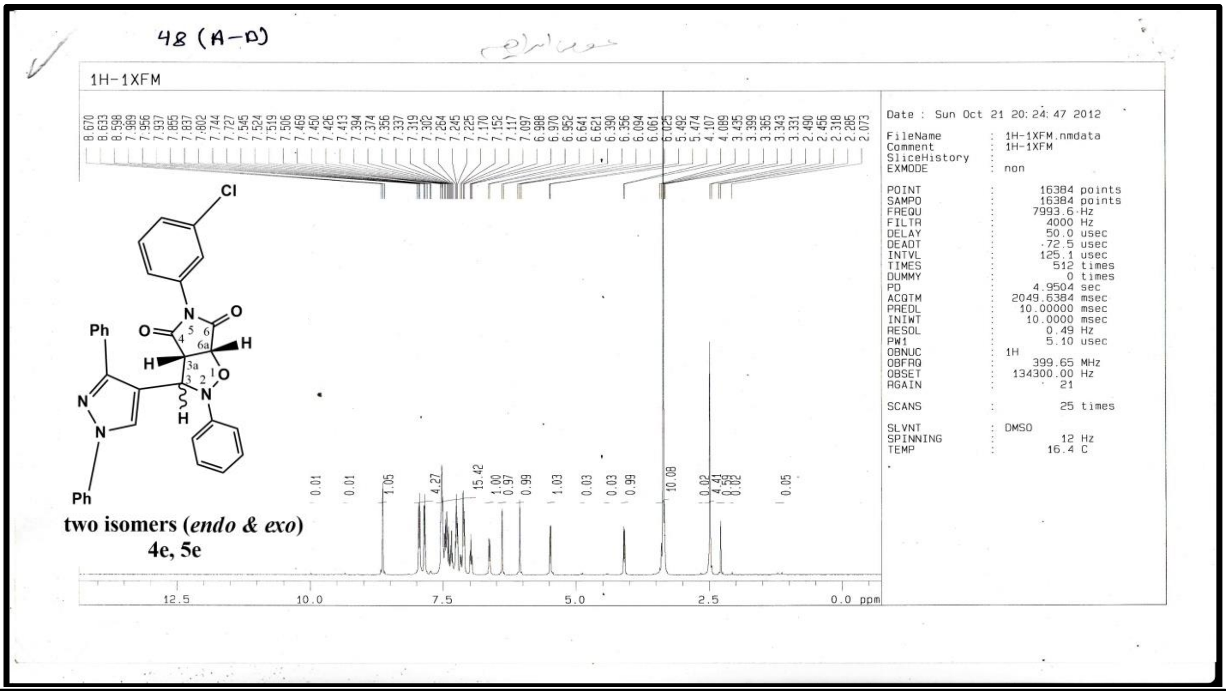

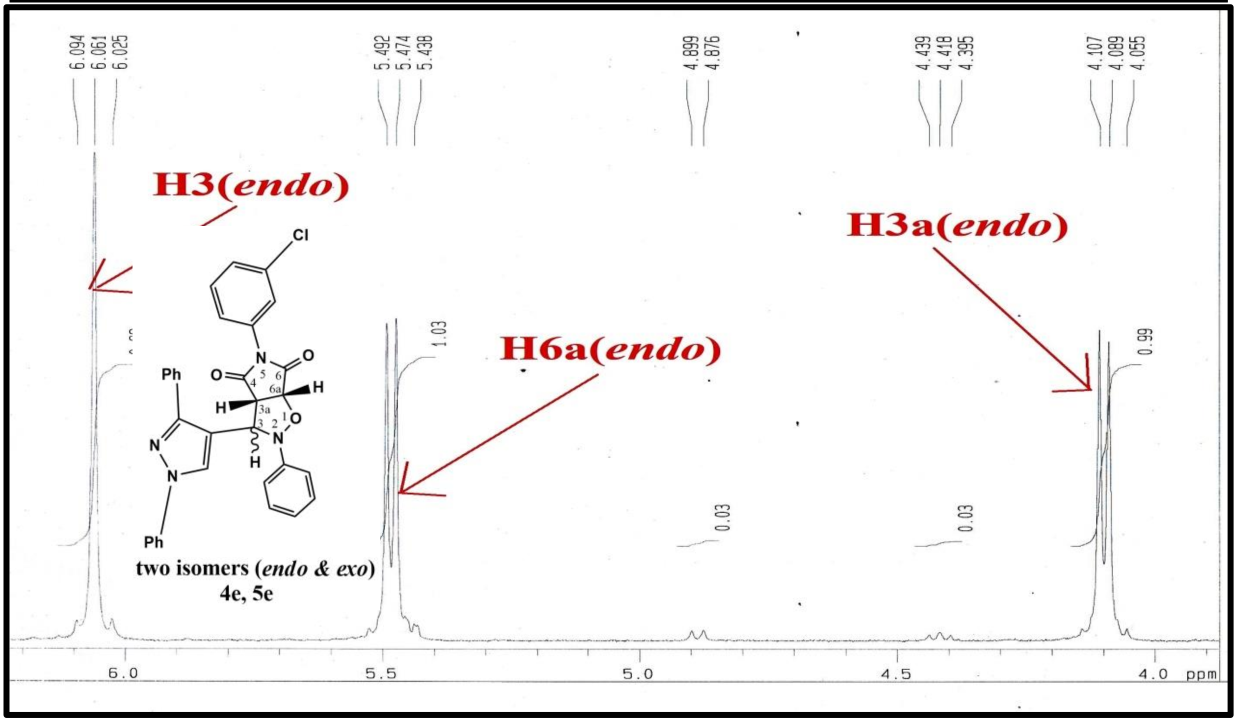

Endo-isomer **(4e)**: (o.96 g, 55%); white crystals; mp: 230-231°C. FTIR (KBr)(cm^{-1}); 3080(Ar.C-H), 2972(Aliph. C-H), 1720(C=O). ^{1}H-NMR spectrum: δ ppm(400 MHz, $CDCl_3$) 4(d, *J* 6 Hz, 1H, H3a), 5.1(d, *J* 6.1 Hz, 1H, H6a), 6.1(s, 1H, H3), 6.5-8.2(m, 20H, Ar-H). ^{13}C{H}NMR spectrum: δ ppm(100.5 MHz, CDCl3) 55.9, 63.3(3Aliphatic **C**); 114.6(2), 119.1(2), 119.2, 123.5, 124.3, 126.5, 126.8, 127.4, 128.3(2), 128.6, 129(2), 129.3, 129.5(2), 129.6(2), 129.9, 131.8, 132.2, 134.7, 139.7, 148.2, 150.2(27 Aromatic **C**); 172.2, 173.4(2 **C**=O). Anal. Calcd for ($C_{32}H_{23}ClN_4O_3$) (%): C, 70.26; H, 4.24; N, 10.24. Found; C, 70.20; H, 4.02; N, 10.35.

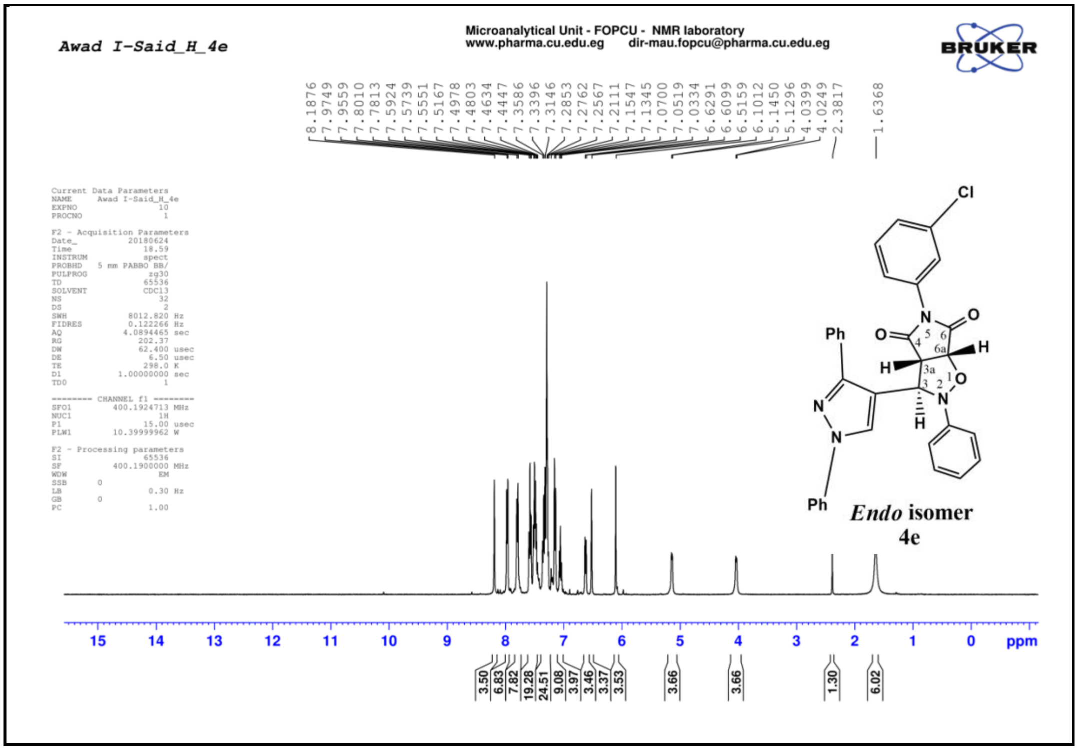

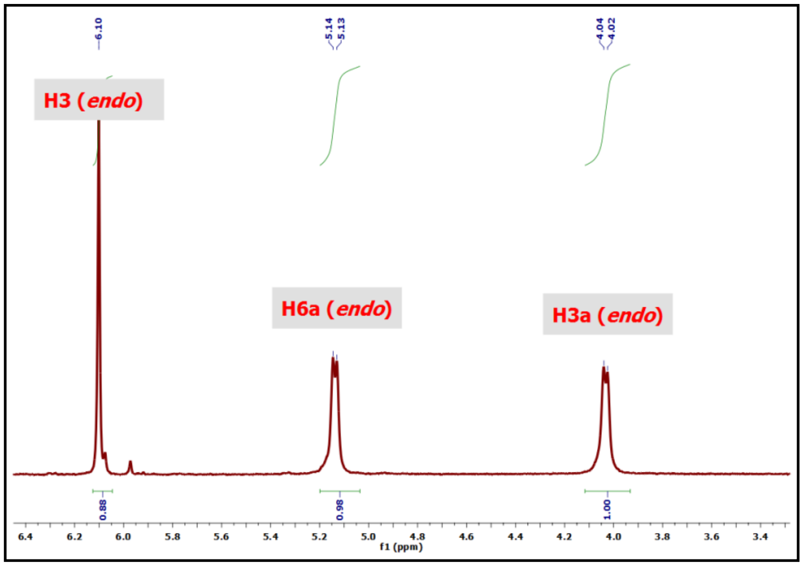

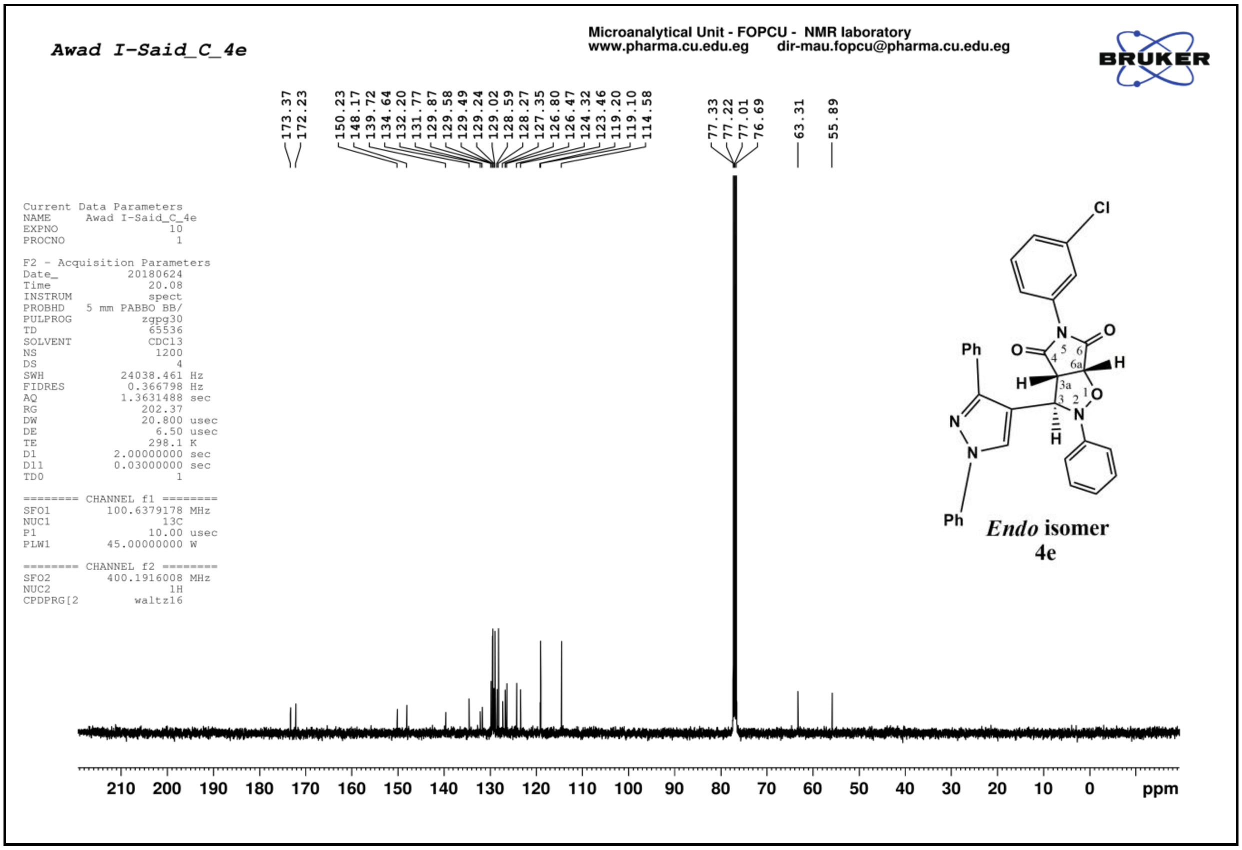
Awad I-Said_C_4e
Microanalytical Unit - FOPCU - NMR laboratory
www.pharma.cu.edu.eg dir-mau.fopcu@pharma.cu.edu.eg
BRUKER
173.37
172.23
150.23
148.17
139.72
134.64
132.20
131.77
129.87
129.58
129.49
129.24
129.02
128.59
128.27
127.35
126.80
126.47
124.32
123.46
119.20
119.10
114.58
77.33
77.22
77.01
76.69
63.31
55.89
Current Data Parameters
NAME Awad I-Said_C_4e
EXPNO 10
PROCNO 1
F2 - Acquisition Parameters
Date_ 20180624
Time 20.08
INSTRUM spect
PROBHD 5 mm PABBO BB/
PULPROG zgpg30
TD 65536
SOLVENT CDCl3
NS 1200
DS 4
SWH 24038.461 Hz
FIDRES 0.366798 Hz
AQ 1.3631488 sec
RG 202.37
DW 20.800 usec
DE 6.50 usec
TE 298.1 K
D1 2.00000000 sec
D11 0.03000000 sec
TD0 1
======== CHANNEL f1 ========
SFO1 100.6379178 MHz
NUC1 13C
P1 10.00 usec
PLW1 45.00000000 W
======== CHANNEL f2 ========
SFO2 400.1916008 MHz
NUC2 1H
CPDPRG[2 waltz16
Endo isomer
4e
ppm

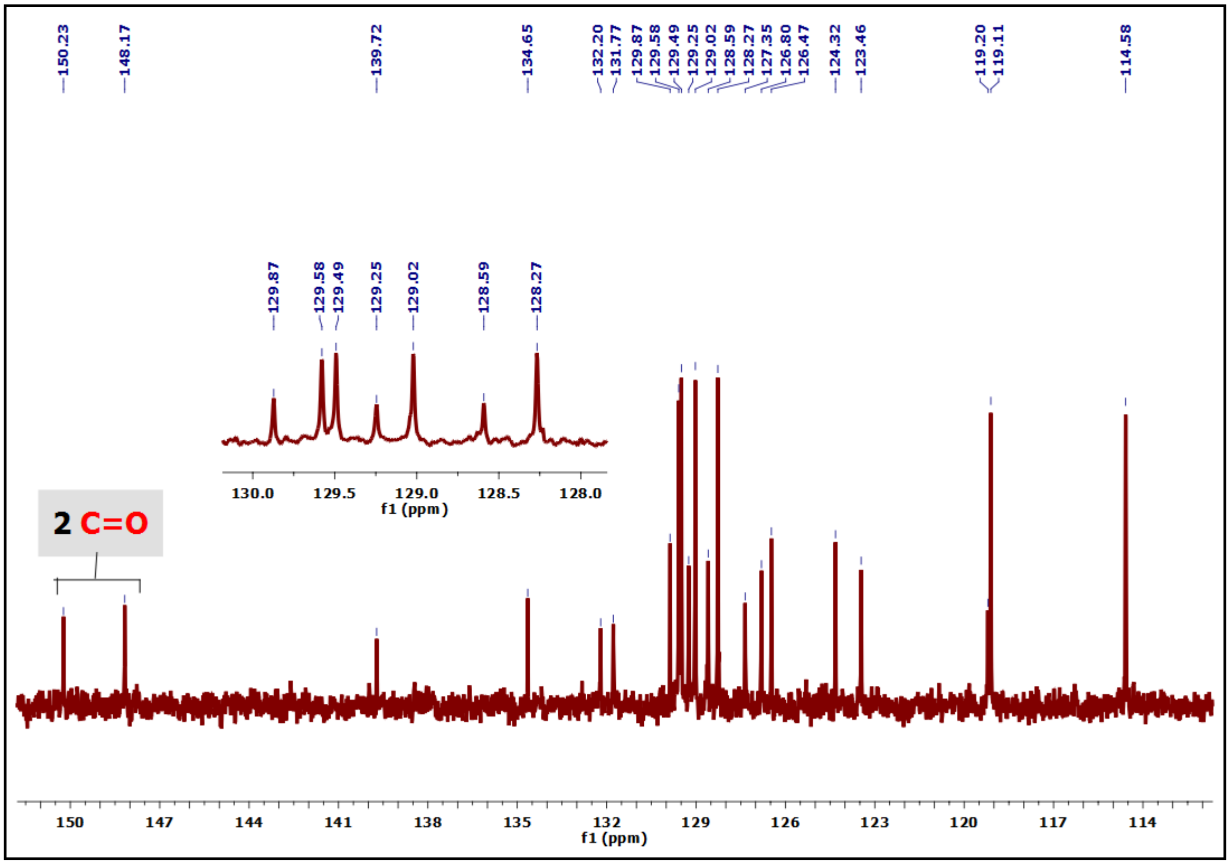
150.23
148.17
139.72
134.65
132.20
131.77
129.87
129.58
129.49
129.25
129.02
128.59
128.27
127.35
126.80
126.47
124.32
123.46
119.20
119.11
114.58
2 C=O
f1 (ppm)

Cycloaddition with N-(2-chlorophenyl) maleimide (3l)

Formation of 5-(2-chlorophenyl)-3-(1,3-diphenyl-1H-pyrazol-4-yl)-2-phenyldihydro-2H-pyrrolo[3,4-d]isoxazole-4,6(5H,6aH)-dione $C_{32}H_{23}ClN_4O_3$.

Reaction mixture **(4l,4'l,5l,5'l)**: ^{1}H-NMR spectrum: δ(400 MHz, $CDCl_3$) 4.19(d, *J* 7.2Hz, 1H, H3a(**4'l**)), 4.4(t, *J* 8Hz, 1H, H3a(**5l**)), 4.53(d, *J* 7.2Hz, 1H, H3a(**4l**)), 4.6(t, *J* 8.4Hz, 1H, H3a(**5'l**)), 4.91(d, *J* 8.8 Hz, 1H, H3 (**5'l**)), 4.96(d, *J* 8.4 Hz, 1H, H3 (**5l**)), 5.38(d, *J* 7.2 Hz, 1H, H6a(**5l**)), 5.55(s, 1H, H3(**4'l**)), 5.59(d, *J* 7.2 Hz, 1H, H6a(**4'l**)), 5.64(d, *J* 7.6 Hz, 1H, H6'(**4l**)), 5.71(d, *J* 7.2 Hz, 1H, H6a (**4'l**)), 5.73(d, 1H, H6a (**5'l**)), 6.12(s, 1H, H3(**4l**)), 6.4-8.7 (m, 80H, Ar H), 9.99 (s, 1H, Nitrone C<u>H</u>=N).

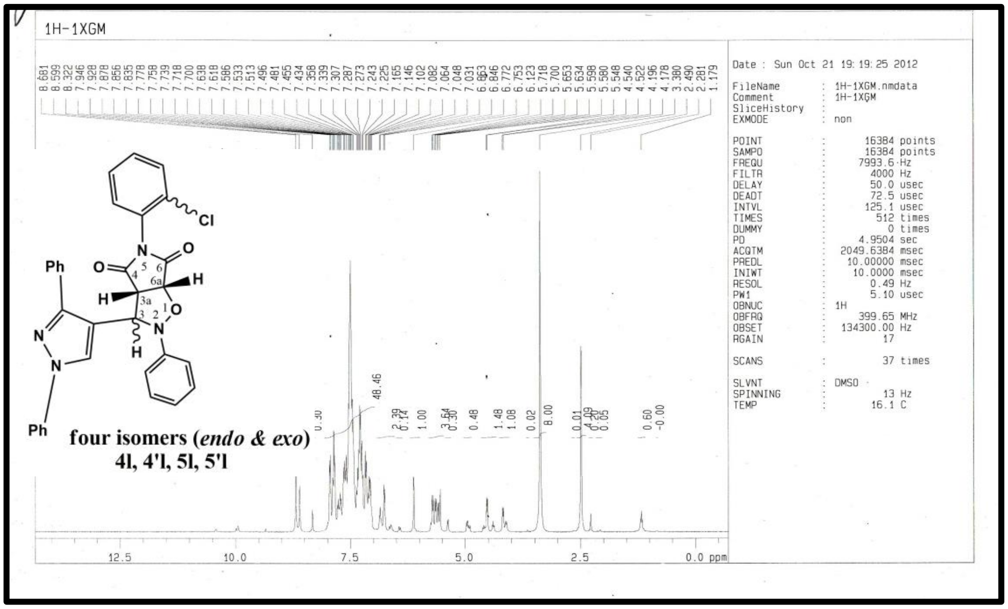

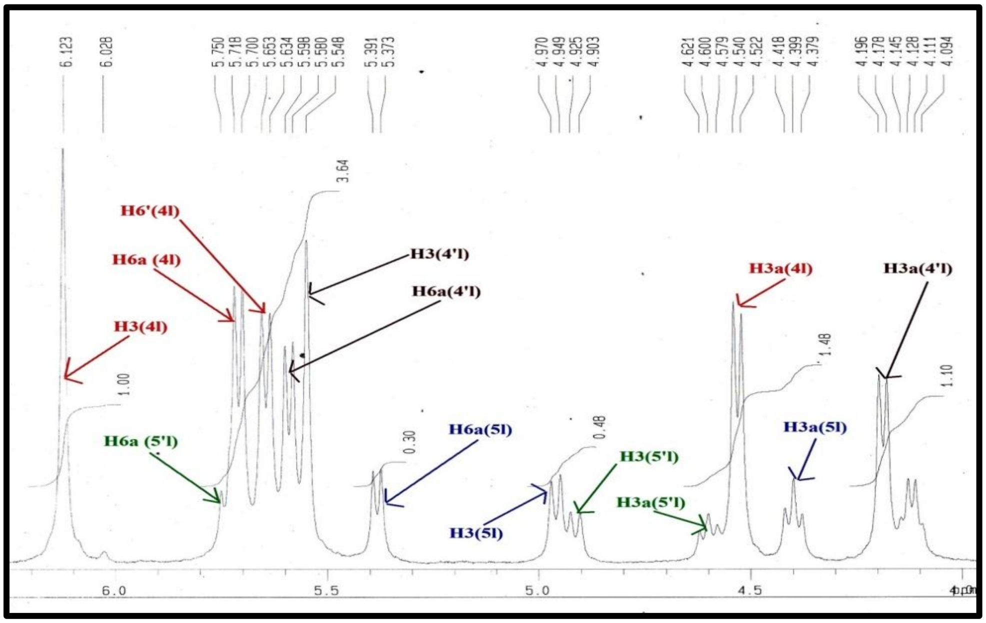

Endo- atropisomers **(4l,4'l)**: (1.2 g, 65%; white crystals; mp: 205-207°C. ^{1}H-NMR spectrum: δ ppm(400 MHz, $CDCl_3$) 3.87(d, J = 5.9 Hz, 1H, H3a(**4'l**)), 3.95(d, J = 6.2 Hz, 1H, H3a(**4l**)), 5.08(d, J = 5.8 Hz, 2H, H6a (4l,4'l)), 5.56(s, 1H, H3 (4'l)), 5.83(d, J = 6.5 Hz, 1H, **H6' (4l)**), δ 5.91(s, 1H, H3 (4l)), 6.75-8.02(m, 39H, ArH).

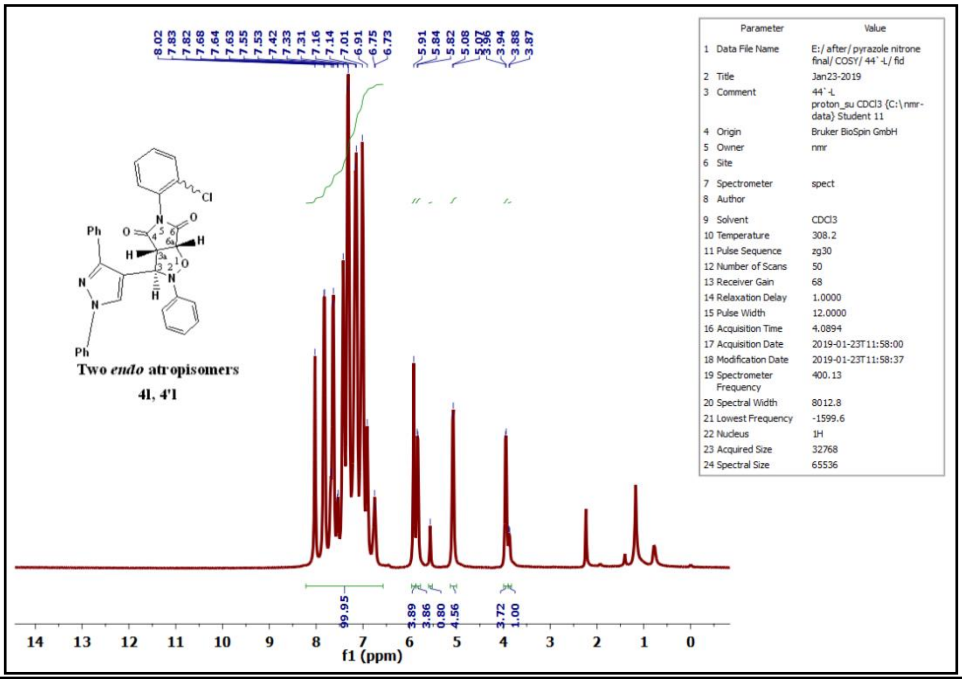

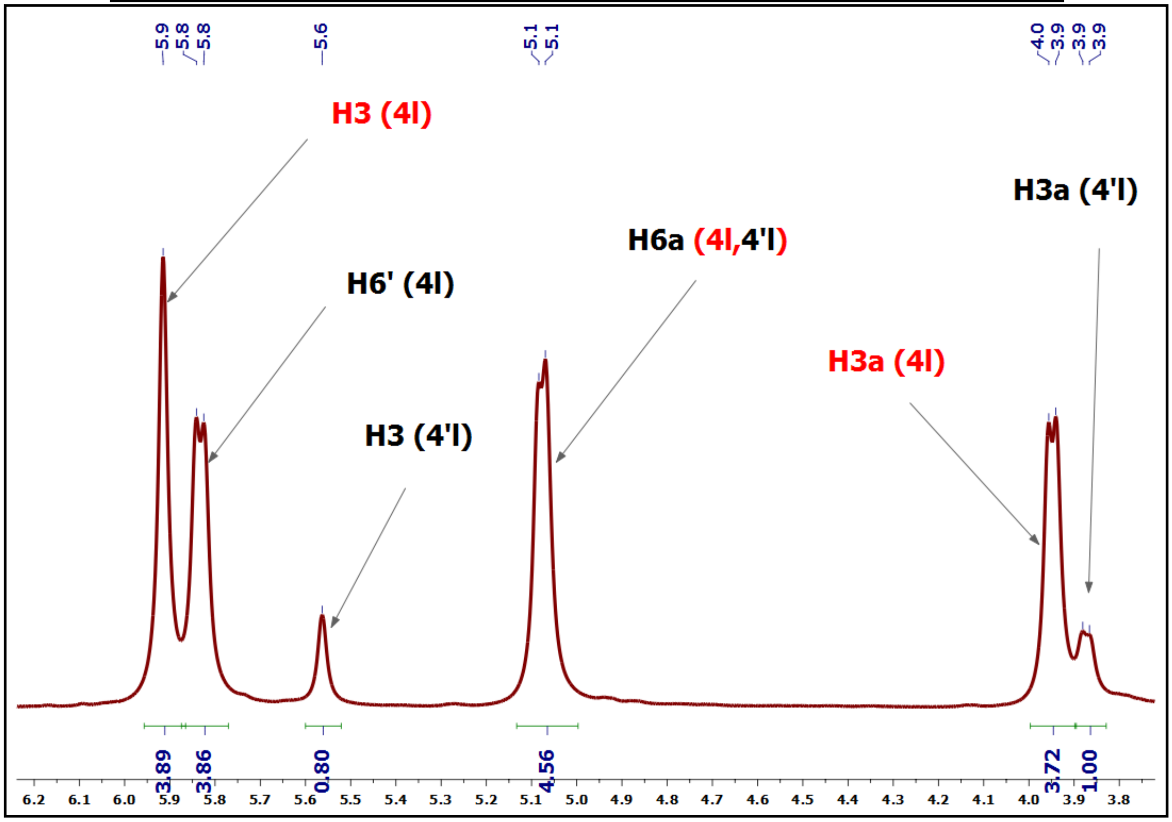

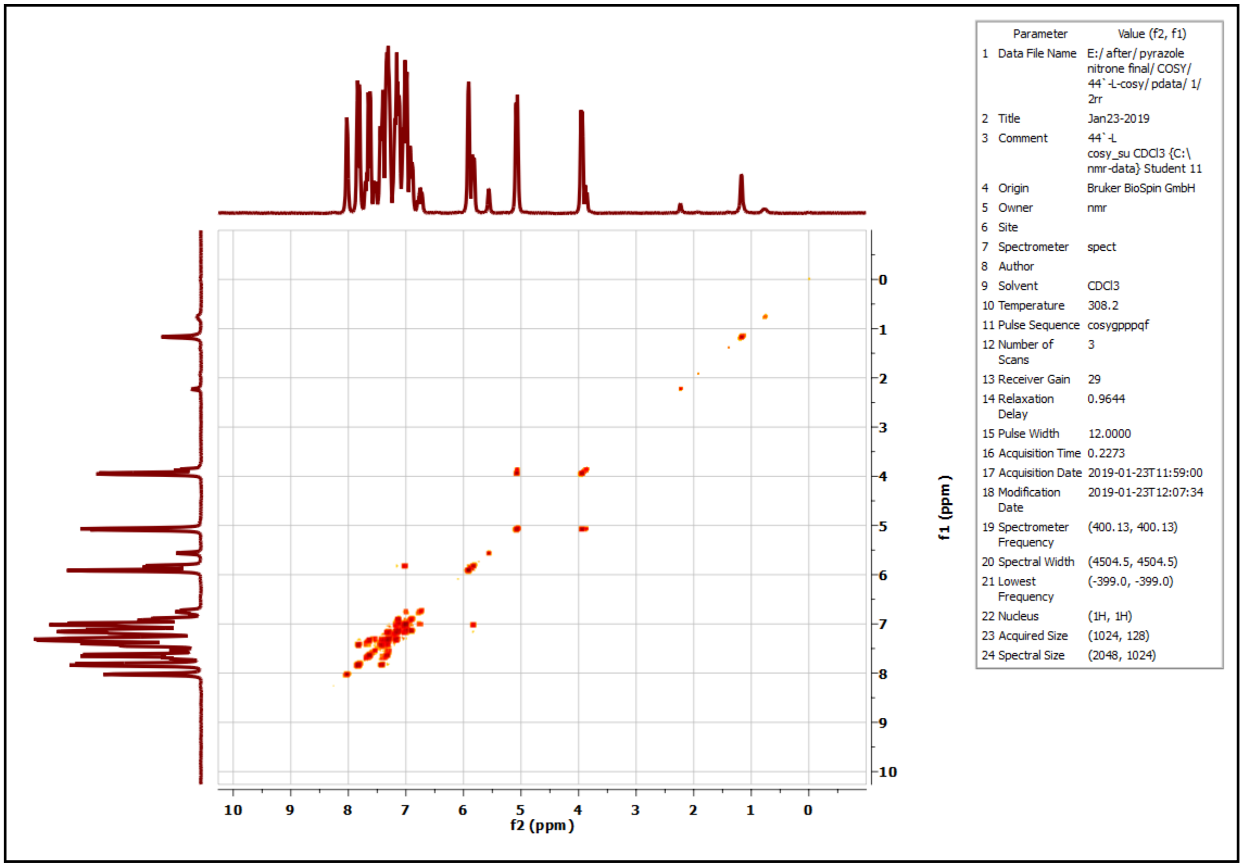

Parameter Value (f2, f1)
1 Data File Name E:/ after/ pyrazole nitrone final/ COSY/ 44`-L-cosy/ pdata/ 1/ 2rr
2 Title Jan23-2019
3 Comment 44`-L cosy_su CDCl3 {C:\ nmr-data} Student 11
4 Origin Bruker BioSpin GmbH
5 Owner nmr
6 Site
7 Spectrometer spect
8 Author
9 Solvent CDCl3
10 Temperature 308.2
11 Pulse Sequence cosygpppqf
12 Number of Scans 3
13 Receiver Gain 29
14 Relaxation Delay 0.9644
15 Pulse Width 12.0000
16 Acquisition Time 0.2273
17 Acquisition Date 2019-01-23T11:59:00
18 Modification Date 2019-01-23T12:07:34
19 Spectrometer Frequency (400.13, 400.13)
20 Spectral Width (4504.5, 4504.5)
21 Lowest Frequency (-399.0, -399.0)
22 Nucleus (1H, 1H)
23 Acquired Size (1024, 128)
24 Spectral Size (2048, 1024)
f2 (ppm)
f1 (ppm)

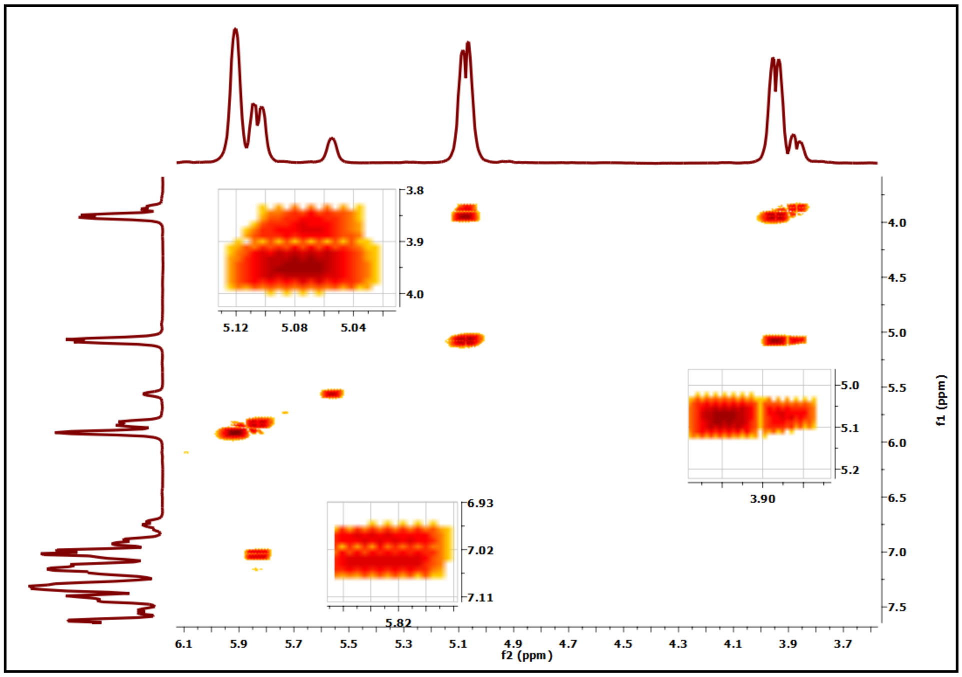

f2 (ppm)
f1 (ppm)

Exo atropisomers **(5l,5'l)**: (0.05g, 0.36 %; white crystals; m.p.; 174-176^oC. ^1H-NMR spectrum: δ (400 MHz, $CDCl_3$) 4.04(t, *J* 8.4Hz, 1H, H3a(**5'l**)), 4.1(t, *J* 8.4Hz, 1H, H3a(**5l**)), 4.98(d, *J* 8.4 Hz, 1H, H3 (**5'l**)), 5.22(d, *J* 8.8 Hz, 1H, H3 (**5l**)), 5.23(d, *J* 7.2 Hz, 1H, H6a(**5'l**)), 5.4 (d, *J* 8.4 Hz, 1H, H6a (**5l**)), 6.7-8.1 (m, 40H, ArH). Anal. Calcd for ($C_{32}H_{23}ClN_4O_3$)(%): C, 70.26; H, 4.24; N, 10.24. Found; C, 70.47; H, 4.31; N, 10.12.

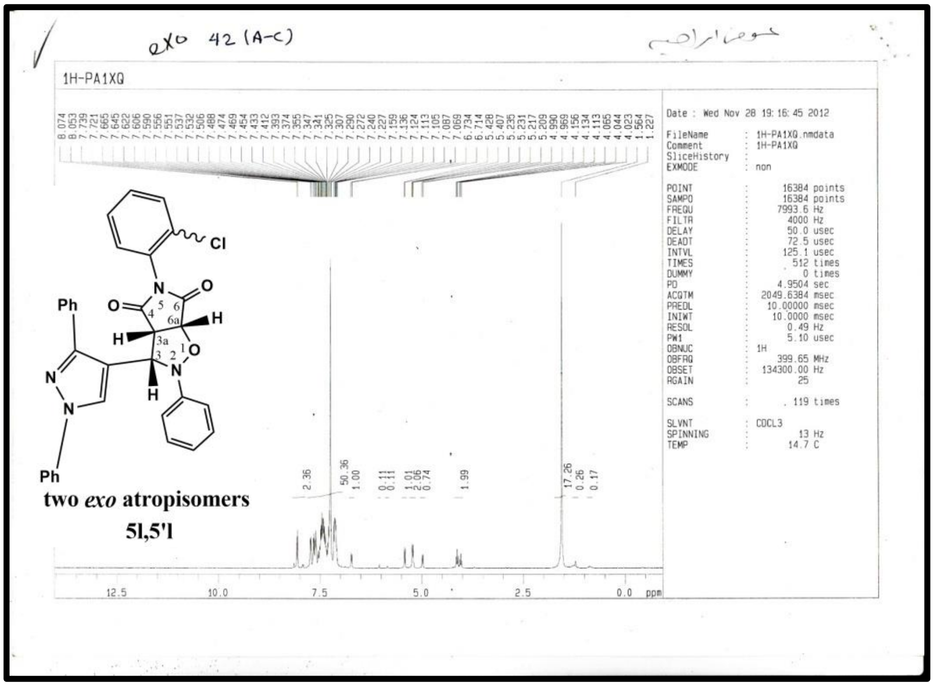

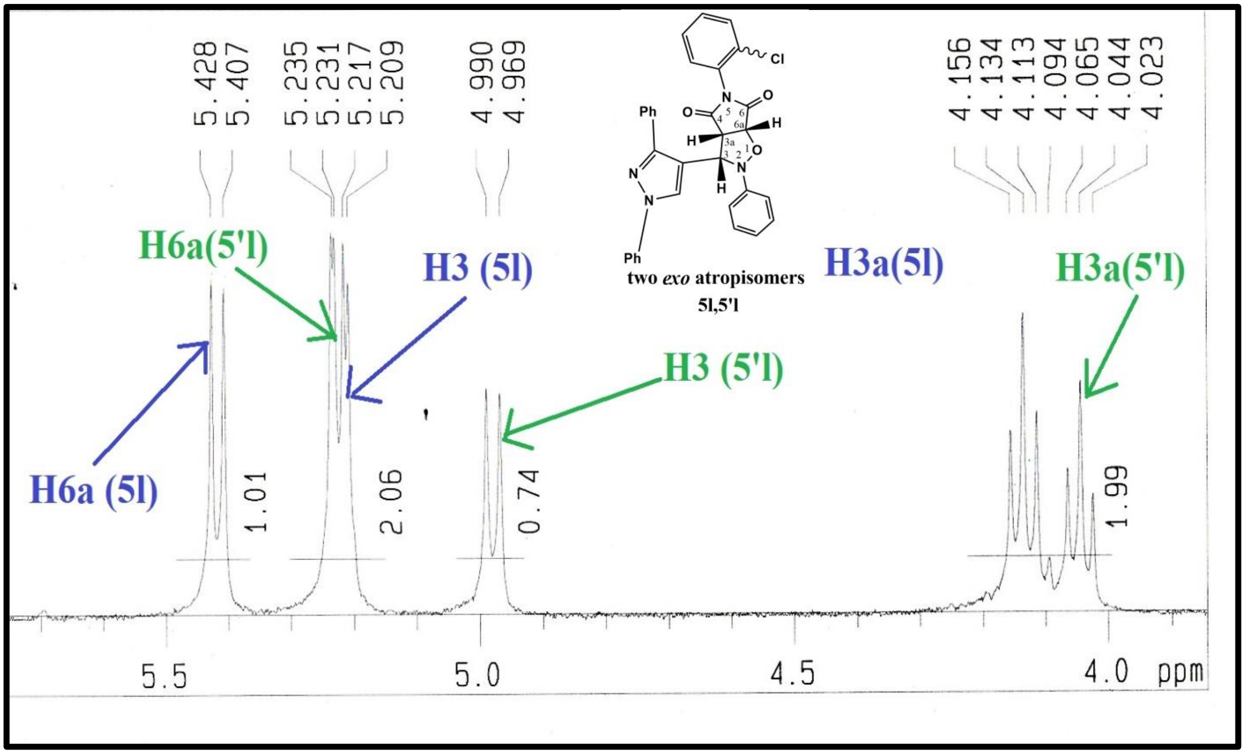

Cycloaddition with N-(4-methoxyphenyl) maleimide (3f)

Formation of 3-(1,3-diphenyl-1H-pyrazol-4-yl)-5-(4-methoxyphenyl)-2-phenyldihydro-2H-pyrrolo[3,4-d]isoxazole-4,6(5H,6aH)-dione $C_{33}H_{26}N_4O_4$

Reaction mixture **(4f,5f)**: ^{1}H-NMR spectrum: δ(400 MHz, $CDCl_3$) 3.72(s, 3H, OCH_3(*endo*)), 3.78(s, 3H, OCH_3(*exo*)), 4.08(d, *J* 7.2 Hz, 1H, H3a(*endo*)), 4.37(t, *J* 8.4 Hz, 1H, H3a(*exo*)), 4.89(d, *J* 9.6 Hz, 1H, H3(*exo*)), 5.44(d, J 7.2 Hz, 1H, H6a(*endo*)), 5.6(dd, *J* 8.8; 5.2Hz, 1H, H6a(*exo*)), 6(s, 1H, H3(*endo*)), 6.4-8.6(m, 40H, Ar H), 9.86(s, 1H, Nitrone C<u>H</u>=N).

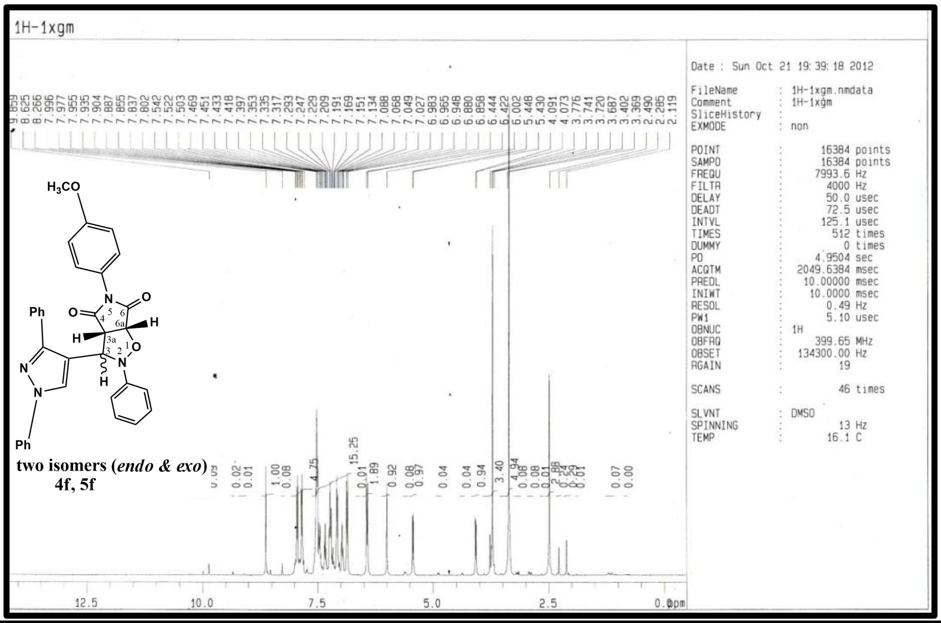

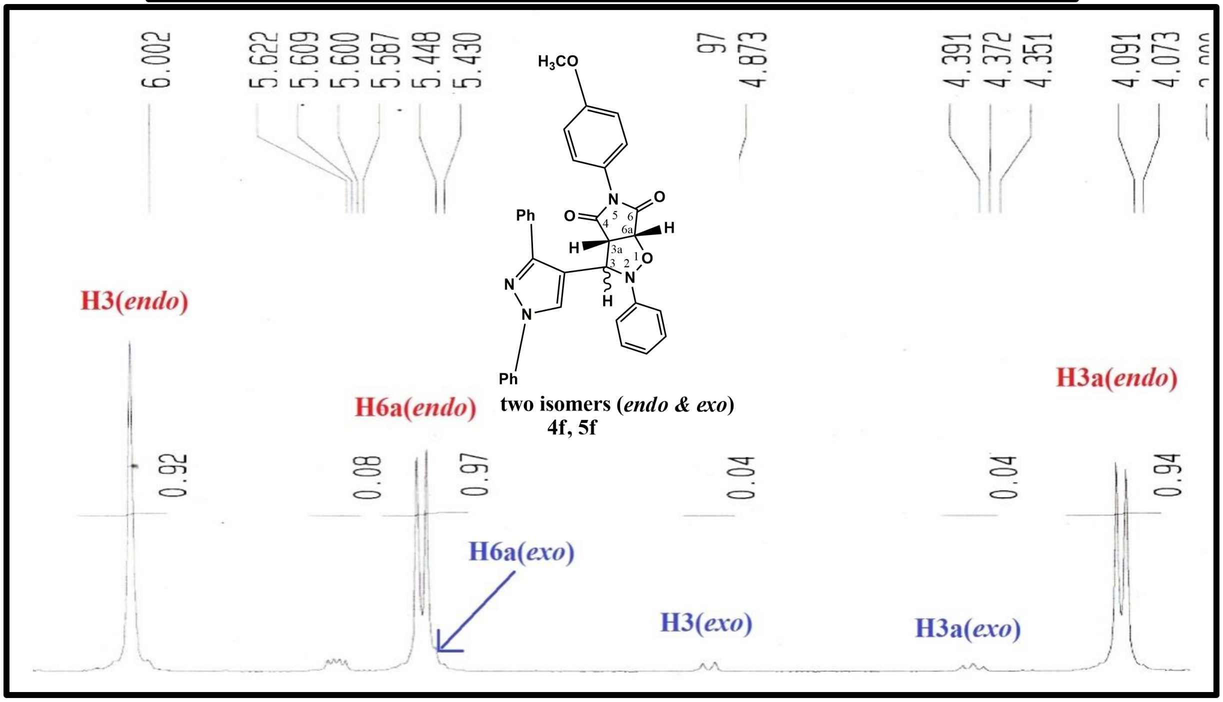

Assiut Univercity Central Lab

11/26/12 8:46:02 PM Page 1

File: 1XG Date Run: 11-21-2012 Time Run: 20:56:12
Sample: Dr/ Awad
Instrument: JEOL JMS600 Run By: Souzan
Inlet: My Inlet Ionization mode: EI+ Printed by: Souzan

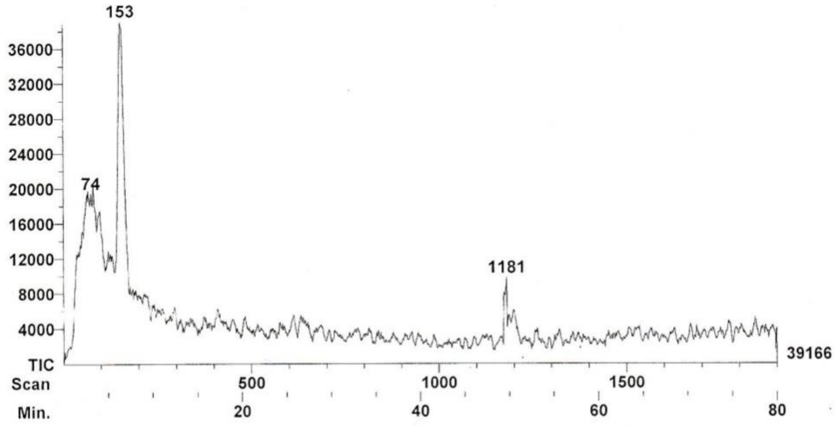

RIC : TIC
Scan range : 78 - 89
Baseline at : 24-34

Total area : 199350
Background : 70257
Peak area : 129093

RIC : TIC
Scan range : 136 - 176 (Scan 153)
Baseline at : 203-214

Total area : 889363
Background : 296960
Peak area : 592403

RIC : TIC
Scan range : 1172 - 1182 (Scan 1181)
Baseline at : 1144-1153

Total area : 78437
Background : 17970
Peak area : 60467

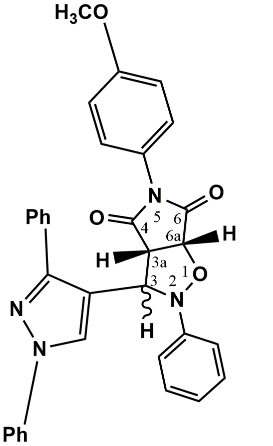

two isomers (*endo & exo*)
4f, 5f

Assiut Univercity Central Lab

11/26/12 8:49:28 PM Page 1

File: 1XG
Sample: Dr/ Awad
Instrument: JEOL JMS600
Inlet: My Inlet

Date Run: 11-21-2012
Ionization mode: EI+

Time Run: 20:56:12
Run By: Souzan
Printed by: Souzan

Scan: 4-111
Base: m/z 186; .1%FS TIC: 12277

R.T.: 2:24.889

#Ions: 527

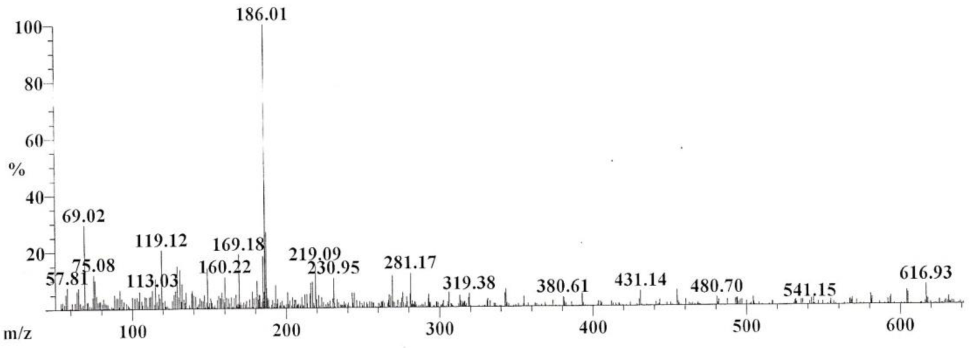

Assiut Univercity Central Lab

11/26/12 8:52:04 PM Page 1

File: 1XG
Sample: Dr/ Awad
Instrument: JEOL JMS600
Inlet: My Inlet

Date Run: 11-21-2012
Ionization mode: EI+

Time Run: 20:56:12
Run By: Souzan
Printed by: Souzan

Scan: 111-230
Base: m/z 80; .2%FS TIC: 13397

R.T.: 7:10.417

#Ions: 494

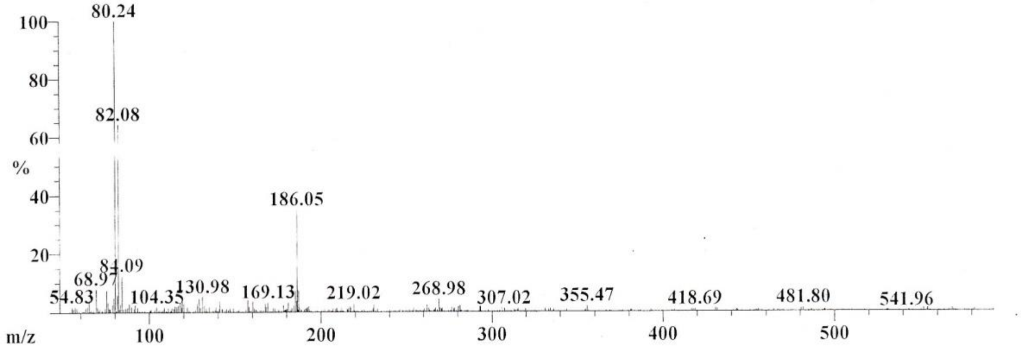

Endo-isomer **(4f)**: (1 g, 60 %); white crystals; mp: 196-198°C. FTIR (KBr) (cm^{-1}): 3100(Ar. C-H), 2900(Aliph. C-H), 1720(C=O). ^{1}H-NMR spectrum: δ ppm(400 MHz, DMSO-d_6) 3.76(s, 3H, OCH_3), 3.98(d, *J* 7.32 Hz, 1H, H3a), 5.09(d, *J* 7.36 Hz, 1H, H6a), 6.03(s, 1H, H3), 6.49-8.15 (m, 20H, Ar H). ^{13}C{H}NMR spectrum: δ ppm(100.53 MHz, $CDCl_3$) 25(OCH_3); 55.5, 55.9, 63.3(3 Aliphatic **C**); 114.4(2), 114.7(2), 119.1(2), 119.4, 123.2, 123.5, 126.8, 127.4(3), 128.3(2), 128.6, 129.1(2), 129.5(4), 132.3, 139.8, 148.3, 150.3, 159.8 (27 Aromatic **C**); 172.91 and 174.01 (2 **C**=O). Anal. Calcd for ($C_{33}H_{26}N_4O_4$) (%): C, 73.05; H, 4.83; N, 10.33. Found; C, 73.40; H, 4.85; N, 10.35.

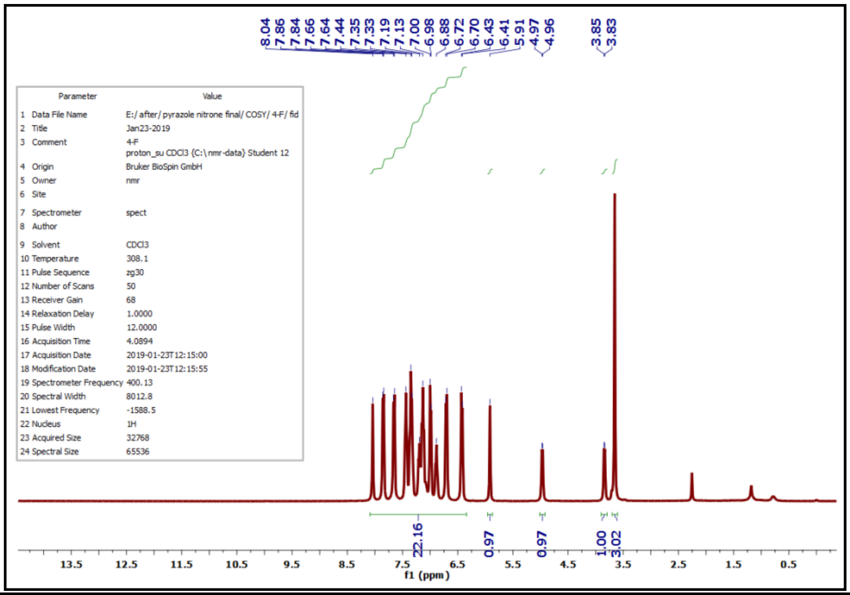

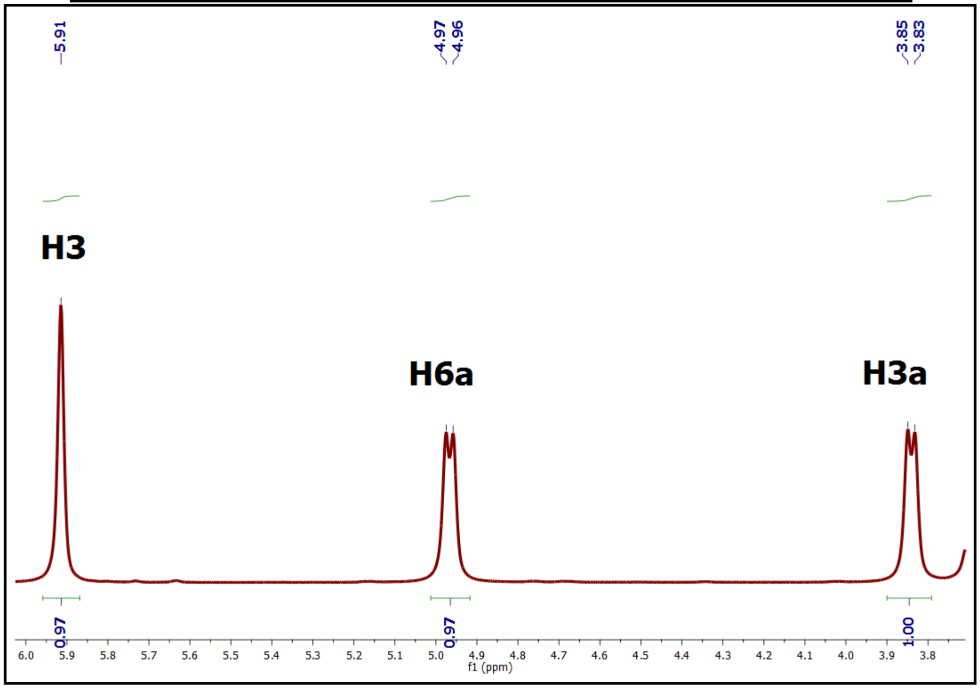

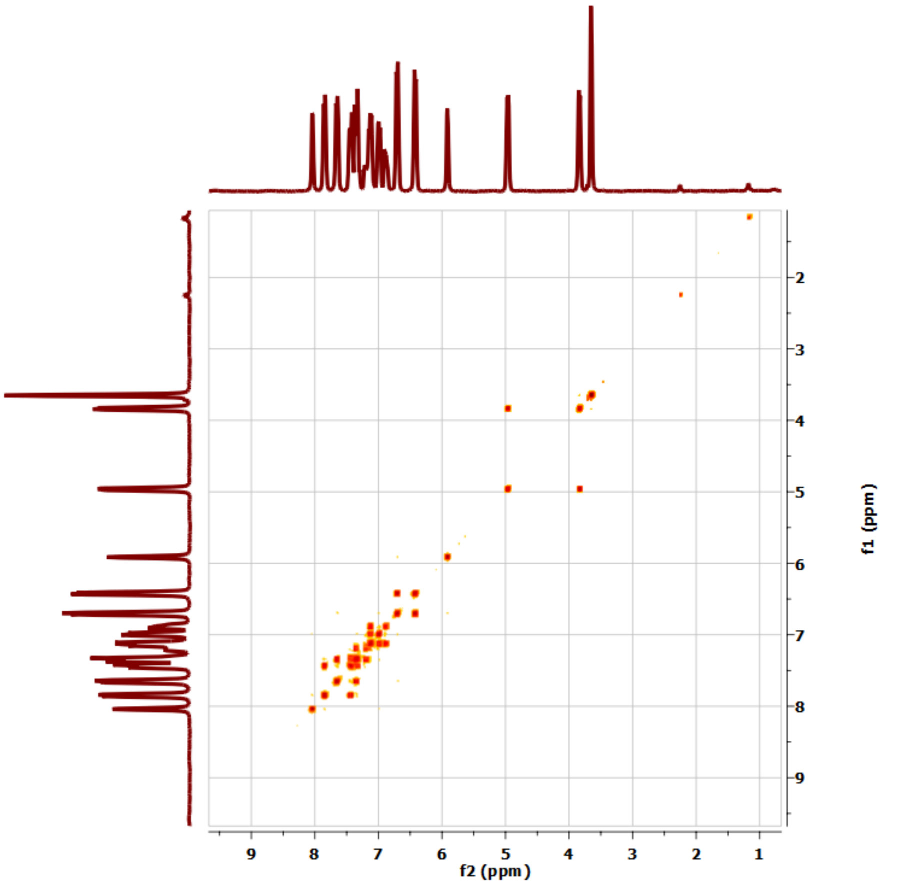

Parameter	Value (f2, f1)
1 Data File Name	E:/ after/ pyrazole nitrone final/ COSY/ 4-f-cosy/ pdata/ 1/ 2rr
2 Title	Jan23-2019
3 Comment	4-F cosy_su CDCl3 {C:\nmr-data} Student 12
4 Origin	Bruker BioSpin GmbH
5 Owner	nmr
6 Site	
7 Spectrometer	spect
8 Author	
9 Solvent	CDCl3
10 Temperature	308.2
11 Pulse Sequence	cosygpppqf
12 Number of Scans	3
13 Receiver Gain	40
14 Relaxation Delay	0.9295
15 Pulse Width	12.0000
16 Acquisition Time	0.2621
17 Acquisition Date	2019-01-23T12:16:00
18 Modification Date	2019-01-23T12:24:59
19 Spectrometer Frequency	(400.13, 400.13)
20 Spectral Width	(3906.2, 3906.2)
21 Lowest Frequency	(-27.5, -27.5)
22 Nucleus	(1H, 1H)
23 Acquired Size	(1024, 128)
24 Spectral Size	(2048, 1024)

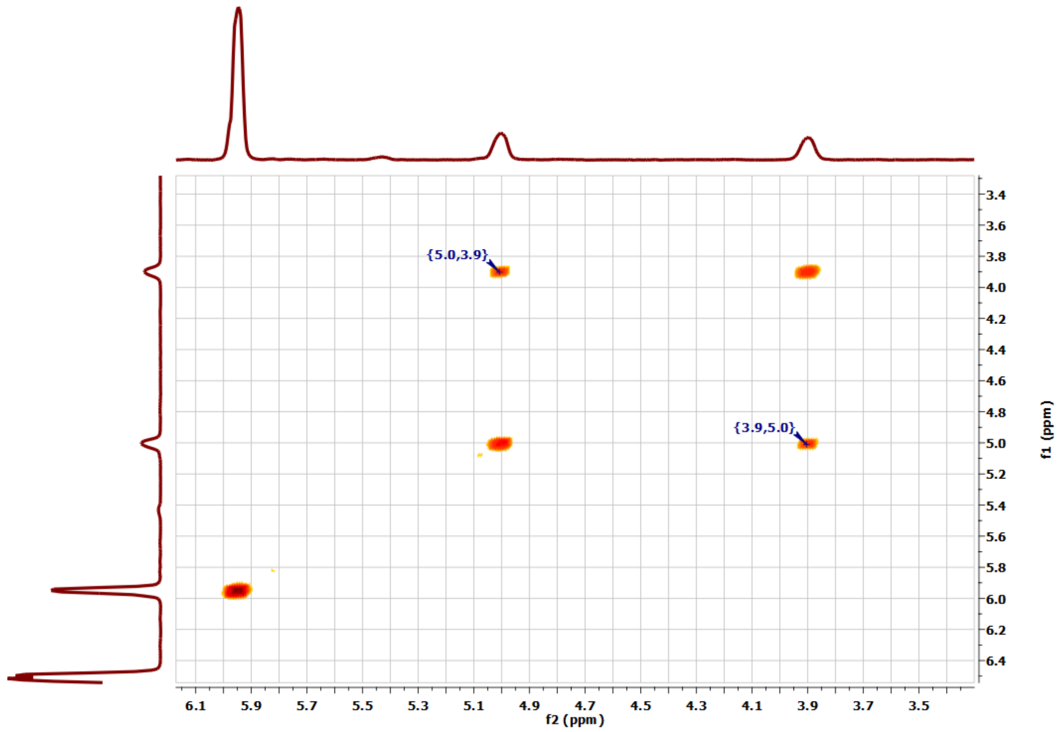

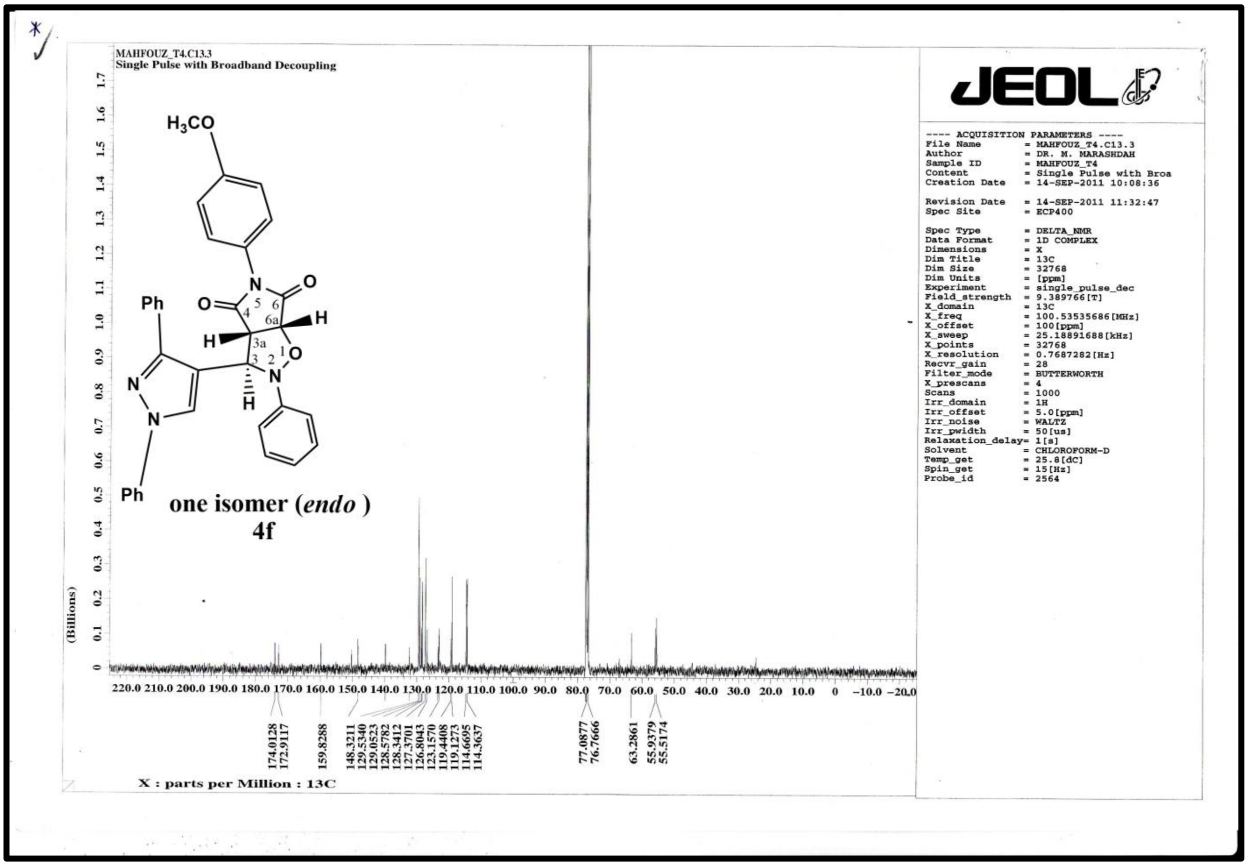
MAHFOUZ_T4.C13.3
Single Pulse with Broadband Decoupling
JEOL
---- ACQUISITION PARAMETERS ----
File Name = MAHFOUZ_T4.C13.3
Author = DR. M. MARASHDAH
Sample ID = MAHFOUZ_T4
Content = Single Pulse with Broa
Creation Date = 14-SEP-2011 10:08:36
Revision Date = 14-SEP-2011 11:32:47
Spec Site = ECP400
Spec Type = DELTA_NMR
Data Format = 1D COMPLEX
Dimensions = X
Dim Title = 13C
Dim Size = 32768
Dim Units = [ppm]
Experiment = single_pulse_dec
Field_strength = 9.389766[T]
X_domain = 13C
X_freq = 100.53535686[MHz]
X_offset = 100[ppm]
X_sweep = 25.18891688[kHz]
X_points = 32768
X_resolution = 0.7687282[Hz]
Recvr_gain = 28
Filter_mode = BUTTERWORTH
X_prescans = 4
Scans = 1000
Irr_domain = 1H
Irr_offset = 5.0[ppm]
Irr_noise = WALTZ
Irr_pwidth = 50[us]
Relaxation_delay= 1[s]
Solvent = CHLOROFORM-D
Temp_get = 25.8[dC]
Spin_get = 15[Hz]
Probe_id = 2564
H_3CO
Ph
Ph
one isomer (*endo*)
4f
(Billions)
174.0128
172.9117
159.8288
148.3211
129.5340
129.0523
128.5782
128.3412
127.3701
126.8043
123.1570
119.4408
119.1273
114.6695
114.3637
77.0877
76.7666
63.2861
55.9379
55.5174
X : parts per Million : 13C

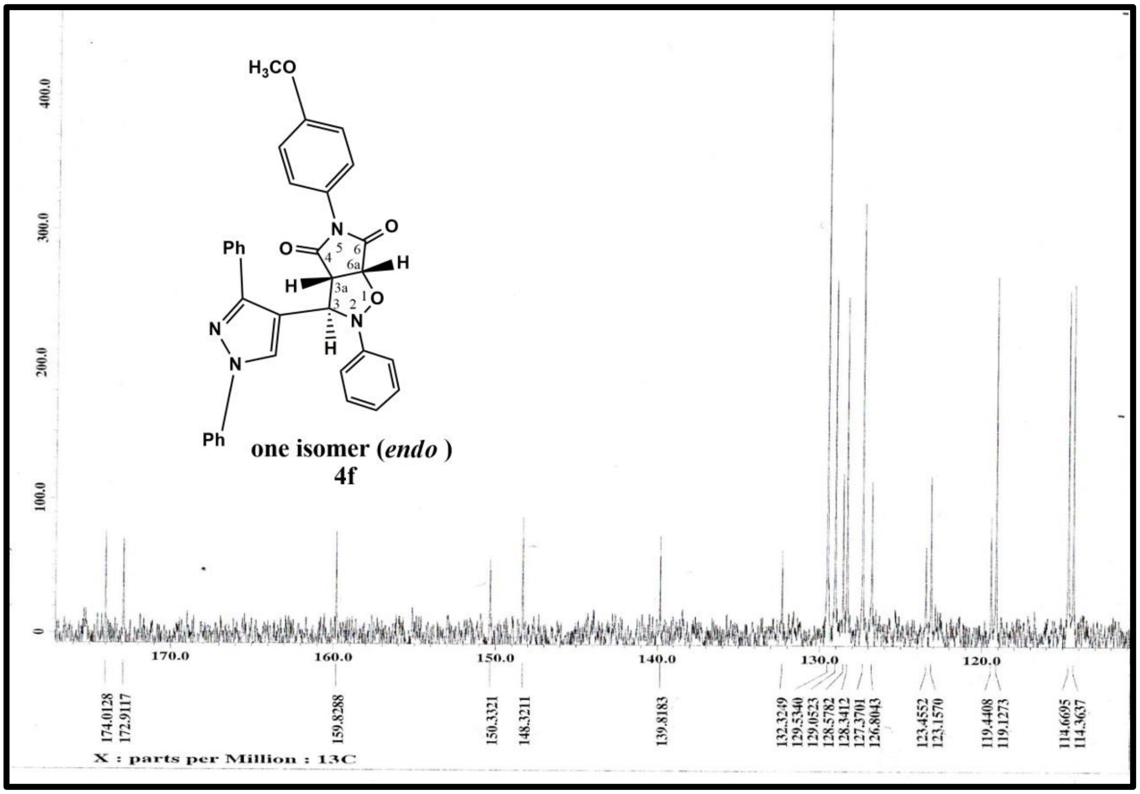
H_3CO
Ph
Ph
one isomer (*endo*)
4f
174.0128
172.9117
159.8288
150.3321
148.3211
139.8183
132.3249
129.5340
129.0523
128.5782
128.3412
127.3701
126.8043
123.4552
123.1570
119.4408
119.1273
114.6695
114.3637
X : parts per Million : 13C

Exo-isomer **(5f)**: (0.006 g, 0.36 %); white crystals; m.p.; 182-183°C. ^{1}H-NMR: δ ppm (400 MHz, DMSO-d$_6$) 3.74(s, 3H, OCH_3), 4.04(t, *J* 8.4 Hz, 1H, H3a), 4.13(d, *J* 8.8 Hz, 1H, H3), 5.3(d, *J* 8.4 Hz, 1H, H6a), 6.6-7.9(m, 20H, Ar H). Anal. Calcd for ($C_{33}H_{26}N_4O_4$) (%): C, 73.05; H, 4.83; N, 10.33. Found; C, 72.94;H, 5.02; N, 10.25.

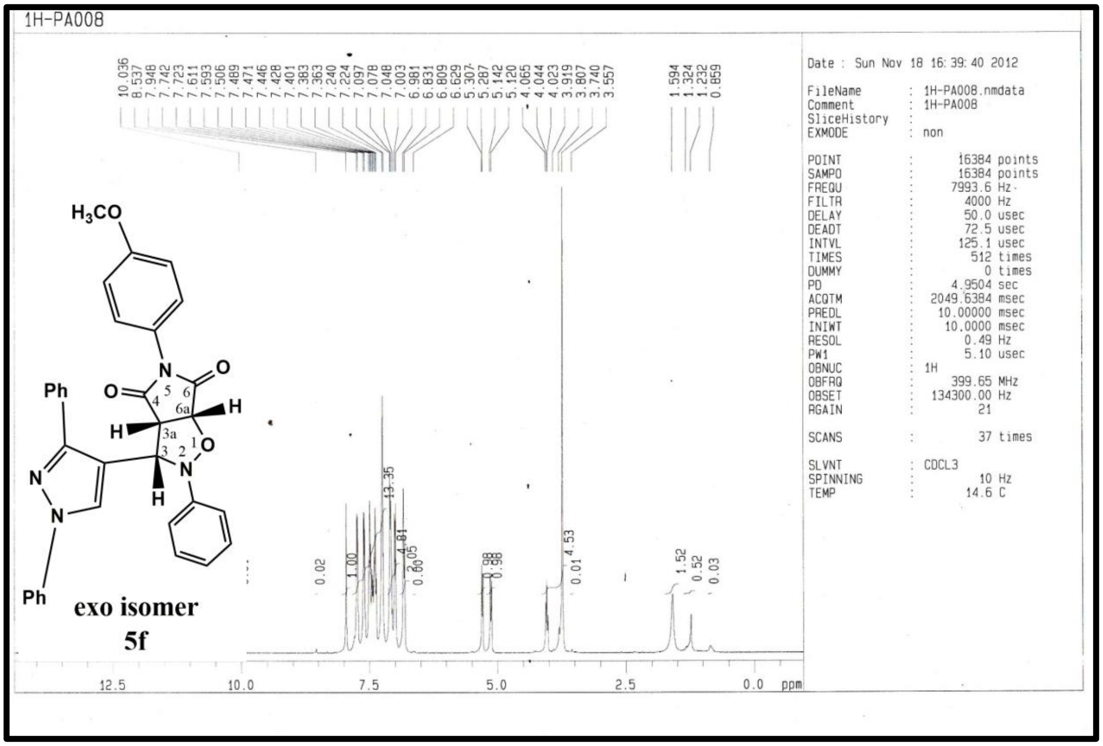

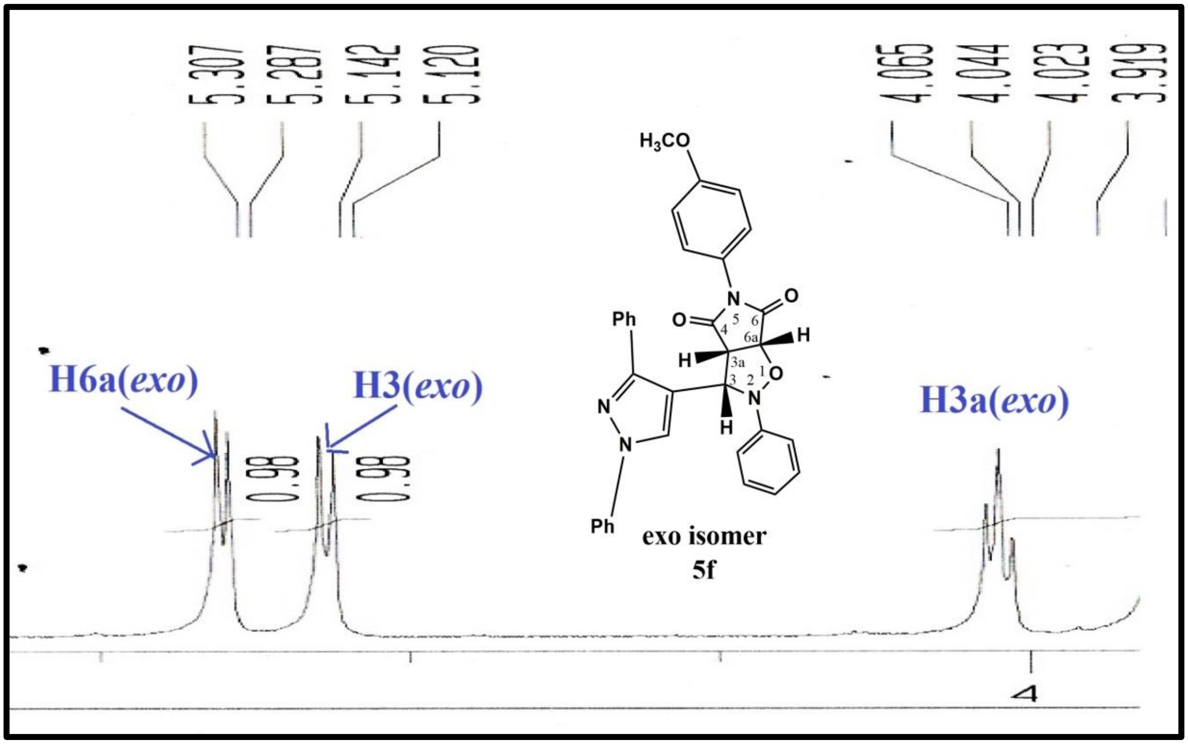

Cycloaddition with N-(2-methoxyphenyl) maleimide (3m)

Formation of 3-(1,3-diphenyl-1H-pyrazol-4-yl)-5-(2-methoxyphenyl)-2-phenyldihydro-2H-pyrrolo[3,4-d]isoxazole-4,6(5H,6aH)-dione $C_{33}H_{26}N_4O_4$.

Reaction mixture **(4m,4'm, 5m, 5'm)**:^{1}H-NMR spectrum: δ ppm (400 MHz, $CDCl_3$) 3.49(s, 3H, OCH_3(**4'm**)), 3.68(s, 3H, OCH_3(**4m**)), 3.72(s, 3H, OCH_3(**5'm**)), 3.78(s, 3H, OCH_3(**5m**)), 4.13(d, *J* 7.6Hz, 1H, H3a(**4'm**)), 4.36(t, *J* 8Hz, 1H, H3a(**5m**)), 4.43(d, *J* 8 Hz, 1H, H3a(**4m**)), 4.52(t, *J* 8.4Hz, 1H, H3a(**5'm**)), 4.79(d, *J* 8.8 Hz, 1H, H3 (**5m**)), 4.87(d, *J* 9.2 Hz, 1H, H3 (**5'm**)), 5.37(d, *J* 7.6 Hz, 1H, H6a(**5m**)), 5.45(s, 1H, H3(**4'm**)), 5.53(d, *J* 7.6 Hz, 1H, H6'(**4m**)), 5.62(d, *J* 7.2 Hz, 1H, H6a(**4m**)), 5.65(d, *J* 8Hz, 1H, H6a(**5'm**)), 6.05(s, 1H, H3(**4m**)), 6.7-8.7(m, 80H, ArH), 9.98(s, 1H, Nitrone C<u>H</u>=N).

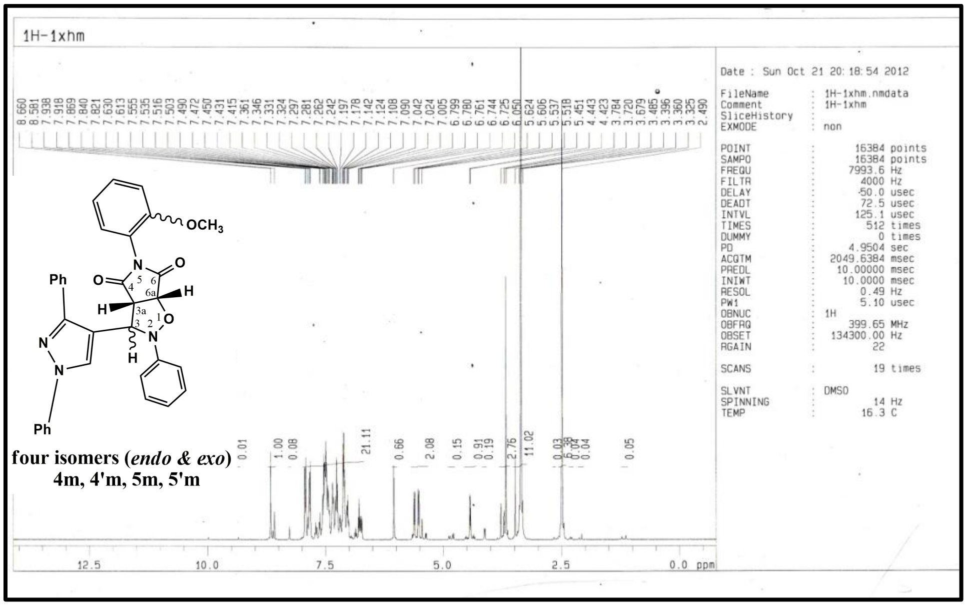

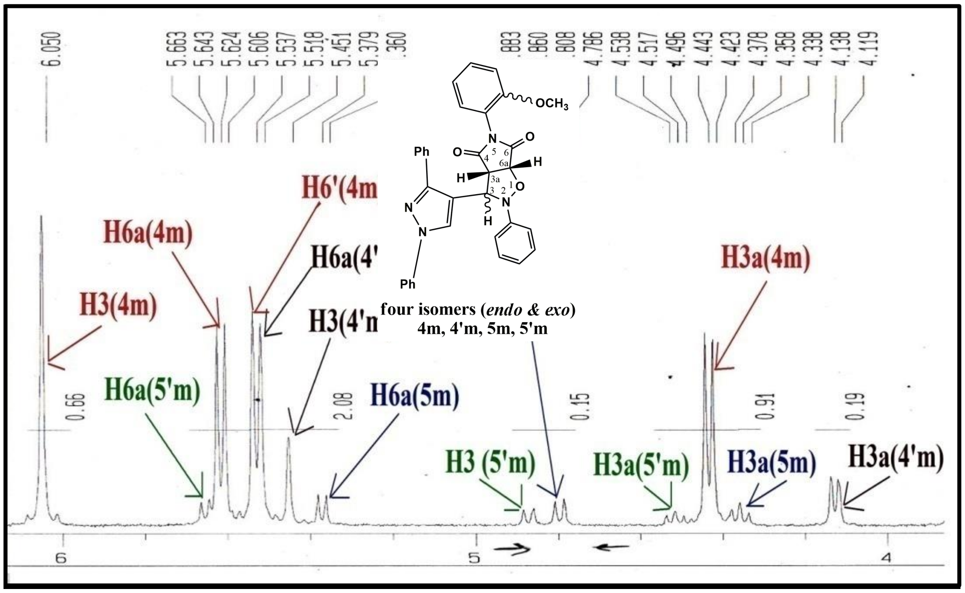

Assiut Univercity Central Lab

11/26/12 11:19:04 PM Page 1

File: CLEAN4 Date Run: 11-17-2012 Time Run: 21:36:18
Sample: Dr/ Awad
Instrument: JEOL JMS600 Run By: Souzan
Inlet: My Inlet Ionization mode: EI+ Printed by: Souzan

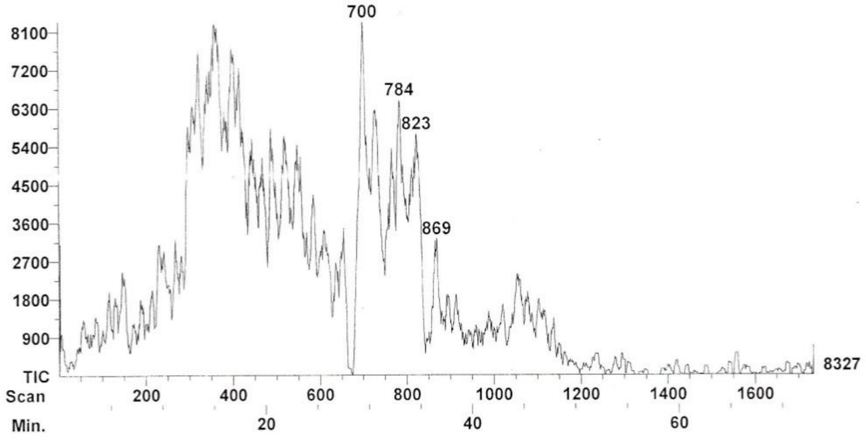

RIC : TIC
Scan range : 676 - 714 (Scan 700)
Baseline at : 669-676

Total area : 176553
Background : 3382
Peak area : 173171

RIC : TIC
Scan range : 775 - 789 (Scan 784)
Baseline at : 756-761

Total area : 75923
Background : 51128
Peak area : 24795

RIC : TIC
Scan range : 819 - 842 (Scan 823)
Baseline at : 842-843

Total area : 84270
Background : 13662
Peak area : 70608

RIC : TIC
Scan range : 853 - 878 (Scan 869)
Baseline at : 840-848

Total area : 50815
Background : 19050
Peak area : 31765

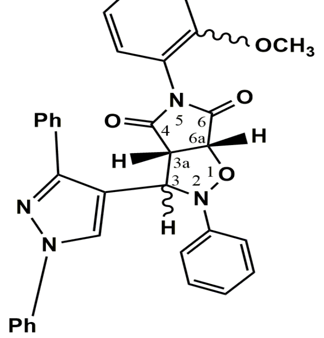

four isomers (*endo & exo*)
4m, 4'm, 5m, 5'm

Assiut Univercity Central Lab

11/26/12 11:26:04 PM Page 1

File: CLEAN4 Date Run: 11-17-2012 Time Run: 21:36:18
Sample: Dr/ Awad
Instrument: JEOL JMS600 Run By: Souzan
Inlet: My Inlet Ionization mode: EI+ Printed by: Souzan

Scan: 676-748 R.T.: 29:58.68
Base: m/z 268; 0%FS TIC: 4646 #Ions: 314

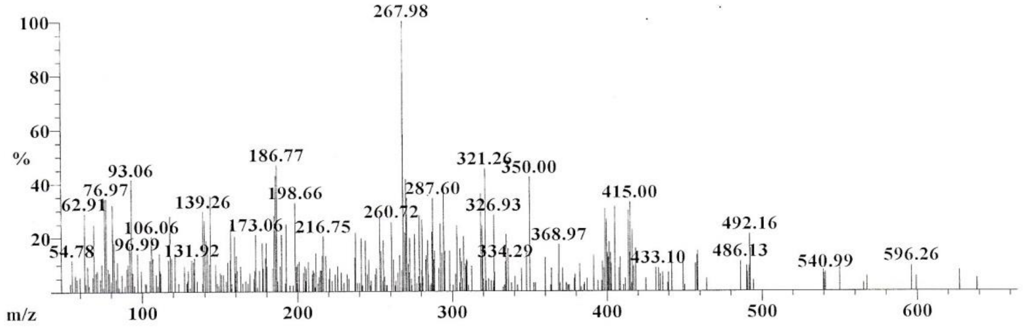

Assiut Univercity Central Lab

11/26/12 11:32:13 PM Page 1

File: CLEAN4 Date Run: 11-17-2012 Time Run: 21:36:18
Sample: Dr/ Awad
Instrument: JEOL JMS600 Run By: Souzan
Inlet: My Inlet Ionization mode: EI+ Printed by: Souzan

Scan: 748-805 R.T.: 32:41.65
Base: m/z 270; 0%FS TIC: 4336 #Ions: 260

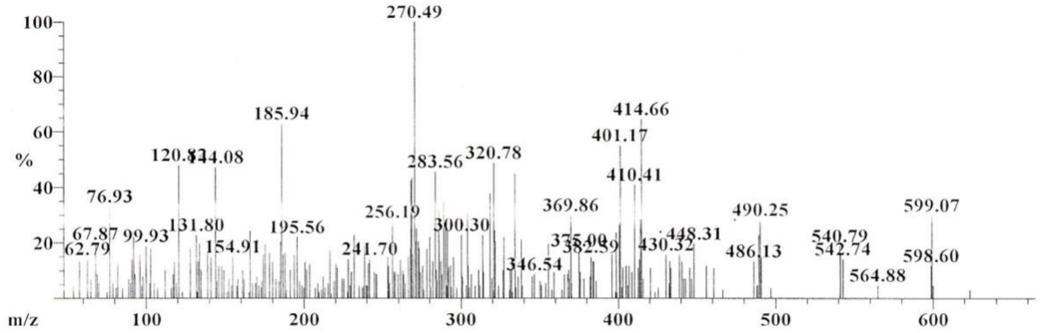

Assiut Univercity Central Lab

11/26/12 11:34:11 PM Page 1

File: CLEAN4 Date Run: 11-17-2012 Time Run: 21:36:18
Sample: Dr/ Awad
Instrument: JEOL JMS600 Run By: Souzan
Inlet: My Inlet Ionization mode: EI+ Printed by: Souzan

Scan: 805-847 R.T.: 34:46.74
Base: m/z 270; 0%FS TIC: 3582 #Ions: 180

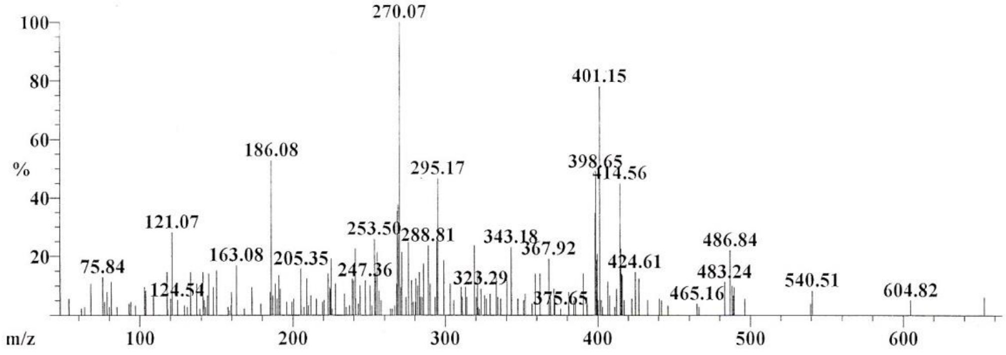

Assiut Univercity Central Lab

11/26/12 11:37:46 PM Page 1

File: CLEAN4 Date Run: 11-17-2012 Time Run: 21:36:18
Sample: Dr/ Awad
Instrument: JEOL JMS600 Run By: Souzan
Inlet: My Inlet Ionization mode: EI+ Printed by: Souzan

Scan: 847-935 R.T.: 37:30.95
Base: m/z 270; 0%FS TIC: 1497 #Ions: 168

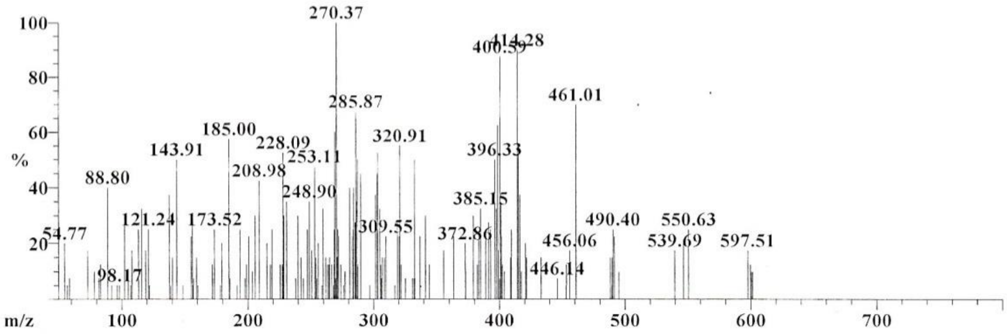

Endo-atropisomers **(4m, 4'm)**: (0.96 g, 55 %); white crystals; mp: 148-150°C. FTIR (KBr) (cm^{-1}): 3030(Ar. C-H), 2968(Aliph. C-H), 1723(C=O). ^{1}H-NMR spectrum: δ ppm(400 MHz, $CDCl_3$) 3.52(s, 3H, OCH_3(**4'm**)), 3.72(s, 3H, OCH_3(**4m**)), 4.04-4.08(m, 2H, H3a(**4m**, **4'm**)), 5.16(d, *J* 8.04Hz, 1H, H6a(**4m**)), 5.21(d, *J* 7.36 Hz, 1H, H6a(**4'm**)), 5.68(s, 1H, H3(**4'm**)), 5.84(d, 1H, H6'(**4m**)), 6.03(s, 1H, H3(**4m**)), 6.8-8.2(m, 39H, Ar-H). The ratio of the isolated *endo*-atropisomers **4m:4'm** is 88:12. Anal. Calcd for ($C_{33}H_{26}N_4O_4$) (%): C, 73.05; H, 4.83; N, 10.33. Found; C, 72.86; H, 4.63; N, 10.18.

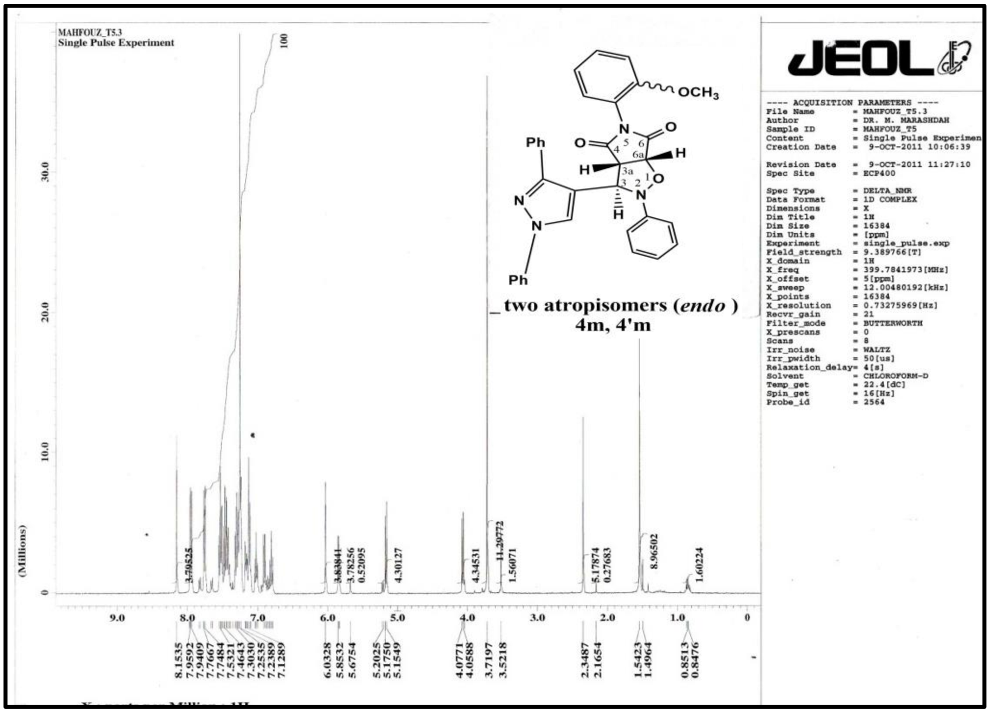

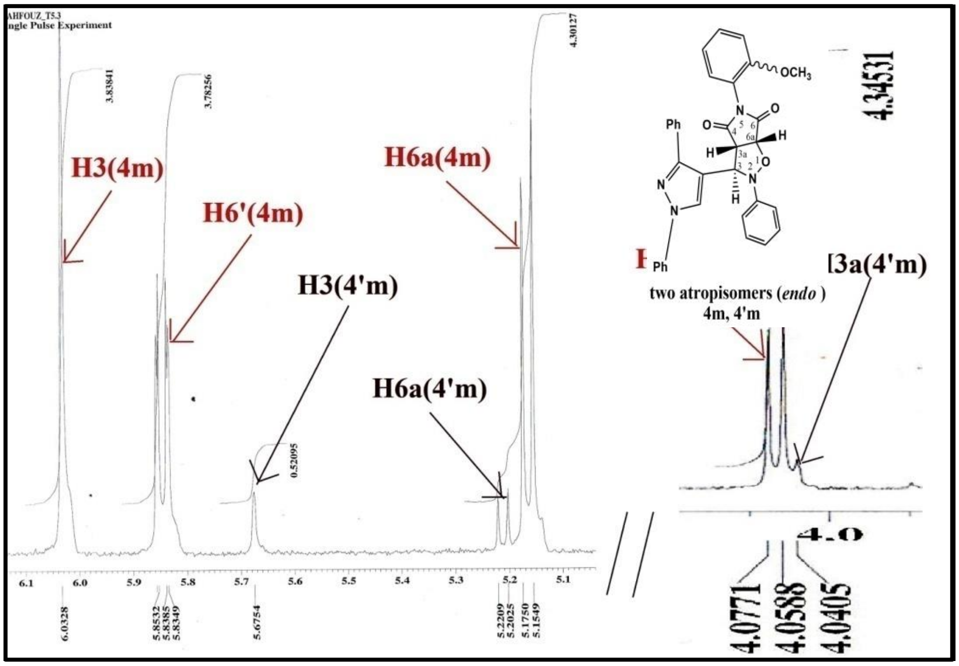

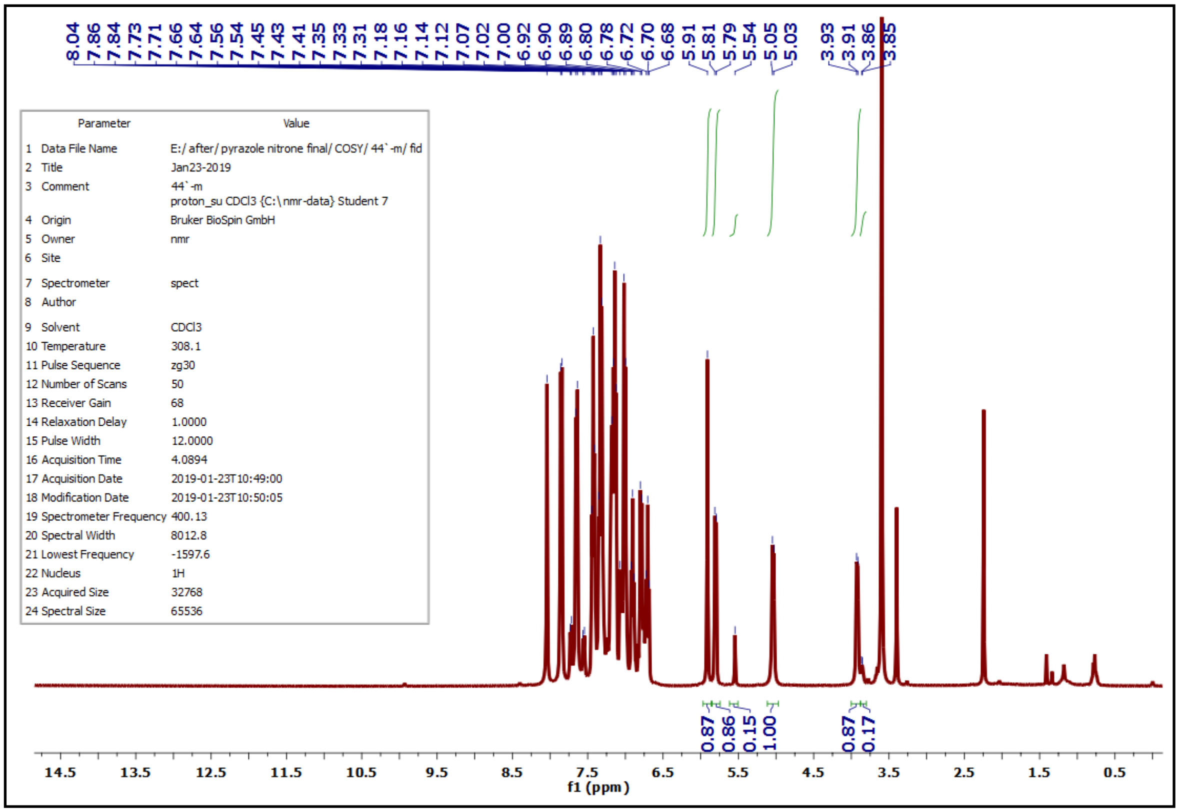

8.04 7.86 7.84 7.73 7.71 7.66 7.64 7.56 7.54 7.45 7.43 7.41 7.35 7.33 7.31 7.18 7.16 7.14 7.12 7.07 7.02 7.00 6.92 6.90 6.89 6.80 6.78 6.72 6.70 6.68 5.91 5.81 5.79 5.54 5.05 5.03 3.93 3.91 3.86 3.85
Parameter Value
1 Data File Name E:/ after/ pyrazole nitrone final/ COSY/ 44`-m/ fid
2 Title Jan23-2019
3 Comment 44`-m proton_su CDCl3 {C:\nmr-data} Student 7
4 Origin Bruker BioSpin GmbH
5 Owner nmr
6 Site
7 Spectrometer spect
8 Author
9 Solvent CDCl3
10 Temperature 308.1
11 Pulse Sequence zg30
12 Number of Scans 50
13 Receiver Gain 68
14 Relaxation Delay 1.0000
15 Pulse Width 12.0000
16 Acquisition Time 4.0894
17 Acquisition Date 2019-01-23T10:49:00
18 Modification Date 2019-01-23T10:50:05
19 Spectrometer Frequency 400.13
20 Spectral Width 8012.8
21 Lowest Frequency -1597.6
22 Nucleus 1H
23 Acquired Size 32768
24 Spectral Size 65536
0.87 0.86 0.15 1.00 0.87 0.17
14.5 13.5 12.5 11.5 10.5 9.5 8.5 7.5 6.5 5.5 4.5 3.5 2.5 1.5 0.5
f1 (ppm)

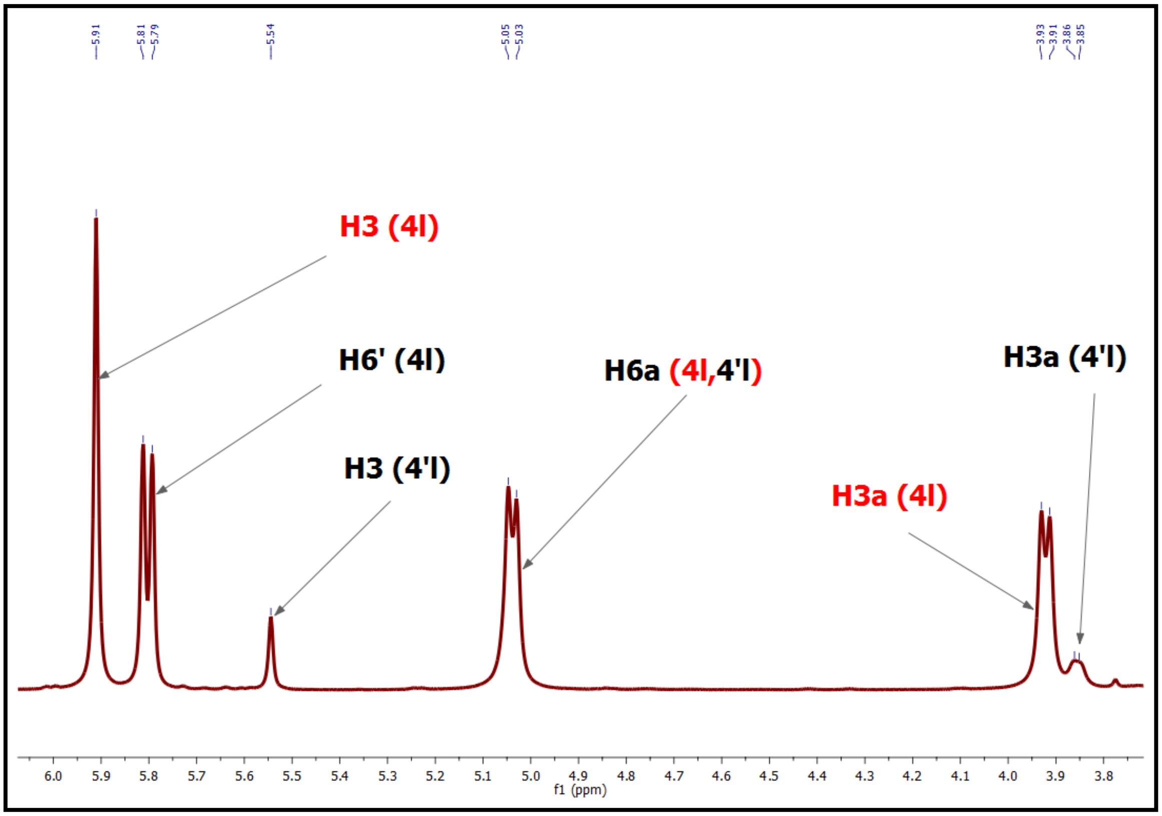

5.91 5.81 5.79 5.54 5.05 5.03 3.93 3.91 3.86 3.85
H3 (4l)
H6' (4l)
H3 (4'l)
H6a (4l,4'l)
H3a (4'l)
H3a (4l)
6.0 5.9 5.8 5.7 5.6 5.5 5.4 5.3 5.2 5.1 5.0 4.9 4.8 4.7 4.6 4.5 4.4 4.3 4.2 4.1 4.0 3.9 3.8
f1 (ppm)

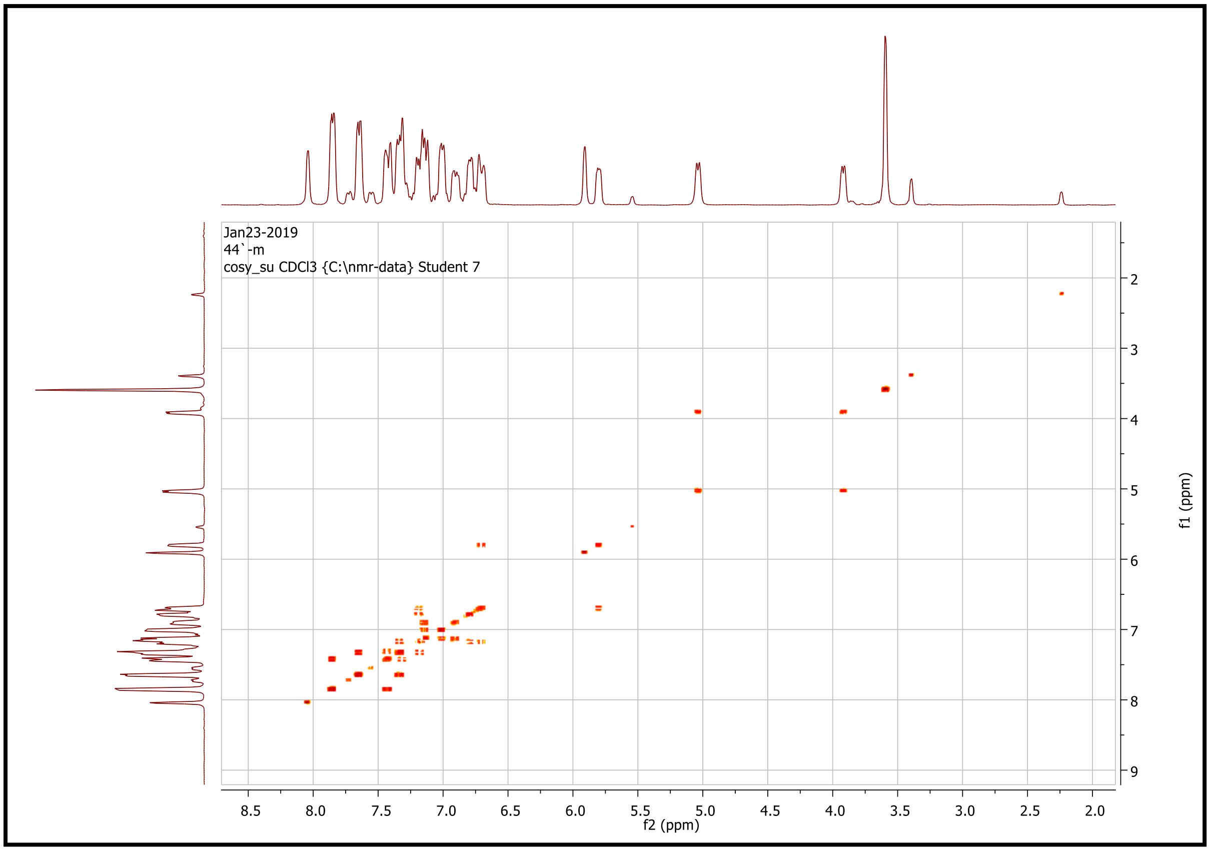
Jan23-2019
44`-m
cosy_su CDCl3 {C:\nmr-data} Student 7
8.5 8.0 7.5 7.0 6.5 6.0 5.5 5.0 4.5 4.0 3.5 3.0 2.5 2.0
f2 (ppm)
2 3 4 5 6 7 8 9
f1 (ppm)

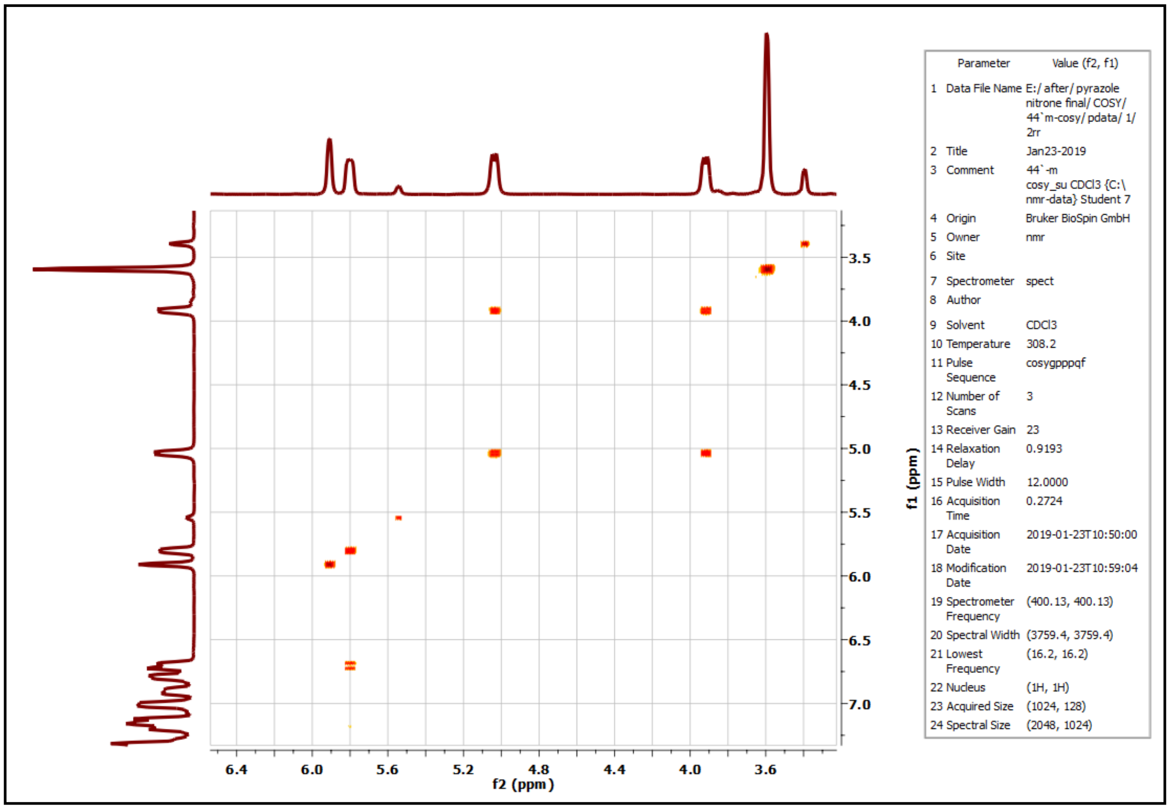
6.4 6.0 5.6 5.2 4.8 4.4 4.0 3.6
f2 (ppm)
3.5 4.0 4.5 5.0 5.5 6.0 6.5 7.0
f1 (ppm)
Parameter Value (f2, f1)
1 Data File Name E:/ after/ pyrazole nitrone final/ COSY/ 44`m-cosy/ pdata/ 1/ 2rr
2 Title Jan23-2019
3 Comment 44`-m cosy_su CDCl3 {C:\ nmr-data} Student 7
4 Origin Bruker BioSpin GmbH
5 Owner nmr
6 Site
7 Spectrometer spect
8 Author
9 Solvent CDCl3
10 Temperature 308.2
11 Pulse Sequence cosygpppqf
12 Number of Scans 3
13 Receiver Gain 23
14 Relaxation Delay 0.9193
15 Pulse Width 12.0000
16 Acquisition Time 0.2724
17 Acquisition Date 2019-01-23T10:50:00
18 Modification Date 2019-01-23T10:59:04
19 Spectrometer Frequency (400.13, 400.13)
20 Spectral Width (3759.4, 3759.4)
21 Lowest Frequency (16.2, 16.2)
22 Nucleus (1H, 1H)
23 Acquired Size (1024, 128)
24 Spectral Size (2048, 1024)

Cycloaddition with N-(4-nitrophenyl) maleimide (3g)

Formation of 3-(1,3-diphenyl-1H-pyrazol-4-yl)-5-(4-nitrophenyl)-2-phenyldihydro-2H-pyrrolo[3,4-d]isoxazole-4,6(5H,6aH)-dione $C_{32}H_{23}N_5O_5$.

Endo-isomer (**4g**): (1.2 g, 68 %); white crystals; mp: 210-212°C. FTIR (KBr) (cm^{-1}): 3030(Ar. C-H), 2950(Aliph. C-H), 1725(C=O). ^{1}H-NMR spectrum: δ ppm (400 MHz, $CDCl_3$) 4.1 (d, *J* 7.2 Hz, 1H, H3a), 5.2(d, *J* 7.2 Hz, 1H, H6a), 6.1(s, 1H, H3), 6.9-8.2(m, 20H, Ar-H). ^{13}C{H}NMR spectrum: δ ppm(100.5 MHz, CDCl3) 55.9, 63.5(3 Aliphatic **C**); 114.7(2), 118.9, 119.1(2), 123.4, 124.2(2), 126.8(2), 126.9,127.3, 128.3(2), 128.6, 129.1(2), 129.5(2), 129.6(2), 132.2, 136.2, 139.7, 147.3, 148.1, 150.2(27 Aromatic **C**); 171.9, 173(2 **C**=O). Anal. Calcd for ($C_{32}H_{23}N_5O_5$) (%): C, 68.93; H, 4.16; N, 12.56. Found; C, 68.91; H, 4.15; N, 12.48.

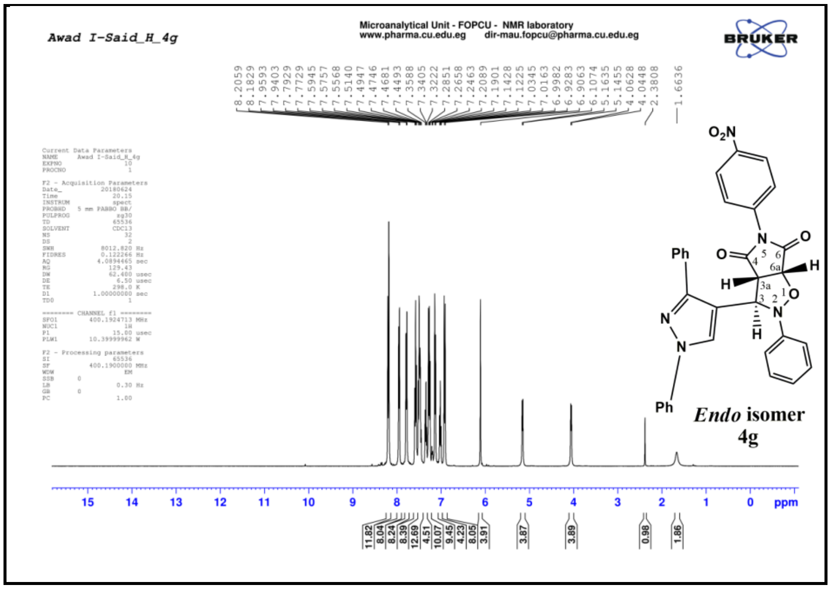

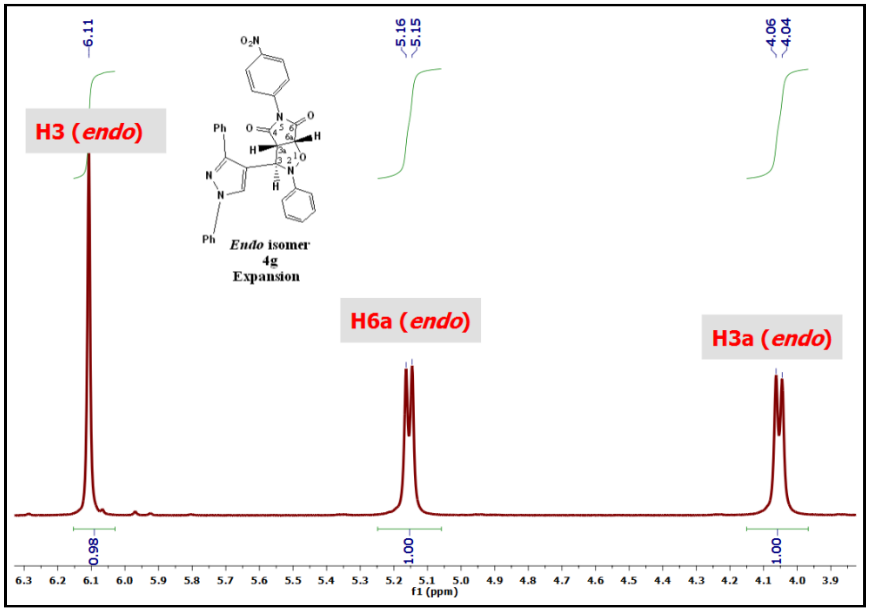

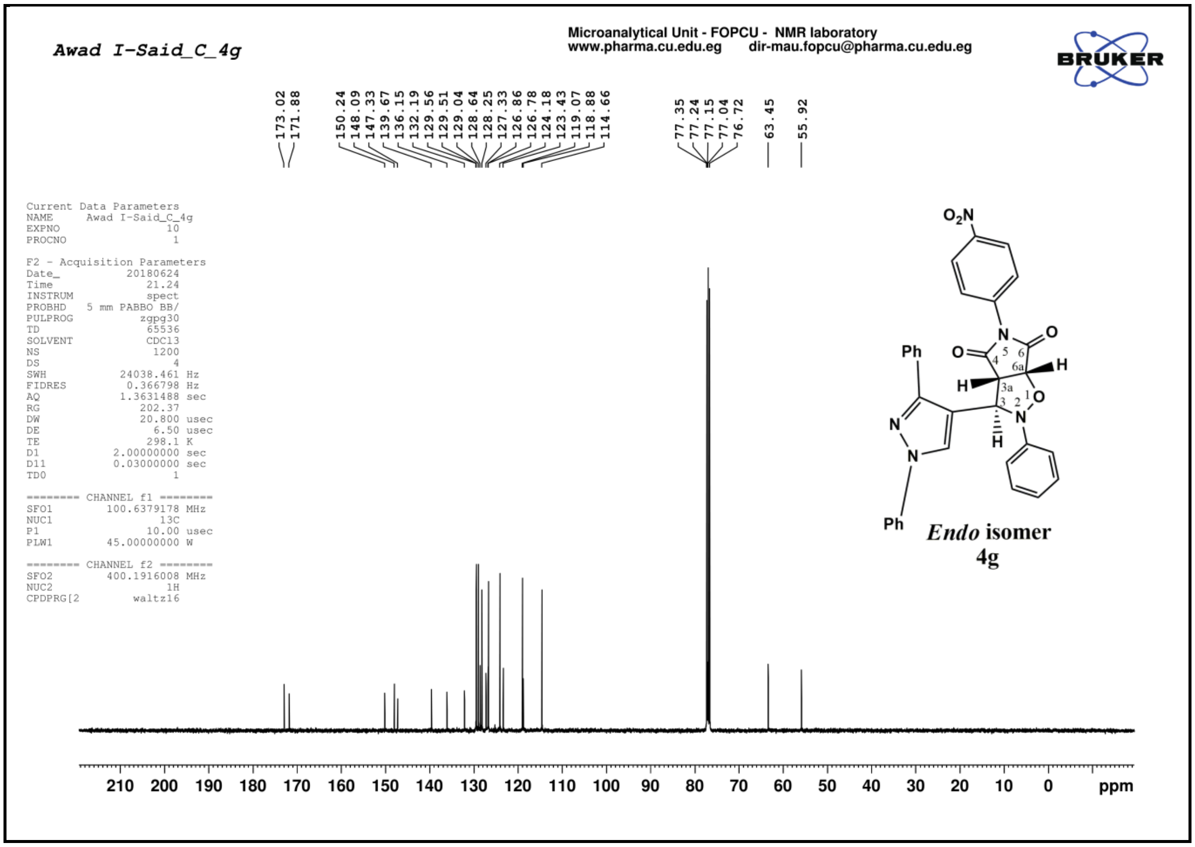
Awad I-Said_C_4g
Microanalytical Unit - FOPCU - NMR laboratory
www.pharma.cu.edu.eg dir-mau.fopcu@pharma.cu.edu.eg
BRUKER
173.02
171.88
150.24
148.09
147.33
139.67
136.15
132.19
129.56
129.51
129.04
128.64
128.25
127.33
126.86
126.78
124.18
123.43
119.07
118.88
114.66
77.35
77.24
77.15
77.04
76.72
63.45
55.92
Current Data Parameters
NAME Awad I-Said_C_4g
EXPNO 10
PROCNO 1
F2 - Acquisition Parameters
Date_ 20180624
Time 21.24
INSTRUM spect
PROBHD 5 mm PABBO BB/
PULPROG zgpg30
TD 65536
SOLVENT CDCl3
NS 1200
DS 4
SWH 24038.461 Hz
FIDRES 0.366798 Hz
AQ 1.3631488 sec
RG 202.37
DW 20.800 usec
DE 6.50 usec
TE 298.1 K
D1 2.00000000 sec
D11 0.03000000 sec
TD0 1
======== CHANNEL f1 ========
SFO1 100.6379178 MHz
NUC1 13C
P1 10.00 usec
PLW1 45.00000000 W
======== CHANNEL f2 ========
SFO2 400.1916008 MHz
NUC2 1H
CPDPRG[2 waltz16
O2N
Ph
O
N
O
4 5 6
6a
H
H
3a
3 2 1 O
N
N
N
H
Ph
Endo isomer
4g
210 200 190 180 170 160 150 140 130 120 110 100 90 80 70 60 50 40 30 20 10 0 ppm

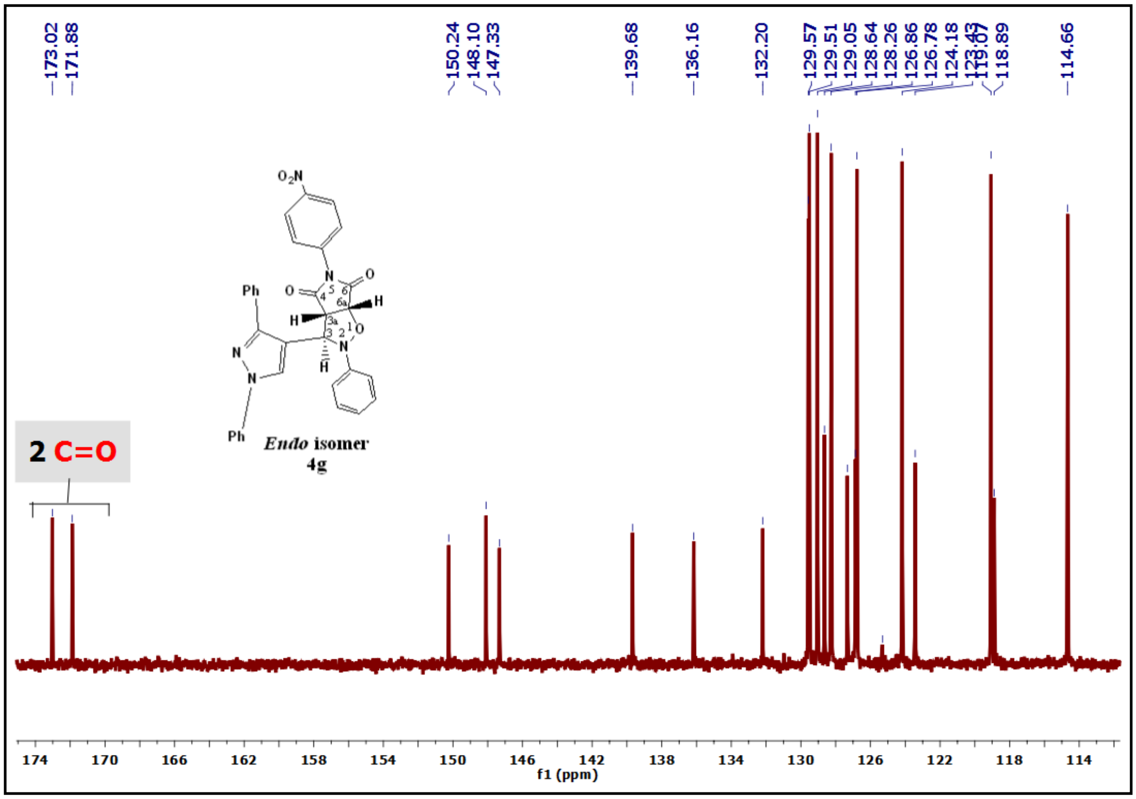
173.02
171.88
150.24
148.10
147.33
139.68
136.16
132.20
129.57
129.51
129.05
128.64
128.26
126.86
126.78
124.18
123.43
119.07
118.89
114.66
O2N
Ph
O
N
O
H
H
N
N
H
Ph
Endo isomer
4g
2 C=O
174 170 166 162 158 154 150 146 142 138 134 130 126 122 118 114
f1 (ppm)

Cycloaddition with N-(1-naphthyl) maleimide (3n)

Formation of 3-(1,3-diphenyl-1H-pyrazol-4-yl)-5-(naphthalen-1-yl)-2-phenyldihydro-2H-pyrrolo[3,4-d]isoxazole-4,6(5H,6aH)-dione $C_{36}H_{26}N_4O_3$.

Reaction Reaction mixture **(4n,4'n,5n,5'n)**: ^{1}H-NMR spectrum: δ ppm (400 MHz, $CDCl_3$) 4.22(d, *J* 7.6Hz, 1H, H3a(**4'n**)), 4.35(d, *J* 7.2Hz, 1H, H3a(**4n**)), 4.45(t, *J* 11.6Hz, 1H, H3a(**5'n**)), 4.54(t, *J* 12Hz, 1H, H3a(**5n**)), 4.99(d, *J* 8.8Hz, 1H, H3(**5'n**)), 5.07(d, *J* 8.4Hz, 1H, H3(**5n**)), 5.47(d, *J* 7.2Hz, 1H, H6a(**5n**)), 5.602(d, *J* 7.6 Hz, 1H, H6a(**4n**)), 5.62(d, *J* 5.6 Hz, 1H, H6'(**4n**)), 5.63(d, *J* 6.4 Hz, 1H, H6a(**4'n**)), 5.72(d, *J* 7.2 Hz, 1H, H6a (**5'n**)), 5.87(s, 1H, H3(**4'n**)), 6.14(s, 1H, H3(**4n**)), 6.6-8.7(m, 92H, Ar H), 9.98(s, 1H, Nitrone CH=N).

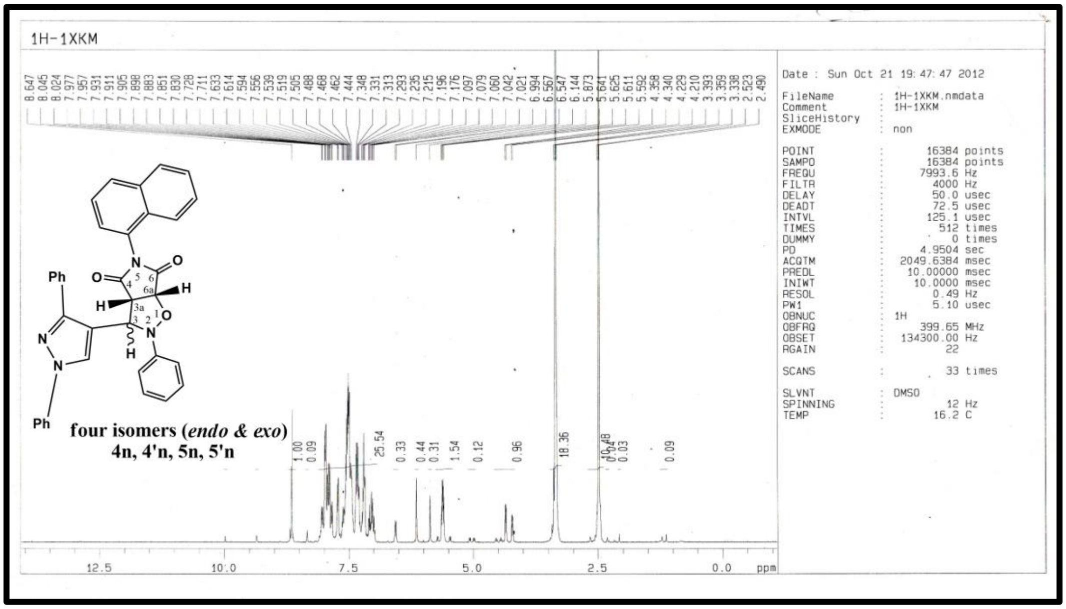

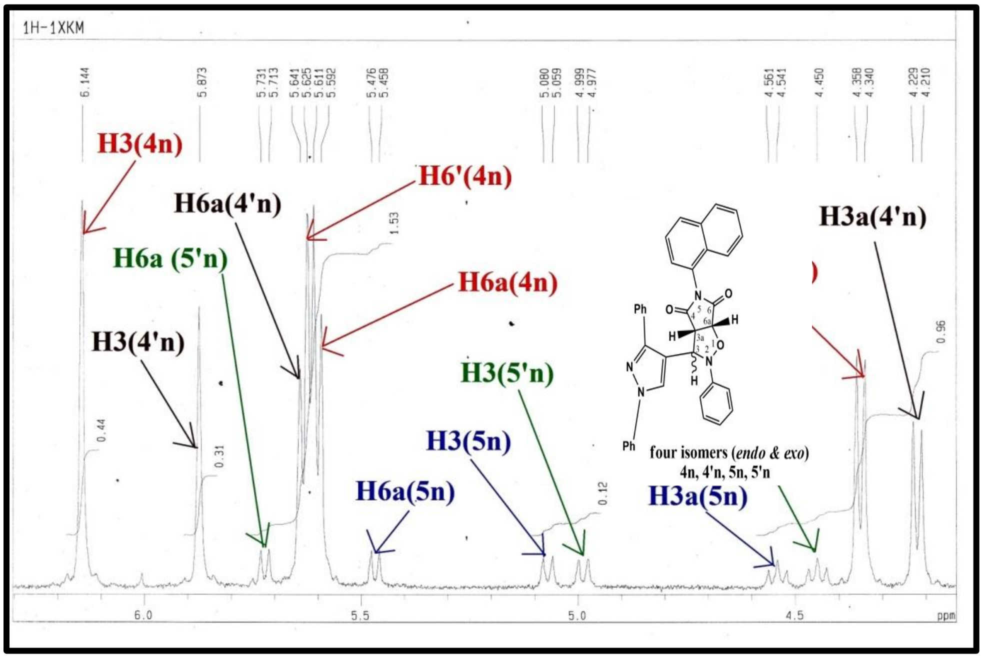

Endo atropisomers **(4n,4'n)**: (1 g, 55 %); white crystals; mp: 140-142°C. FTIR (KBr) (cm^{-1}): 3056(Ar. C-H), 2950(Aliph. C-H),1724(C=O). ^{1}H-NMR spectrum: δppm(400 MHz, $CDCl_3$) 4.17-4.2(m,2H, H3a(**4n,4'n**)), 5.28-5.3(m, 2H, H6a(**4n,4'n**)), 5.89-5.92(m, 2H, (H3(**4'n**), H6'(**4n**)), 6.13(s, 1H, H3(**4n**)), 7.19-8.19(m, 46H, ArH). I.R. Spectrum: ύ (cm^{-1}), The ratio of the isolated *endo* atropisomers **4n:4'n** is 56:44. Anal. Calcd for ($C_{36}H_{26}N_4O_3$) (%): C, 76.85; H, 4.66; N, 9.96. Found; C, 76.75; H, 4.35; N, 9.92.

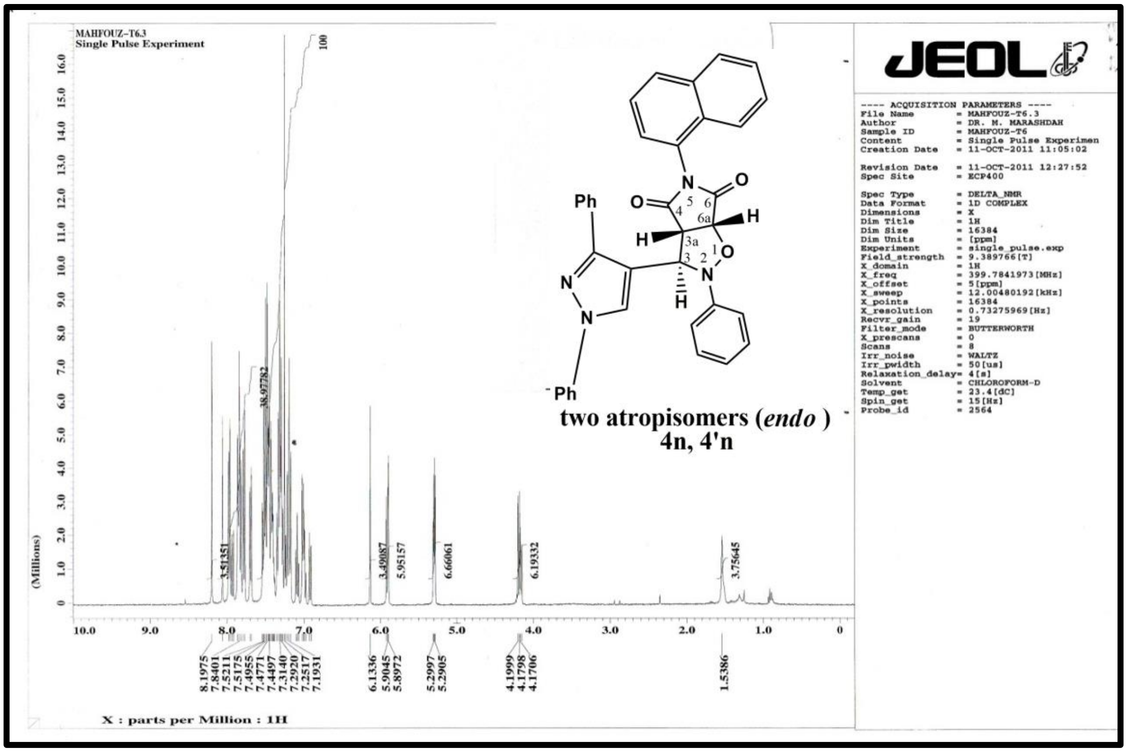

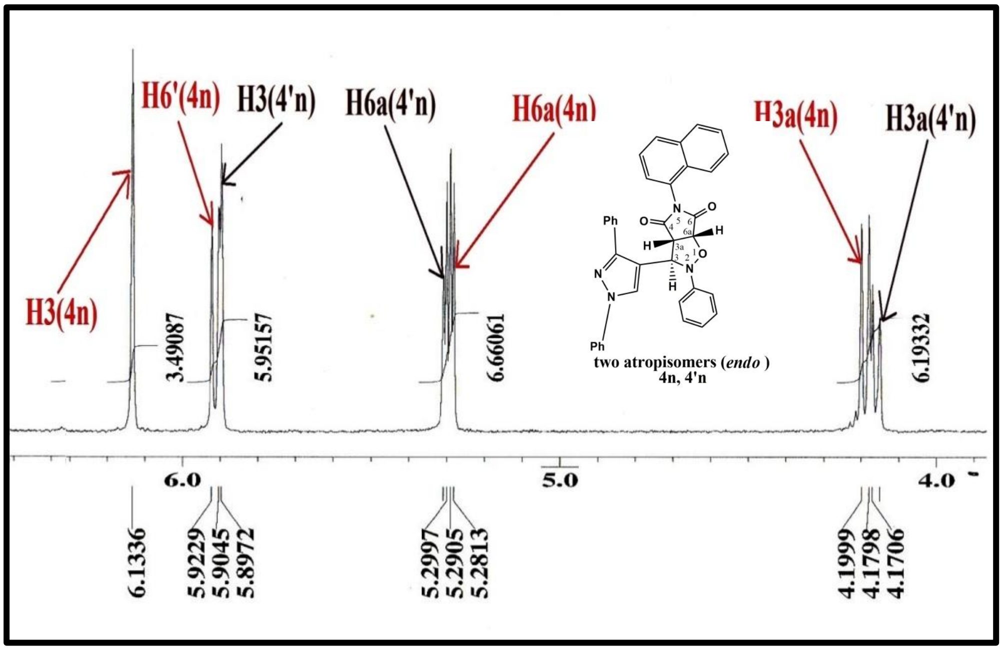

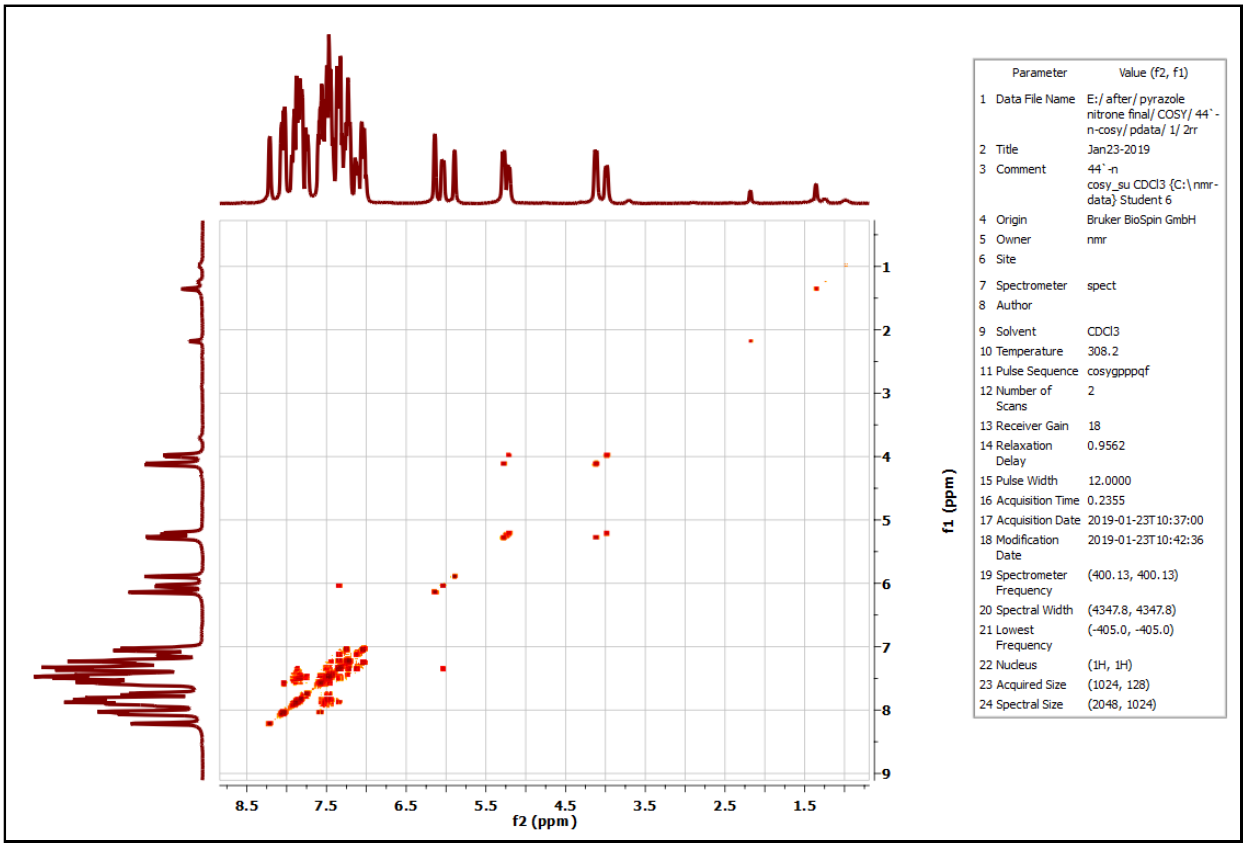
Parameter
Value (f2, f1)
1 Data File Name
E:/after/pyrazole nitrone final/COSY/44`-n-cosy/pdata/1/2rr
2 Title
Jan23-2019
3 Comment
44`-n cosy_su CDCl3 {C:\nmr-data} Student 6
4 Origin
Bruker BioSpin GmbH
5 Owner
nmr
6 Site
7 Spectrometer
spect
8 Author
9 Solvent
CDCl3
10 Temperature
308.2
11 Pulse Sequence
cosygpppqf
12 Number of Scans
2
13 Receiver Gain
18
14 Relaxation Delay
0.9562
15 Pulse Width
12.0000
16 Acquisition Time
0.2355
17 Acquisition Date
2019-01-23T10:37:00
18 Modification Date
2019-01-23T10:42:36
19 Spectrometer Frequency
(400.13, 400.13)
20 Spectral Width
(4347.8, 4347.8)
21 Lowest Frequency
(-405.0, -405.0)
22 Nucleus
(1H, 1H)
23 Acquired Size
(1024, 128)
24 Spectral Size
(2048, 1024)
f1 (ppm)
f2 (ppm)

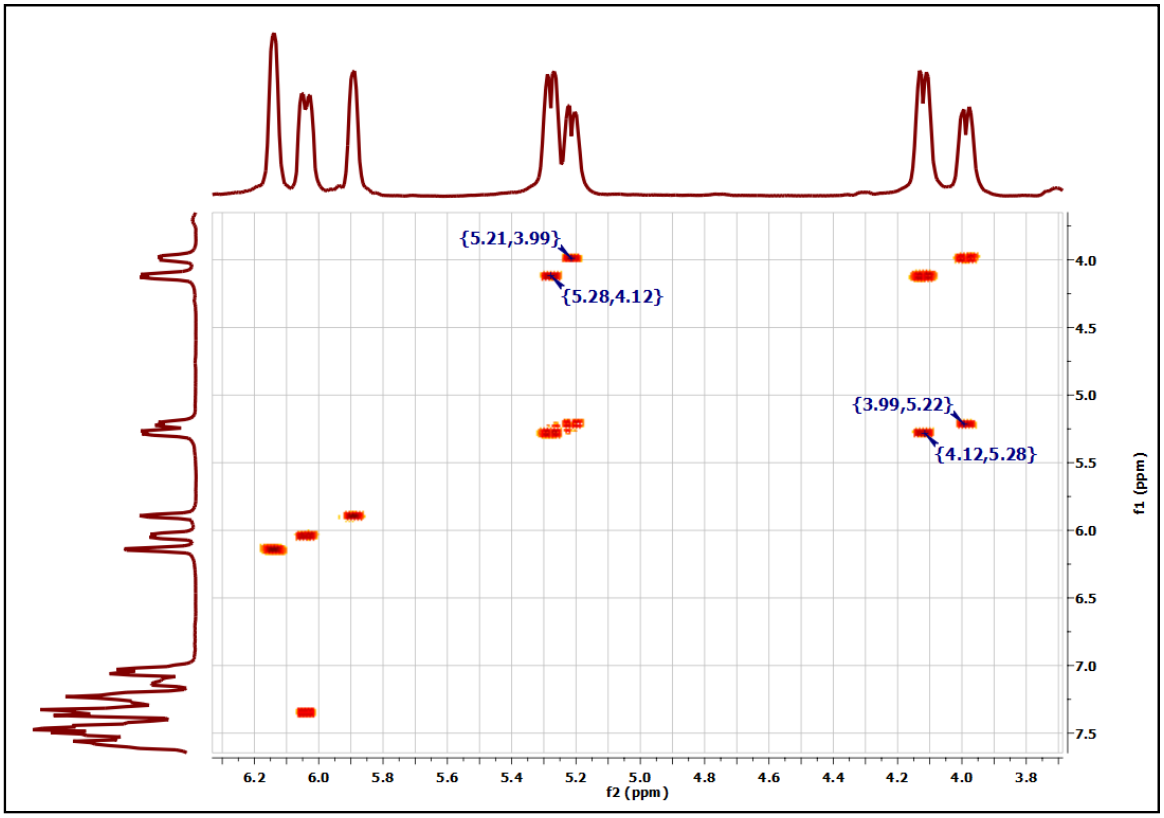
{5.21,3.99}
{5.28,4.12}
{3.99,5.22}
{4.12,5.28}
f1 (ppm)
f2 (ppm)

Cycloaddition with N-(2-naphthyl) maleimide (3h)

Formation of 3-(1,3-diphenyl-1H-pyrazol-4-yl)-5-(naphthalen-2-yl)-2-phenyldihydro-2H-pyrrolo[3,4-d]isoxazole-4,6(5H,6aH)-dione $C_{36}H_{26}N_4O_3$.

Reaction mixture**(4h, 5h)**: ^{1}H-NMR spectrum: δ ppm (400 MHz, $CDCl_3$) 4.16(d, *J* 7.2 Hz, 1H, H3a(*endo*)), 4.47(t, *J* 8.8 Hz, 1H, H3a(*exo*)), 4.94(d, *J* 9.2 Hz, 1H, H3(*exo*)), 5.48-5.54(m, 2H, H6a(*endo,exo*)), 6.04(s, 1H, H3(*endo*)), 6.7-8.7(m, 40H, Ar H), 9.98(s, 1H, Nitrone CH=N).

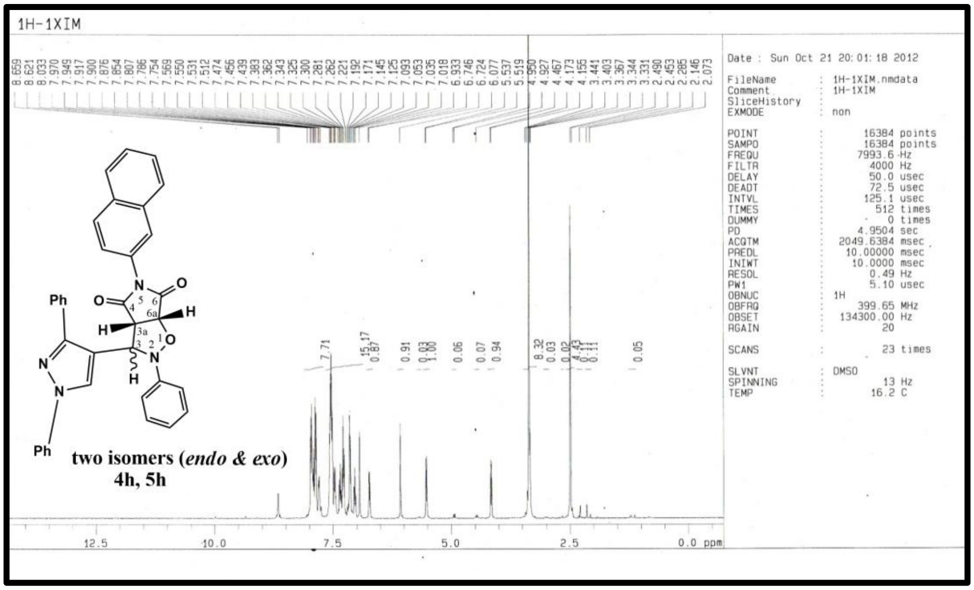

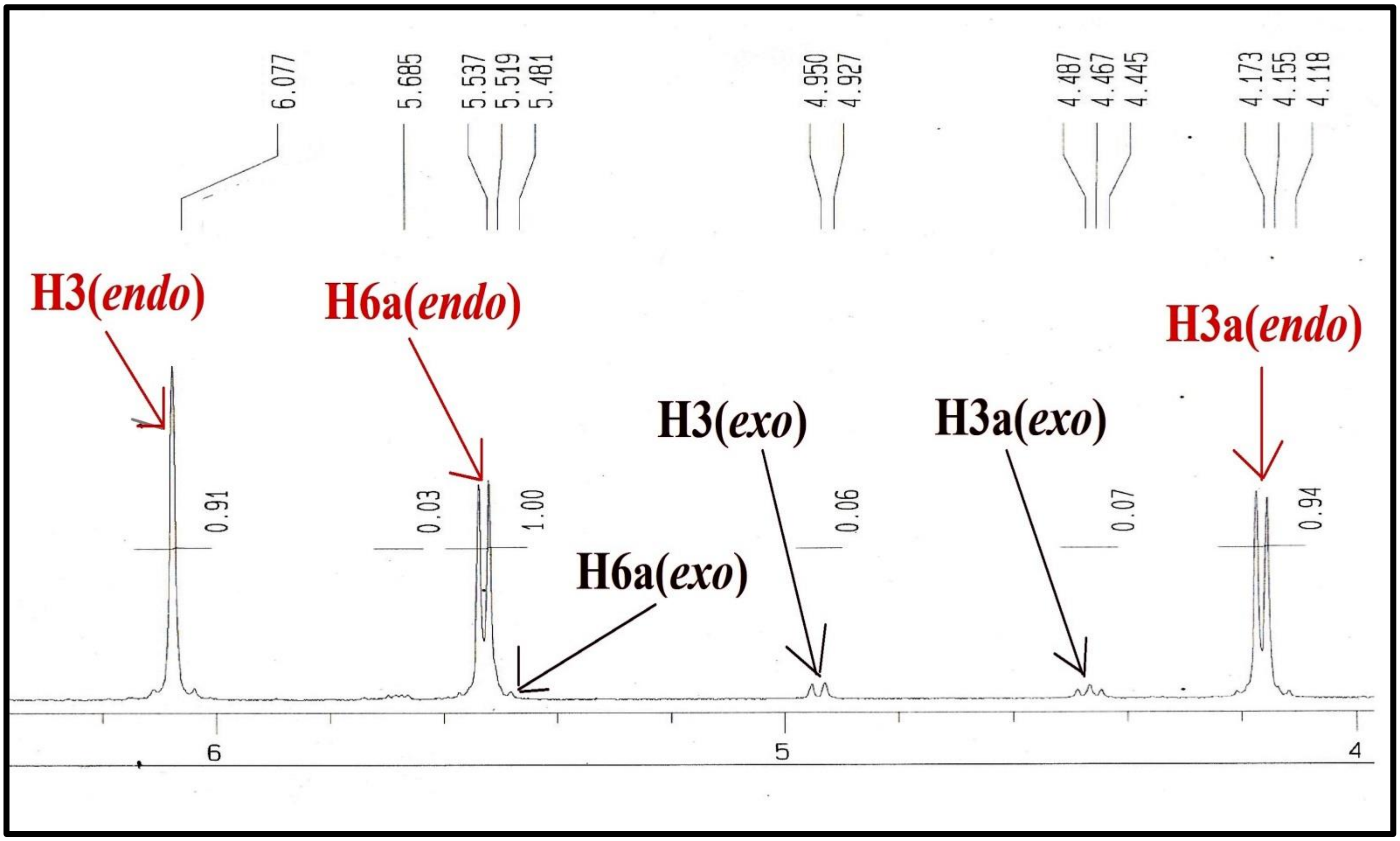

Endo-isomer **(4h)**: (1.2 g, 68 %); white crystals; mp: 140-141°C. FTIR (KBr) (cm^{-1}): 3058(Ar. C-H), 2970(Aliph. C-H), 1720(C=O). ^{1}H-NMR spectrum: δ ppm (400 MHz, $CDCl_3$) 4.05(d, *J* 7.32 Hz, 1H, H3a), 5.16(d, *J* 7.32 Hz, 1H, H6a), 6.11(s, 1H, H3), 6.73-8.18(m, 23H, Ar H). $^{13}C\{H\}$NMR spectrum: δ ppm (100.5 MHz, $CDCl_3$) 24.8, 56.1, 63.4(3 Aliphatic **C**); 114.7, 119.1(2), 123.23, 123.3, 125.4, 125.6, 126.7, 126.8, 127.1, 127.4, 127.8, 128.3, 128.4(4), 128.6, 129, 129.1(3), 132.25, 132.30, 129.6(2), 129.7(2), 139.8, 148.4, 150.3(31 Aromatic **C**); 172.8, 173.9 (2 **C**=O). Anal. Calcd for ($C_{36}H_{26}N_4O_3$) (%): C, 76.85; H, 4.66; N, 9.96. Found; C, 77.01; H, 4.46; N, 9.92.

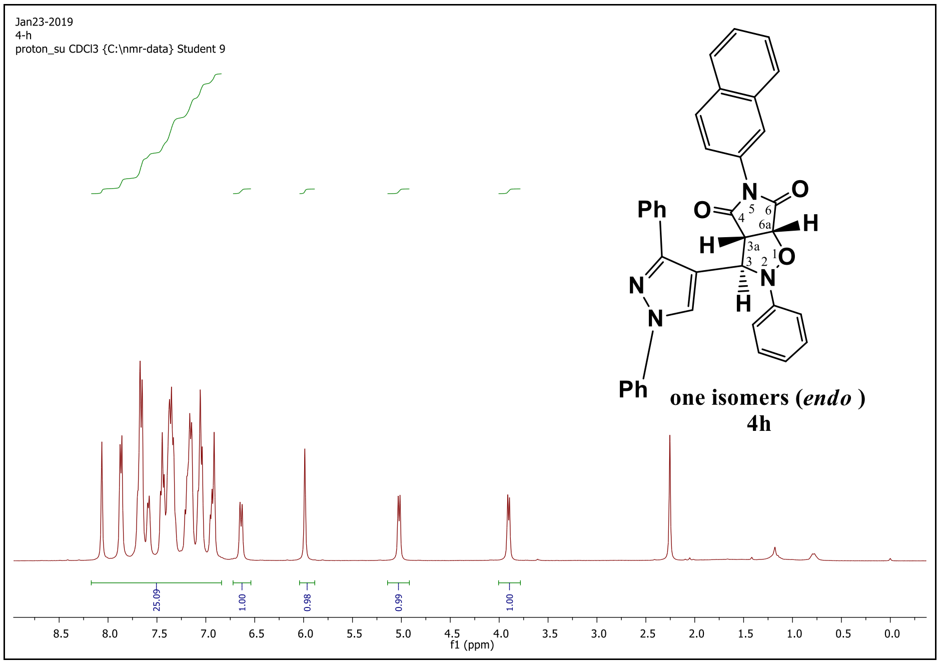

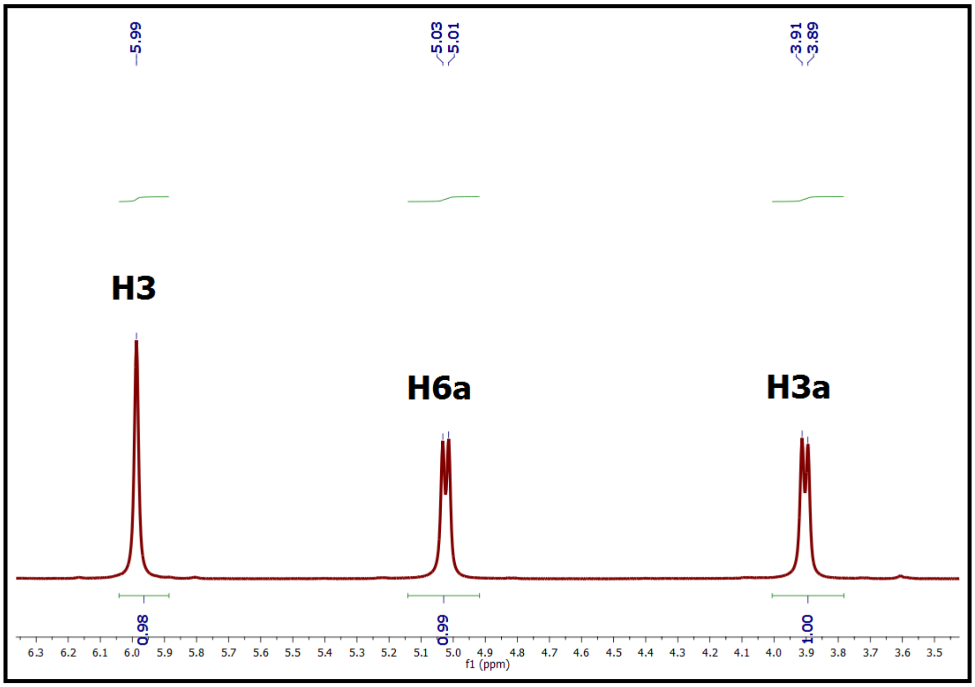

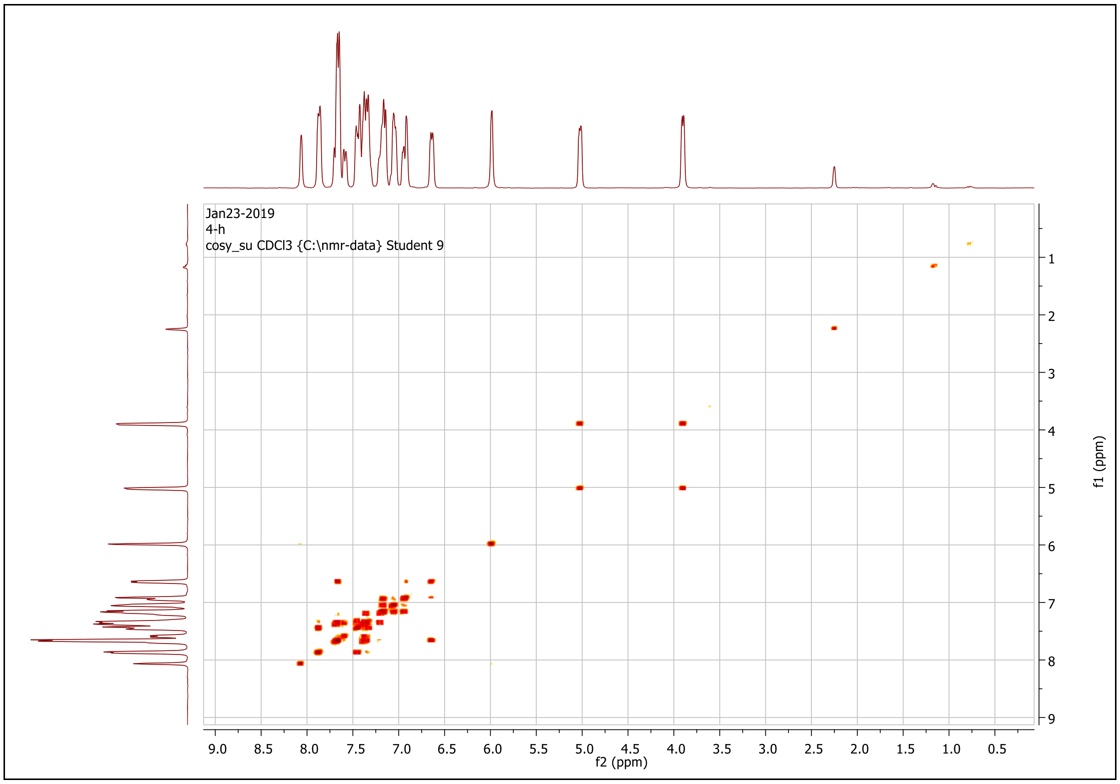
Jan23-2019
4-h
cosy_su CDCl3 {C:\nmr-data} Student 9
f1 (ppm)
f2 (ppm)
9.0 8.5 8.0 7.5 7.0 6.5 6.0 5.5 5.0 4.5 4.0 3.5 3.0 2.5 2.0 1.5 1.0 0.5
1 2 3 4 5 6 7 8 9

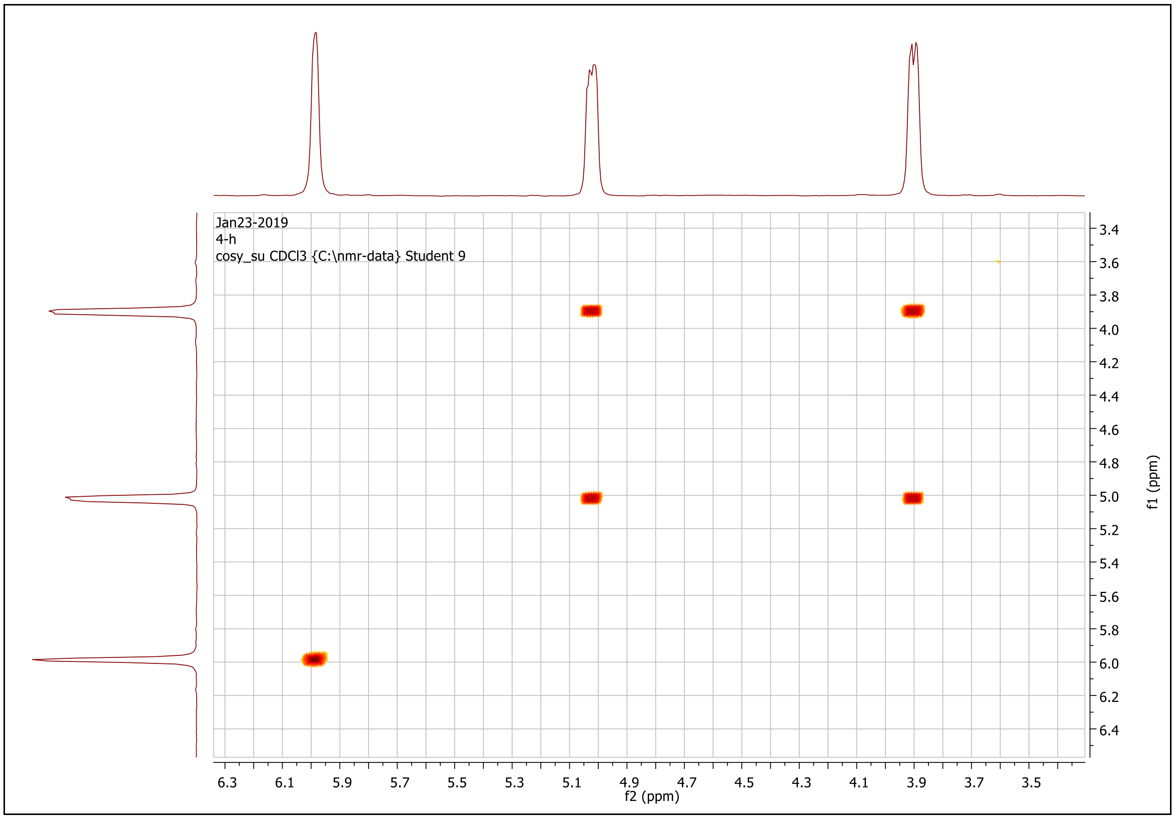
Jan23-2019
4-h
cosy_su CDCl3 {C:\nmr-data} Student 9
f1 (ppm)
f2 (ppm)
6.3 6.1 5.9 5.7 5.5 5.3 5.1 4.9 4.7 4.5 4.3 4.1 3.9 3.7 3.5
3.4 3.6 3.8 4.0 4.2 4.4 4.6 4.8 5.0 5.2 5.4 5.6 5.8 6.0 6.2 6.4

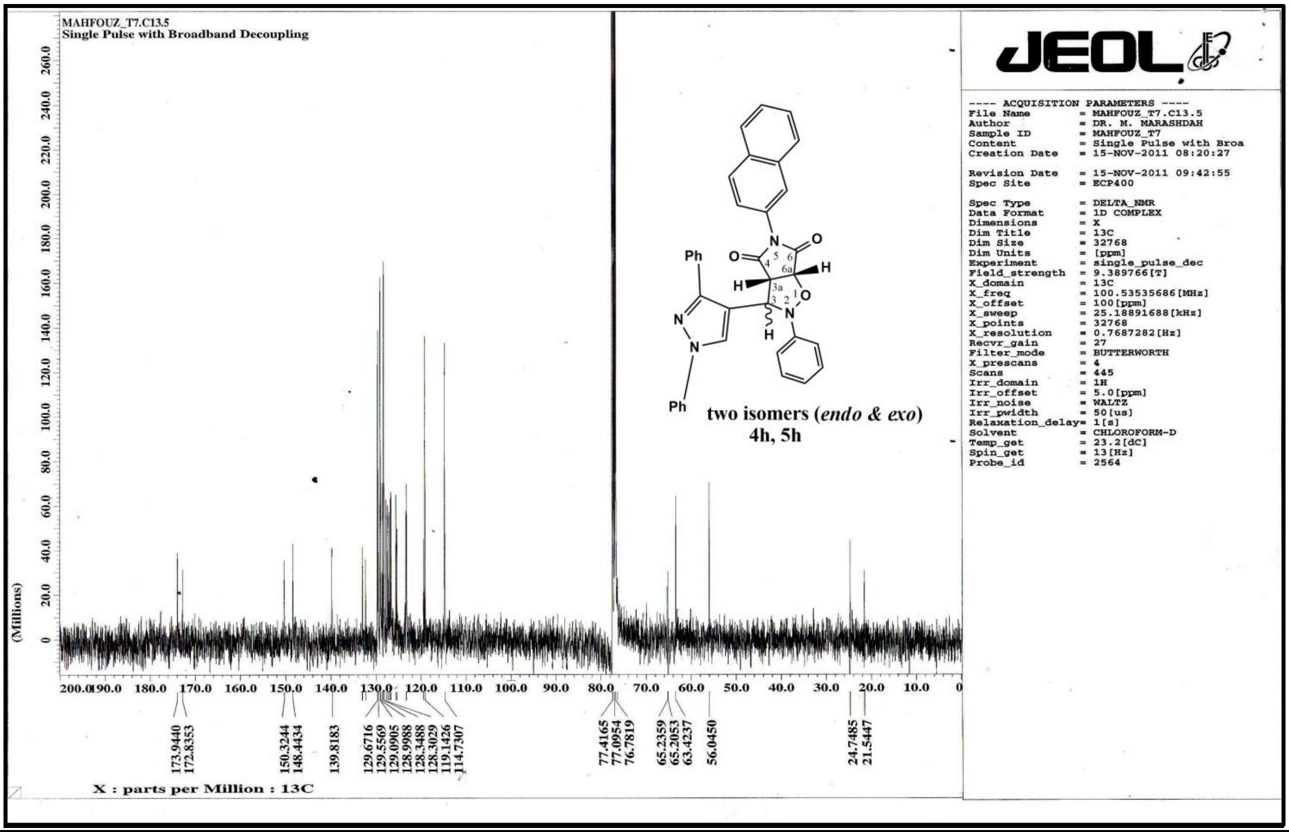
MAHFOUZ_T7.C13.5
Single Pulse with Broadband Decoupling
two isomers (*endo* & *exo*)
4h, 5h
(Millions)
X : parts per Million : 13C
173.9440
172.8353
150.3244
148.4434
139.8183
129.6716
129.5569
129.0905
128.9988
128.3488
128.3029
119.1426
114.7307
77.4165
77.0954
76.7819
65.2359
65.2053
63.4237
56.0450
24.7485
21.5447
JEOL
---- ACQUISITION PARAMETERS ----
File Name = MAHFOUZ_T7.C13.5
Author = DR. M. MARASHDAH
Sample ID = MAHFOUZ_T7
Content = Single Pulse with Broa
Creation Date = 15-NOV-2011 08:20:27
Revision Date = 15-NOV-2011 09:42:55
Spec Site = ECP400
Spec Type = DELTA_NMR
Data Format = 1D COMPLEX
Dimensions = X
Dim Title = 13C
Dim Size = 32768
Dim Units = [ppm]
Experiment = single_pulse_dec
Field_strength = 9.389766[T]
X_domain = 13C
X_freq = 100.53535686[MHz]
X_offset = 100[ppm]
X_sweep = 25.18891688[kHz]
X_points = 32768
X_resolution = 0.7687282[Hz]
Recvr_gain = 27
Filter_mode = BUTTERWORTH
X_prescans = 4
Scans = 445
Irr_domain = 1H
Irr_offset = 5.0[ppm]
Irr_noise = WALTZ
Irr_pwidth = 50[us]
Relaxation_delay= 1[s]
Solvent = CHLOROFORM-D
Temp_get = 23.2[dC]
Spin_get = 13[Hz]
Probe_id = 2564

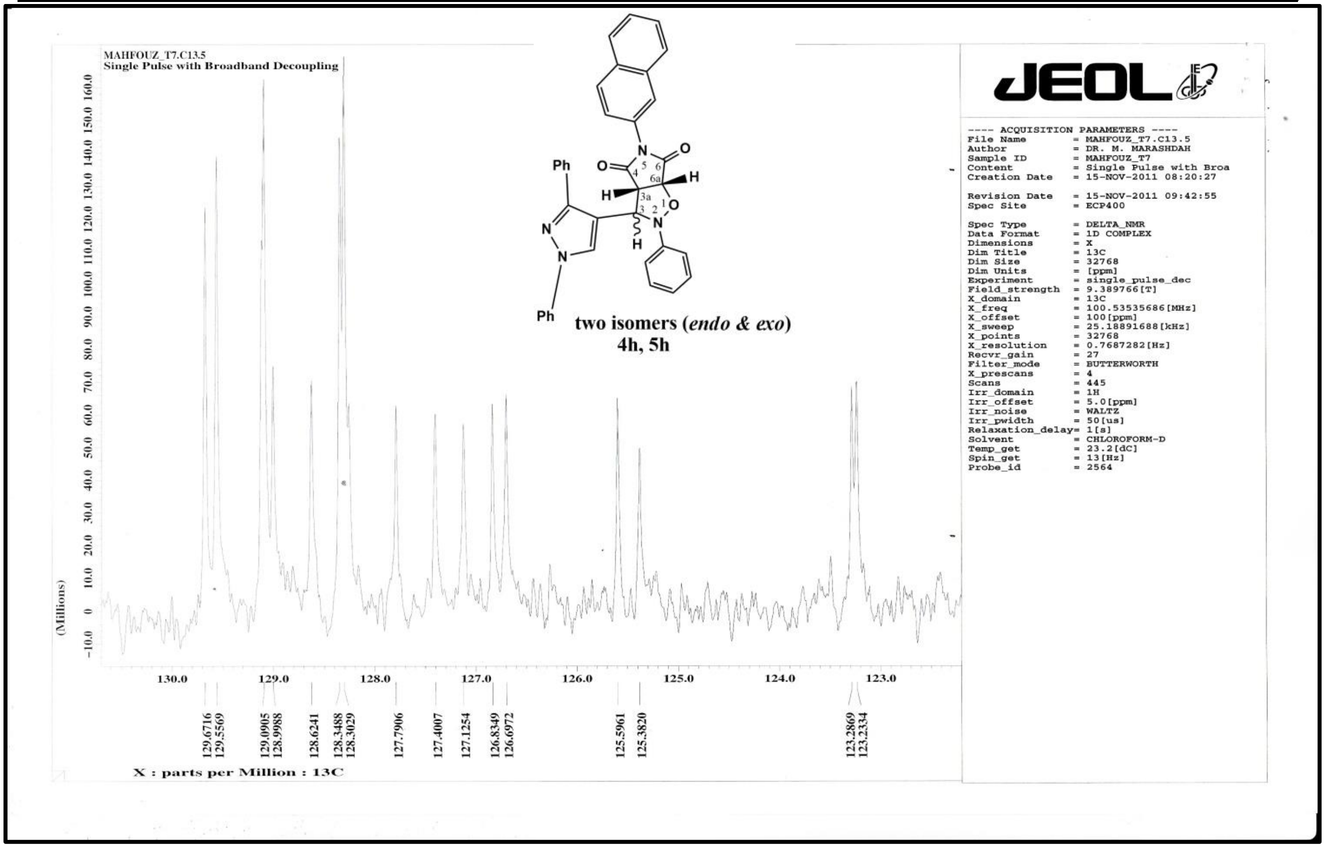
MAHFOUZ_T7.C13.5
Single Pulse with Broadband Decoupling
two isomers (*endo* & *exo*)
4h, 5h
(Millions)
X : parts per Million : 13C
129.6716
129.5569
129.0905
128.9988
128.6241
128.3488
128.3029
127.7906
127.4007
127.1254
126.8349
126.6972
125.5961
125.3820
123.2869
123.2334
JEOL
---- ACQUISITION PARAMETERS ----
File Name = MAHFOUZ_T7.C13.5
Author = DR. M. MARASHDAH
Sample ID = MAHFOUZ_T7
Content = Single Pulse with Broa
Creation Date = 15-NOV-2011 08:20:27
Revision Date = 15-NOV-2011 09:42:55
Spec Site = ECP400
Spec Type = DELTA_NMR
Data Format = 1D COMPLEX
Dimensions = X
Dim Title = 13C
Dim Size = 32768
Dim Units = [ppm]
Experiment = single_pulse_dec
Field_strength = 9.389766[T]
X_domain = 13C
X_freq = 100.53535686[MHz]
X_offset = 100[ppm]
X_sweep = 25.18891688[kHz]
X_points = 32768
X_resolution = 0.7687282[Hz]
Recvr_gain = 27
Filter_mode = BUTTERWORTH
X_prescans = 4
Scans = 445
Irr_domain = 1H
Irr_offset = 5.0[ppm]
Irr_noise = WALTZ
Irr_pwidth = 50[us]
Relaxation_delay= 1[s]
Solvent = CHLOROFORM-D
Temp_get = 23.2[dC]
Spin_get = 13[Hz]
Probe_id = 2564

Exo-isomer **(5h)**: (0.03 g, 1.8 %); white crystals; mp: 174-176°C. 1H-NMR spectrum: δ ppm (400 MHz, $CDCl_3$) 4.11(t, *J* 8.8 Hz, 1H, H3a), 5.16(d, *J* 8.8 Hz, 1H, H3), 5.36(d, *J* 8 Hz, 1H, H6a), 6.7-8.2(m, 23H, Ar H). Mass spectrum (electron impact): m/e (%) 526.2(17), 435.9 (38), 324(100), 243(85), 76 (34).

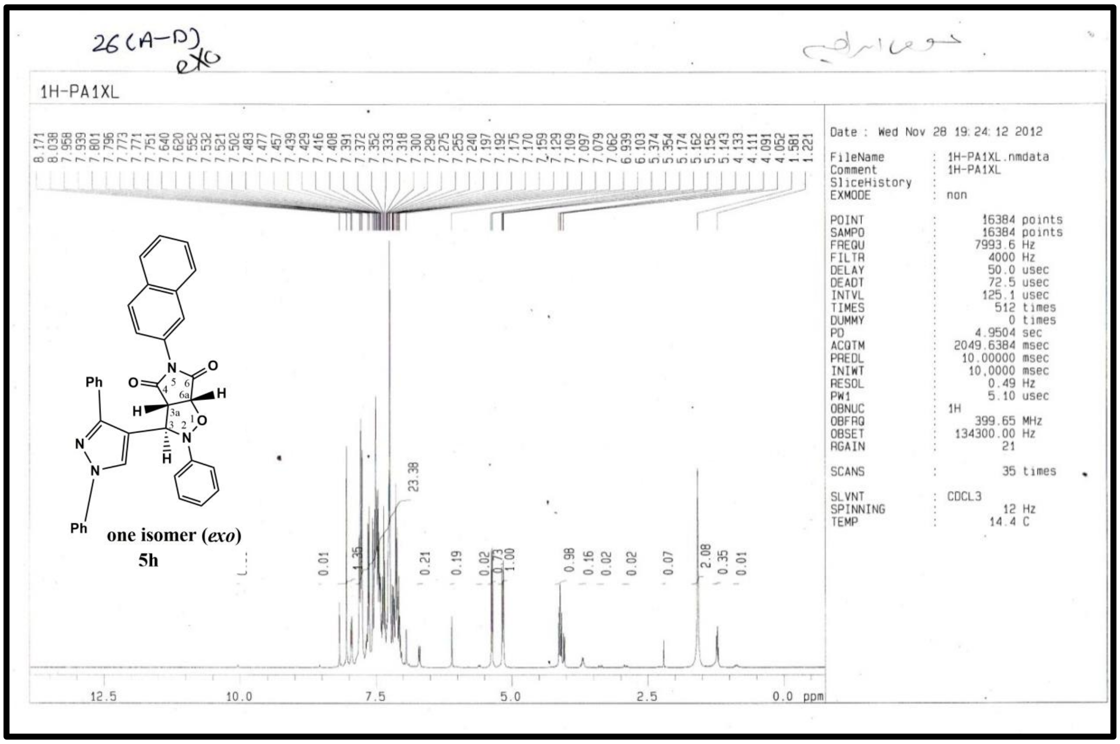

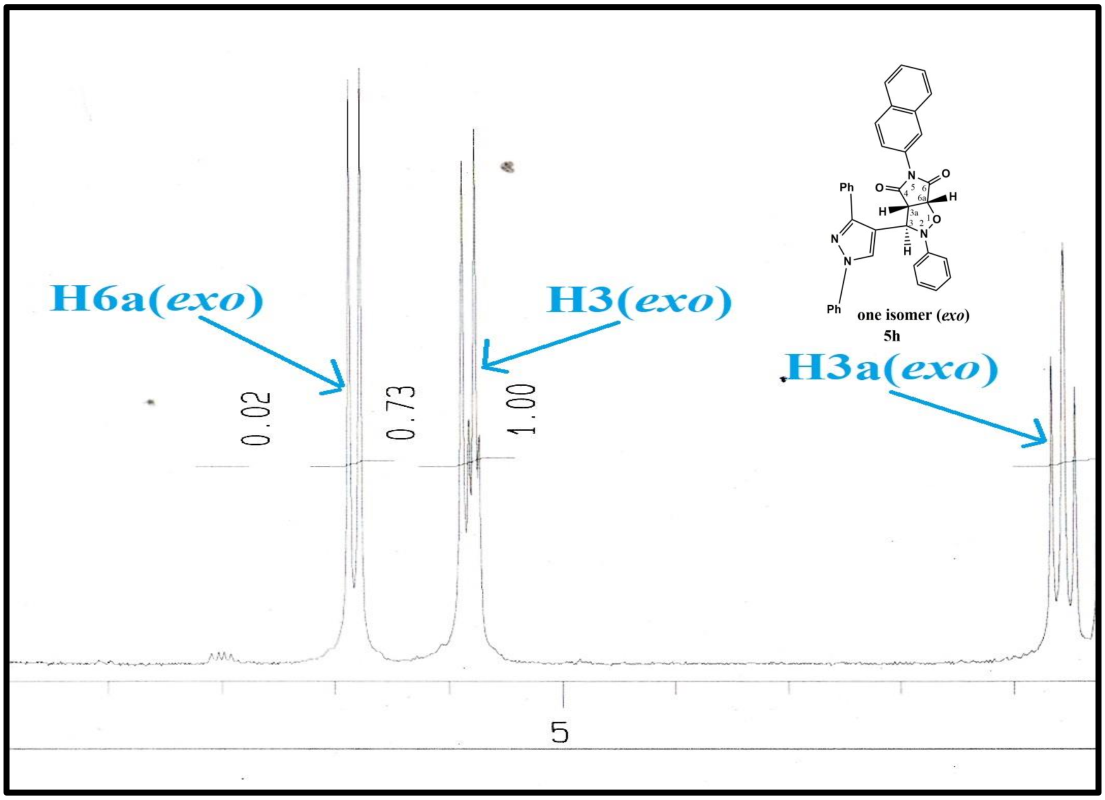

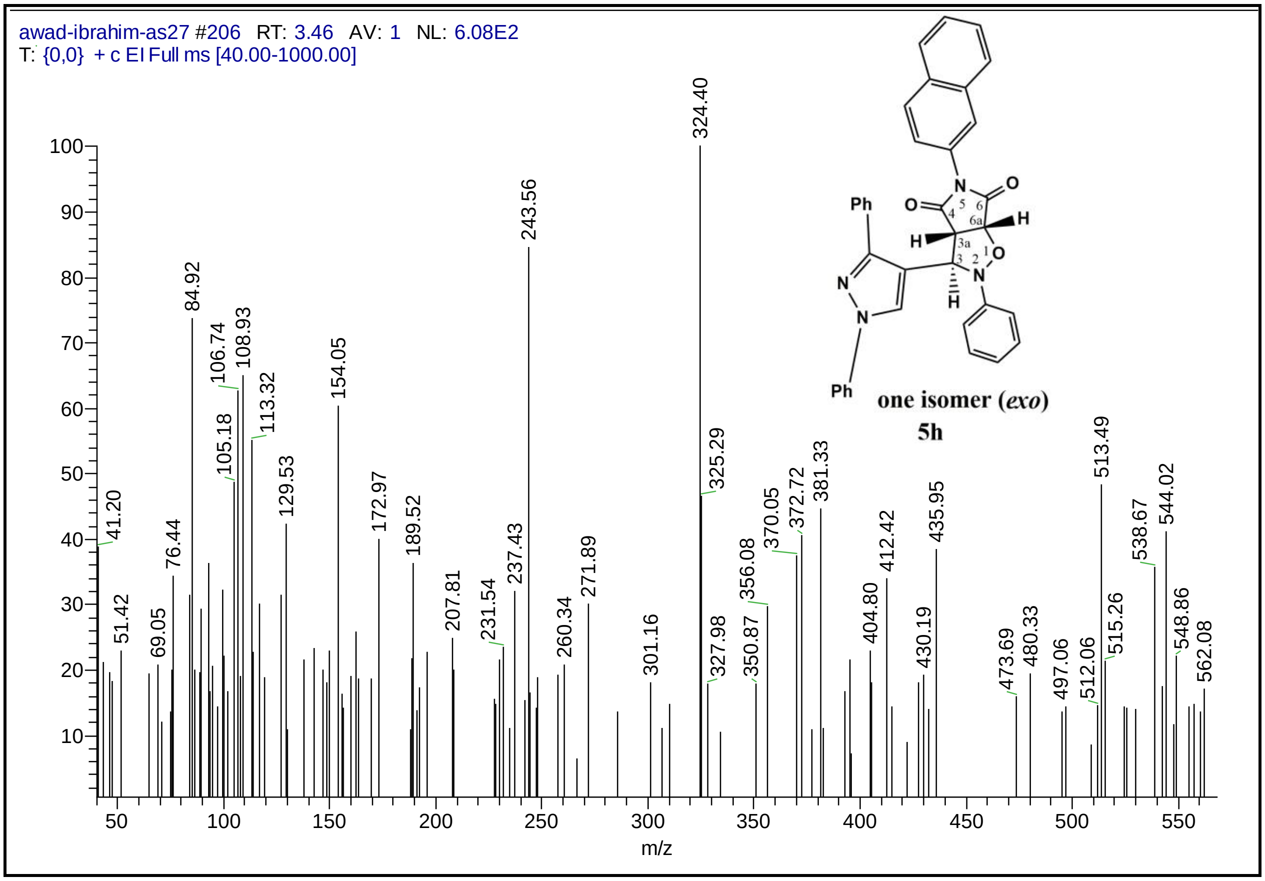
awad-ibrahim-as27 #206 RT: 3.46 AV: 1 NL: 6.08E2
T: {0,0} + c EI Full ms [40.00-1000.00]
m/z
324.40
243.56
84.92
106.74
108.93
113.32
154.05
105.18
129.53
41.20
76.44
51.42
69.05
172.97
189.52
207.81
231.54
237.43
260.34
271.89
301.16
325.29
327.98
350.87
356.08
370.05
372.72
381.33
404.80
412.42
430.19
435.95
473.69
480.33
497.06
512.06
513.49
515.26
538.67
544.02
548.86
562.08
Ph
N
O
H
one isomer (exo)
5h

Cycloaddition with N-(benzyl) maleimide (3i)

Formation of 5-benzyl-3-(1,3-diphenyl-1H-pyrazol-4-yl)-2-phenyldihydro-2H-pyrrolo[3,4-d]isoxazole-4,6(5H,6aH)-dione $C_{33}H_{26}N_4O_3$.

Reaction mixture **(4i,5i)**:^{1}H-NMR spectrum: δ ppm (400 MHz, $CDCl_3$) 4.1(d, *J* 7.6 Hz, 1H, H3a(*endo*)), 4.35(dd, 2H, CH_2(*endo*))4.61 (b, 2H, CH_2 (*exo*)), 4.76(d, *J* 8.8Hz, 1H, H3(*exo*)), 5.36(d,*J* 7.6Hz,1H, H6a(*exo*)), 5.45(d, *J* 6.8Hz, 1H, H6a(*exo*)), 5.68(s, 1H, H3(*endo*)), 6.8-8.6(m, 42H, Ar H), 9.98 (s, 1H, Nitrone C<u>H</u>=N).

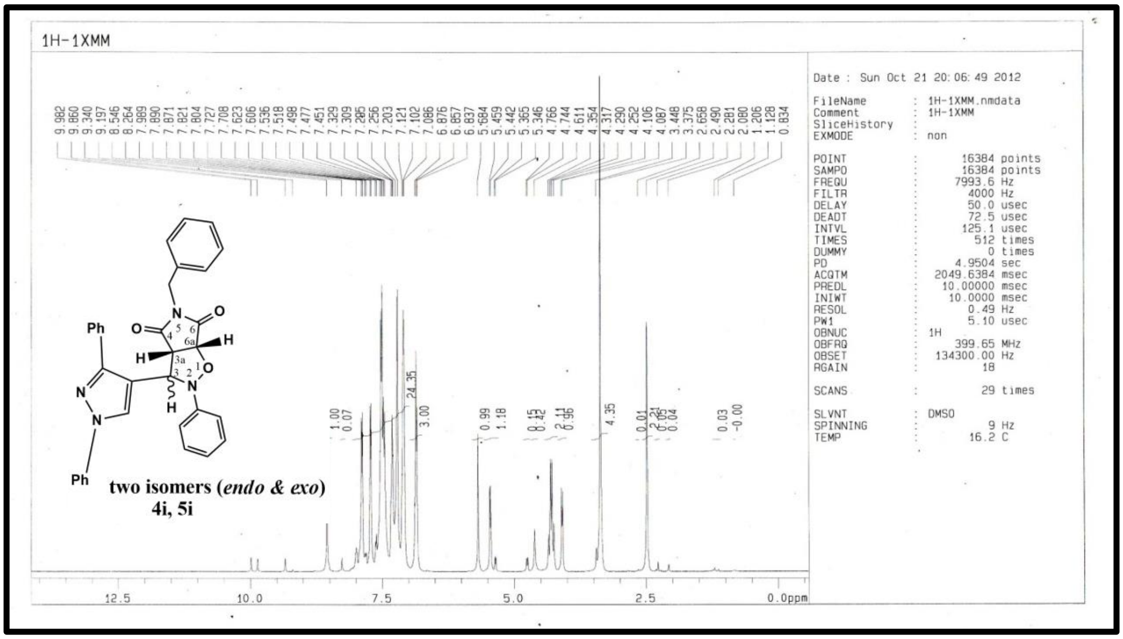

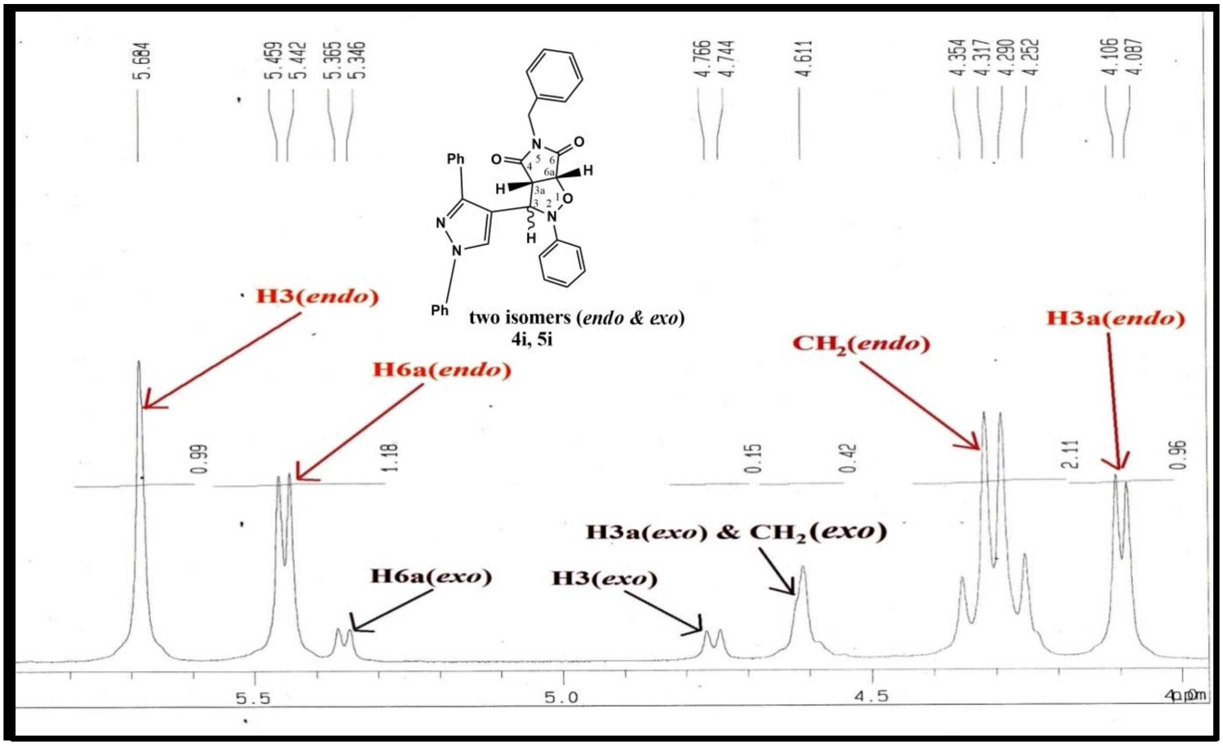

Endo-isomer **(4i)**: (1.1 g, 65 %); white crystals; mp: 176-178°C. FTIR(KBr) (cm^{-1}); 3150(Ar.C-H), 2900(Aliph. C-H), 1712(C=O). ^{1}H-NMR spectrum: δ ppm(400 MHz, $CDCl_3$) 3.8(d, *J* 7.32 Hz, 1H, H3a), 4.4(dd, 2H, CH_2), 4.99(d, *J* 7.32 Hz, 1H, H6a), 5.8(s, 1H, H3), 6.9-8 (m, 21H, Ar H). ^{13}C{H}NMR spectrum: δ ppm(100.5 MHz, $CDCl_3$) 42.9, 25.2, 56.3, 62.5(4 Aliphatic **C**); 115.2(2), 118.6, 119.1(2), 123.1, 126.8, 127.1, 128.1, 128.4 (2), 128.6, 128.8(3), 128.98(2), 129 (3), 129.5 (2), 132.3, 134.6, 139.8, 147.2, 152.5(27 Aromatic **C**); 173.4, 74.3 (2 **C**=O). Anal. Calcd for ($C_{33}H_{26}N_4O_3$) (%): C, 75.27; H, 4.98; N, 10.64. Found; C, 74.91; H, 5.16; N, 10.38.

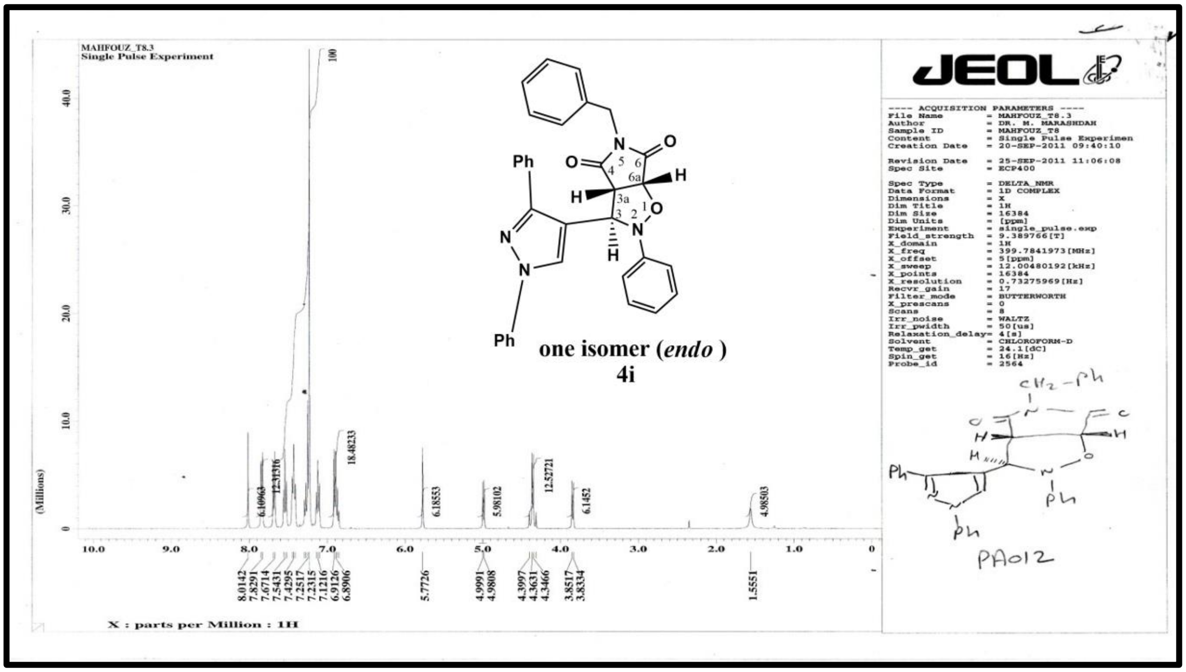

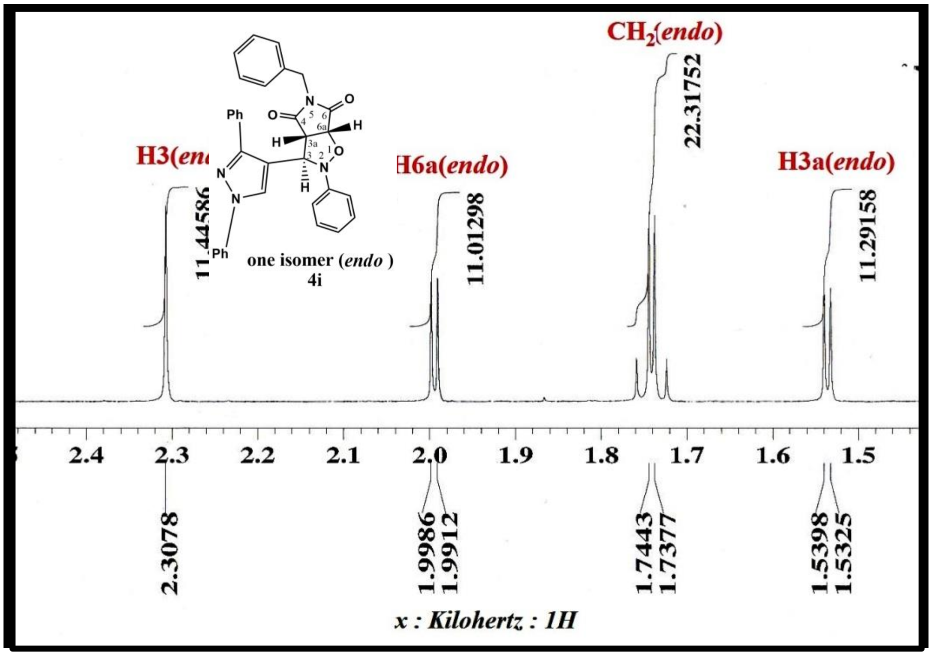

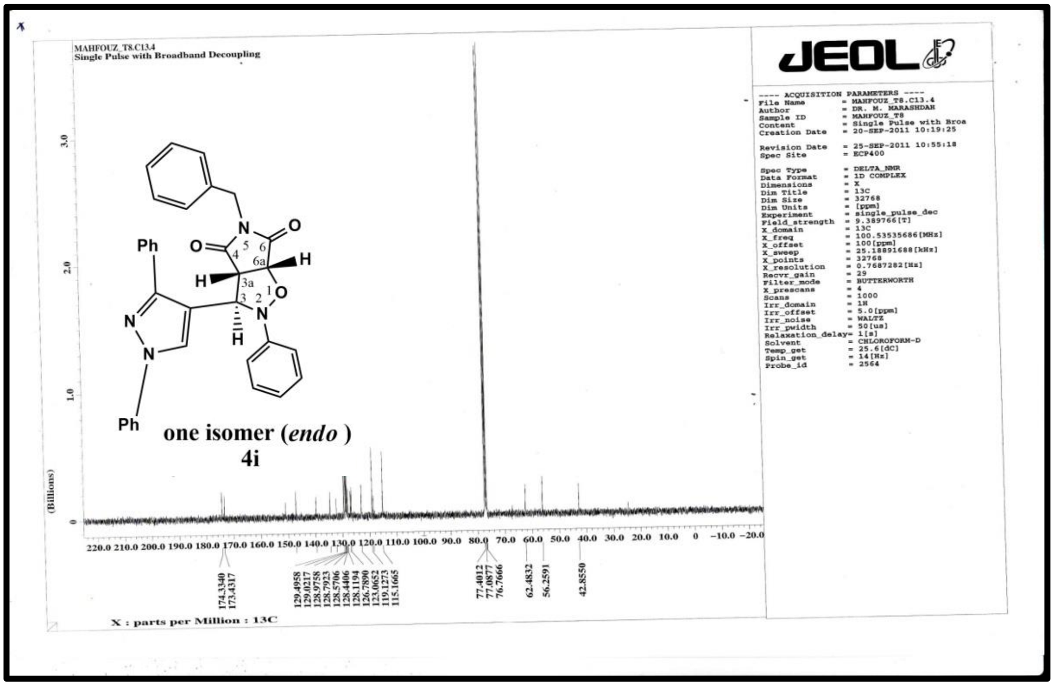
MAHFOUZ_T8.C13.4
Single Pulse with Broadband Decoupling
one isomer (endo)
4i
X : parts per Million : 13C

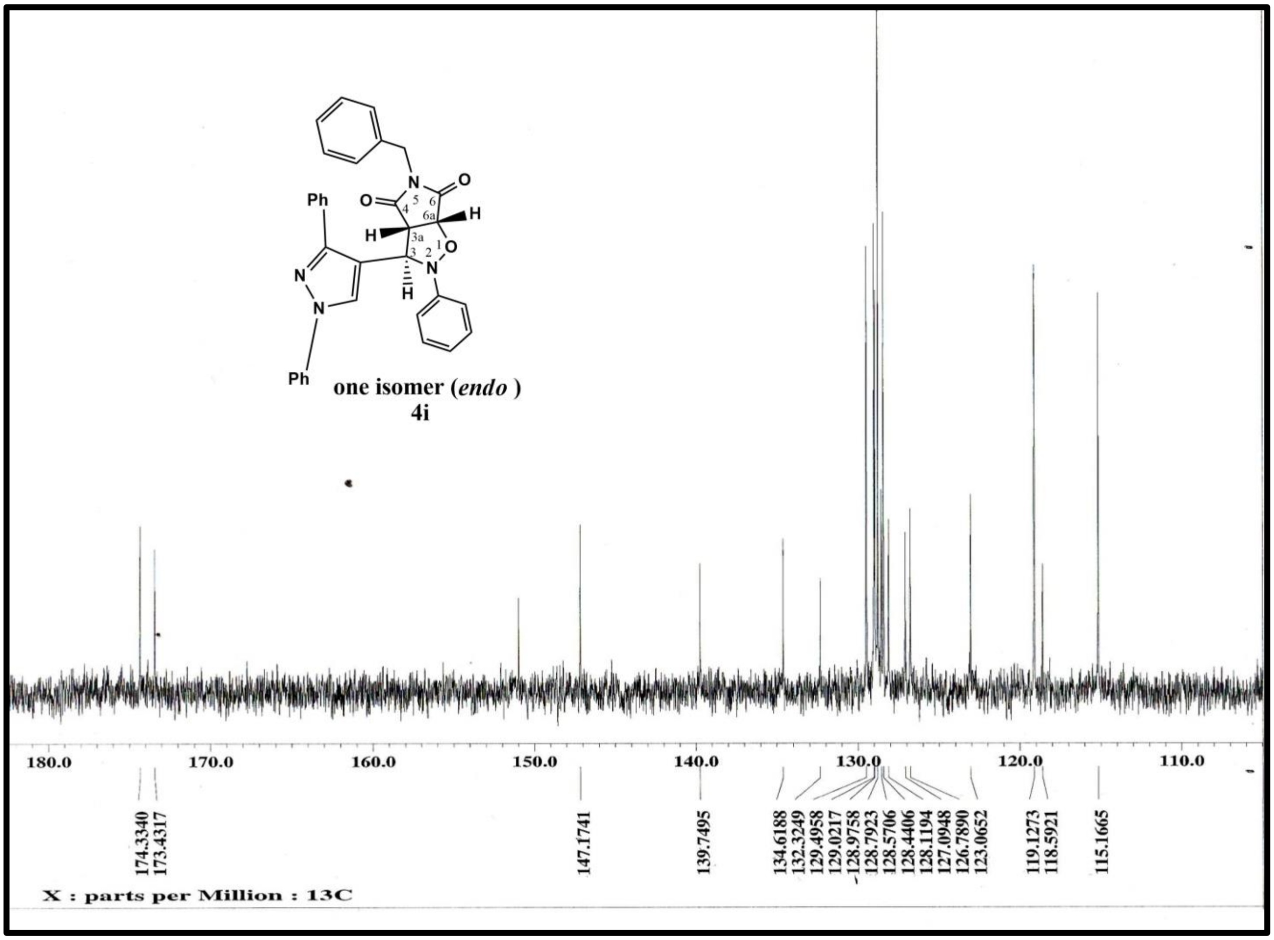
one isomer (endo)
4i
174.3340
173.4317
147.1741
139.7495
134.6188
132.3249
129.4958
129.0217
128.9758
128.7923
128.5706
128.4406
128.1194
127.0948
126.7890
123.0652
119.1273
118.5921
115.1665
X : parts per Million : 13C

Exo-isomer (**5i**): (0.02 g, 1.14 %); white crystals; mp: 158-160°C. Mass spectrum (electron impact): m/e (%) 526.5(23), 435(44), 274.6(100), 246.8(92)

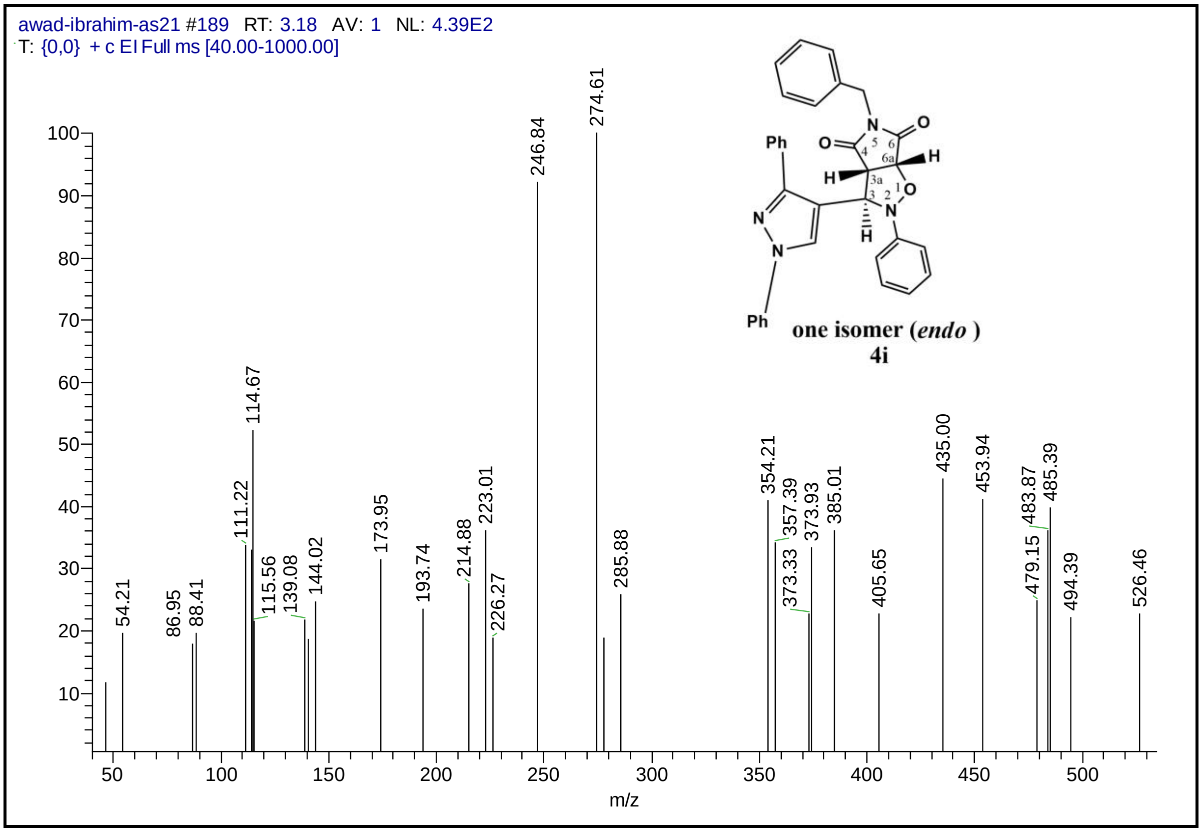

Cycloaddition with N-(4-acetylphenyl) maleimide (3j)

Formation of 5-(4-acetylphenyl)-3-(1,3-diphenyl-1H-pyrazol-4-yl)-2-phenyldihydro-2H-pyrrolo[3,4-d]isoxazole-4,6(5H,6aH)-dione $C_{34}H_{26}N_4O_4$.

Reaction mixture **(4j,5j)**:^{1}H-NMR spectrum: δ ppm (400 MHz, CDCl$_3$) 2.49(s, 3H, COCH$_3$(*endo*)), 2.52(s, 3H, COCH$_3$(*exo*)), 4.12(d, *J* 7.2 Hz, 1H, H3a(*endo*)), 4.43(t, *J* 8.4 Hz, 1H, H3a(*exo*)), 4.9(d, *J* 8.4 Hz, 1H, H3(*exo*)), 5.38(d, *J* 8.8 Hz, 1H, H6a(*exo*)), 5.48(d, *J* 6.8 Hz, 1H, H6a(*endo*)), 6.(s, 1H, H3(*endo*)), 6.7-8.6 (m, 40H, Ar H), 9.98 (s, 1H, Nitrone CH=N).

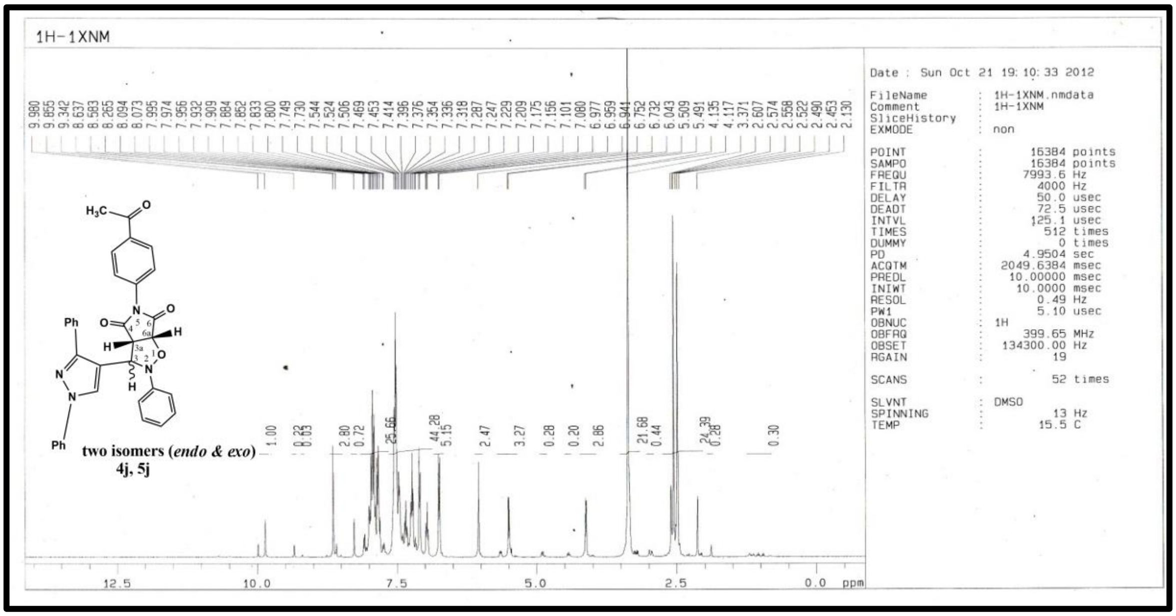

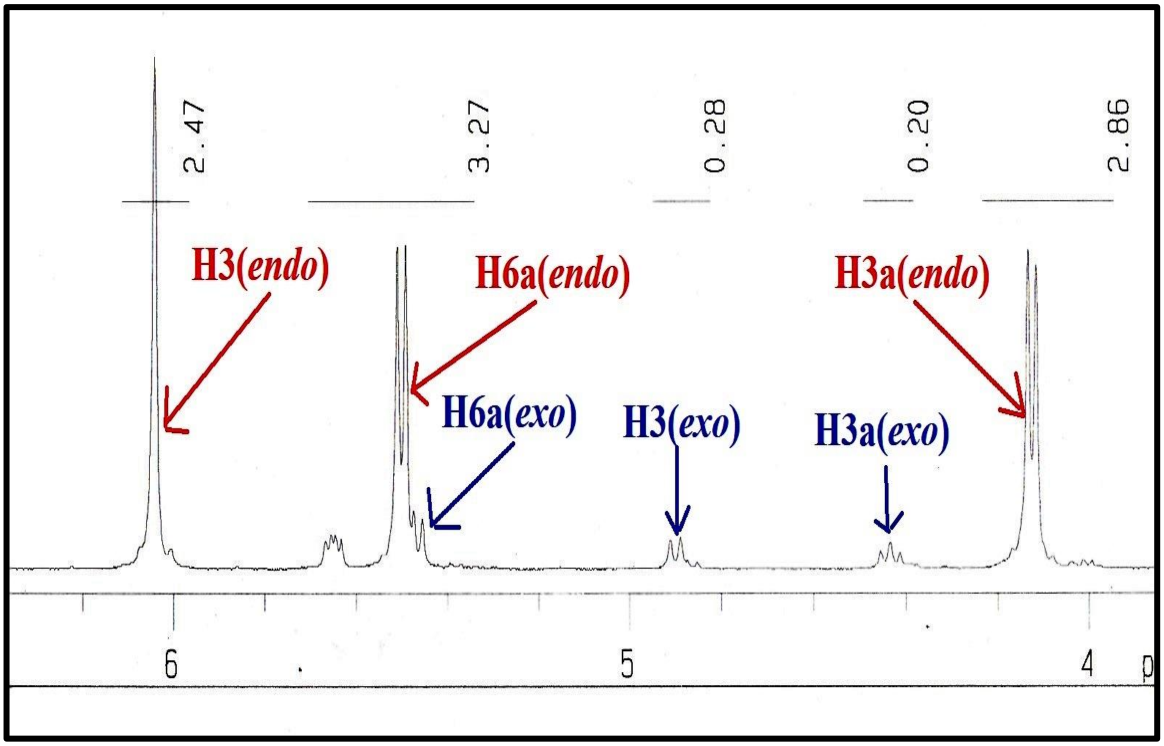

Endo-isomer **(4j)***:* (1.1 g, 60 %); white crystals; mp: 148-150ºC. FTIR (KBr) (cm^{-1}): 3030(Ar. C-H), 2950(Aliph C-H), 1725(C=O). ^{1}H-NMR spectrum: δ ppm(400 MHz, $CDCl_3$) 2.6(s, 3H, $COCH_3$), 4(d, *J* 7.3 Hz, 1H, H3a), 5.1(d, *J* 7.3 Hz, 1H, H6a), 6.1(s, 1H, H3), 6.8-8.2(m, 20H, Ar-H). ^{13}C{H}NMR spectrum: δ ppm(100.5 MHz, CDCl3) 26.7, 55.9, 63.4(3 Aliphatic C); 114.7(2), 119.08(2), 119.1, 123.3, 125.3, 126.1(2), 126.8, 127.3, 128.3(2), 128.6, 128.9(2), (2), 129.5(2), 129.5 (2), 132.2, 134.8, 137, 139.7, 148.2, 150.3(27 Aromatic C); 172.2, 173.32, 196.9 (3 C=O). Anal. Calcd for ($C_{33}H_{26}N_4O_3$) (%): C, 73.63; H, 4.73; N, 10.10. Found; C, 73.92;H, 5.07; N, 9.82.

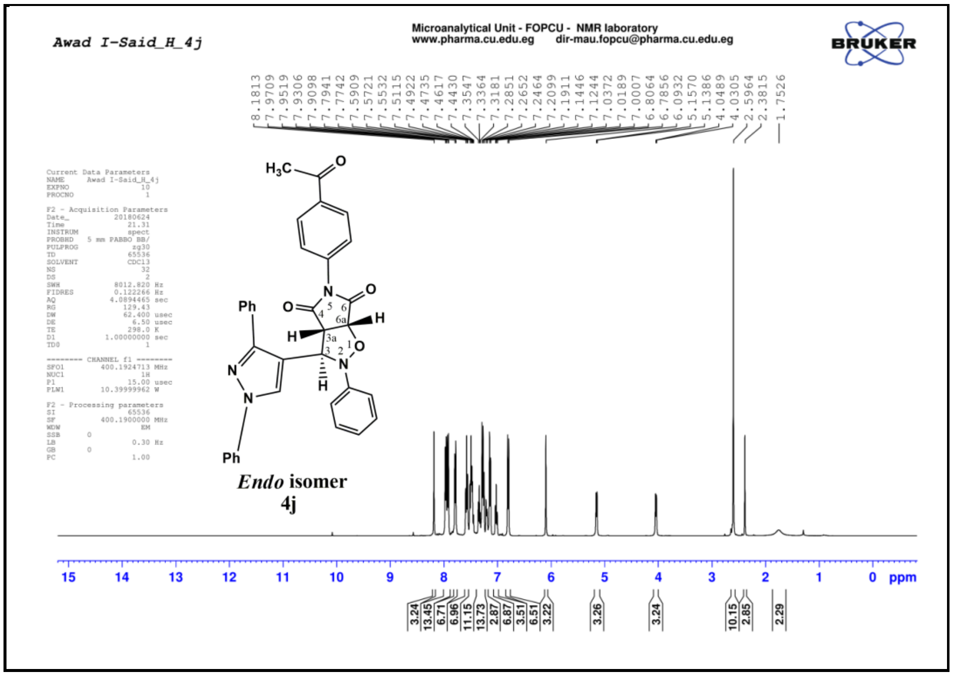

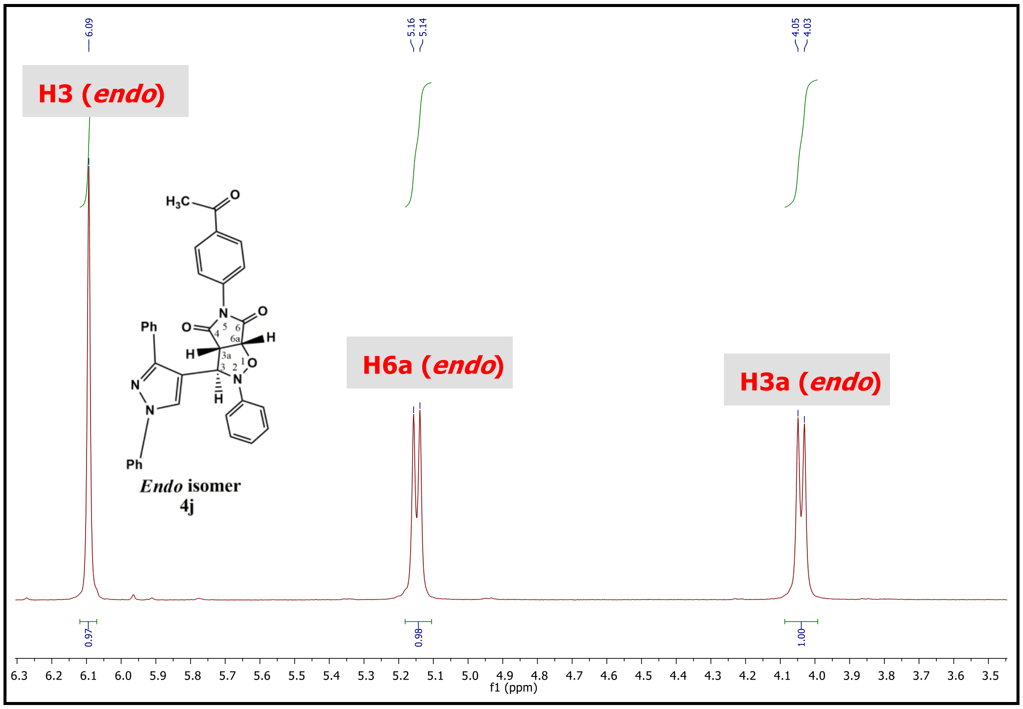

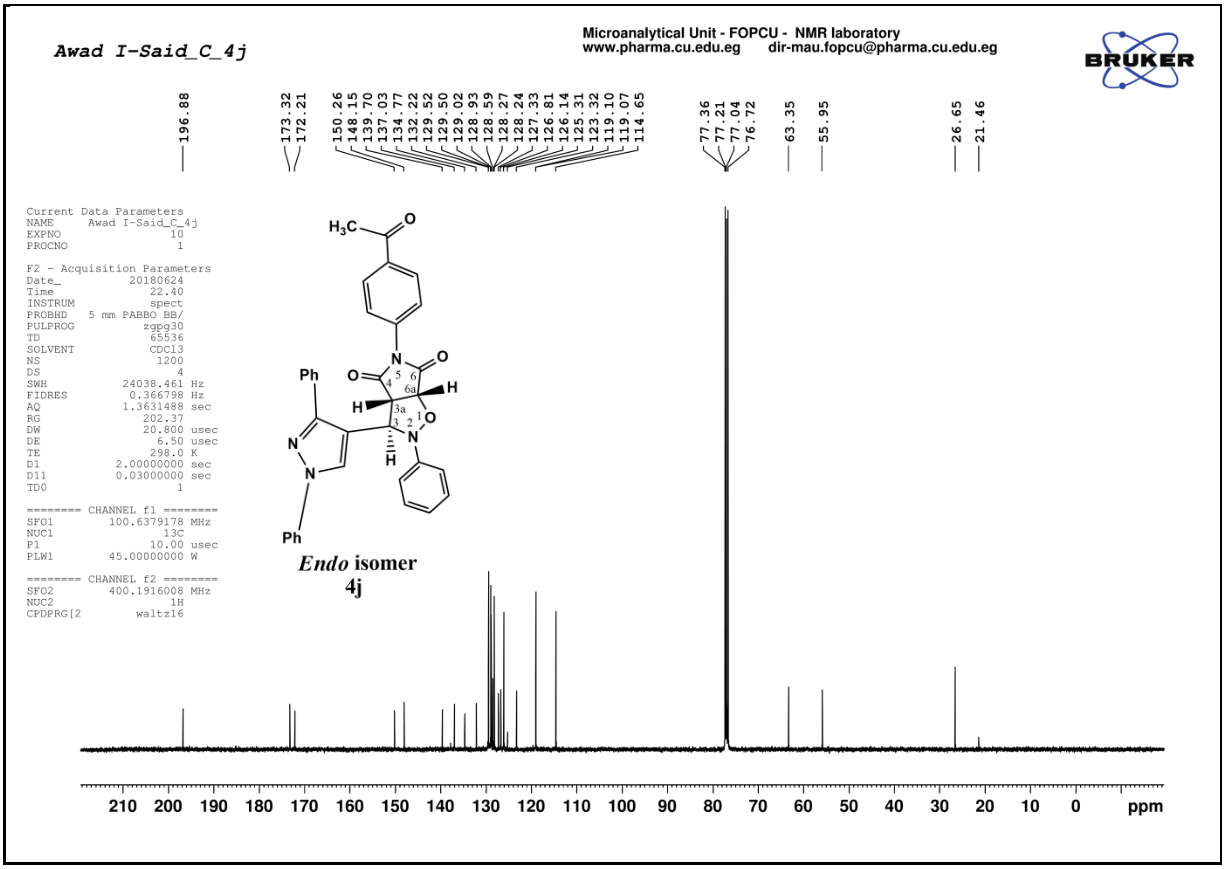
Awad I-Said_C_4j
Microanalytical Unit - FOPCU - NMR laboratory
www.pharma.cu.edu.eg dir-mau.fopcu@pharma.cu.edu.eg
BRUKER
196.88
173.32
172.21
150.26
148.15
139.70
137.03
134.77
132.22
129.52
129.50
129.02
128.93
128.59
128.27
128.24
127.33
126.81
126.14
125.31
123.32
119.10
119.07
114.65
77.36
77.21
77.04
76.72
63.35
55.95
26.65
21.46
Current Data Parameters
NAME Awad I-Said_C_4j
EXPNO 10
PROCNO 1
F2 - Acquisition Parameters
Date_ 20180624
Time 22.40
INSTRUM spect
PROBHD 5 mm PABBO BB/
PULPROG zgpg30
TD 65536
SOLVENT CDCl3
NS 1200
DS 4
SWH 24038.461 Hz
FIDRES 0.366798 Hz
AQ 1.3631488 sec
RG 202.37
DW 20.800 usec
DE 6.50 usec
TE 298.0 K
D1 2.00000000 sec
D11 0.03000000 sec
TD0 1
======== CHANNEL f1 ========
SFO1 100.6379178 MHz
NUC1 13C
P1 10.00 usec
PLW1 45.00000000 W
======== CHANNEL f2 ========
SFO2 400.1916008 MHz
NUC2 1H
CPDPRG[2 waltz16
Endo isomer
4j
ppm

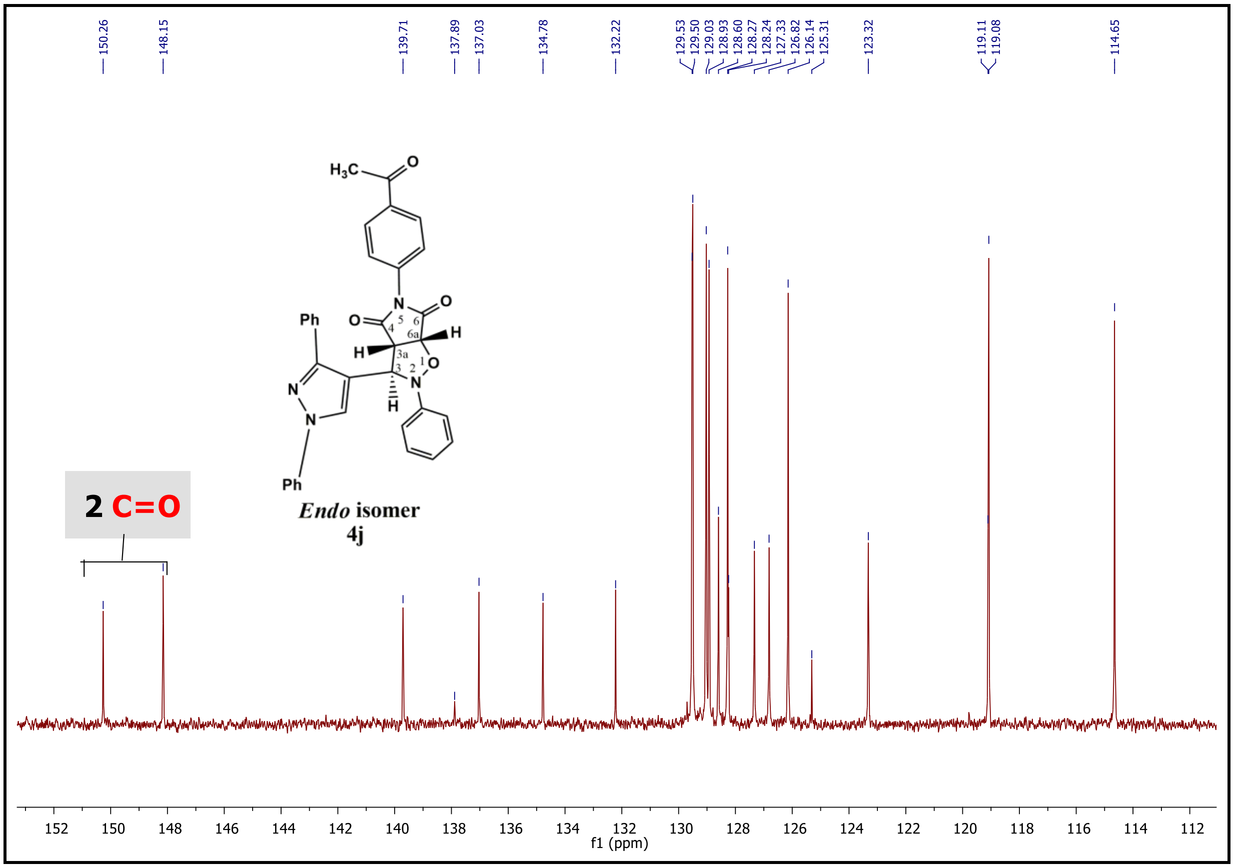
150.26
148.15
139.71
137.89
137.03
134.78
132.22
129.53
129.50
129.03
128.93
128.60
128.27
128.24
127.33
126.82
126.14
125.31
123.32
119.11
119.08
114.65
2 C=O
Endo isomer
4j
f1 (ppm)

Cycloaddition with N-(2-flourophenyl) maleimide (3o)

Formation of (3R(S),3aS(R),6aR(S))-3-(1,3-diphenyl-1H-pyrazol-4-yl)-5-(2-fluorophenyl)-2-phenyldihydro-2H-pyrrolo[3,4-d]isoxazole-4,6(5H,6aH)-dione $C_{32}H_{23}FN_4O_3$.

Reaction Reaction mixture **(4o.5o)**:^{1}H-NMR spectrum: δ ppm (400 MHz, $CDCl_3$) 4.37(d, *J* 5.6Hz, 1H, H3a(*endo*)), 4.46(m,1H, H3a(*exo*)),4.91(d, *J* 8.4 Hz, 1H, H3(*exo*)), 5.46(b, 1H, H6a(*exo*)), 5.59(d, *J* 6.4 Hz, 1H, H6a(*endo*)), 5.85(s, 1H, H3(*endo*)), 6.8-8.6 (m, 40H, Ar H), 9.98(s, 1H, Nitrone C<u>H</u>=N).

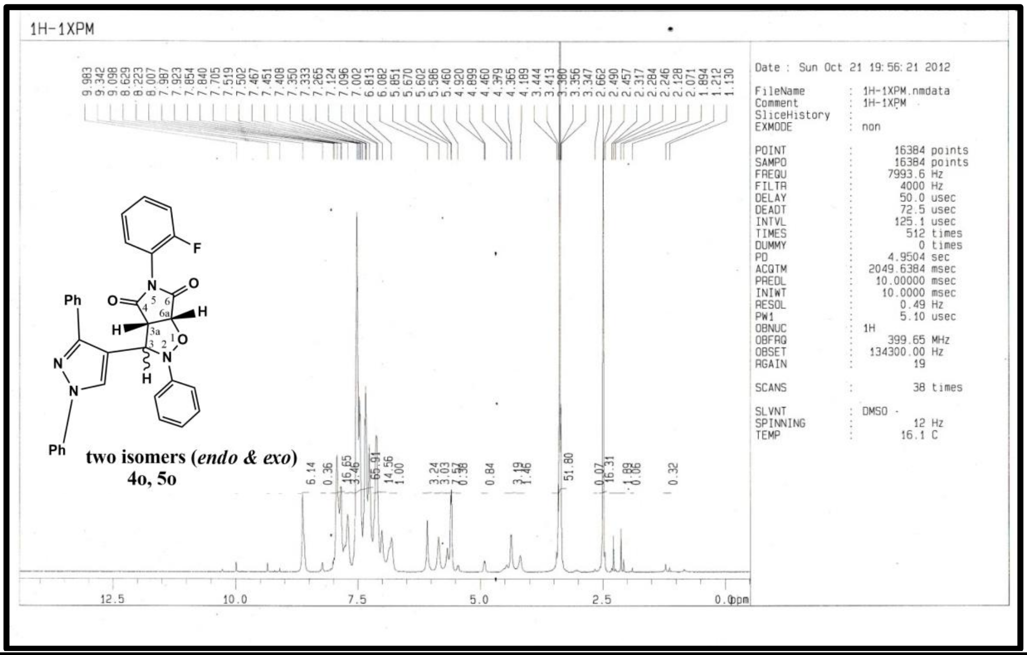

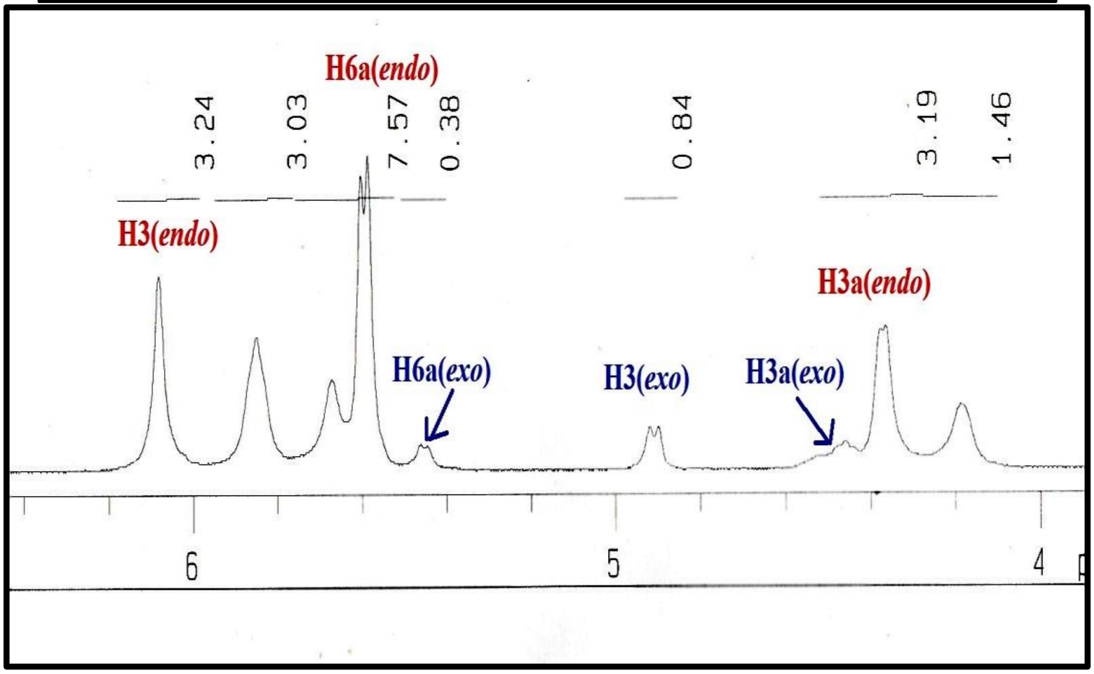

Endo-isomer **(4o)**: (0.9 g, 55 %); white crystals; mp: 188-190°C. FTIR (KBr) (cm^{-1}): 3030(Ar. C-H), 2950(Aliph. C-H), 1730(C=O). ^{1}H-NMR spectrum: δ ppm(400 MHz, $CDCl_3$) 4.1(d, *J* 7 Hz, 1H, H3a), 5.2(d, *J* 7.1 Hz, 1H, H6a), 6.1(s, 1H, H3), 7.1-8.17(m, 20H, Ar-H). ^{13}C{H}NMR spectrum: δ ppm(100.5 MHz, CDCl3) 56.3, 63 (3 Aliphatic **C**); 114.8, 119.1(3), 123.2, 124.5, 126.8(2), 127.2, 128.3(2), 128.6(2), 128.8(2), 129(3), 129.5(3), 131.2, 131.3, 132.2, 139.7, 155.86-158.38 (27 Aromatic **C**); 171.8, 172.9 (2 **C**=O). Anal. Calcd for ($C_{32}H_{23}FN_4O_3$) (%): C, 72.44; H, 4.37; N, 10.56. Found; C, 72.21;H, 4.24; N, 10.55.

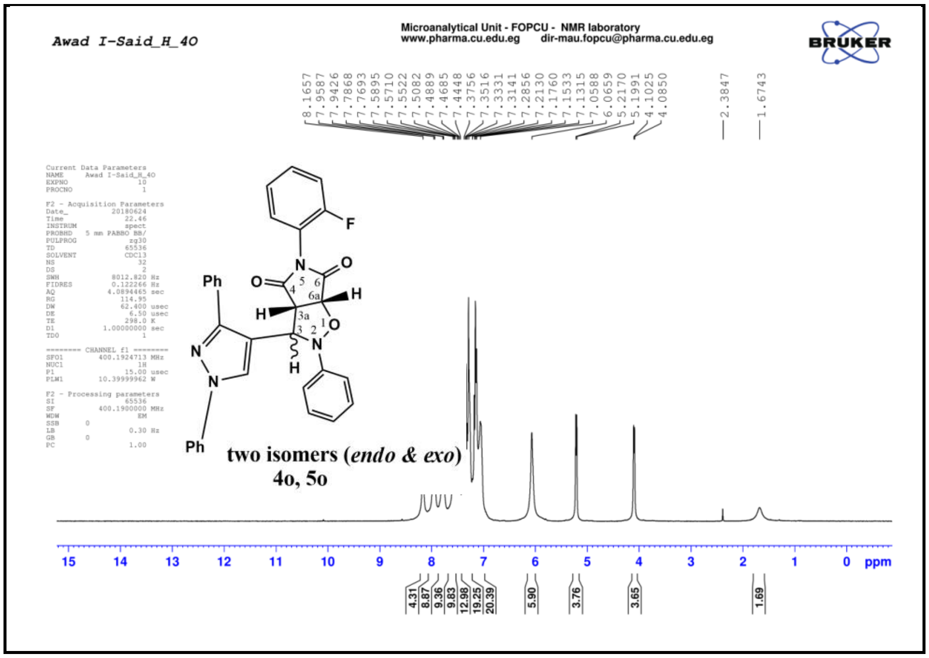

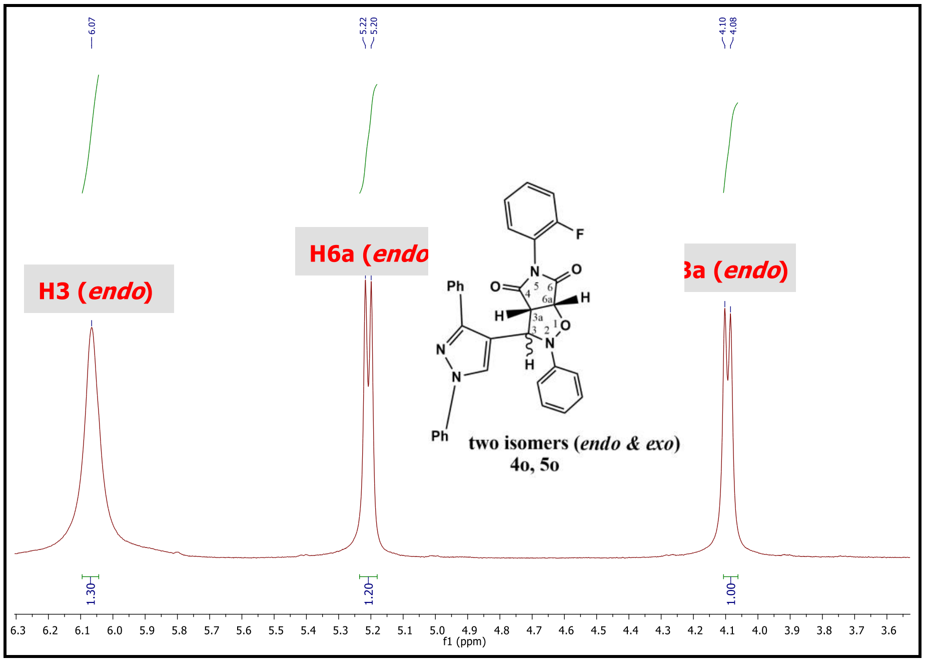

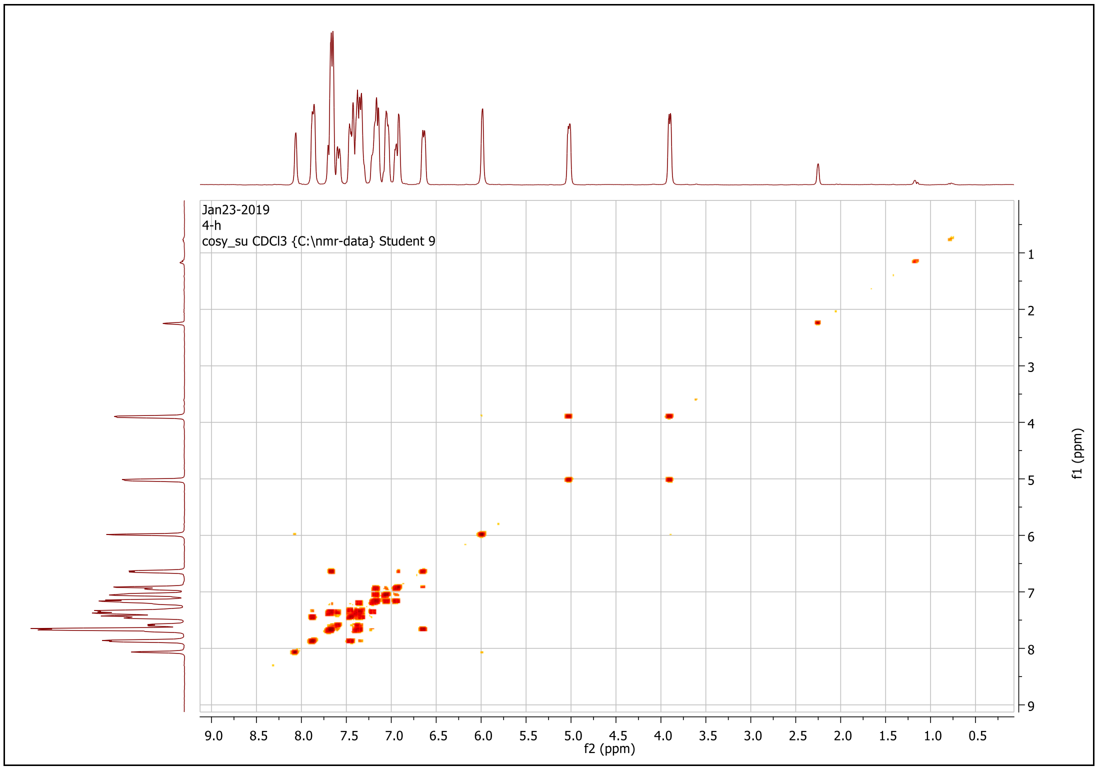

Jan23-2019
4-h
cosy_su CDCl3 {C:\nmr-data} Student 9
f2 (ppm)
f1 (ppm)

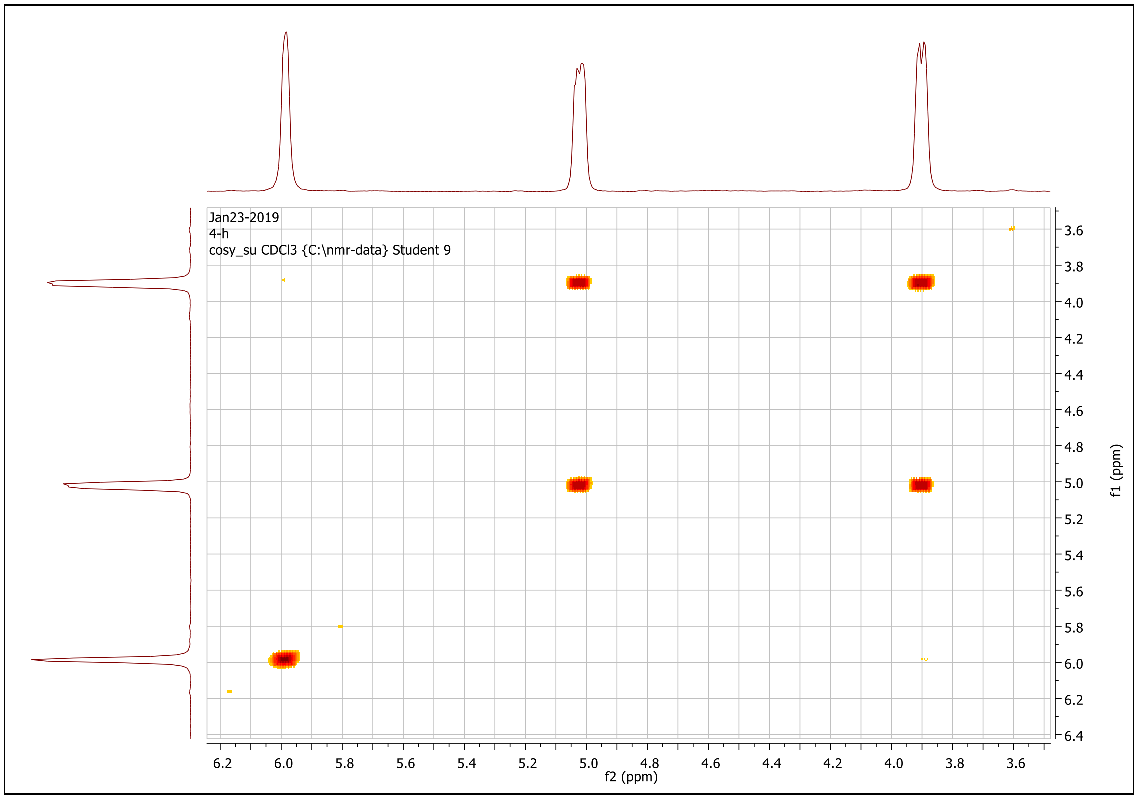

Jan23-2019
4-h
cosy_su CDCl3 {C:\nmr-data} Student 9
f2 (ppm)
f1 (ppm)

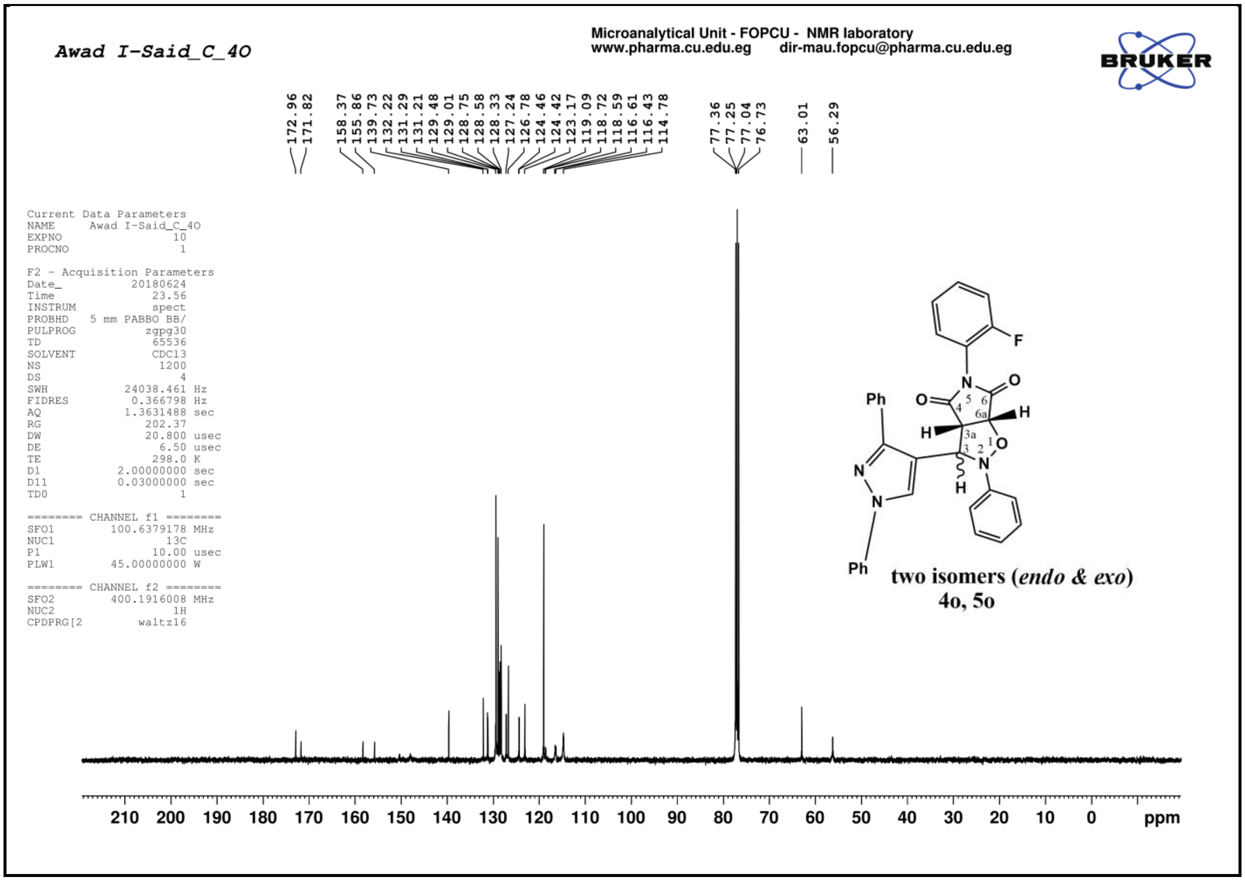

Awad I-Said_C_4O
Microanalytical Unit - FOPCU - NMR laboratory
www.pharma.cu.edu.eg dir-mau.fopcu@pharma.cu.edu.eg
BRUKER
172.96 171.82 158.37 155.86 139.73 132.22 131.29 131.21 129.48 129.01 128.75 128.58 128.33 127.24 126.78 124.46 124.42 123.17 119.09 118.72 118.59 116.61 116.43 114.78 77.36 77.25 77.04 76.73 63.01 56.29
Current Data Parameters
NAME Awad I-Said_C_4O
EXPNO 10
PROCNO 1
F2 - Acquisition Parameters
Date_ 20180624
Time 23.56
INSTRUM spect
PROBHD 5 mm PABBO BB/
PULPROG zgpg30
TD 65536
SOLVENT CDCl3
NS 1200
DS 4
SWH 24038.461 Hz
FIDRES 0.366798 Hz
AQ 1.3631488 sec
RG 202.37
DW 20.800 usec
DE 6.50 usec
TE 298.0 K
D1 2.00000000 sec
D11 0.03000000 sec
TD0 1
======= CHANNEL f1 =======
SFO1 100.6379178 MHz
NUC1 13C
P1 10.00 usec
PLW1 45.00000000 W
======= CHANNEL f2 =======
SFO2 400.1916008 MHz
NUC2 1H
CPDPRG[2 waltz16
two isomers (endo & exo)
4o, 5o
210 200 190 180 170 160 150 140 130 120 110 100 90 80 70 60 50 40 30 20 10 0 ppm

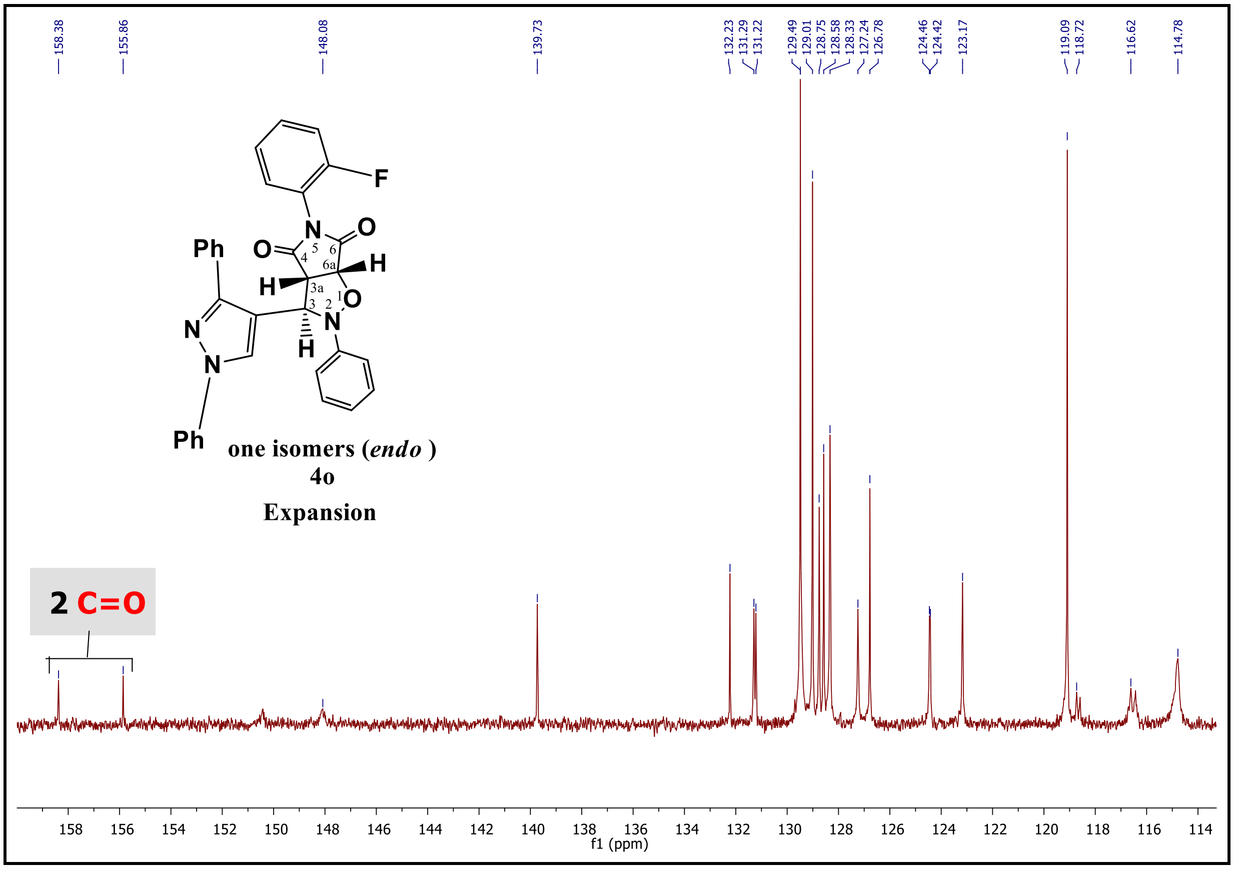

158.38 155.86 148.08 139.73 132.23 131.29 131.22 129.49 129.01 128.75 128.58 128.33 127.24 126.78 124.46 124.42 123.17 119.09 118.72 116.62 114.78
one isomers (endo)
4o
Expansion
2 C=O
158 156 154 152 150 148 146 144 142 140 138 136 134 132 130 128 126 124 122 120 118 116 114
f1 (ppm)

Exo-isomer **(5o)**: (0.08 g, 5.2 %); white crystals; mp: 158-160°C. Mass spectrum (electron impact): m/e (%) 535.1(7.3), 339.1(16.7), 190.9(5.52), 76.96(100).

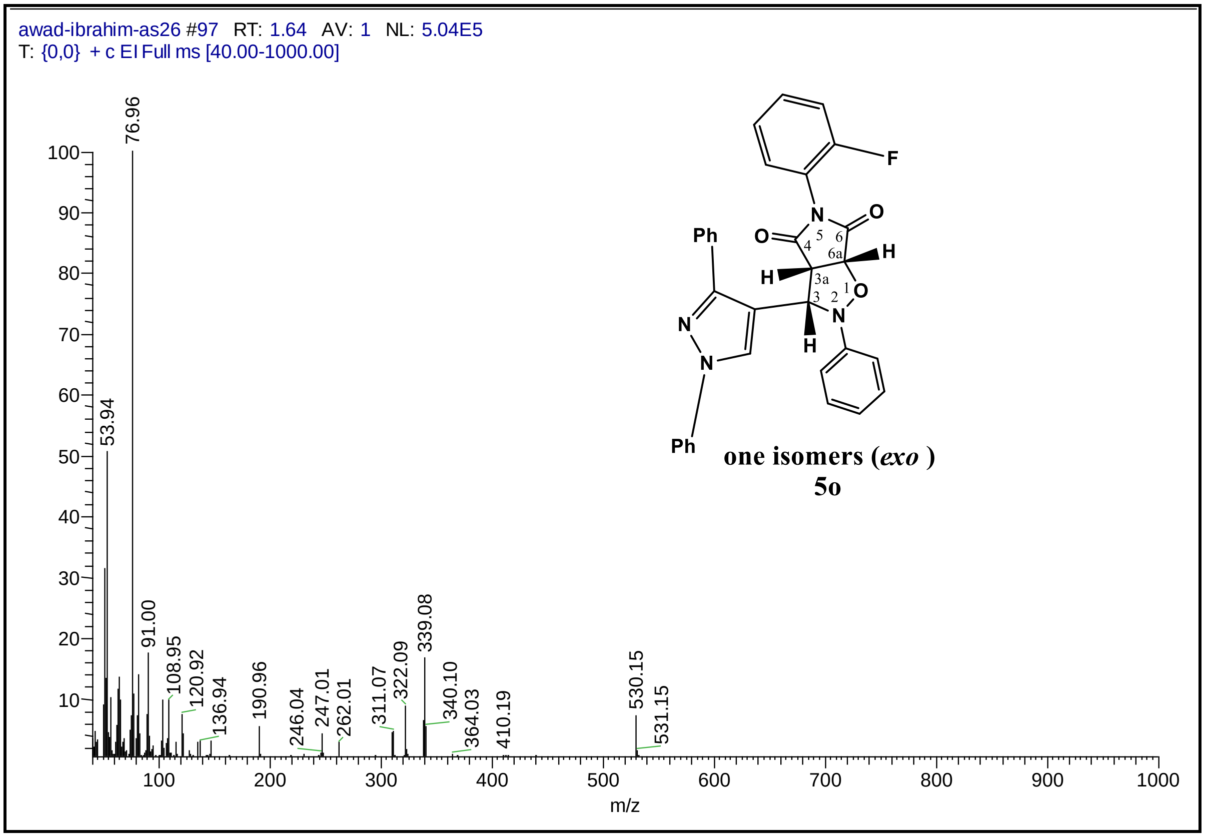

Computational calculations

Table S1. Cartesian coordinates (in Å) of the M062X/6-311G** optimized structure of the electronic ground state of 4**b**. (Total energy: RB3LYP/6-31G(d,p): -1717.85523 Hartree, 0 imaginary frequencies)

Atom	x	y	Z
C	-0.68134	0.07565	1.29984
C	0.45588	0.29967	0.45595
N	0.22814	1.66895	-0.13011
O	1.47802	1.70693	-0.47764
C	2.25842	1.82142	0.71081
C	1.78887	0.46042	1.246
C	3.45348	1.89221	-0.18514
N	3.67029	0.47701	-0.51848
C	2.84022	-0.28789	0.41116
O	2.53126	-1.57064	0.51945
C	-0.35237	1.53692	-1.47416
C	-0.17369	0.48223	-2.37674
C	-0.81668	0.53076	-3.61157
C	-1.53916	1.66959	-3.96631
C	-1.62493	2.75828	-3.10064
C	-1.00788	2.70452	-1.85844
C	4.40521	0.18068	-1.59671
O	3.57346	2.97831	-0.79974
C	-1.61057	-0.94593	1.42646
N	-2.3522	-0.60223	2.47365
N	-1.91381	0.63846	2.89491
C	-0.88674	1.09533	2.16675
C	5.83821	-0.22642	-1.53083
C	6.64635	-0.27024	-2.59823
C	6.10852	-0.03014	-3.9595
C	4.64223	0.1924	-4.12129
C	3.82723	0.26343	-2.88173
C	-1.67241	-2.12102	0.56501
C	-2.65741	-3.07538	0.81908
C	-2.67263	-4.19497	-0.0159
C	-1.77964	-4.38429	-1.07864
C	-0.80602	-3.44522	-1.36656
C	-0.80412	-2.36648	-0.50524
C	-2.39957	1.38114	3.93987
C	-1.81385	2.61843	4.25654
C	-2.32949	3.33247	5.32107
C	-3.40872	2.79423	6.02321
C	-3.9863	1.56751	5.68803
C	-3.48277	0.83323	4.6257
H	0.61645	-0.40098	-0.37868

H	2.12827	2.68288	1.31165
H	1.847	0.03121	2.29289
H	0.44824	-0.40459	-2.19779
H	-0.73368	-0.32205	-4.3074
H	-2.03911	1.71996	-4.94561
H	-2.1947	3.6609	-3.36052
H	-1.05963	3.5445	-1.14479
H	-0.30652	2.02492	2.24291
H	7.72454	-0.46043	-2.41786
H	4.17194	0.40752	-5.10216
H	2.77886	0.50153	-3.13235
H	-3.37112	-2.9253	1.66029
H	-3.43044	-4.96077	0.18067
H	-1.7815	-5.2655	-1.73808
H	-0.0425	-3.48978	-2.17136
H	0.04365	-1.79855	-0.85415
H	-0.95869	3.0701	3.72252
H	-1.86225	4.29761	5.5758
H	-3.81273	3.36407	6.8683
H	-4.83899	1.14775	6.24104
H	-3.92058	-0.13953	4.33086
H	6.38105	-0.41397	-0.58144
C	7.0587	-0.09541	-5.16966
H	6.53042	-0.47846	-6.01766
H	7.88248	-0.7392	-4.94201
H	7.42338	0.88606	-5.39015

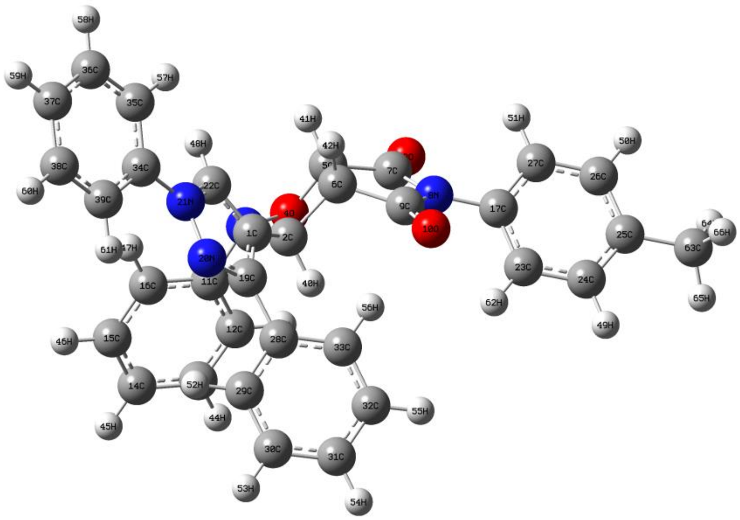

**Table S2.** Cartesian coordinates (in Å) of the M062X/6-311G** optimized structure of the electronic ground state of **5b**. (Total energy: RB3LYP/6-31G(d,p): -1717.85479Hartree, 0 imaginary frequencies)			
Atom	x	y	z
C	-0.73488	0.23485	0.87876
C	0.34846	0.47963	-0.13581
N	-0.09025	0.12379	-1.50462
O	0.84127	-0.34732	-2.30435
C	1.82294	-0.92043	-1.48069
C	1.54416	-0.49162	-0.03985
C	1.68855	-2.43771	-1.45082
N	1.12613	-2.90388	-0.25271
C	1.27938	-1.81703	0.66614
O	1.12386	-1.84238	1.96627
C	-1.31419	0.63868	-1.9997
C	-1.83939	0.05919	-3.16988
C	-3.0525	0.48787	-3.70935
C	-3.77046	1.51321	-3.09229
C	-3.25802	2.11464	-1.94187
C	-2.0427	1.68539	-1.40705
O	2.82956	-2.94971	-1.59054
C	-1.16083	0.998	1.99256
N	-2.14751	0.33295	2.58629
N	-2.41516	-0.82138	1.902
C	-1.5519	-0.88352	0.85221
C	-0.70158	2.29578	2.53905
C	-0.94273	2.57903	3.89445
C	-0.57802	3.80055	4.46288
C	0.03712	4.78005	3.68243
C	0.27406	4.52431	2.33253
C	-0.09796	3.29991	1.77108
C	-3.43088	-1.75805	2.26791
C	-3.63716	-2.9532	1.55679
C	-4.6379	-3.84975	1.93404
C	-5.4593	-3.56738	3.02675
C	-5.26867	-2.38533	3.74412
C	-4.26275	-1.49334	3.37028
H	0.73136	1.51889	-0.176
H	2.83576	-0.62544	-1.83669
H	2.4245	-0.02087	0.4554
H	-1.3076	-0.76655	-3.67025
H	-3.45014	0.00857	-4.61847
H	-4.73122	1.84937	-3.51354
H	-3.81511	2.93305	-1.45692
H	-1.67377	2.20297	-0.51137

H	-1.54187	-1.70289	0.12713
H	-1.43053	1.8365	4.54688
H	-0.78612	3.99879	5.52732
H	0.31155	5.75254	4.12234
H	0.72808	5.30537	1.70038
H	0.04731	3.1866	0.68978
H	-3.01443	-3.21645	0.68852
H	-4.78233	-4.78392	1.36712
H	-6.25247	-4.2732	3.3215
H	-5.91543	-2.15683	4.60698
H	-4.14247	-0.56888	3.95738
C	0.48905	-4.21161	-0.04089
C	0.77216	-4.94714	1.11034
C	-0.39855	-4.71711	-0.99072
C	0.16828	-6.18817	1.31128
H	1.47257	-4.54869	1.85861
C	-1.00346	-5.95806	-0.7894
H	-0.62194	-4.13745	-1.89804
H	0.62435	-6.90955	2.04551
H	-1.93606	-6.2149	-1.36546
C	-0.7201	-6.69368	0.3613
C	-1.38761	-8.06362	0.58334
H	-2.27648	-8.12744	-0.00889
H	-1.63929	-8.17432	1.61742
H	-0.71085	-8.84092	0.29578

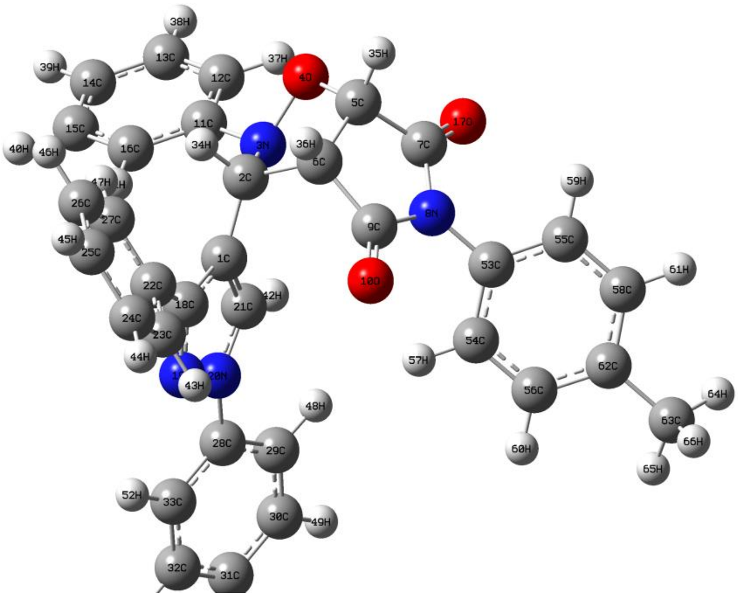

Table S3. Cartesian coordinates (in Å) of the M062X/6-311G** optimized structure of the electronic ground state of 4**k**. (Total energy: RB3LYP/6-31G(d,p): -1717.85467 Hartree, 0 imaginary frequencies)

Atom	x	y	z
C	1.50897	-1.37734	0.15804
C	0.28344	-0.62164	-0.25509
N	0.59502	0.30556	-1.40023
O	-0.73919	0.63467	-1.91559
C	-1.33711	-0.647	-2.0756
C	-0.8629	-1.4742	-0.87556
C	-2.84634	-0.48008	-1.8929
N	-3.19108	-1.05061	-0.6576
C	-2.09179	-1.61291	0.0127
O	-2.14094	-2.12542	1.11183
C	1.22012	1.54286	-1.00981
C	0.49199	2.63193	-0.5142
C	1.16196	3.79764	-0.14407
C	2.55289	3.87751	-0.24662
C	3.2744	2.78828	-0.73831
C	2.61015	1.62668	-1.13373
C	-4.51967	-1.02935	-0.12023
O	-3.61119	0.06018	-2.65961
C	2.13135	-1.4717	1.44101
N	3.26912	-2.1695	1.36652
N	3.39398	-2.52753	0.06398
C	2.35794	-2.0651	-0.68858
C	-4.77821	-1.61888	1.11962
C	-6.07026	-1.58744	1.63877
C	-7.12411	-0.98731	0.93594
C	-6.84005	-0.40485	-0.30641
C	-5.55131	-0.41739	-0.83571
C	1.70988	-0.908	2.73953
C	2.67006	-0.33423	3.59021
C	2.29784	0.19477	4.82406
C	0.96059	0.16154	5.2283
C	0.00154	-0.41457	4.39408
C	0.37041	-0.95096	3.15919
C	4.51637	-3.28641	-0.3658
C	4.45006	-4.03674	-1.54503
C	5.56733	-4.75816	-1.96429
C	6.74058	-4.75031	-1.20831
C	6.79092	-4.0113	-0.0241
C	5.68703	-3.27493	0.40048
H	-0.10115	-0.04613	0.59553
H	-1.10095	-1.08349	-3.05194

H	-0.5146	-2.47224	-1.14945
H	-0.5871	2.5661	-0.44072
H	0.59458	4.64472	0.2301
H	3.06957	4.78618	0.04711
H	4.35473	2.84595	-0.83097
H	3.15664	0.78377	-1.54161
H	2.31752	-2.20273	-1.75752
H	-6.26205	-2.04112	2.60724
H	-7.63771	0.07284	-0.86858
H	3.70615	-0.30318	3.26968
H	3.05095	0.63852	5.4688
H	0.67032	0.57791	6.18848
H	-1.03755	-0.45877	4.70703
H	-0.37823	-1.43299	2.53858
H	3.53295	-4.07647	-2.1222
H	5.51126	-5.33854	-2.88003
H	7.60533	-5.31849	-1.53606
H	7.69763	-4.0002	0.573
H	5.71473	-2.69405	1.31375
H	-3.97514	-2.08719	1.67629
C	-5.26322	0.23433	-2.20095
H	-4.69509	-0.44114	-2.80583
H	-4.70692	1.13665	-2.05515
H	-6.18747	0.46029	-2.69044
H	-8.11628	-0.97437	1.33633

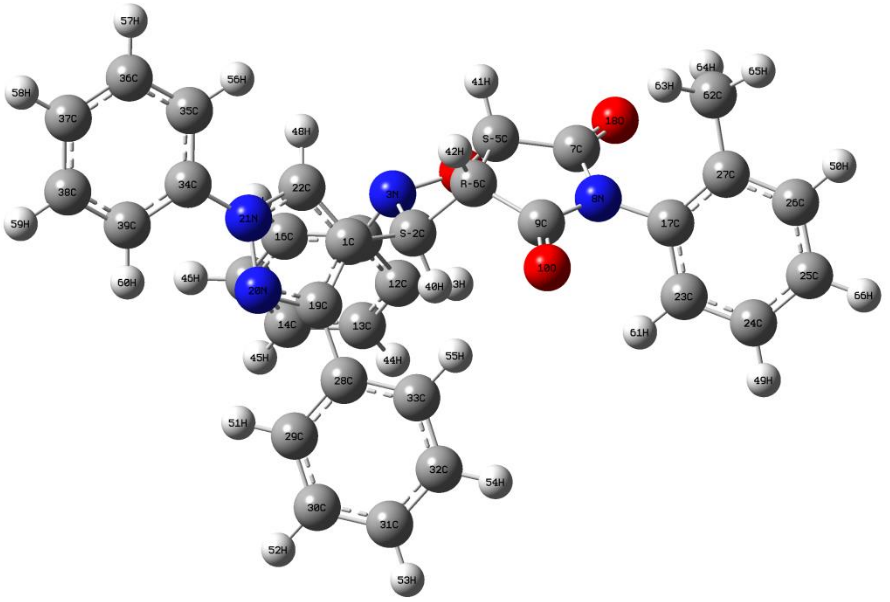

Table S4. Cartesian coordinates (in Å) of the M062X/6-311G** optimized structure of the electronic ground state of **5k**. (Total energy: RB3LYP/6-31G(d,p): -1717.85396Hartree, 0 imaginary frequencies)

Atom	x	y	z
C	-1.22594	0.60944	-1.34384
C	-1.45886	-0.87126	-1.26483
N	-1.32669	-1.36213	0.12812
O	-1.14002	-2.80956	-0.02974
C	-0.13992	-2.8886	-1.02229
C	-0.40054	-1.75507	-2.02276
C	1.26533	-2.59898	-0.46363
N	1.8459	-1.602	-1.26035
C	0.95889	-1.10086	-2.23301
O	1.2506	-0.29376	-3.08765
C	-2.39044	-1.17176	1.05529
C	-2.37265	-1.92396	2.24247
C	-3.36251	-1.73699	3.20164
C	-4.37334	-0.79064	3.00703
C	-4.38405	-0.03819	1.83436
C	-3.40681	-0.22795	0.85388
O	1.78576	-3.14519	0.48316
C	-1.66187	1.53931	-2.33715
N	-1.14425	2.75009	-2.11033
N	-0.38517	2.62039	-0.99533
C	-0.41158	1.34853	-0.50926
C	-2.51451	1.32576	-3.52594
C	-2.15977	1.91877	-4.74914
C	-2.95331	1.741	-5.88091
C	-4.11355	0.96591	-5.81275
C	-4.48024	0.37865	-4.60076
C	-3.69005	0.55993	-3.46534
C	0.29913	3.74393	-0.45621
C	1.44383	3.55677	0.32623
C	2.09455	4.66589	0.86534
C	1.62315	5.95543	0.61367
C	0.49035	6.13155	-0.18432
C	-0.17906	5.03208	-0.718
H	-2.44107	-1.13644	-1.67339
H	-0.1742	-3.89914	-1.43571
H	-0.79733	-2.06776	-2.98887
H	-1.58557	-2.6529	2.39344
H	-3.33988	-2.33107	4.11051
H	-5.13982	-0.64283	3.76132
H	-5.16159	0.70124	1.66688
H	-3.43129	0.37544	-0.0445

H	0.11054	1.06148	0.38896
H	-1.25338	2.51247	-4.80067
H	-2.66203	2.20356	-6.81938
H	-4.72872	0.82405	-6.69631
H	-5.38856	-0.21287	-4.53385
H	-4.00958	0.12781	-2.52228
H	1.83974	2.56095	0.49283
H	2.98267	4.51757	1.47178
H	2.13711	6.81566	1.03084
H	0.11753	7.13066	-0.38818
H	-1.06238	5.1536	-1.33262
C	3.18779	-1.12945	-1.0912
C	3.68841	-0.12652	-1.925
C	3.9921	-1.67113	-0.08437
C	4.99143	0.33182	-1.74197
C	5.29607	-1.20759	0.07694
H	3.60321	-2.45128	0.55874
H	5.37032	1.11769	-2.38961
H	5.91534	-1.63479	0.86104
C	5.82004	-0.20107	-0.74558
C	2.79623	0.47819	-3.02497
H	2.20353	1.26657	-2.61017
H	2.1539	-0.28066	-3.42052
H	6.82259	0.14987	-0.61656
H	3.41136	0.86882	-3.8085

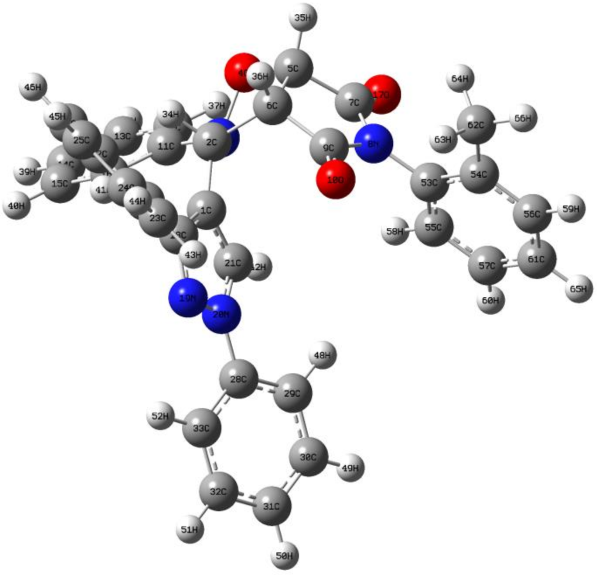

**Table S5. The energy (Hatree) as a function of the (C6-N5-C1'-C6') dihedral angle in *endo* and *exo* isomers with either *para* or *ortho* methyl substitution.**				
Dihedral Angle	Total Energy (Hartree)			
	Endo (*ortho*)	Endo (*para*)	Exo (*ortho*)	Exo (*para*)
0	-1717.83094	-1717.85035	-1717.83618	-1717.85069
10	-1717.82407	-1717.85088	-1717.84115	-1717.85166
20	-1717.84122	-1717.85242	-1717.84536	-1717.85301
30	-1717.84611	-1717.85375	-1717.84862	-1717.85412
40	-1717.84988	-1717.85461	-1717.85092	-1717.85473
50	-1717.85249	-1717.85496	-1717.8522	-1717.85476
60	-1717.85395	-1717.85485	-1717.85279	-1717.85435
70	-1717.85441	-1717.8546	-1717.85286	-1717.85384
80	-1717.8543	-1717.85432	-1717.85274	-1717.85332
90	-1717.85416	-1717.85423	-1717.85289	-1717.85325
100	-1717.85429	-1717.85436	-1717.85335	-1717.85344
110	-1717.85455	-1717.85464	-1717.8538	-1717.85398
120	-1717.85454	-1717.85504	-1717.85367	-1717.85451
130	-1717.85394	-1717.85523	-1717.85266	-1717.85479
140	-1717.85249	-1717.85505	-1717.85041	-1717.85461
150	-1717.84998	-1717.85434	-1717.84668	-1717.85375
160	-1717.84635	-1717.8529	-1717.84137	-1717.85252
170	-1717.8415	-1717.85126	-1717.83477	-1717.85119
180	-1717.83552	-1717.85033	-1717.82787	-1717.85065
190	-1717.82888	-1717.85093	-1717.83666	-1717.85151
200	-1717.84472	-1717.85243	-1717.84284	-1717.85292
210	-1717.84868	-1717.85381	-1717.84766	-1717.85409
220	-1717.85152	-1717.85463	-1717.85097	-1717.85473
230	-1717.8533	-1717.85495	-1717.85292	-1717.85475
240	-1717.8542	-1717.85485	-1717.85377	-1717.85436
250	-1717.85444	-1717.85459	-1717.85378	-1717.85384
260	-1717.85432	-1717.85431	-1717.85334	-1717.85333
270	-1717.85427	-1717.85422	-1717.85317	-1717.85325
280	-1717.85438	-1717.85435	-1717.85348	-1717.85344
290	-1717.85467	-1717.85463	-1717.85392	-1717.85396
300	-1717.85447	-1717.85503	-1717.85396	-1717.85449
310	-1717.85341	-1717.85523	-1717.85331	-1717.85478
320	-1717.85114	-1717.85506	-1717.85177	-1717.85461
330	-1717.8477	-1717.85436	-1717.84913	-1717.85383
340	-1717.8431	-1717.85306	-1717.84543	-1717.85257
350	-1717.8374	-1717.85147	-1717.84091	-1717.85125
360	-1717.83094	-1717.85035	-1717.83572	-1717.85069

References:

1) M. P. Cava, A. A. Deana, K. Muth and A. J. Mitechell, *Org. Synth.* 1961, **41**, 93.
